# From gut to proteomics: the impact of *Roseburia intestinalis* on post-translational modifications in colorectal cancer

**DOI:** 10.3389/fonc.2025.1599183

**Published:** 2025-08-18

**Authors:** Lu Li, Yong Zhang, Qianqian Wu, Ziyu Jin, Hongbing Xia, Huili Wu, Kunkun Li, Lihong Wang

**Affiliations:** ^1^ Department of Gastroenterology, Zhengzhou Central Hospital Affiliated to Zhengzhou University, Zhengzhou, China; ^2^ Medical Key Laboratory for Diagnosis and Treatment of Colorectal Cancer in Henan Province, Zhengzhou, China; ^3^ Endoscopy Department, Yuzhou Traditional Chinese Medicine Hospital, Xuchang, Henan, China

**Keywords:** colorectal cancer, *Roseburia intestinalis*, global proteomics, acetylation, lactylation

## Abstract

**Background:**

Colorectal cancer (CRC) represents a significant global health challenge. Gut microbiota imbalance and abnormal chromatin modifications play critical roles in the progression of CRC. However, the mechanisms by which they exert their influences, particularly the involvement of *Roseburia intestinalis* (*R.i*)-mediated post-translational modifications (PTMs), remain inadequately understood. This study aims to explore global acylase change map induced by *R.i* in CRC cells.

**Methods:**

The 4D-Fast DIA quantitative acetylated modified proteome and the 4D-Fast DIA quantitative lactated modified proteome combined with proteomics were used to detect CRC cells (HCT116) co-cultured with *R.i*. Cell proliferation was evaluated by the Cell Counting Kit-8 (CCK-8) assay and colony formation assay.

**Results:**

In this study, we verified elevated levels of lactylation in CRC tumor tissues and cells. Intervention with *R.i* was shown to induce a reduction in cellular lactylation levels while increasing acetylation levels. A total of 6,134 acetylation (Kac) sites were identified across 3,037 acetylated proteins, and 7,882 lactylation (Lac) sites were identified among 2,386 lactylated proteins. Notably, the subcellular distribution of proteins modified at Kac and Lac sites exhibited distinct patterns. Additionally, there were differences in specific sequence motifs surrounding acetylated or lactylated lysine residues. To further investigate the differentially expressed proteins involved in Kac and Lac modifications, we conducted enrichment analyses using Biological Process and the Kyoto Encyclopedia of Genes and Genomes. The acetylome profiling identified significant enrichment of differentially expressed proteins in several critical metabolic pathways, including: Glycolysis, Lipid metabolism, Pyruvate metabolism, Glycerophospholipid metabolism. Concurrently, lactylome analysis demonstrated distinct protein enrichment in: Glycolysis, Galactose metabolism, Pentose phosphate pathway, Non-homologous end-joining. Notably, glycolysis emerged as the principal convergent pathway between acetylation and lactylation modifications, suggesting its central regulatory role in metabolic reprogramming under these PTMs.

**Conclusions:**

Our study reveals a previously unrecognized mechanism by which *R.i* orchestrates metabolic-translational post-translational modification crosstalk in CRC through bidirectional modulation of protein markers. These findings offer insights into the underlying mechanisms that may influence CRC progression.

## Introduction

Recent epidemiological findings reveal that colorectal cancer (CRC) ranks as the third most prevalent cancer and the third highest contributor to cancer mortality among both males and females worldwide in 2022 (1). Given the high prevalence and poor outcome, CRC is a global issue impacting public health. The incidence of CRC has been increasing among young individuals, especially in recent years (2). Therefore, investigating the molecular pathways involved in the advancement is essential in order to develop efficient early detection methods and treatment interventions for CRC.

The gut microbiome has been implicated in the onset and advancement of CRC, as well as in the effectiveness of treating the disease. And microbiota patterns have been strongly associated with CRC (3). Specific intestinal microbes and their derivatives affect the development of CRC via host metabolism, immune function, host/microbial sensing pathways, and so on (4). Intestinal dysbiosis is characterized by an increased abundance of pathogenic bacteria and a decrease in beneficial bacteria, especially SCFA-producing bacteria (5, 6). Consistent with our previous findings (7), *Roseburia* has been observed to be depleted in colorectal cancer (8, 9). *Roseburia intestinalis* (*R.i*), an anaerobic, Gram-positive organism, belongs to one of the species of the *Roseburia* genus (10). Recent studies demonstrate that gut microbiota-derived metabolites, particularly butyrate, serve as key mediators between microbial activity and host epigenetic reprogramming. Butyrate, a primary metabolite of *R.i*, acts as an HDAC inhibitor, elevating global histone acetylation (Kac) by blocking deacetylase activity, thereby promoting an open chromatin state at tumor suppressor loci (11). As of yet, the mechanism of *R.i* in CRC has not been thoroughly examined.

Post-translational modifications regulate the localization, function and mechanism of proteins, which is an important regulatory mechanism in cell biology (12, 13). There is a growing body of evidence that PTMs, such as acylation, ubiquitination, methylation, phosphorylation, and so on, play an important role in tumorigenesis (14, 15). In colorectal cancer, the modulation of histone acetylation levels is closely linked to tumor progression (16). Histone acetylation represents a critical epigenetic modification that regulates gene expression by altering chromatin structure (17, 18). Specifically, acetylation can enhance the transcriptional activity of genes, thereby inhibiting the expression of oncogenes (19). Additionally, the aberrant expression of histone deacetylases (HDACs) has been associated with cancer development and progression (20). HDACs remove acetyl groups from histones, leading to chromatin condensation and transcriptional silencing of genes (21). In colorectal cancer, the overexpression of HDACs may contribute to the silencing of tumor suppressor genes, thereby promoting oncogenesis (22). Consequently, HDAC inhibitors have emerged as a potential therapeutic strategy and have been extensively studied for their antitumor effects. These inhibitors function by blocking HDAC activity, thus restoring histone acetylation levels and reactivating silenced tumor suppressor genes (23). In summary, the interplay between histone acetylation and deacetylation plays a pivotal role in the initiation and progression of colorectal cancer. Modulating these epigenetic modifications may provide novel therapeutic avenues for cancer treatment, with agents targeting histone acetylation likely to play a significant role in future oncological therapies (24).

Lactylation, defined as the covalent modification of proteins through the esterification of lactate with amino acid residues, significantly alters the structural and functional dynamics of these proteins (25). Within the tumor microenvironment, lactate is not merely a byproduct of metabolism; rather, it plays multifaceted roles in tumor progression (26). The accumulation of lactate is often closely associated with metabolic reprogramming within cancer cells (27). In colorectal cancer, lactate serves not only as an alternative energy source but also enhances tumor cell proliferation and migration by modulating the activity of critical metabolic enzymes and signaling molecules (28). Moreover, lactate accumulation leads to the acidification of the tumor microenvironment, which facilitates tumor cell invasion and metastasis while concurrently suppressing the activity of immune cells, thereby enabling tumor cells to evade immune surveillance (29, 30). In the immunosuppressive tumor microenvironment, PD-L1 lactylation promotes immune evasion by stabilizing PD-L1 and inhibiting T cell function. Blocking PD-1/PD-L1 signaling restores CD8+ T cell activity, particularly under serine/glycine restriction (31). Notably, metastatic CRC cells exhibit elevated H3K18la levels, which recruit hepatocyte nuclear factors (FOXA2/HNF1A) to activate liver-specific genes, facilitating hepatic metastasis. Lactylation also modifies non-histone proteins such as PKM2 (K62la), enhancing its nuclear translocation to transcriptionally upregulate glycolytic enzymes (LDHA, HK2), thereby creating a feed-forward loop to sustain lactate production (32). Consequently, the role of lactate in the tumor microenvironment has emerged as a focal point of current research, and strategies targeting lactate metabolism may offer promising avenues for therapeutic interventions in oncology. However, the overall lactate group and acetylation of CRC cells regulated by *R.i* have not been reported.

This study established an elevated level of lactylation in tumor tissues from patients with colorectal cancer. Notably, CRC cells co-cultured with *R.i* exhibited a significant increase in acetylation levels, accompanied by a suppression of lactylation levels. Employing 4D-FastDIA for quantitative proteomics, both for lactylated (Kla) and acetylated (Kac) proteins, we reported the dynamic changes induced by *R.i* in CRC. These findings provide a valuable foundation for further investigations into the role of *R.i* in CRC, highlighting the interplay between lactylation and acetylation modifications in tumor biology. The data generated may provide new insights into novel therapeutic strategies targeting these epigenetic modifications in CRC treatment.

## Materials and methods

### Tissue specimens

Participants were recruited from patients diagnosed with colorectal cancer through endoscopic examination and pathological assessment at Zhengzhou Central Hospital Affiliated to Zhengzhou University in 2024. Exclusion criteria included patients with recurrent gastric cancer or secondary malignancies, as well as those undergoing chemotherapy, immunotherapy, or radiotherapy. Ultimately, patients with complete information were selected for the study. Participant clinical data are provided in **Supplementary Table 1**. The procedures used in this study adhere to the tenets of the Declaration of Helsinki and have been approved by the Institutional Review Board (Ethical Batch Number: ZXYY2024278). Informed consent was obtained from all the participants. Tumor and paired normal tissues were collected and promptly frozen in a -80°C refrigerator.

### Cell culture and co-cultivation with bacteria

The CRC cell lines HCT116 and SW480 were purchased from Procell Life Science Technology. They were routinely monitored by PCR to ensure they were mycoplasma-free and authenticated by short tandem repeat profiling. HCT116 cells in McCoy’s 5A medium, SW480 cells in Iscove’s Modified Dulbecco’s Medium. All the cells were cultured under standard incubator conditions (37°C, 5% CO_2_) and passaged at approximately 90% confluence. We obtained *Roseburia intestinalis* from Ningbo Mingzhou Biotechnology Co., Ltd., with a concentration of 10^9^ colony-forming units (CFU)/mL (63, 64). The *Roseburia intestinalis* and HCT116 cells were co-cultured for 24 hours and the cells were collected for follow-up experiments.

### Quantitative proteomics of modified proteins using 4D-fast DIA

Samples were retrieved from -80°C and each group of samples was mixed with four volumes of lysis buffer (8 M urea, 1% protease inhibitor, 3 μM TSA, and 50 mM NAM) and subjected to ultrasonic lysis. The lysate was then centrifuged at 4°C and 12,000 g for 10 minutes to remove cell debris. The supernatant was transferred to a new centrifuge tube, and the protein concentration was determined using a BCA assay kit.

Equal amounts of protein from each sample were subjected to enzymatic digestion. The volume was adjusted to uniformity using lysis buffer, and 20% TCA was slowly added to achieve a final concentration of 20%. The mixture was vortexed and allowed to precipitate at 4°C for 2 hours. The samples were then centrifuged at 4,500 g for 5 minutes to discard the supernatant, and the precipitate was washed 2–3 times with pre-cooled acetone. After air-drying the precipitate, 200 mM TEAB was added, and the precipitate was sonicated to disperse it. Trypsin was added at a 1:50 ratio (w/w, enzyme:protein), and digestion was allowed to proceed overnight. Dithiothreitol (DTT) was then added to achieve a final concentration of 5 mM, followed by reduction at 56°C for 30 minutes. Subsequently, iodoacetamide (IAA) was added to achieve a final concentration of 11 mM, and the mixture was incubated in the dark at room temperature for 15 minutes.

The peptide segments were dissolved in immunoprecipitation (IP) buffer (100 mM NaCl, 1 mM EDTA, 50 mM Tris-HCl, 0.5% NP-40, pH 8.0) and the supernatant was transferred to pre-washed resin. The mixture was placed on a rotating shaker at 4°C and gently shaken for overnight incubation. After incubation, the resin was washed four times with IP buffer and twice with deionized water. Finally, the elution was performed using 0.1% trifluoroacetic acid (TFA) elution buffer, and the peptides bound to the resin were eluted three times. The elution fractions were collected and vacuum freeze-dried. After drying, the samples were desalted according to the instructions provided with the C18 ZipTips and subsequently vacuum freeze-dried before analysis by liquid chromatography-mass spectrometry (LC-MS).

The peptides were dissolved in mobile phase A and separated using the NanoElute ultra-high-performance liquid chromatography (UHPLC) system. Mobile phase A consisted of an aqueous solution containing 0.1% formic acid and 2% acetonitrile, while mobile phase B was an acetonitrile-water solution containing 0.1% formic acid. The liquid chromatography gradient was set as follows: from 0 to 18 minutes, 6%-22% B; from 18 to 22 minutes, 22%-30% B; from 22 to 26 minutes, 30%-80% B; and from 26 to 30 minutes, 80% B, with a flow rate maintained at 450 nl/min. After separation via the ultra-high-performance liquid chromatography system, the peptides were injected into a capillary ion source for ionization and subsequently analyzed by the timsTOF Pro 2 mass spectrometer for data acquisition. The ion source voltage was set to 1.65 kV, and both the peptide precursor ions and their fragment ions were detected and analyzed using TOF. The data acquisition mode utilized was data-independent parallel accumulation serial fragmentation (dia-PASEF), with the first mass spectrum scan range set from 100 to 1700 m/z. Following the acquisition of a single first mass spectrum, eight acquisitions in PASEF mode were performed, with the second mass spectrum scan covering a range of 425-1025, using a 25 m/z window.

Using the MoMo tool based on the Motif-X algorithm to analyze the motif characteristics of modification sites. The analysis was based on peptide sequences composed of 10 amino acids upstream and downstream of all potential modification sites within the species. A feature sequence was considered a motif of modified peptides if the quantity of peptides exhibiting this sequence exceeded 20 and the statistical significance (*P*-value) was less than 0.000001. The relative quantification ratio of modification sites between two groups of samples was used as the fold change, with a threshold of greater than 1.5 for significant up-regulation and less than 1/1.5 for significant down-regulation.

### 4D-fast DIA quantitative proteomics

The peptides were dissolved in mobile phase A and separated using the NanoElute ultra-high-performance liquid chromatography (UHPLC) system. Mobile phase A comprised an aqueous solution containing 0.1% formic acid and 2% acetonitrile, while mobile phase B consisted of an acetonitrile-water solution containing 0.1% formic acid. The liquid chromatography gradient was configured as follows: from 0 to 14 minutes, 6%-24% B; from 14 to 16 minutes, 24%-35% B; from 16 to 18 minutes, 35%-80% B; and from 18 to 20 minutes, 80% B, with a flow rate maintained at 500 nl/min. After separation via the ultra-high-performance liquid chromatography system, the peptides were injected into a capillary ion source for ionization and subsequently analyzed by the timsTOF Pro 2 mass spectrometer for data acquisition. The ion source voltage was set to 1.75 kV, and both the peptide precursor ions and their fragment ions were detected and analyzed using TOF. The data acquisition mode utilized was data-independent parallel accumulation serial fragmentation (dia-PASEF), with the first mass spectrum scan range set from 300 to 1500 m/z. After acquiring a single first mass spectrum, twenty acquisitions in PASEF mode were performed, with the second mass spectrum scan covering a range of 400-850, using a 7 m/z window.

For proteomic analysis, the raw LC-MS datasets were first searched against database and converted into matrices containing normalized intensity (the raw intensity after correcting the sample/batch effect) of proteins. The normalized intensity (I) was transformed to the relative quantitative value (R) after centralization. The formula is listed as follow: Rij= Iij/Mean(Ij), where i represents sample and j represents protein. For post-translational modification omics, firstly, the intensities of modified peptides (I) were centralized and transformed into relative quantitative values (R) of modified peptides in each sample. Then, the relative quantitative value of the modified peptide was divided by the relative quantitative value of corresponding protein to remove the influence from protein expression of modifications (65).

Differentially expressed proteins (DEPs) with a fold change ≥1.5 were mapped to the STRING database to extract high-confidence interactions (confidence score >0.7). The PPI network was visualized using the R package “visNetwork”. In the network, nodes represent DEPs, with colors indicating expression changes (blue: downregulated; red: upregulated) and intensity reflecting fold-change magnitude. Node size corresponds to the number of interacting partners. The proteomics data generated by mass spectrometry have been deposited in the ProteomeXchange Consortium (http://proteomecentral.proteomexchange.org) through the iProX partner repository with the accession number PXD066351.

### Western blotting

An aliquot of 20 μg of protein sample was mixed with 4× sample buffer and diluted to 1×. An appropriate volume of protein lysis buffer was then added to achieve a final protein concentration of 1–2 mg/mL. The mixture was heated at 95°C for 10 minutes. Equal volumes of the sample and 20% pre-stained protein marker were loaded. Electrophoresis was conducted at a constant voltage of 80 V for 30 minutes, followed by an increase to 120 V until the bromophenol blue dye exited the separating gel. The NC membrane was equilibrated by immersing it in pre-chilled transfer buffer for 30 minutes. The transfer apparatus was assembled with the correct electrode orientation and placed in a 4°C environment, applying a constant current of 200 mA for 1 hour for the transfer process. The membrane was blocked with 5% non-fat dry milk prepared in 1× TBST at room temperature for 1 hour. The membrane was rinsed three times with TBST, each rinse lasting 10 minutes. The primary antibody was diluted in TBST containing 2.5% BSA and incubated overnight on a roller at 4°C. Afterward, a secondary antibody was applied and incubated at room temperature for 1 hour. A chemiluminescent HRP substrate was added and incubated for 2 minutes, followed by signal capture according to the operating instructions of the chemiluminescent imaging system.

### CCK-8 cell viability assays

The viability of cells was determined with Cell Counting Kit-8 (CCK-8). At a density of 2000 cells per well, cells were seeded into 96-well plates. Six replicate wells were set up for each group. The medium was replaced with 100μl fresh complete medium containing 10μl CCK8 solution following a culture of 0, 24, 48 and 72h, respectively. Then, the plates were incubated at 37°C and 5% CO_2_ for 2h, and the absorbance was measured at a wavelength of 450nm. Three independent experiments were performed.

### Colony formation assays

1000 cells/well were plated in 6-well plates, and pools of cells were used to assess growth and clonogenic ability. Ten days after plating the cells, clonogenic progenitors were determined and cells in each group were replated for 6 times. Colonies were rinsed twice with ice-cold PBS, fixed with 4% paraformaldehyde for 20 min on ice and washed twice with PBS. After fixed with methanol, cells were stained with 0.1% crystal violet solution 30 min and then the colonies were imaged and counted. The clone formation rate was calculated as follows: number of cell clone formation/number of inoculated cells ×100%. Three independent experiments were performed.

### Statistical analysis

The data were presented as mean ± standard deviation. Statistical analysis between two groups of normally distributed data was performed using the Student’s t-test. Non-normally distributed data were analyzed using the nonparametric Mann-Whitney U test. For comparisons among multiple groups, homogeneity of variance was first verified using Levene’s test, followed by standard one-way analysis of variance (ANOVA). The *P* <0 .05 was considered significant. Statistical analysis was performed on SPSS 24.0.

## Results

### Systematic profiling of acetylation and lactylation in CRC cells induced by *Roseburia intestinalis*

**Figure 1:**
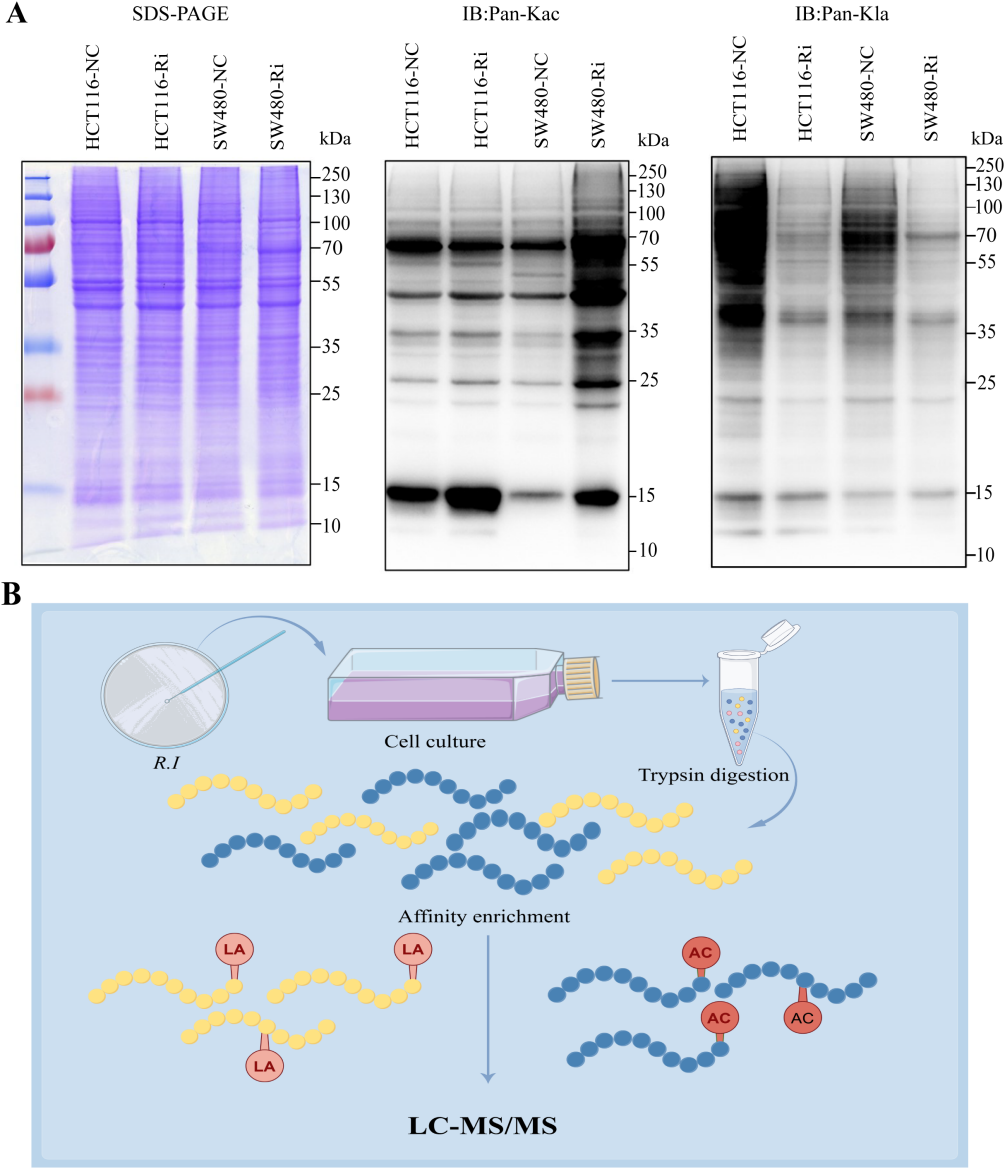
Global landscape of lysine acetylation (Kac) and lysine lactylation (Kla) in CRC cells stimulated by *Roseburia intestinalis*. **(A)** Immunoblotting was performed on whole cell lysates to determine the expression of Kac and Kla. Protein loading was normalized, and samples were run in triplicate as technical replicates. **(B)** To characterize global Kac and Kla profiles in ImKCs, tryptic digests were prepared and Kac/Kla-modified peptides were selectively enriched using pan-specific anti-Kac and anti-Kla antibodies immobilized on agarose beads. The resulting enriched peptides were analyzed by LC-MS/MS. HCT116-NC, HCT116 cell control; HCT116-Ri, Ri-treated HCT116 cell; SW480-NC, SW480 cell control; SW480-Ri, Ri-treated SW480cell.

In colorectal cancer (CRC) tumor tissues, there is a marked increase in global lysine lactylation levels, indicating a metabolic reprogramming characteristic of tumor cells (**Supplementary Figures 1A, B**). Upon intervention with *R.i*, we observed a significant increase in acetylation levels alongside a decrease in lactylation levels in CRC cells (**Figure 1A**; **Supplementary Figure 1C**). This shift suggests a potential interplay between these two PTMs that may influence cancer cell behavior and metabolism. To systematically investigate the regulatory patterns of *Ruminococcus intestinalis* on lactylation and acetylation in CRC, we performed a quantitative proteomics study focusing on both lactylation and acetylation. Our experimental workflow, depicted in **Figure 1B**, illustrates the steps taken to investigate these modifications systematically, including protein extraction, trypsin digestion, and enrichment of lactylated peptides using anti-Pan Kla antibodies, followed by identification through liquid chromatography/tandem mass spectrometry (LC-MS/MS). Overall, this study provides insights into the dynamic changes in acetylation and lactylation in CRC and highlights the potential role of *R.i* in metabolic regulation within this cancer context.

In a total of 8123 peptide segments, 5791 modified peptides were identified, leading to the detection of 6134 distinct acetylation modification sites across 3037 acetylated proteins. Among these, 5443 acetylation modification sites were identified in 2710 quantifiable acetylated proteins (**Supplementary Figure 2A**). A total of 23564 peptide segments were analyzed, resulting in the identification of 7594 modified peptides, which revealed 7882 distinct lactylation modification sites across 2386 lactylated proteins. Among these, 7095 lactylation modification sites were identified in 2156 quantifiable lactylated proteins (**Supplementary Figure 2B**).

**Figure 2:**
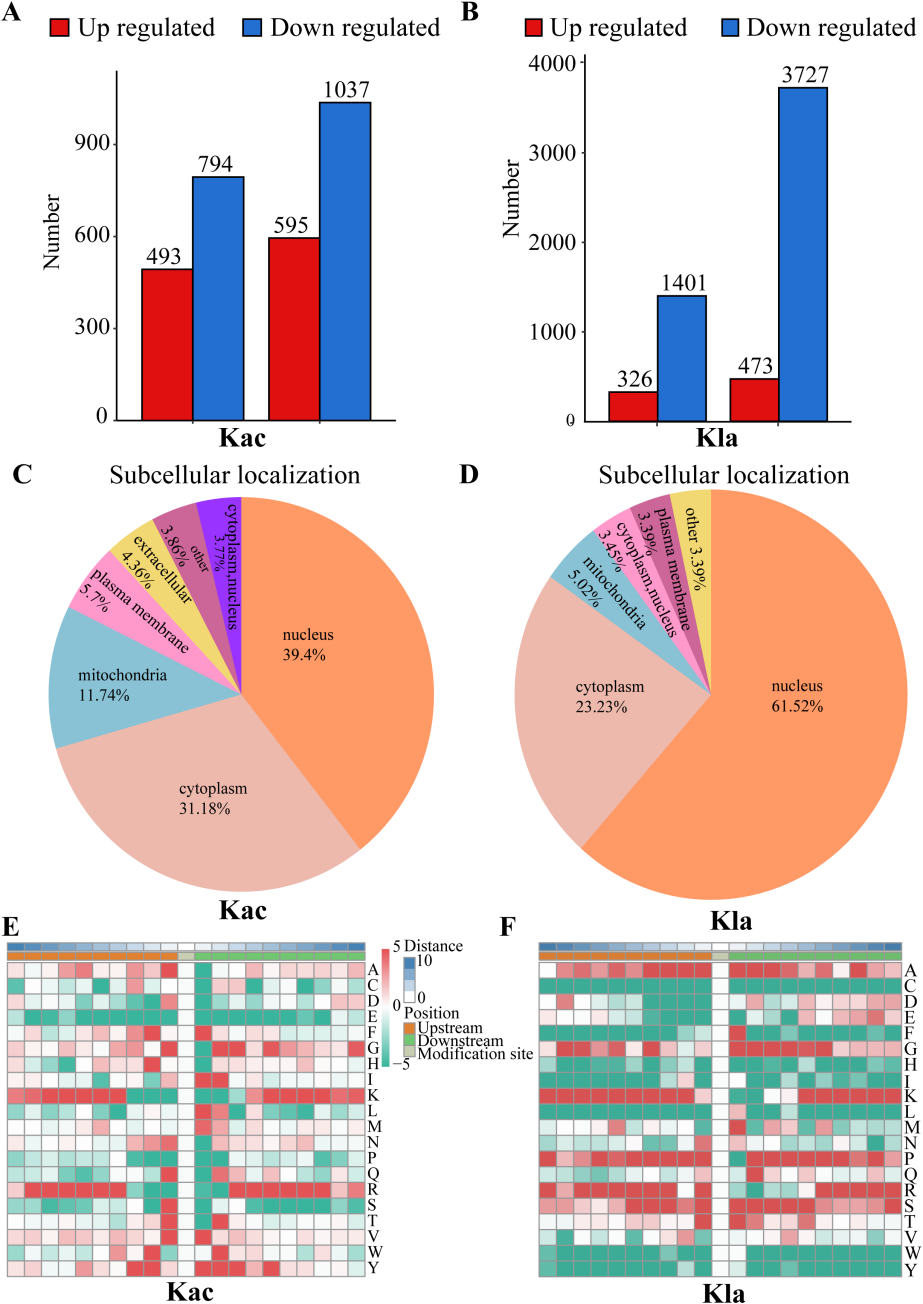
Distribution of Kac and Kla proteins in CRC cells stimulated by *Roseburia intestinalis*. **(A)** Number of peptides, peptides with identified Kac-modified sites and identified proteins detected by mass spectrometry. **(B)** Number of peptides, peptides with identified Kla-modified sites and identified proteins detected by mass spectrometry. **(C)** The subcellular distribution of Kac was characterized. **(D)** The subcellular distribution of Kla was characterized. **(E, F)** The motif characteristics of modified sites were analyzed using the motif-x algorithm.

Acetylation analysis revealed a total of 493 up-regulated proteins and 794 down-regulated proteins. The acetylation sites demonstrated a distribution of 595 up-regulated sites and 1037 down-regulated sites (**Figure 2A**). In addition, lactylation profiling identified 326 up-regulated proteins alongside 1401 down-regulated proteins, with the corresponding lactylation sites showing 473 upregulated and 3727 down-regulated sites (**Figure 2B**). The differentially expressed proteins are presented in **Supplementary Tables 2, 3**.

### Functional categories of protein acetylation in CRC cells induced by *Roseburia intestinalis*

The functional roles of proteins are intricately linked to their subcellular localization. To characterize the distribution of each acetylated (Kac) protein, we conducted a subcellular distribution analysis, as shown in **Figure 2C**. Our results revealed that, in HCT116 cells induced by *R.i*, acetylated proteins were predominantly localized in the nucleus (39.4%), followed by the cytoplasm (31.18%), mitochondria (11.74%), plasma membrane (5.7%), and extracellular (4.36%). To achieve a thorough understanding of the functional characteristics of various proteins, we performed a comprehensive functional annotation of the identified proteins. The **Supplementary Figure 2C** illustrates the number of acetylated proteins and modification sites annotated to COG/KOG functional classification, protein domains, KEGG pathways, Gene Ontology, WikiPathways, Transcription Factor, Reactome and HallMark. Using the MoMo analysis tool based on the motif-x algorithm, we examined the motif characteristics of the acetylation modification sites. The analysis focused on peptide sequences composed of 10 amino acids upstream and downstream of all identified modification sites. **Figure 2E** displays the variation in frequency of amino acid occurrence near the modification sites. According to COG/KOG category analysis, proteins involved in Posttranslational modification, transport, and metabolism are more likely to be acetylated (**Supplementary Figure 3A**). Most differentially expressed proteins are enriched in Posttranslational modification, Translation, Lipid transport and metabolism (**Supplementary Figure 3B**).

### Functional categories of protein lactylation in CRC cells induced by *Roseburia intestinalis*

Lactylated proteins exhibited a different distribution (**Figure 2D**), with the majority found in the nucleus (61.52%), followed by the cytoplasm (23.23%), mitochondria (5.02%), and plasma membrane (3.39%). **Supplementary Figure 2D** illustrates the distribution of lactylated proteins and their corresponding modification sites across various functional categories, including COG/KOG classifications, protein domains, KEGG pathways, Gene Ontology terms, WikiPathways, transcription factors, Reactome annotations, and Hallmark signatures. To further elucidate the motif characteristics of the lactylation modification sites, we employed the MoMo analysis tool based on the motif-x algorithm. This analysis focused on the peptide sequences, consisting of 10 amino acids, flanking all identified modification sites. As depicted in **Figure 2F**, we observed notable variations in the frequency of amino acid occurrences adjacent to the modification sites.

According to the COG/KOG classification analysis, proteins involved in post-translational modifications, signaling mechanisms, transcription, lipid transport, and metabolism are more susceptible to lactylation (**Supplementary Figure 4A**). The majority of differentially expressed proteins were found to be enriched in pathways associated with translation, infectious diseases, and cancer (**Supplementary Figure 4B**). This enrichment suggests that lactylation may play a crucial role in modulating key biological processes and pathways implicated in tumorigenesis and disease progression. The propensity for lactylation among these functionally significant proteins underscores its potential impact on cellular signaling networks and highlights lactylation as an essential post-translational modification warranting further investigation in the context of cancer biology.

### Global profiling of quantitative proteome in CRC cells induced by *Roseburia intestinalis*

The quantitative proteomic analysis of CRC cells treated with *R.i* was conducted using the 4D-Fast DIA. An overview of protein identification is presented in **Supplementary Figure 5A**, where a total of 60,089 identified peptide segments were recorded, with 57,492 unique peptide sequences; a total of 7,987 proteins were identified, and 7,857 proteins were suitable for quantitative comparison. To gain an in-depth understanding of the functional characteristics of the identified proteins, we performed comprehensive functional annotations on them. Among these, 6,591 proteins were classified into COG/KOG functional categories, 4,803 proteins were associated with protein domains, 3,850 proteins were enriched in KEGG pathways, 7,635 proteins were enriched in Gene Ontology (GO) terms, 3,923 proteins were enriched in WikiPathways, 795 proteins were identified as transcription factors (TFs), 5,293 proteins were enriched in Reactome pathways, and 2,497 proteins were associated with Hallmark pathways (**Supplementary Figure 5B**).

Compared to the control group, there were 1,872 upregulated proteins and 2,280 downregulated proteins (**Supplementary Figure 5C**). The functional classification of the differentially expressed proteins is presented in **Supplementary Figure 5D**. Based on the findings presented in **Supplementary Figure 5E**, the differentially expressed proteins were significantly enriched in several key metabolic pathways, including glutathione metabolism, N-glycan biosynthesis, sphingolipid metabolism, the interconversion of pentose and glucuronate, and mucin-type O-glycan biosynthesis. These pathways are critical for various cellular functions, and their alteration may have significant implications for cellular behavior and the overall metabolic landscape within the context of the treatment being investigated. Further exploration of these enriched pathways could provide valuable insights into the molecular mechanisms underlying the observed changes in protein expression.

### Proteomics of acetylation modification in CRC cells induced by *Roseburia intestinalis*

First, a comparison of the protein groups and the acetylation modified groups was conducted at the quantitative level, and the expression abundance of the proteins was statistically analyzed. Subsequently, differentially expressed proteins and differential modification sites were identified based on a specified threshold, followed by significance distribution analysis.

**Figure 3:**
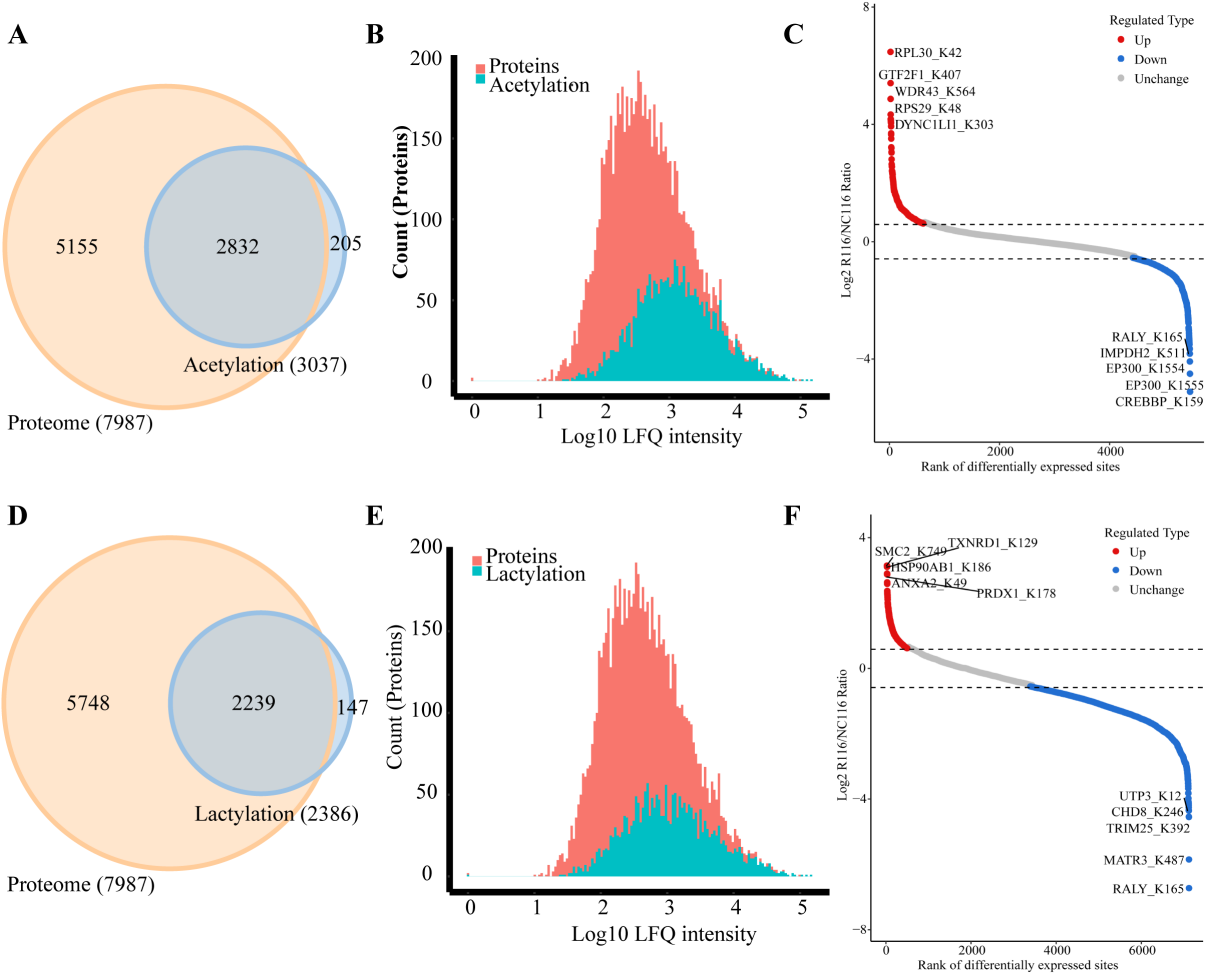
Integrated analysis of acetylation, lactylation and proteomics in CRC cells induced by *Roseburia intestinalis*. **(A)** The Venn diagram was used to illustrate the overlap between the proteins identified in the proteome and acetylome analyses. **(B)** The distribution of protein intensity values for both the total proteome and the acetylated proteome was visualized using histograms. **(C)** Differentially expressed proteins and modified sites from the acetylome were visualized using scatter plots. **(D)** The overlap of proteins identified through proteomic and lactylomic analyses was visualized using a Venn diagram. **(E)** The distribution of protein intensity values in the total proteome and the lactylated proteome was visualized using histograms. **(F)** Differentially expressed lactylated proteins and modified sites were visualized using scatter plots.

**Figure 4:**
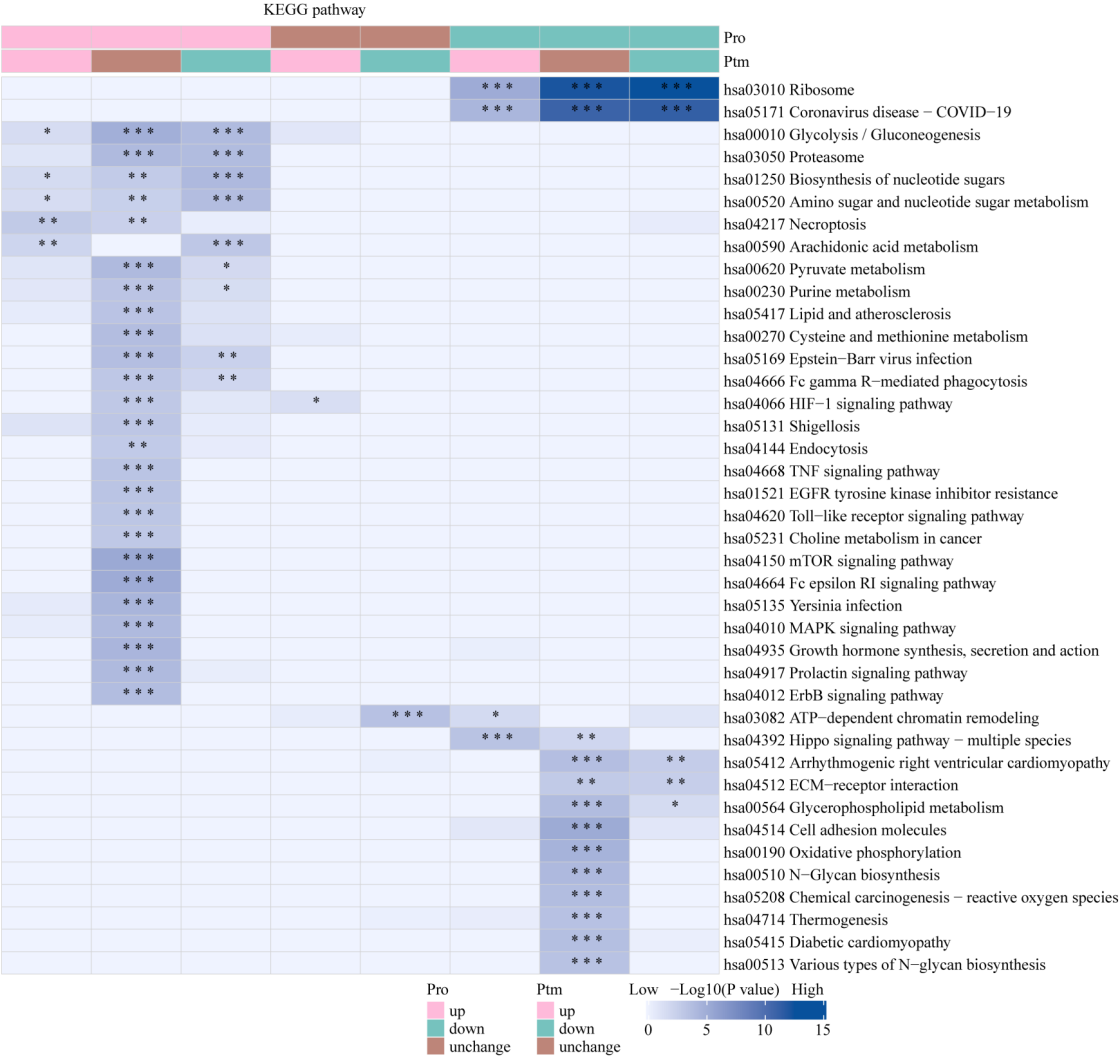
KEGG pathway functional enrichment analysis of acetylated proteins. * p < 0.05, ** p < 0.01, *** p <0.001.

The Venn diagram was used to illustrate the intersection of identified proteins between the protein group and the modified group, with a total of 2,832 proteins (**Figure 3A**). The distribution of intensity values for the proteins identified in the protein group and those that underwent modifications was illustrated using a histogram (**Figure 3B**). Based on the criteria for differential selection from proteomics and modificationomics, differentially expressed proteins and differential modification sites were identified separately. The significantly upregulated modification sites in the experimental group are RPL30_K42, GTF2F1_K407, WDR43_K564, RPS29_K48 and DYNC1LI1_K303, while the significantly downregulated proteins are EP300_K1554, CREBBP_K1597, EP300_K1555, IMPDH2_K511 and RALY_K165 (**Figure 3C**). The acetylome profiling identified significant enrichment of differentially expressed proteins in several critical metabolic pathways, including: Glycolysis, Lipid metabolism, Pyruvate metabolism, Glycerophospholipid metabolism. The pathways enriched with modified proteins are presented in **Figure 4**.

### Proteomics of lactylation modification in CRC cells induced by *Roseburia intestinalis*

**Figure 5:**
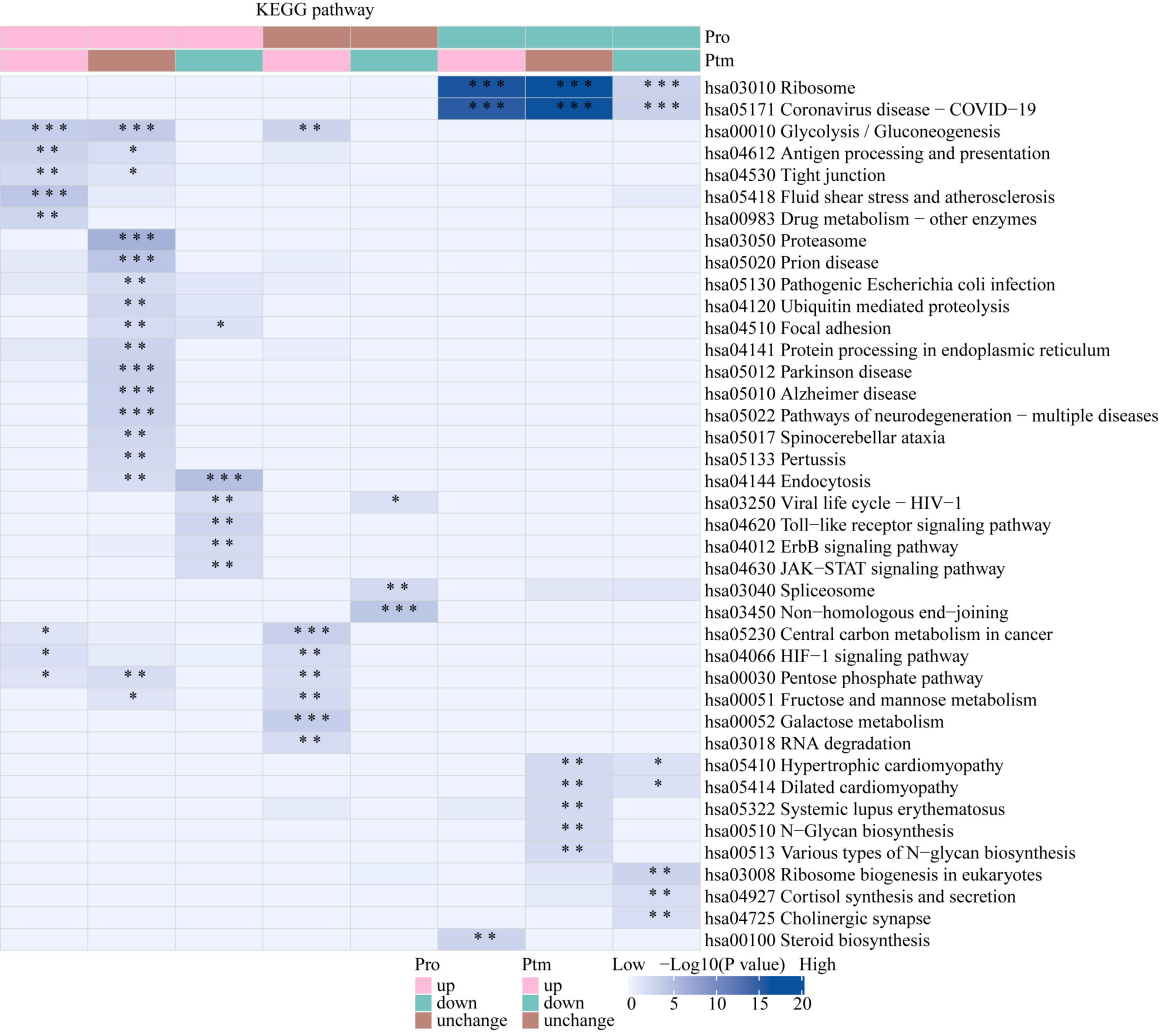
KEGG pathway functional enrichment analysis of lactylated proteins. * p < 0.05, ** p < 0.01, *** p <0.001.

The Venn diagram was used to illustrate the intersection of identified proteins between the protein group and the lactylation modified group, with a total of 2,239 proteins (**Figure 3D**). The distribution of intensity values for the proteins identified in the protein group and those that underwent modifications was illustrated using a histogram (**Figure 3E**). The significantly upregulated proteins and their modified sites are SMC2_K749, HSP90AB1_K186, TXNRD1_K129, PRDX1_K178, and ANXA2_K49, while the significantly downregulated modified proteins and their sites are RALY_K165, MATR3_K487, TRIM25_K392, CHD8_K246, and UTP3_K12 (**Figure 3F**). Lactylome analysis demonstrated distinct protein enrichment in: Glycolysis, Galactose metabolism, Pentose phosphate pathway, Non-homologous end-joining. The pathways enriched with modified proteins are illustrated in **Figure 5**. Notably, glycolysis emerged as the principal convergent pathway between acetylation and lactylation modifications, suggesting its central regulatory role in metabolic reprogramming under these PTMs.

### Characterization of Kac and Kla patterns in CRC cells induced by *Roseburia intestinalis*

Functional enrichment analysis of proteins with concurrent lactylation and acetylation modifications clustered into pathways such as ATP-dependent chromatin remodeling, RNA degradation, glycolysis, and the cell cycle (**Supplementary Figure 6A**). Similarly, functional enrichment analysis of the modification sites exhibiting both lactylation and acetylation revealed clustering into pathways including the spliceosome, ATP-dependent chromatin remodeling, glycolysis, lysine degradation, and central carbon metabolism (**Supplementary Figure 6B**). This analysis highlights the significant biological pathways associated with proteins undergoing simultaneous lactylation and acetylation, indicating their potential roles in critical cellular processes such as chromatin remodeling, energy metabolism, and RNA processing. The overlapping pathways suggest a complex interplay between these PTMs in regulating fundamental cellular functions.

### Co-expression network between differentially expressed proteins and lactylation modification sites

**Figure 6:**
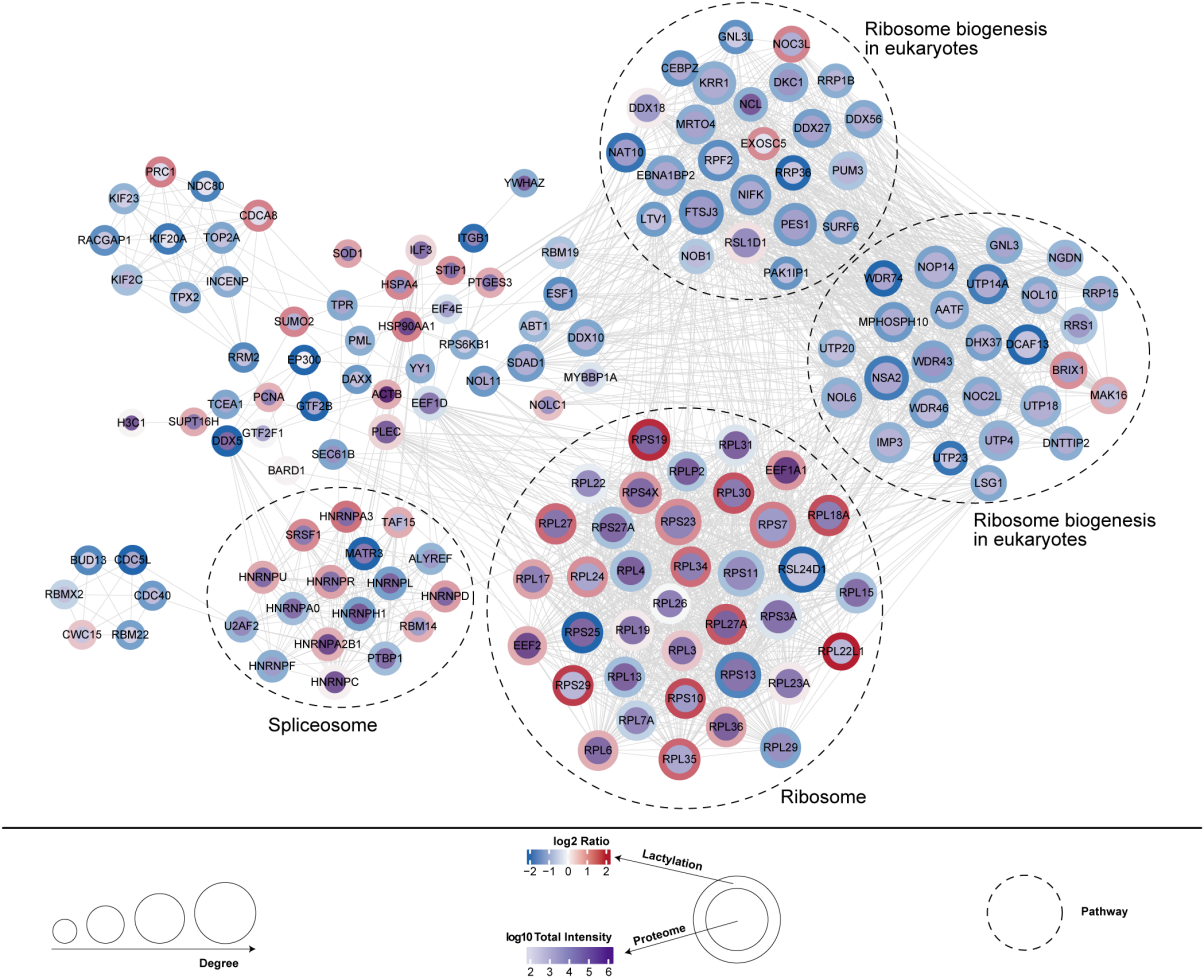
Co-expression network between differentially expressed proteins and lactylation modification sites.

This study further analyzed the co-expression network between differentially expressed proteins (DEPs) and lactylation modification sites, revealing potential functional interactions. Functional enrichment analysis demonstrated significant associations with ribosome biogenesis (20 upregulated and 14 downregulated proteins) and spliceosome (8 upregulated and 8 downregulated proteins) (**Figure 6**).

### The effect of *Roseburia intestinalis* on the proliferative capacity of CRC cells

The proliferation of cells is a major feature of tumors. Using CCK-8 and colony formation assays, we evaluated the effects of *R.i* and *R.i*-conditioned medium (*R.i*-CM) on CRC cell proliferation. CCK8 kit was used to determine cell viability. The results demonstrated that *R.i*-CM significantly inhibited CRC cell viability compared to the control group, whereas live *R.i* showed no significant inhibitory effect (**Supplementary Figure 7A**). The colony formation assay was performed to confirm the clonogenic potential. The results showed that *R.i*-CM triggered a significant decrease of colony numbers in CRC cells (**Supplementary Figures 7B, C**).

## Discussion

Dysbiosis of the gut microbiota and aberrant chromatin modifications play significant roles in the progression of CRC. As a unique functional organ in the human body, the intestines harbor approximately 3×10^13^ bacterial cells (33–35). The gut microbiota maintains a dynamic balance, playing critical roles in food digestion and absorption, enhancing intestinal defense mechanisms, and promoting the development of the immune system (36). However, this balance can be disrupted by host and environmental factors, leading to dysbiosis that may be associated with various diseases, including colorectal cancer. Numerous studies employing 16S rRNA sequencing and metagenomics have revealed that, compared to healthy individuals, CRC patients exhibit higher abundances of pathogenic bacteria such as Escherichia/Shigella, Fusobacterium nucleatum, Enterococcus faecalis, and enterotoxigenic Bacteroides fragilis (37–40). Conversely, beneficial bacteria, including Roseburia and Bifidobacterium, are found to be significantly reduced in the CRC cohort (41). CRC appears to be a “bacterial-associated disease,” suggesting that the gut microbiota provides a novel perspective for studying the progression of CRC. Further exploration of the mechanisms by which key intestinal bacteria influence CRC is currently a frontier of scientific research.

Recent studies have revealed that lactate, which accumulates in the tumor microenvironment, is not merely a “metabolic waste product” but also functions as a multifaceted bio-signaling molecule that regulates tumor proliferation, metastasis, inflammation, angiogenesis, immunity, chemoresistance, and gene expression (42). In 2019, Professor Zhao Yingming first reported lactate-derived histone lysine lactylation as a novel post-translational modification of histones that plays a role in gene transcription regulation (43). Lactylation refers to the modification of lysine residues on histones by accumulated lactate, which alters the conformation, stability, and function of proteins, thereby regulating biological functions. Recent research has demonstrated that lactate drives macrophage remodeling by inhibiting RARγ expression, enhancing interleukin-6 levels in the tumor microenvironment, and activating the STAT3 signaling pathway to promote CRC progression (44). Cai et al. reported that lactate acts as a signaling molecule and serves as a specific ligand for G protein-coupled receptor 81 (GPR81), exerting various biological effects through GPR81 binding (45). Additionally, lactate can induce lipid metabolic reprogramming, leading to the formation and mobilization of lipid droplets (46). Chen et al. identified 444 lactylated proteins (including histones and non-histones) with 637 lactylation sites in the colorectal cancer cell line SW480 through proteomic analysis (47).

Numerous studies have demonstrated elevated lactylation levels in CRC tumor tissues, a finding that has been further validated in our center’s samples. Moreover, the present study revealed that *R.i* intervention could reduce global lactylation levels while increasing acetylation levels in CRC cells. Further investigations revealed distinct subcellular localization patterns between lactylated and acetylated proteins, with 61.52% of lactylated proteins localized in the nucleus compared to only 39.4% of acetylated proteins. This analysis highlights the distinct subcellular localization patterns of Kac and Kla proteins in CRC cell, which may influence their biological functions. These results underscore the extensive alterations in both acetylation and lactylation modifications in the context of colorectal cancer, indicating a complex regulatory network that may influence tumor progression and response to treatment.

The functional properties of proteins demonstrate complex relationships with their intracellular localization, followed by functional annotation of the identified modified proteins. Proteins participating in post-translational modifications, transport, and metabolic processes demonstrate higher susceptibility to acetylation. In contrast, differentially expressed lactylated proteins were predominantly enriched in translation-related and cancer-associated pathways. This distinct enrichment pattern suggests that lactylation may serve as a critical regulatory mechanism governing fundamental biological processes involved in tumorigenesis and disease progression. Functional enrichment analysis of dually lactylated and acetylated proteins revealed significant enrichment in ATP-dependent chromatin remodeling, RNA degradation, glycolysis, and cell cycle regulation pathways. The observed modification bias among these functionally diverse protein groups highlights their potential to reshape cellular signaling networks, underscoring the necessity for deeper investigation into acylation as a biologically significant post-translational modification in cancer biology.

Histone acetylation is a common form of chromatin modification and is closely associated with the gut microbiota (48). This process primarily occurs on lysine residues, where the amino group present on the lysine side chain carries a positive charge under physiological conditions, allowing it to bind tightly with negatively charged DNA phosphate groups (49, 50). Following acetylation, the positive charge is neutralized, resulting in a reduced affinity for DNA that leads to a more relaxed chromatin structure and enhances gene expression (51, 52). The dynamic balance of histone acetylation is regulated by histone HDACs and HATs. Given that acetylation is a reversible modification, intervening in the functions of HATs, HDACs, and acetyl-lysine readers can influence the expression levels and activity of downstream target genes, thereby exerting biological effects (53, 54). Research has shown that HDAC inhibitors significantly suppress intracellular lipid accumulation and alleviate hepatic steatosis, inflammation, and liver injury in mouse models of non-alcoholic steatohepatitis (55). In this study, we established a model of CRC cell lines treated with *R. intestinalis*. The results indicated that *R. intestinalis* significantly upregulates the overall acetylation levels in CRC cells.

Preliminary mechanistic investigations revealed that *R.i.*cm exhibits more potent suppression of CRC cell viability, suggesting the bioactive effects of *R.i* may be mediated through microbial metabolites. Existing studies have demonstrated that *R.i* may modulate post-translational modification (PTM) pathways through its metabolic derivatives, particularly butyrate - a short-chain fatty acid (SCFA) produced by gut microbiota that plays a pivotal role in epigenetic regulation and PTMs of proteins. As a competitive inhibitor of class I/IIa histone deacetylases (HDACs), butyrate elevates histone acetylation by blocking deacetylation, leading to chromatin relaxation and transcriptional activation of tumor suppressor and anti-inflammatory genes, thereby modulating cell proliferation, differentiation, and apoptosis (56). It may also regulate acetylation of transcription factors (e.g., NF-κB, STAT3) through HDAC inhibition (57). In colon cancer cells, butyrate induces hyperacetylation of H3/H4 histones, causing cell cycle arrest (58). Additionally, butyrate may influence lactylation by: metabolic reprogramming to reduce lactate availability, competitive inhibition of lactate transferases (e.g., p300/CBP) due to structural similarity to lactyl-CoA, and altering the acetylation-lactylation balance via HDAC inhibition. Butyrate also modulates methylation by acetyl-CoA-dependent crosstalk and enhances USP5-mediated GPX4 ubiquitination, synergizing with anti-PD-1 therapy (59). As a key energy source for colonocytes, its β-oxidation generates acetyl-CoA (a direct substrate for histone acetylation), linking metabolic rewiring to epigenetic regulation (60–62). Collectively, butyrate orchestrates PTMs through HDAC inhibition, metabolic interference, and cross-talk between modifications, highlighting its multifaceted roles in cellular physiology.

## Conclusions

In summary, we analyzed the global acylome characteristics of Kac and Kla in CRC cells after *R.i* intervention and discovered that these two PTMs exhibit distinct features. This indicates that these modifications may play differential roles in cellular regulation and function. This distinction enhances our understanding of the specific biological implications of these PTMs in the context of colorectal cancer. These findings enhance our understanding of the specific contexts within which acylation modifications occur, providing insights into the potential regulatory mechanisms underlying protein function in the context of cellular signaling and metabolism.

## Data Availability

The proteomics data generated by mass spectrometry have been deposited in the ProteomeXchange Consortium (http://proteomecentral.proteomexchange.org) through the iProX partner repository with the accession number PXD066351.
