# Supplementary material for: From gut to proteomics: the impact of Roseburia intestinalis on post-translational modifications in colorectal cancer
Source: Front Oncol. 2025 Aug 18;15:1599183. doi: 10.3389/fonc.2025.1599183 (PMC12399380; doi:10.3389/fonc.2025.1599183)
Supplement: Supplementary file 1 [file DataSheet1.docx]

Supplementary Material

# Supplementary Figures


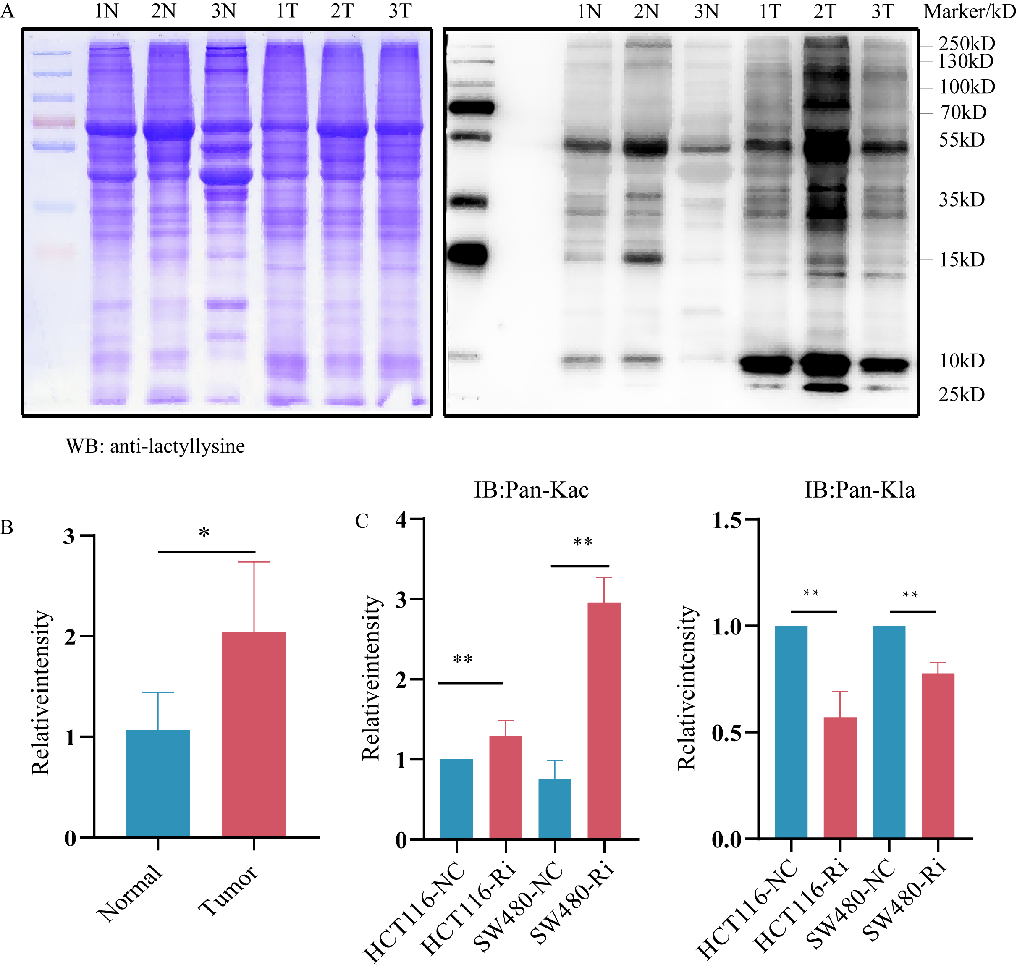


**Supplementary Figure 1.**  **Lactylome landscape in colorectal cancer tissue.**

1. Coomassie brilliant blue staining and Western blotting with pan anti-lactyllysine antibody. (B) Quantification of relative band intensity in tissue samples. (C) Quantification of relative band intensity in cell samples.

N:Normal intestinal tissue from colorectal cancer patients; T: Intestinal tumor tissue from colorectal cancer patients. HCT116-NC: HCT116 cell control, HCT116-Ri: Ri-treated HCT116 cell, SW480-NC: SW480 cell control, SW480-Ri: Ri-treated SW480cell.


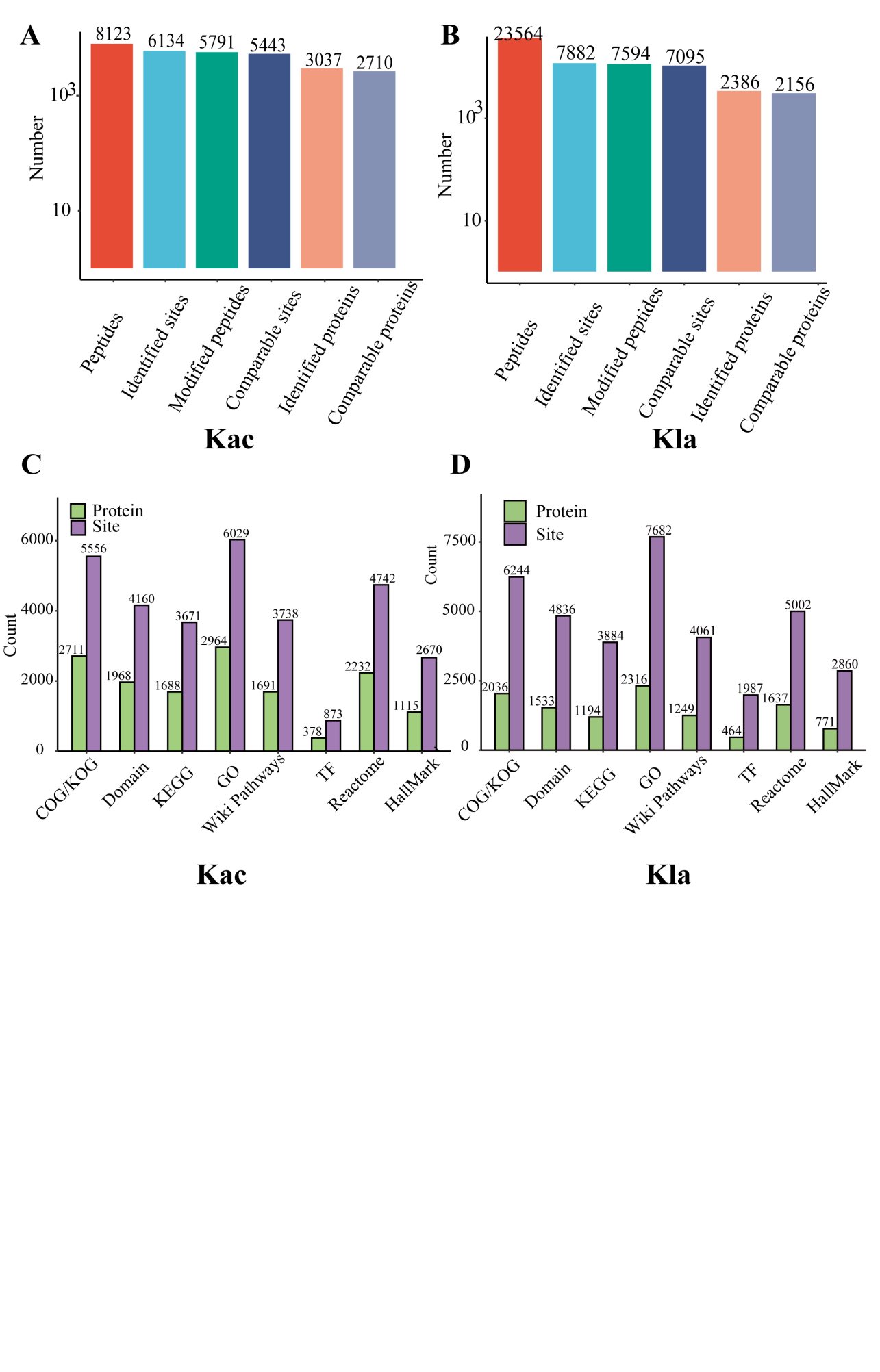


**Supplementary Figure 2. Identification of modification sites and functional annotation of proteins**

1. Identification of acetylation sites; (B) Identification of lactylation sites; (C) Functional annotation of acetylated proteins; (D) Functional annotation of lactylated proteins.


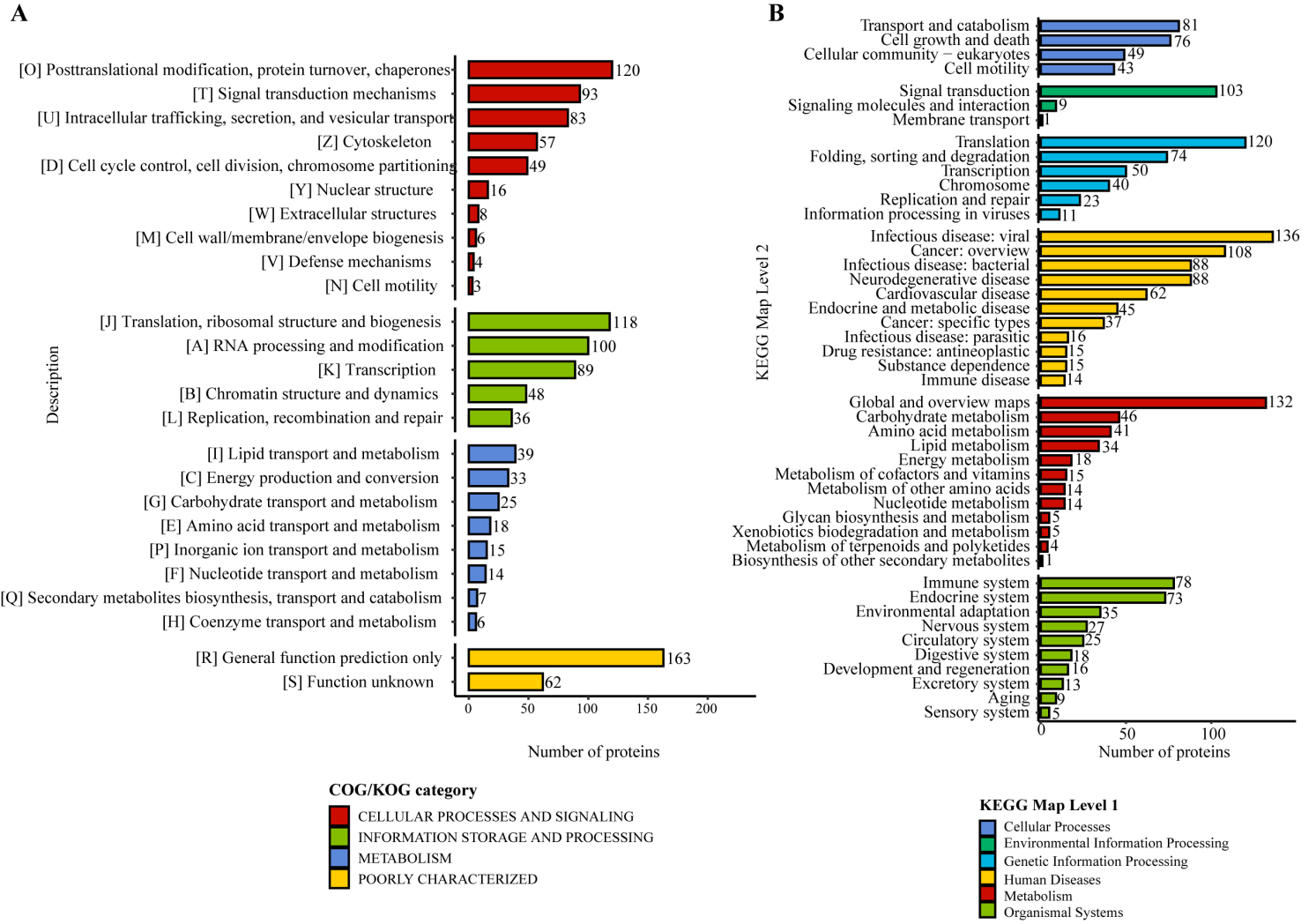


**Supplementary Figure 3. Functional Categories of Protein Acetylation in CRC cells induced by *Roseburia intestinalis***

1. Enrichment analysis of COG/KOG category was performed on the identified Kac-modified proteins. (B) Enrichment analysis of identified Kac proteins based on KEGG pathway.

***
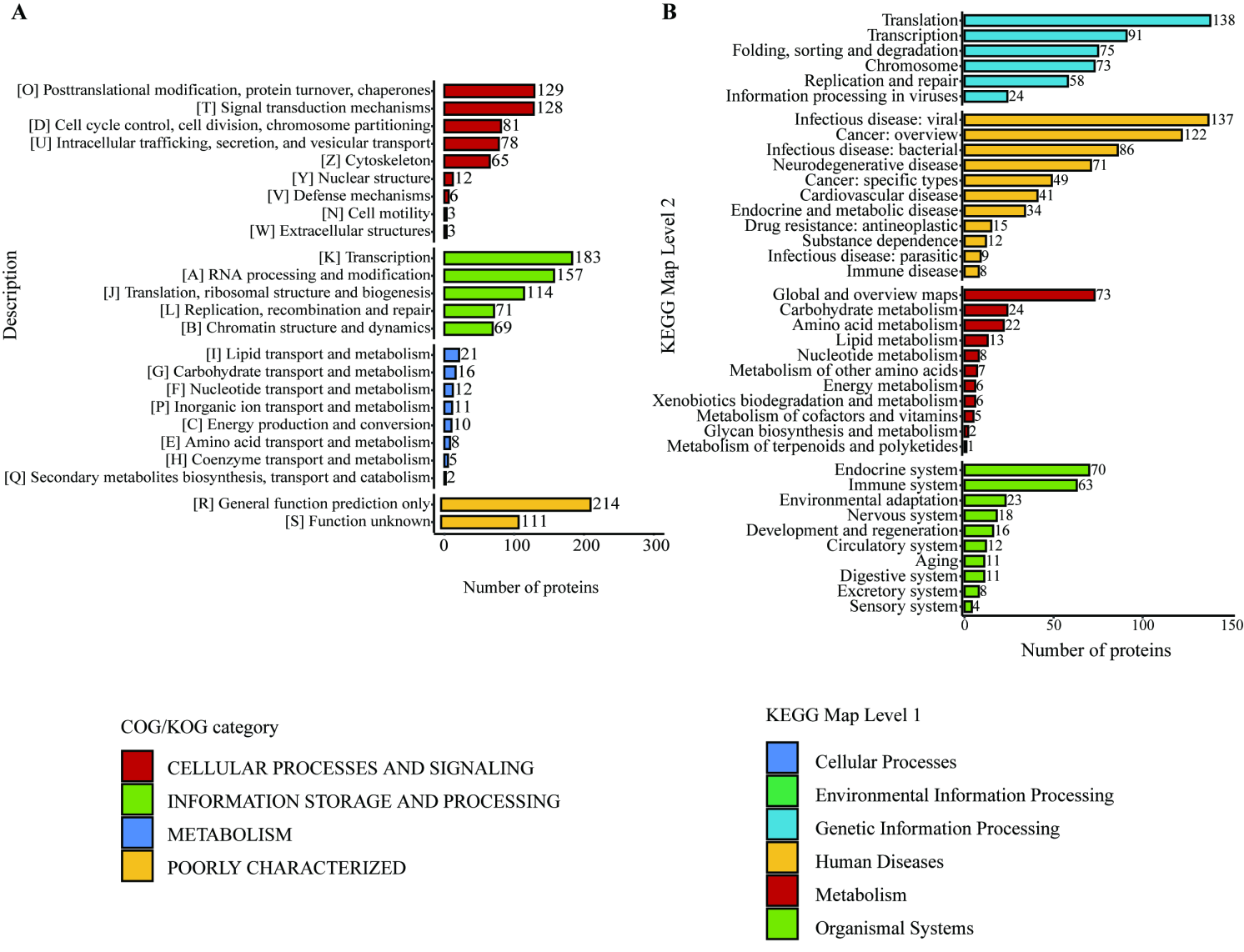
***

**Supplementary Figure 4. Functional Categories of Protein Lactylation in CRC cells induced by *Roseburia intestinalis.***

1. Enrichment analysis of COG/KOG category was performed on the identified Kla-modified proteins. (B) Enrichment analysis of identified Kla proteins based on KEGG pathway.

***
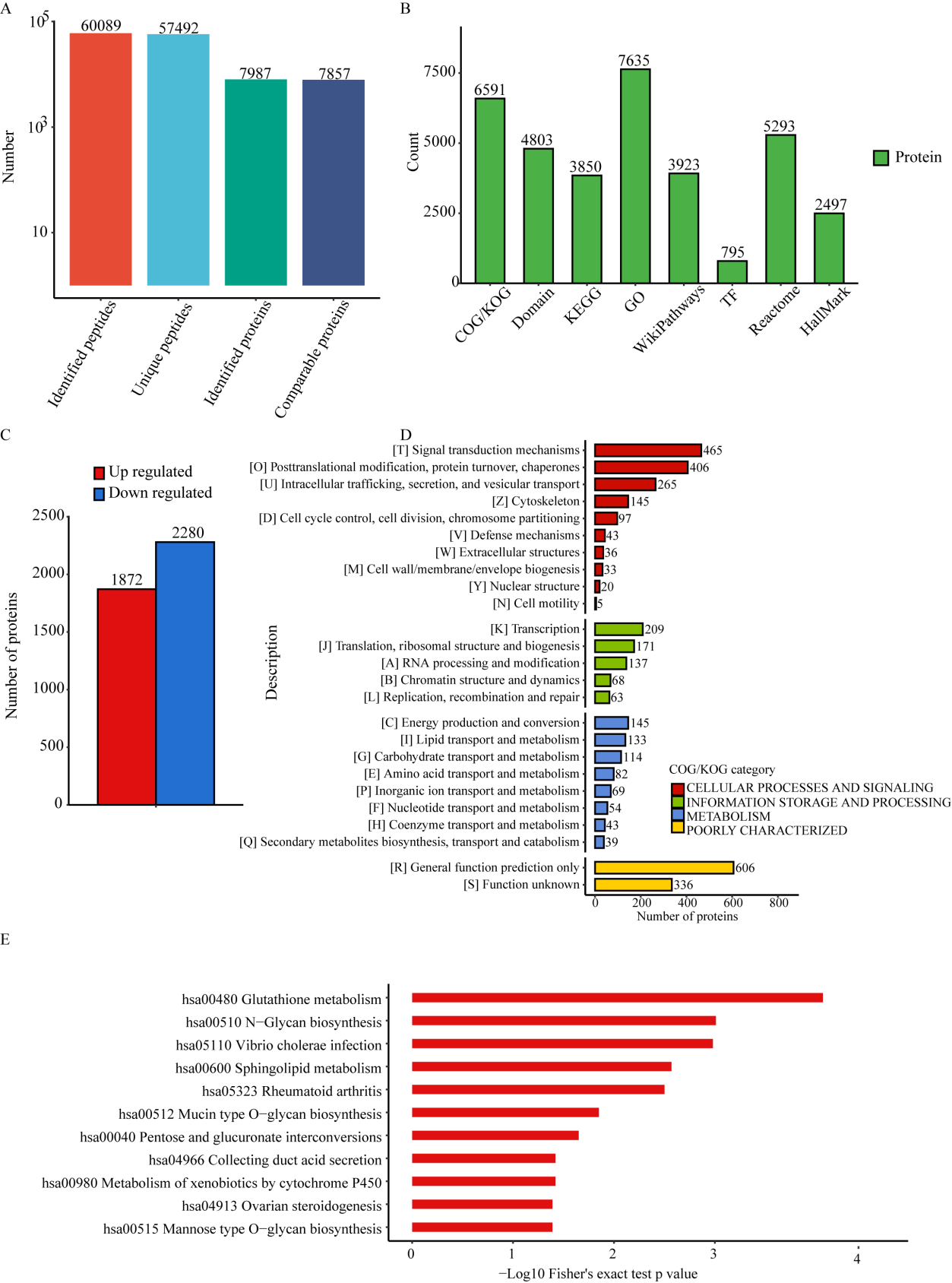
***

**Supplementary Figure 5. Global Profiling of quantitative proteome in CRC cells induced by *Roseburia intestinalis***

1. Overview of Protein Identification; (B) Annotation of Protein Functions; (C) Differential Protein Screening; (D-E) Classification of Differential Proteins Functionality.


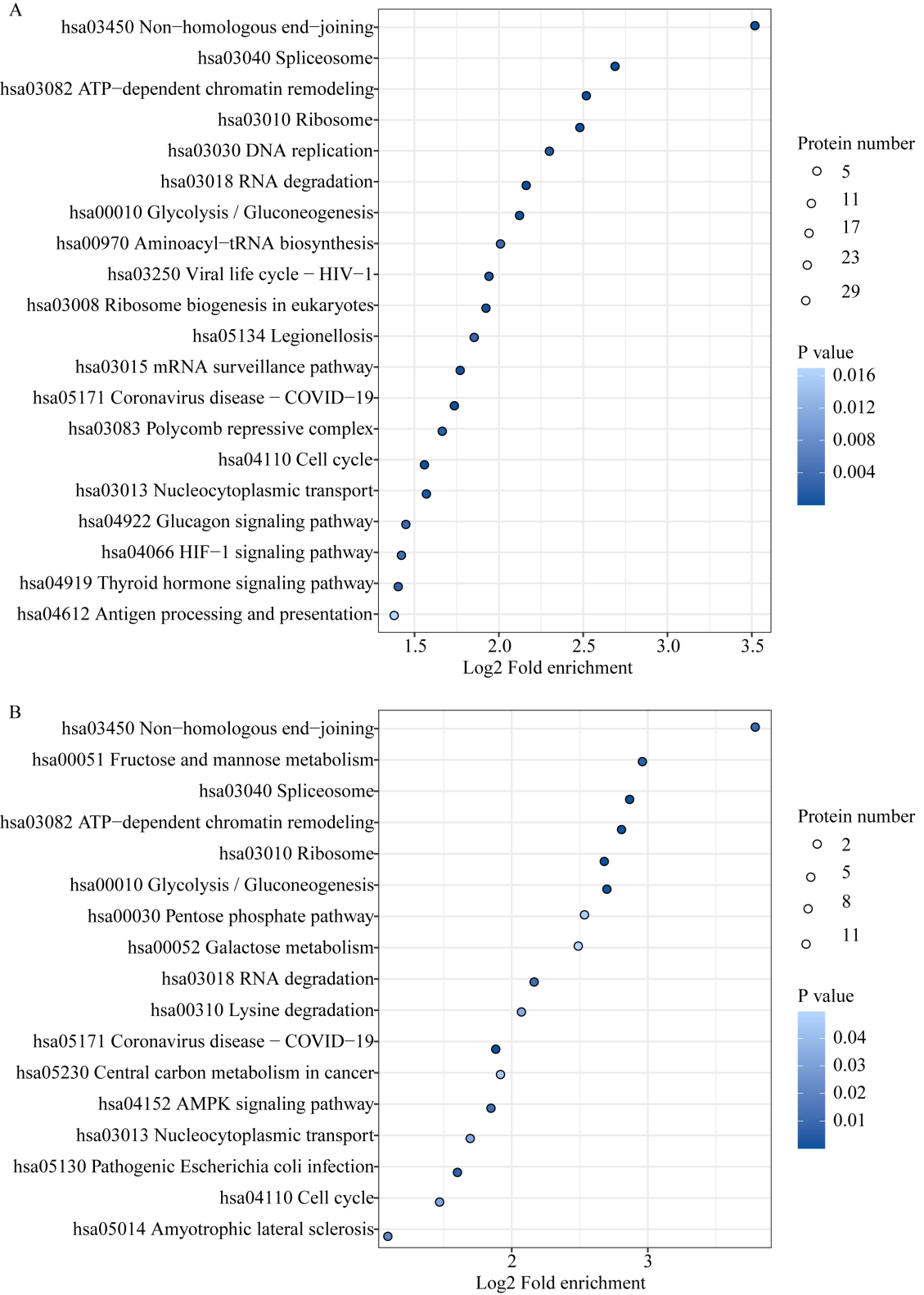


**Supplementary Figure 6. Functional enrichment analysis of proteins with both lactylation and acetylation modifications.**

1. Functional enrichment analysis of the overlapping proteins modified by both acetylation and lactylation. (B)Functional enichment analysis of proteins corresponding to shared modified sites.


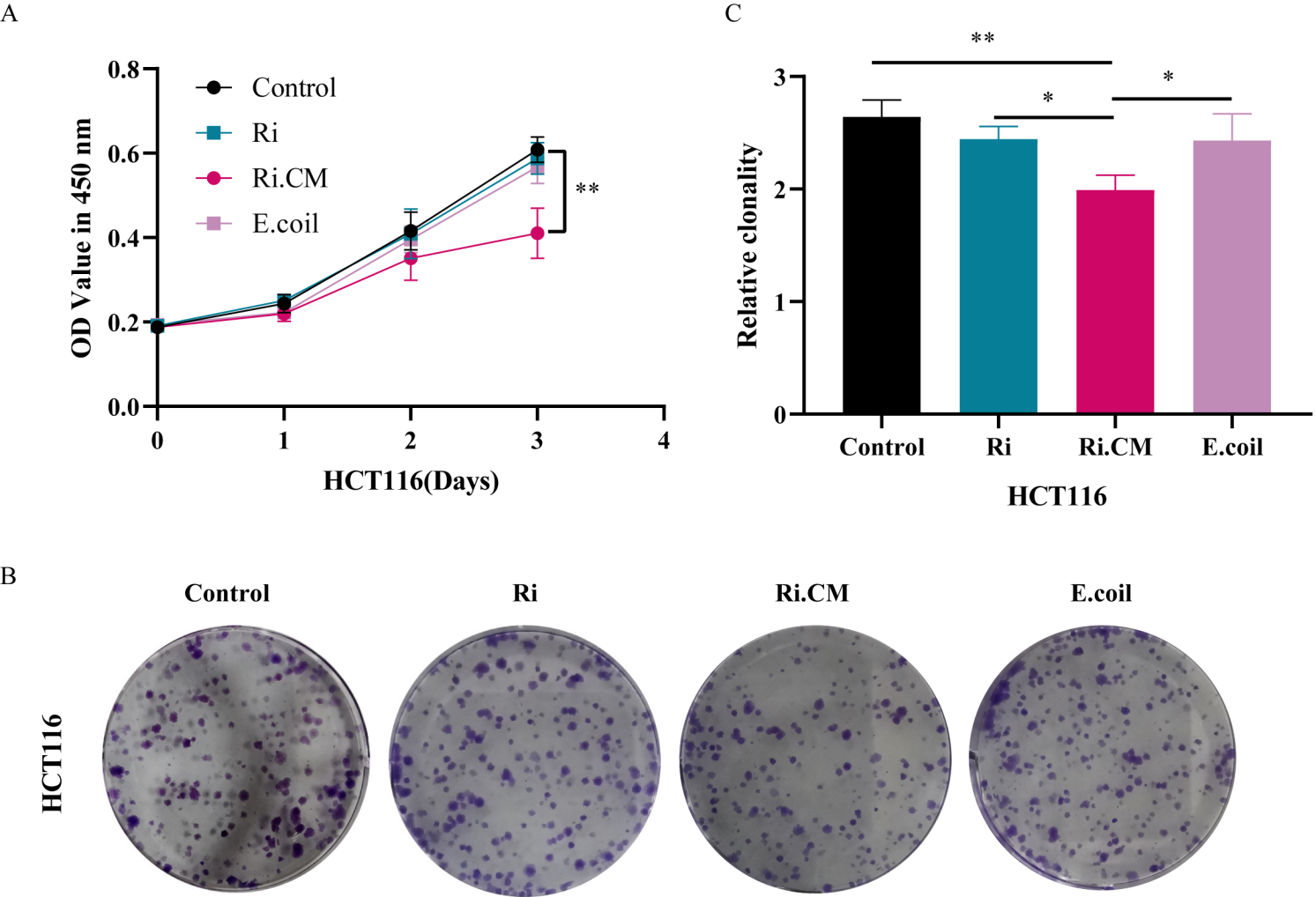


**Supplementary Figure 7. The effect of Roseburia intestinalis on the proliferative capacity of CRC cells**

1. CCK-8 assay to evaluate the effect of Ri on CRC cell viability. (B) Colony formation assay to evaluate Ri's effect on clonogenic ability of CRC cells. (C) Quantitative assessment of colony-forming capacity in different cohorts. blank control (untreated cells), live *Ri* bacteria, *Ri*-conditioned medium, and *E. coli* control; n=6.

**Supplementary Table 1. Detailed clinical characteristics**

| Clinical characteristics | Patient 1 | Patient 2 | Patient 3 |
| --- | --- | --- | --- |
| Age(years) | 67 | 75 | 56 |
| Gender | Female | Female | Male |
| Tumor volume (cm) | 3*3*1 | 3.7*3.7*2.2 | 4*3*1 |
| Tumor location | Sigmoid Colon | Rectum | Rectum |
| Histological Typing | Tubular Adenocarcinoma | Tubular Adenocarcinoma | Tubular Adenocarcinoma |
| Grade | G2 | G1-G2 | G2-G3 |
| TNM stage | T3N1bMx | T3N0Mx | T3N2Mx |
| Serum CEA level (ng/ml) | 6.23 | 3.38 | 2.46 |
| Smoking history | N | N | N |
| Drinking history | N | N | N |

**Supplementary Table 2. Differentially Expressed Acetylated Proteins.**

| **Protein accession** | **Position** | **Amino acid** | **HCT116 R.i/HCT116NC Ratio** | **Regulated Type** | **Protein description** | **Gene name** | **Modified sequence** |
| --- | --- | --- | --- | --- | --- | --- | --- |
| A0A8I5KQE6 | 52 | K | 0.562878648 | Down | Small ribosomal subunit protein uS2B OS=Homo sapiens OX=9606 GN=RPSA2 PE=3 SV=1 | RPSA2 | _SDGIYIINLK[Acetyl (K)]R_ |
| A1L0T0 | 303 | K | 0.657364107 | Down | 2-hydroxyacyl-CoA lyase 2 OS=Homo sapiens OX=9606 GN=ILVBL PE=1 SV=2 | ILVBL | _RPLMVLGSQALLTPTSADK[Acetyl (K)]LR_ |
| A1L0T0 | 369 | K | 0.610560169 | Down | 2-hydroxyacyl-CoA lyase 2 OS=Homo sapiens OX=9606 GN=ILVBL PE=1 SV=2 | ILVBL | _VLSHSSK[Acetyl (K)]IIIVNR_ |
| A1X283 | 609 | K | 0.226413689 | Down | SH3 and PX domain-containing protein 2B OS=Homo sapiens OX=9606 GN=SH3PXD2B PE=1 SV=3 | SH3PXD2B | _K[Acetyl (K)]PNLRPISK_ |
| A2RRP1 | 1261 | K | 1.599658978 | Up | NBAS subunit of NRZ tethering complex OS=Homo sapiens OX=9606 GN=NBAS PE=1 SV=2 | NBAS | _QSTK[Acetyl (K)]LLGLAELLR_ |
| A6NGB9 | 372 | K | 2.101615348 | Up | WAS/WASL-interacting protein family member 3 OS=Homo sapiens OX=9606 GN=WIPF3 PE=1 SV=4 | WIPF3 | _PGAGGGK[Acetyl (K)]LNPPPAPPAR_ |
| A6NHR9 | 771 | K | 2.009009639 | Up | Structural maintenance of chromosomes flexible hinge domain-containing protein 1 OS=Homo sapiens OX=9606 GN=SMCHD1 PE=1 SV=2 | SMCHD1 | _EIISHISQHGGK[Acetyl (K)]WPYWFK_ |
| A6NHR9 | 777 | K | 1.684593378 | Up | Structural maintenance of chromosomes flexible hinge domain-containing protein 1 OS=Homo sapiens OX=9606 GN=SMCHD1 PE=1 SV=2 | SMCHD1 | _WPYWFK[Acetyl (K)]K_ |
| A6NHR9 | 1309 | K | 2.566735258 | Up | Structural maintenance of chromosomes flexible hinge domain-containing protein 1 OS=Homo sapiens OX=9606 GN=SMCHD1 PE=1 SV=2 | SMCHD1 | _ASNLK[Acetyl (K)]LMPSNQQHK_ |
| A6NHR9 | 1717 | K | 0.397503535 | Down | Structural maintenance of chromosomes flexible hinge domain-containing protein 1 OS=Homo sapiens OX=9606 GN=SMCHD1 PE=1 SV=2 | SMCHD1 | _SC[Carbamidomethyl (C)]TLPNYTK[Acetyl (K)]GSGDVLGK_ |
| A6NHR9 | 1852 | K | 0.6084244 | Down | Structural maintenance of chromosomes flexible hinge domain-containing protein 1 OS=Homo sapiens OX=9606 GN=SMCHD1 PE=1 SV=2 | SMCHD1 | _EVVK[Acetyl (K)]ITHC[Carbamidomethyl (C)]PTLLTR_ |
| A6NHR9 | 1918 | K | 0.661454018 | Down | Structural maintenance of chromosomes flexible hinge domain-containing protein 1 OS=Homo sapiens OX=9606 GN=SMCHD1 PE=1 SV=2 | SMCHD1 | _SAVC[Carbamidomethyl (C)]K[Acetyl (K)]LDSVNK_ |
| A7KAX9 | 341 | K | 1.683388081 | Up | Rho GTPase-activating protein 32 OS=Homo sapiens OX=9606 GN=ARHGAP32 PE=1 SV=1 | ARHGAP32 | _HGK[Acetyl (K)]LITFLR_ |
| E9PAV3 | 1945 | K | 1.638028119 | Up | Nascent polypeptide-associated complex subunit alpha, muscle-specific form OS=Homo sapiens OX=9606 GN=NACA PE=1 SV=1 | NACA | _AMSK[Acetyl (K)]LGLR_ |
| O00139 | 452 | K | 0.491552893 | Down | Kinesin-like protein KIF2A OS=Homo sapiens OX=9606 GN=KIF2A PE=1 SV=3 | KIF2A | _LHGK[Acetyl (K)]FSLIDLAGNER_ |
| O00139 | 508 | K | 0.655436213 | Down | Kinesin-like protein KIF2A OS=Homo sapiens OX=9606 GN=KIF2A PE=1 SV=3 | KIF2A | _ASK[Acetyl (K)]LTQVLR_ |
| O00148 | 333 | K | 0.458426025 | Down | ATP-dependent RNA helicase DDX39A OS=Homo sapiens OX=9606 GN=DDX39A PE=1 SV=2 | DDX39A | _YQQFK[Acetyl (K)]DFQR_ |
| O00154 | 157 | K | 0.663749584 | Down | Cytosolic acyl coenzyme A thioester hydrolase OS=Homo sapiens OX=9606 GN=ACOT7 PE=1 SV=3 | ACOT7 | _LTNK[Acetyl (K)]ATLWYVPLSLK_ |
| O00159 | 647 | K | 2.249413819 | Up | Unconventional myosin-Ic OS=Homo sapiens OX=9606 GN=MYO1C PE=1 SV=4 | MYO1C | _HQVK[Acetyl (K)]YLGLLENLR_ |
| O00231 | 32 | K | 0.522307061 | Down | 26S proteasome non-ATPase regulatory subunit 11 OS=Homo sapiens OX=9606 GN=PSMD11 PE=1 SV=3 | PSMD11 | _EASIDILHSIVK[Acetyl (K)]R_ |
| O00244 | 25 | K | 0.610511947 | Down | Copper transport protein ATOX1 OS=Homo sapiens OX=9606 GN=ATOX1 PE=1 SV=1 | ATOX1 | _VLNK[Acetyl (K)]LGGVK_ |
| O00264 | 105 | K | 0.421311769 | Down | Membrane-associated progesterone receptor component 1 OS=Homo sapiens OX=9606 GN=PGRMC1 PE=1 SV=3 | PGRMC1 | _K[Acetyl (K)]FYGPEGPYGVFAGR_ |
| O00299 | 20 | K | 0.544524365 | Down | Chloride intracellular channel protein 1 OS=Homo sapiens OX=9606 GN=CLIC1 PE=1 SV=4 | CLIC1 | _AGSDGAK[Acetyl (K)]IGNC[Carbamidomethyl (C)]PFSQR_ |
| O00442 | 163 | K | 1.579894323 | Up | RNA 3'-terminal phosphate cyclase OS=Homo sapiens OX=9606 GN=RTCA PE=1 SV=1 | RTCA | _GYYPK[Acetyl (K)]GGGEVIVR_ |
| O00442 | 191 | K | 0.513761055 | Down | RNA 3'-terminal phosphate cyclase OS=Homo sapiens OX=9606 GN=RTCA PE=1 SV=1 | RTCA | _GC[Carbamidomethyl (C)]VTK[Acetyl (K)]IYGR_ |
| O00483 | 55 | K | 0.661885107 | Down | Cytochrome c oxidase subunit NDUFA4 OS=Homo sapiens OX=9606 GN=NDUFA4 PE=1 SV=1 | NDUFA4 | _NNPEPWNK[Acetyl (K)]LGPNDQYK_ |
| O00499 | 28 | K | 0.569826587 | Down | Myc box-dependent-interacting protein 1 OS=Homo sapiens OX=9606 GN=BIN1 PE=1 SV=1 | BIN1 | _AQEK[Acetyl (K)]VLQK_ |
| O00541 | 68 | K | 0.102736974 | Down | Pescadillo homolog OS=Homo sapiens OX=9606 GN=PES1 PE=1 SV=1 | PES1 | _TFYLIK[Acetyl (K)]DIR_ |
| O00566 | 649 | K | 0.599678673 | Down | U3 small nucleolar ribonucleoprotein protein MPP10 OS=Homo sapiens OX=9606 GN=MPHOSPH10 PE=1 SV=2 | MPHOSPH10 | _SSQAFFSK[Acetyl (K)]LQDQVK_ |
| O00571 | 55 | K | 0.461709048 | Down | ATP-dependent RNA helicase DDX3X OS=Homo sapiens OX=9606 GN=DDX3X PE=1 SV=3 | DDX3X | _GFYDK[Acetyl (K)]DSSGWSSSK_ |
| O14531 | 557 | K | 2.869056663 | Up | Dihydropyrimidinase-related protein 4 OS=Homo sapiens OX=9606 GN=DPYSL4 PE=1 SV=2 | DPYSL4 | _TAQK[Acetyl (K)]IMAPPGGR_ |
| O14548 | 69 | K | 0.499749571 | Down | Cytochrome c oxidase subunit 7A-related protein, mitochondrial OS=Homo sapiens OX=9606 GN=COX7A2L PE=1 SV=2 | COX7A2L | _FFQK[Acetyl (K)]ADGVPVYLK_ |
| O14646 | 1311 | K | 0.542593736 | Down | Chromodomain-helicase-DNA-binding protein 1 OS=Homo sapiens OX=9606 GN=CHD1 PE=1 SV=2 | CHD1 | _ADYLIK[Acetyl (K)]LLSR_ |
| O14656 | 53 | K | 0.440436084 | Down | Torsin-1A OS=Homo sapiens OX=9606 GN=TOR1A PE=1 SV=1 | TOR1A | _LYC[Carbamidomethyl (C)]LFAEC[Carbamidomethyl (C)]C[Carbamidomethyl (C)]GQK[Acetyl (K)]R_ |
| O14734 | 52 | K | 4.884420201 | Up | Acyl-coenzyme A thioesterase 8 OS=Homo sapiens OX=9606 GN=ACOT8 PE=1 SV=1 | ACOT8 | _HYWVPAK[Acetyl (K)]R_ |
| O14737 | 110 | K | 0.586859758 | Down | Programmed cell death protein 5 OS=Homo sapiens OX=9606 GN=PDCD5 PE=1 SV=3 | PDCD5 | _TTTVK[Acetyl (K)]FNR_ |
| O14757 | 461 | K | 2.559731865 | Up | Serine/threonine-protein kinase Chk1 OS=Homo sapiens OX=9606 GN=CHEK1 PE=1 SV=2 | CHEK1 | _GK[Acetyl (K)]LIDIVSSQK_ |
| O14776 | 146 | K | 0.530747751 | Down | Transcription elongation regulator 1 OS=Homo sapiens OX=9606 GN=TCERG1 PE=1 SV=2 | TCERG1 | _TPDGK[Acetyl (K)]VYYYNAR_ |
| O14787 | 862 | K | 1.683084349 | Up | Transportin-2 OS=Homo sapiens OX=9606 GN=TNPO2 PE=1 SV=3 | TNPO2 | _DMFYK[Acetyl (K)]ILHGFK_ |
| O14880 | 111 | K | 0.605782598 | Down | Glutathione S-transferase 3, mitochondrial OS=Homo sapiens OX=9606 GN=MGST3 PE=1 SV=1 | MGST3 | _VLYAYGYYTGEPSK[Acetyl (K)]R_ |
| O14936 | 277 | K | 0.585449065 | Down | Peripheral plasma membrane protein CASK OS=Homo sapiens OX=9606 GN=CASK PE=1 SV=3 | CASK | _ITVYEALNHPWLK[Acetyl (K)]ER_ |
| O14936 | 495 | K | 0.586491891 | Down | Peripheral plasma membrane protein CASK OS=Homo sapiens OX=9606 GN=CASK PE=1 SV=3 | CASK | _LVQFQK[Acetyl (K)]NTDEPMGITLK_ |
| O14974 | 420 | K | 0.581819285 | Down | Protein phosphatase 1 regulatory subunit 12A OS=Homo sapiens OX=9606 GN=PPP1R12A PE=1 SV=1 | PPP1R12A | _FPTTATK[Acetyl (K)]ISPK_ |
| O14979 | 216 | K | 1.612504988 | Up | Heterogeneous nuclear ribonucleoprotein D-like OS=Homo sapiens OX=9606 GN=HNRNPDL PE=1 SV=3 | HNRNPDL | _LDGK[Acetyl (K)]LIDPK_ |
| O14979 | 288 | K | 0.468443054 | Down | Heterogeneous nuclear ribonucleoprotein D-like OS=Homo sapiens OX=9606 GN=HNRNPDL PE=1 SV=3 | HNRNPDL | _GFC[Carbamidomethyl (C)]FITYTDEEPVK[Acetyl (K)]K_ |
| O15031 | 941 | K | 0.452109647 | Down | Plexin-B2 OS=Homo sapiens OX=9606 GN=PLXNB2 PE=1 SV=3 | PLXNB2 | _VTK[Acetyl (K)]FGAQLQC[Carbamidomethyl (C)]VTGPQATR_ |
| O15042 | 88 | K | 0.442788839 | Down | U2 snRNP-associated SURP motif-containing protein OS=Homo sapiens OX=9606 GN=U2SURP PE=1 SV=2 | U2SURP | _AFSIGK[Acetyl (K)]MSTAK_ |
| O15143 | 82 | K | 0.582512128 | Down | Actin-related protein 2/3 complex subunit 1B OS=Homo sapiens OX=9606 GN=ARPC1B PE=1 SV=3 | ARPC1B | _NAYVWTLK[Acetyl (K)]GR_ |
| O15144 | 256 | K | 0.523537832 | Down | Actin-related protein 2/3 complex subunit 2 OS=Homo sapiens OX=9606 GN=ARPC2 PE=1 SV=1 | ARPC2 | _DYLHYHIK[Acetyl (K)]C[Carbamidomethyl (C)]SK_ |
| O15144 | 275 | K | 0.465168469 | Down | Actin-related protein 2/3 complex subunit 2 OS=Homo sapiens OX=9606 GN=ARPC2 PE=1 SV=1 | ARPC2 | _TSDFLK[Acetyl (K)]VLNR_ |
| O15160 | 224 | K | 0.548570347 | Down | DNA-directed RNA polymerases I and III subunit RPAC1 OS=Homo sapiens OX=9606 GN=POLR1C PE=1 SV=1 | POLR1C | _DHAK[Acetyl (K)]FSPVATASYR_ |
| O15164 | 341 | K | 0.459698825 | Down | Transcription intermediary factor 1-alpha OS=Homo sapiens OX=9606 GN=TRIM24 PE=1 SV=3 | TRIM24 | _MK[Acetyl (K)]LMQQQQEVAGLSK_ |
| O15194 | 206 | K | 1.582289238 | Up | CTD small phosphatase-like protein OS=Homo sapiens OX=9606 GN=CTDSPL PE=1 SV=2 | CTDSPL | _GNYVK[Acetyl (K)]DLSR_ |
| O15228 | 146 | K | 0.568402257 | Down | Dihydroxyacetone phosphate acyltransferase OS=Homo sapiens OX=9606 GN=GNPAT PE=1 SV=1 | GNPAT | _VC[Carbamidomethyl (C)]VNEEGIQK[Acetyl (K)]LQR_ |
| O15305 | 26 | K | 0.524211971 | Down | Phosphomannomutase 2 OS=Homo sapiens OX=9606 GN=PMM2 PE=1 SV=1 | PMM2 | _ITK[Acetyl (K)]EMDDFLQK_ |
| O15400 | 24 | K | 0.431677525 | Down | Syntaxin-7 OS=Homo sapiens OX=9606 GN=STX7 PE=1 SV=4 | STX7 | _ISSNIQK[Acetyl (K)]ITQC[Carbamidomethyl (C)]SVEIQR_ |
| O15460 | 516 | K | 2.387564006 | Up | Prolyl 4-hydroxylase subunit alpha-2 OS=Homo sapiens OX=9606 GN=P4HA2 PE=1 SV=1 | P4HA2 | _WVSNK[Acetyl (K)]WFHER_ |
| O43143 | 132 | K | 0.564383197 | Down | ATP-dependent RNA helicase DHX15 OS=Homo sapiens OX=9606 GN=DHX15 PE=1 SV=2 | DHX15 | _YYDILK[Acetyl (K)]K_ |
| O43143 | 786 | K | 0.39070426 | Down | ATP-dependent RNA helicase DHX15 OS=Homo sapiens OX=9606 GN=DHX15 PE=1 SV=2 | DHX15 | _IIAK[Acetyl (K)]LQSK_ |
| O43159 | 389 | K | 1.801930186 | Up | Ribosomal RNA-processing protein 8 OS=Homo sapiens OX=9606 GN=RRP8 PE=1 SV=2 | RRP8 | _VLKPGGLLK[Acetyl (K)]VAEVSSR_ |
| O43175 | 69 | K | 0.604085348 | Down | D-3-phosphoglycerate dehydrogenase OS=Homo sapiens OX=9606 GN=PHGDH PE=1 SV=4 | PHGDH | _VTADVINAAEK[Acetyl (K)]LQVVGR_ |
| O43252 | 12 | K | 0.237445748 | Down | Bifunctional 3'-phosphoadenosine 5'-phosphosulfate synthase 1 OS=Homo sapiens OX=9606 GN=PAPSS1 PE=1 SV=2 | PAPSS1 | _VK[Acetyl (K)]LSNNAQNWGMQR_ |
| O43324 | 166 | K | 1.909550019 | Up | Eukaryotic translation elongation factor 1 epsilon-1 OS=Homo sapiens OX=9606 GN=EEF1E1 PE=1 SV=1 | EEF1E1 | _QHLSSVVFIK[Acetyl (K)]NR_ |
| O43390 | 300 | K | 0.371316399 | Down | Heterogeneous nuclear ribonucleoprotein R OS=Homo sapiens OX=9606 GN=HNRNPR PE=1 SV=1 | HNRNPR | _GFC[Carbamidomethyl (C)]FLEYEDHK[Acetyl (K)]SAAQAR_ |
| O43396 | 181 | K | 0.639905448 | Down | Thioredoxin-like protein 1 OS=Homo sapiens OX=9606 GN=TXNL1 PE=1 SV=3 | TXNL1 | _LYSMK[Acetyl (K)]FQGPDNGQGPK_ |
| O43491 | 83 | K | 2.08313354 | Up | Band 4.1-like protein 2 OS=Homo sapiens OX=9606 GN=EPB41L2 PE=1 SV=1 | EPB41L2 | _FIPPWLK[Acetyl (K)]K_ |
| O43491 | 447 | K | 0.585447126 | Down | Band 4.1-like protein 2 OS=Homo sapiens OX=9606 GN=EPB41L2 PE=1 SV=1 | EPB41L2 | _ILK[Acetyl (K)]ISYK_ |
| O43491 | 458 | K | 0.428606524 | Down | Band 4.1-like protein 2 OS=Homo sapiens OX=9606 GN=EPB41L2 PE=1 SV=1 | EPB41L2 | _SNFYIK[Acetyl (K)]VR_ |
| O43491 | 486 | K | 0.342562271 | Down | Band 4.1-like protein 2 OS=Homo sapiens OX=9606 GN=EPB41L2 PE=1 SV=1 | EPB41L2 | _LWK[Acetyl (K)]VC[Carbamidomethyl (C)]VEHHTFYR_ |
| O43491 | 507 | K | 0.509476675 | Down | Band 4.1-like protein 2 OS=Homo sapiens OX=9606 GN=EPB41L2 PE=1 SV=1 | EPB41L2 | _AK[Acetyl (K)]FLTLGSK_ |
| O43491 | 514 | K | 0.412685017 | Down | Band 4.1-like protein 2 OS=Homo sapiens OX=9606 GN=EPB41L2 PE=1 SV=1 | EPB41L2 | _FLTLGSK[Acetyl (K)]FR_ |
| O43670 | 43 | K | 1.573866161 | Up | BUB3-interacting and GLEBS motif-containing protein ZNF207 OS=Homo sapiens OX=9606 GN=ZNF207 PE=1 SV=1 | ZNF207 | _K[Acetyl (K)]LYTGPGLAIHC[Carbamidomethyl (C)]MQVHK_ |
| O43676 | 69 | K | 0.607874538 | Down | NADH dehydrogenase [ubiquinone] 1 beta subcomplex subunit 3 OS=Homo sapiens OX=9606 GN=NDUFB3 PE=1 SV=3 | NDUFB3 | _SVSFSDVFFK[Acetyl (K)]GFK_ |
| O43678 | 13 | K | 1.616900656 | Up | NADH dehydrogenase [ubiquinone] 1 alpha subcomplex subunit 2 OS=Homo sapiens OX=9606 GN=NDUFA2 PE=1 SV=3 | NDUFA2 | _GVGAK[Acetyl (K)]LGLR_ |
| O43707 | 625 | K | 2.596512475 | Up | Alpha-actinin-4 OS=Homo sapiens OX=9606 GN=ACTN4 PE=1 SV=2 | ACTN4 | _WEK[Acetyl (K)]VQQLVPK_ |
| O43707 | 779 | K | 0.540491412 | Down | Alpha-actinin-4 OS=Homo sapiens OX=9606 GN=ACTN4 PE=1 SV=2 | ACTN4 | _ASFNHFDK[Acetyl (K)]DHGGALGPEEFK_ |
| O43719 | 217 | K | 0.644592688 | Down | HIV Tat-specific factor 1 OS=Homo sapiens OX=9606 GN=HTATSF1 PE=1 SV=1 | HTATSF1 | _LHVEVAK[Acetyl (K)]FQLK_ |
| O43747 | 571 | K | 0.577174384 | Down | AP-1 complex subunit gamma-1 OS=Homo sapiens OX=9606 GN=AP1G1 PE=1 SV=5 | AP1G1 | _AVEYNALFK[Acetyl (K)]K_ |
| O43776 | 116 | K | 0.509940232 | Down | Asparagine--tRNA ligase, cytoplasmic OS=Homo sapiens OX=9606 GN=NARS1 PE=1 SV=1 | NARS1 | _C[Carbamidomethyl (C)]VK[Acetyl (K)]IGALEGYR_ |
| O43837 | 187 | K | 0.557477895 | Down | Isocitrate dehydrogenase [NAD] subunit beta, mitochondrial OS=Homo sapiens OX=9606 GN=IDH3B PE=1 SV=2 | IDH3B | _GVIEC[Carbamidomethyl (C)]LK[Acetyl (K)]IVTR_ |
| O43847 | 694 | K | 0.530465489 | Down | Nardilysin OS=Homo sapiens OX=9606 GN=NRDC PE=1 SV=3 | NRDC | _IVNTPQGC[Carbamidomethyl (C)]LWYK[Acetyl (K)]K_ |
| O60216 | 596 | K | 0.370111507 | Down | Double-strand-break repair protein rad21 homolog OS=Homo sapiens OX=9606 GN=RAD21 PE=1 SV=2 | RAD21 | _QAAAK[Acetyl (K)]FYSFLVLK_ |
| O60231 | 69 | K | 0.411846235 | Down | Pre-mRNA-splicing factor ATP-dependent RNA helicase DHX16 OS=Homo sapiens OX=9606 GN=DHX16 PE=1 SV=2 | DHX16 | _LWNK[Acetyl (K)]VPR_ |
| O60231 | 447 | K | 0.544021897 | Down | Pre-mRNA-splicing factor ATP-dependent RNA helicase DHX16 OS=Homo sapiens OX=9606 GN=DHX16 PE=1 SV=2 | DHX16 | _GMK[Acetyl (K)]IAC[Carbamidomethyl (C)]TQPR_ |
| O60264 | 836 | K | 0.587771236 | Down | SWI/SNF-related matrix-associated actin-dependent regulator of chromatin subfamily A member 5 OS=Homo sapiens OX=9606 GN=SMARCA5 PE=1 SV=1 | SMARCA5 | _EK[Acetyl (K)]LLTQGFTNWNK_ |
| O60287 | 1555 | K | 0.62631975 | Down | Nucleolar pre-ribosomal-associated protein 1 OS=Homo sapiens OX=9606 GN=URB1 PE=1 SV=4 | URB1 | _AYEQNK[Acetyl (K)]LSLINFR_ |
| O60306 | 576 | K | 0.295131165 | Down | RNA helicase aquarius OS=Homo sapiens OX=9606 GN=AQR PE=1 SV=4 | AQR | _PTKPYGTK[Acetyl (K)]FDR_ |
| O60343 | 209 | K | 0.501119689 | Down | TBC1 domain family member 4 OS=Homo sapiens OX=9606 GN=TBC1D4 PE=1 SV=2 | TBC1D4 | _FEVLYC[Carbamidomethyl (C)]GK[Acetyl (K)]VTVTHK_ |
| O60476 | 504 | K | 0.610862275 | Down | Mannosyl-oligosaccharide 1,2-alpha-mannosidase IB OS=Homo sapiens OX=9606 GN=MAN1A2 PE=1 SV=1 | MAN1A2 | _TALK[Acetyl (K)]LGPESFK_ |
| O60488 | 576 | K | 0.14758685 | Down | Long-chain-fatty-acid--CoA ligase 4 OS=Homo sapiens OX=9606 GN=ACSL4 PE=1 SV=2 | ACSL4 | _DLVK[Acetyl (K)]LQAGEYVSLGK_ |
| O60488 | 670 | K | 0.643690302 | Down | Long-chain-fatty-acid--CoA ligase 4 OS=Homo sapiens OX=9606 GN=ACSL4 PE=1 SV=2 | ACSL4 | _FEIPIK[Acetyl (K)]VR_ |
| O60488 | 702 | K | 0.582854535 | Down | Long-chain-fatty-acid--CoA ligase 4 OS=Homo sapiens OX=9606 GN=ACSL4 PE=1 SV=2 | ACSL4 | _NHYLK[Acetyl (K)]DIER_ |
| O60506 | 297 | K | 0.609596783 | Down | Heterogeneous nuclear ribonucleoprotein Q OS=Homo sapiens OX=9606 GN=SYNCRIP PE=1 SV=2 | SYNCRIP | _GFC[Carbamidomethyl (C)]FLEYEDHK[Acetyl (K)]TAAQAR_ |
| O60664 | 296 | K | 0.591766105 | Down | Perilipin-3 OS=Homo sapiens OX=9606 GN=PLIN3 PE=1 SV=3 | PLIN3 | _LVEGQEK[Acetyl (K)]LHQMWLSWNQK_ |
| O60763 | 580 | K | 0.626819155 | Down | General vesicular transport factor p115 OS=Homo sapiens OX=9606 GN=USO1 PE=1 SV=2 | USO1 | _ENFIEK[Acetyl (K)]LGFISK_ |
| O60832 | 203 | K | 1.673522006 | Up | H/ACA ribonucleoprotein complex subunit DKC1 OS=Homo sapiens OX=9606 GN=DKC1 PE=1 SV=3 | DKC1 | _TIYESK[Acetyl (K)]MIEYDPER_ |
| O60841 | 424 | K | 0.662740154 | Down | Eukaryotic translation initiation factor 5B OS=Homo sapiens OX=9606 GN=EIF5B PE=1 SV=4 | EIF5B | _AEATLK[Acetyl (K)]LLQAQGVEVPSK_ |
| O60869 | 135 | K | 1.791294637 | Up | Endothelial differentiation-related factor 1 OS=Homo sapiens OX=9606 GN=EDF1 PE=1 SV=1 | EDF1 | _GK[Acetyl (K)]DIGKPIEK_ |
| O60885 | 1177 | K | 0.535587262 | Down | Bromodomain-containing protein 4 OS=Homo sapiens OX=9606 GN=BRD4 PE=1 SV=2 | BRD4 | _PVIRPPEQNAPPPGAPDK[Acetyl (K)]DK_ |
| O60942 | 502 | K | 0.633620998 | Down | mRNA-capping enzyme OS=Homo sapiens OX=9606 GN=RNGTT PE=1 SV=1 | RNGTT | _PFAQIK[Acetyl (K)]VTK_ |
| O75054 | 679 | K | 1.514733112 | Up | Immunoglobulin superfamily member 3 OS=Homo sapiens OX=9606 GN=IGSF3 PE=2 SV=3 | IGSF3 | _VLQPVTK[Acetyl (K)]LQVSK_ |
| O75063 | 261 | K | 0.482114537 | Down | Glycosaminoglycan xylosylkinase OS=Homo sapiens OX=9606 GN=FAM20B PE=1 SV=1 | FAM20B | _WEYDESYC[Carbamidomethyl (C)]DAVK[Acetyl (K)]K_ |
| O75116 | 229 | K | 1.557025703 | Up | Rho-associated protein kinase 2 OS=Homo sapiens OX=9606 GN=ROCK2 PE=1 SV=4 | ROCK2 | _HGHLK[Acetyl (K)]LADFGTC[Carbamidomethyl (C)]MK_ |
| O75116 | 1368 | K | 0.572678633 | Down | Rho-associated protein kinase 2 OS=Homo sapiens OX=9606 GN=ROCK2 PE=1 SV=4 | ROCK2 | _TSMK[Acetyl (K)]IQQNQSIR_ |
| O75131 | 153 | K | 1.519400058 | Up | Copine-3 OS=Homo sapiens OX=9606 GN=CPNE3 PE=1 SV=1 | CPNE3 | _KLDNK[Acetyl (K)]DLFGK_ |
| O75145 | 997 | K | 1.556835514 | Up | Liprin-alpha-3 OS=Homo sapiens OX=9606 GN=PPFIA3 PE=1 SV=3 | PPFIA3 | _GQLK[Acetyl (K)]MVDSFHR_ |
| O75323 | 75 | K | 0.582288288 | Down | Protein NipSnap homolog 2 OS=Homo sapiens OX=9606 GN=NIPSNAP2 PE=1 SV=1 | NIPSNAP2 | _ETSNLYK[Acetyl (K)]LQFHNVKPEC[Carbamidomethyl (C)]LEAYNK_ |
| O75367 | 123 | K | 1.664722404 | Up | Core histone macro-H2A.1 OS=Homo sapiens OX=9606 GN=MACROH2A1 PE=1 SV=5 | MACROH2A1 | _GK[Acetyl (K)]LEAIITPPPAK_ |
| O75368 | 17 | K | 1.571814166 | Up | Adapter SH3BGRL OS=Homo sapiens OX=9606 GN=SH3BGRL PE=1 SV=1 | SH3BGRL | _VYIASSSGSTAIK[Acetyl (K)]K_ |
| O75369 | 241 | K | 0.453974221 | Down | Filamin-B OS=Homo sapiens OX=9606 GN=FLNB PE=1 SV=2 | FLNB | _AK[Acetyl (K)]LKPGAPLKPK_ |
| O75369 | 948 | K | 0.564455459 | Down | Filamin-B OS=Homo sapiens OX=9606 GN=FLNB PE=1 SV=2 | FLNB | _IK[Acetyl (K)]LNGLENR_ |
| O75369 | 976 | K | 0.665247443 | Down | Filamin-B OS=Homo sapiens OX=9606 GN=FLNB PE=1 SV=2 | FLNB | _GAGGQGK[Acetyl (K)]LDVTILSPSR_ |
| O75475 | 422 | K | 0.645945747 | Down | PC4 and SFRS1-interacting protein OS=Homo sapiens OX=9606 GN=PSIP1 PE=1 SV=1 | PSIP1 | _STMLYNK[Acetyl (K)]FK_ |
| O75531 | 64 | K | 0.252917631 | Down | Barrier-to-autointegration factor OS=Homo sapiens OX=9606 GN=BANF1 PE=1 SV=1 | BANF1 | _EWLK[Acetyl (K)]DTC[Carbamidomethyl (C)]GANAK_ |
| O75533 | 554 | K | 0.585989219 | Down | Splicing factor 3B subunit 1 OS=Homo sapiens OX=9606 GN=SF3B1 PE=1 SV=3 | SF3B1 | _HLLVK[Acetyl (K)]VIDR_ |
| O75569 | 25 | K | 0.44706124 | Down | Interferon-inducible double-stranded RNA-dependent protein kinase activator A OS=Homo sapiens OX=9606 GN=PRKRA PE=1 SV=1 | PRKRA | _EDSGTFSLGK[Acetyl (K)]MITAK_ |
| O75586 | 236 | K | 1.932959931 | Up | Mediator of RNA polymerase II transcription subunit 6 OS=Homo sapiens OX=9606 GN=MED6 PE=1 SV=2 | MED6 | _NVQQTVSAK[Acetyl (K)]GPPEK[Acetyl (K)]R_ |
| O75586 | 241 | K | 1.932959931 | Up | Mediator of RNA polymerase II transcription subunit 6 OS=Homo sapiens OX=9606 GN=MED6 PE=1 SV=2 | MED6 | _NVQQTVSAK[Acetyl (K)]GPPEK[Acetyl (K)]R_ |
| O75643 | 1874 | K | 0.477474454 | Down | U5 small nuclear ribonucleoprotein 200 kDa helicase OS=Homo sapiens OX=9606 GN=SNRNP200 PE=1 SV=2 | SNRNP200 | _QLAQK[Acetyl (K)]VPHK_ |
| O75663 | 19 | K | 0.191276239 | Down | TIP41-like protein OS=Homo sapiens OX=9606 GN=TIPRL PE=1 SV=2 | TIPRL | _DFC[Carbamidomethyl (C)]FGPWK[Acetyl (K)]LTASK_ |
| O75676 | 60 | K | 0.575808667 | Down | Ribosomal protein S6 kinase alpha-4 OS=Homo sapiens OX=9606 GN=RPS6KA4 PE=1 SV=1 | RPS6KA4 | _AGGHDAGK[Acetyl (K)]LYAMK_ |
| O75689 | 176 | K | 1.722090228 | Up | Arf-GAP with dual PH domain-containing protein 1 OS=Homo sapiens OX=9606 GN=ADAP1 PE=1 SV=2 | ADAP1 | _AVMK[Acetyl (K)]IEHLNATFQPAK_ |
| O75694 | 819 | K | 0.474334609 | Down | Nuclear pore complex protein Nup155 OS=Homo sapiens OX=9606 GN=NUP155 PE=1 SV=1 | NUP155 | _ELQEQLK[Acetyl (K)]ITTFK_ |
| O75821 | 153 | K | 9.071375431 | Up | Eukaryotic translation initiation factor 3 subunit G OS=Homo sapiens OX=9606 GN=EIF3G PE=1 SV=2 | EIF3G | _GQK[Acetyl (K)]IVSC[Carbamidomethyl (C)]R_ |
| O75844 | 273 | K | 0.591082993 | Down | CAAX prenyl protease 1 homolog OS=Homo sapiens OX=9606 GN=ZMPSTE24 PE=1 SV=2 | ZMPSTE24 | _SSHSNAYFYGFFK[Acetyl (K)]NK_ |
| O75880 | 218 | K | 0.511172167 | Down | Protein SCO1 homolog, mitochondrial OS=Homo sapiens OX=9606 GN=SCO1 PE=1 SV=1 | SCO1 | _EAIANYVK[Acetyl (K)]EFSPK_ |
| O75976 | 1052 | K | 0.573417596 | Down | Carboxypeptidase D OS=Homo sapiens OX=9606 GN=CPD PE=1 SV=2 | CPD | _DC[Carbamidomethyl (C)]TSK[Acetyl (K)]IGQTNAR_ |
| O76021 | 249 | K | 0.592194722 | Down | Ribosomal L1 domain-containing protein 1 OS=Homo sapiens OX=9606 GN=RSL1D1 PE=1 SV=3 | RSL1D1 | _WESVK[Acetyl (K)]LLFVK_ |
| O76021 | 254 | K | 2.139424335 | Up | Ribosomal L1 domain-containing protein 1 OS=Homo sapiens OX=9606 GN=RSL1D1 PE=1 SV=3 | RSL1D1 | _LLFVK[Acetyl (K)]TEK_ |
| O76054 | 257 | K | 0.617694689 | Down | SEC14-like protein 2 OS=Homo sapiens OX=9606 GN=SEC14L2 PE=1 SV=1 | SEC14L2 | _SK[Acetyl (K)]INYGGDIPR_ |
| O76080 | 209 | K | 0.448020849 | Down | AN1-type zinc finger protein 5 OS=Homo sapiens OX=9606 GN=ZFAND5 PE=1 SV=1 | ZFAND5 | _ENPVVVAEK[Acetyl (K)]IQR_ |
| O76094 | 73 | K | 0.498280964 | Down | Signal recognition particle subunit SRP72 OS=Homo sapiens OX=9606 GN=SRP72 PE=1 SV=3 | SRP72 | _EALNVINTHTK[Acetyl (K)]VLANNSLSFEK_ |
| O94811 | 81 | K | 2.210818975 | Up | Tubulin polymerization-promoting protein OS=Homo sapiens OX=9606 GN=TPPP PE=1 SV=1 | TPPP | _LC[Carbamidomethyl (C)]K[Acetyl (K)]DC[Carbamidomethyl (C)]QVIDGR_ |
| O94826 | 168 | K | 0.66018026 | Down | Mitochondrial import receptor subunit TOM70 OS=Homo sapiens OX=9606 GN=TOMM70 PE=1 SV=1 | TOMM70 | _AAAFEQLQK[Acetyl (K)]WK_ |
| O94826 | 188 | K | 0.122649764 | Down | Mitochondrial import receptor subunit TOM70 OS=Homo sapiens OX=9606 GN=TOMM70 PE=1 SV=1 | TOMM70 | _YVK[Acetyl (K)]ALFR_ |
| O94826 | 501 | K | 0.303846846 | Down | Mitochondrial import receptor subunit TOM70 OS=Homo sapiens OX=9606 GN=TOMM70 PE=1 SV=1 | TOMM70 | _ADEMYDK[Acetyl (K)]C[Carbamidomethyl (C)]IDLEPDNATTYVHK_ |
| O94842 | 285 | K | 1.545935013 | Up | TOX high mobility group box family member 4 OS=Homo sapiens OX=9606 GN=TOX4 PE=1 SV=1 | TOX4 | _EYLK[Acetyl (K)]ALAAYK_ |
| O94855 | 576 | K | 0.364782276 | Down | Protein transport protein Sec24D OS=Homo sapiens OX=9606 GN=SEC24D PE=1 SV=2 | SEC24D | _AADC[Carbamidomethyl (C)]PGK[Acetyl (K)]LFIFHSSLPTAEAPGK_ |
| O94874 | 730 | K | 0.36967273 | Down | E3 UFM1-protein ligase 1 OS=Homo sapiens OX=9606 GN=UFL1 PE=1 SV=2 | UFL1 | _IPEDQHALLVK[Acetyl (K)]YQGLVVK_ |
| O94905 | 192 | K | 2.382702615 | Up | Erlin-2 OS=Homo sapiens OX=9606 GN=ERLIN2 PE=1 SV=1 | ERLIN2 | _TK[Acetyl (K)]LLIAAQK_ |
| O94905 | 232 | K | 0.645105968 | Down | Erlin-2 OS=Homo sapiens OX=9606 GN=ERLIN2 PE=1 SV=1 | ERLIN2 | _VAQVAEITYGQK[Acetyl (K)]VMEK_ |
| O94925 | 158 | K | 0.599943165 | Down | Glutaminase kidney isoform, mitochondrial OS=Homo sapiens OX=9606 GN=GLS PE=1 SV=1 | GLS | _IPVHK[Acetyl (K)]FITALK_ |
| O94979 | 1156 | K | 1.892704989 | Up | Protein transport protein Sec31A OS=Homo sapiens OX=9606 GN=SEC31A PE=1 SV=3 | SEC31A | _LEFLYDK[Acetyl (K)]LR_ |
| O94986 | 1341 | K | 0.319803327 | Down | Centrosomal protein of 152 kDa OS=Homo sapiens OX=9606 GN=CEP152 PE=1 SV=4 | CEP152 | _IMNAASK[Acetyl (K)]LATMAK_ |
| O95163 | 345 | K | 0.564980443 | Down | Elongator complex protein 1 OS=Homo sapiens OX=9606 GN=ELP1 PE=1 SV=3 | ELP1 | _SK[Acetyl (K)]IVSLMWDPVTPYR_ |
| O95163 | 945 | K | 0.636886973 | Down | Elongator complex protein 1 OS=Homo sapiens OX=9606 GN=ELP1 PE=1 SV=3 | ELP1 | _RYEK[Acetyl (K)]AIGHLSK_ |
| O95163 | 1055 | K | 0.637338606 | Down | Elongator complex protein 1 OS=Homo sapiens OX=9606 GN=ELP1 PE=1 SV=3 | ELP1 | _TLAGK[Acetyl (K)]LVEQR_ |
| O95182 | 48 | K | 0.560093433 | Down | NADH dehydrogenase [ubiquinone] 1 alpha subcomplex subunit 7 OS=Homo sapiens OX=9606 GN=NDUFA7 PE=1 SV=3 | NDUFA7 | _LPVGPSHK[Acetyl (K)]LSNNYYC[Carbamidomethyl (C)]TR_ |
| O95197 | 943 | K | 0.390516102 | Down | Reticulon-3 OS=Homo sapiens OX=9606 GN=RTN3 PE=1 SV=2 | RTN3 | _ALK[Acetyl (K)]LIIR_ |
| O95197 | 1022 | K | 0.427075411 | Down | Reticulon-3 OS=Homo sapiens OX=9606 GN=RTN3 PE=1 SV=2 | RTN3 | _IQAK[Acetyl (K)]LPGIAK_ |
| O95210 | 92 | K | 0.110908558 | Down | Starch-binding domain-containing protein 1 OS=Homo sapiens OX=9606 GN=STBD1 PE=1 SV=1 | STBD1 | _DLGK[Acetyl (K)]LQAASWR_ |
| O95219 | 361 | K | 0.648171127 | Down | Sorting nexin-4 OS=Homo sapiens OX=9606 GN=SNX4 PE=1 SV=1 | SNX4 | _GMTTK[Acetyl (K)]LFGQETPEQR_ |
| O95232 | 78 | K | 1.563025224 | Up | Luc7-like protein 3 OS=Homo sapiens OX=9606 GN=LUC7L3 PE=1 SV=2 | LUC7L3 | _FMK[Acetyl (K)]VGYER_ |
| O95235 | 819 | K | 0.623294949 | Down | Kinesin-like protein KIF20A OS=Homo sapiens OX=9606 GN=KIF20A PE=1 SV=1 | KIF20A | _AAC[Carbamidomethyl (C)]IAEQYHTVLK[Acetyl (K)]LQGQVSAK_ |
| O95239 | 635 | K | 1.508558759 | Up | Chromosome-associated kinesin KIF4A OS=Homo sapiens OX=9606 GN=KIF4A PE=1 SV=3 | KIF4A | _TVSK[Acetyl (K)]LNQEIR_ |
| O95249 | 32 | K | 1.577335265 | Up | Golgi SNAP receptor complex member 1 OS=Homo sapiens OX=9606 GN=GOSR1 PE=1 SV=1 | GOSR1 | _LVSFSK[Acetyl (K)]LC[Carbamidomethyl (C)]TSYSHSSTR_ |
| O95249 | 204 | K | 2.099009675 | Up | Golgi SNAP receptor complex member 1 OS=Homo sapiens OX=9606 GN=GOSR1 PE=1 SV=1 | GOSR1 | _SIHSK[Acetyl (K)]MNTLANR_ |
| O95336 | 254 | K | 0.453559055 | Down | 6-phosphogluconolactonase OS=Homo sapiens OX=9606 GN=PGLS PE=1 SV=2 | PGLS | _LLTVPFEK[Acetyl (K)]HSTL_ |
| O95363 | 99 | K | 1.517690851 | Up | Phenylalanine--tRNA ligase, mitochondrial OS=Homo sapiens OX=9606 GN=FARS2 PE=1 SV=1 | FARS2 | _EHFYK[Acetyl (K)]QYVGR_ |
| O95372 | 53 | K | 0.514020022 | Down | Acyl-protein thioesterase 2 OS=Homo sapiens OX=9606 GN=LYPLA2 PE=1 SV=1 | LYPLA2 | _LPHVK[Acetyl (K)]YIC[Carbamidomethyl (C)]PHAPR_ |
| O95394 | 350 | K | 0.299802768 | Down | Phosphoacetylglucosamine mutase OS=Homo sapiens OX=9606 GN=PGM3 PE=1 SV=1 | PGM3 | _VPVYC[Carbamidomethyl (C)]TK[Acetyl (K)]TGVK_ |
| O95453 | 566 | K | 0.483869404 | Down | Poly(A)-specific ribonuclease PARN OS=Homo sapiens OX=9606 GN=PARN PE=1 SV=1 | PARN | _NNSFTAPSTVGK[Acetyl (K)]R_ |
| O95470 | 91 | K | 0.520842726 | Down | Sphingosine-1-phosphate lyase 1 OS=Homo sapiens OX=9606 GN=SGPL1 PE=1 SV=3 | SGPL1 | _IQDK[Acetyl (K)]LNK_ |
| O95478 | 152 | K | 0.61845199 | Down | Ribosome biogenesis protein NSA2 homolog OS=Homo sapiens OX=9606 GN=NSA2 PE=1 SV=1 | NSA2 | _M[Oxidation (M)]VTK[Acetyl (K)]VC[Carbamidomethyl (C)]FVGDGFTR_ |
| O95487 | 849 | K | 0.66059452 | Down | Protein transport protein Sec24B OS=Homo sapiens OX=9606 GN=SEC24B PE=1 SV=2 | SEC24B | _VVQHLGPATDFYK[Acetyl (K)]K_ |
| O95602 | 1095 | K | 0.641470572 | Down | DNA-directed RNA polymerase I subunit RPA1 OS=Homo sapiens OX=9606 GN=POLR1A PE=1 SV=2 | POLR1A | _GAFLSYSQK[Acetyl (K)]IQEAVK_ |
| O95619 | 131 | K | 0.447399703 | Down | YEATS domain-containing protein 4 OS=Homo sapiens OX=9606 GN=YEATS4 PE=1 SV=1 | YEATS4 | _LFQSDTNAMLGK[Acetyl (K)]K_ |
| O95696 | 331 | K | 1.678390972 | Up | Bromodomain-containing protein 1 OS=Homo sapiens OX=9606 GN=BRD1 PE=1 SV=1 | BRD1 | _LTC[Carbamidomethyl (C)]YLC[Carbamidomethyl (C)]K[Acetyl (K)]QK_ |
| O95714 | 1535 | K | 0.608465036 | Down | E3 ubiquitin-protein ligase HERC2 OS=Homo sapiens OX=9606 GN=HERC2 PE=1 SV=2 | HERC2 | _FK[Acetyl (K)]LLSSLPR_ |
| O95758 | 506 | K | 0.428911695 | Down | Polypyrimidine tract-binding protein 3 OS=Homo sapiens OX=9606 GN=PTBP3 PE=1 SV=2 | PTBP3 | _AFK[Acetyl (K)]FFQK_ |
| O95785 | 1112 | K | 1.810093402 | Up | Protein Wiz OS=Homo sapiens OX=9606 GN=WIZ PE=1 SV=2 | WIZ | _ALAK[Acetyl (K)]MMGGAGPGSSLEAR_ |
| O95801 | 37 | K | 1.513553033 | Up | Tetratricopeptide repeat protein 4 OS=Homo sapiens OX=9606 GN=TTC4 PE=1 SV=3 | TTC4 | _GGFHEDQWEK[Acetyl (K)]EFEK_ |
| O95816 | 5 | K | 2.485952183 | Up | BAG family molecular chaperone regulator 2 OS=Homo sapiens OX=9606 GN=BAG2 PE=1 SV=1 | BAG2 | _[Acetyl (Protein N-term)]AQAK[Acetyl (K)]INAK_ |
| O95817 | 230 | K | 0.471928635 | Down | BAG family molecular chaperone regulator 3 OS=Homo sapiens OX=9606 GN=BAG3 PE=1 SV=3 | BAG3 | _PAAQPSFHQAQK[Acetyl (K)]THYPAQQGEYQTHQPVYHK_ |
| O95865 | 51 | K | 0.648158501 | Down | N(G),N(G)-dimethylarginine dimethylaminohydrolase 2 OS=Homo sapiens OX=9606 GN=DDAH2 PE=1 SV=1 | DDAH2 | _EHGVLGGK[Acetyl (K)]LR_ |
| O95983 | 141 | K | 0.263599377 | Down | Methyl-CpG-binding domain protein 3 OS=Homo sapiens OX=9606 GN=MBD3 PE=1 SV=1 | MBD3 | _QLFWEK[Acetyl (K)]K_ |
| O96008 | 102 | K | 0.446779116 | Down | Mitochondrial import receptor subunit TOM40 homolog OS=Homo sapiens OX=9606 GN=TOMM40 PE=1 SV=1 | TOMM40 | _ELFPIQM[Oxidation (M)]EGVK[Acetyl (K)]LTVNK_ |
| O96028 | 1261 | K | 2.924171583 | Up | Histone-lysine N-methyltransferase NSD2 OS=Homo sapiens OX=9606 GN=NSD2 PE=1 SV=1 | NSD2 | _FC[Carbamidomethyl (C)]TK[Acetyl (K)]AYHLSC[Carbamidomethyl (C)]LGLGK_ |
| P00338 | 90 | K | 0.453491562 | Down | L-lactate dehydrogenase A chain OS=Homo sapiens OX=9606 GN=LDHA PE=1 SV=2 | LDHA | _DYNVTANSK[Acetyl (K)]LVIITAGAR_ |
| P00338 | 118 | K | 0.51328562 | Down | L-lactate dehydrogenase A chain OS=Homo sapiens OX=9606 GN=LDHA PE=1 SV=2 | LDHA | _NVNIFK[Acetyl (K)]FIIPNVVK_ |
| P00338 | 126 | K | 0.532094211 | Down | L-lactate dehydrogenase A chain OS=Homo sapiens OX=9606 GN=LDHA PE=1 SV=2 | LDHA | _FIIPNVVK[Acetyl (K)]YSPNC[Carbamidomethyl (C)]K_ |
| P00338 | 222 | K | 0.589531633 | Down | L-lactate dehydrogenase A chain OS=Homo sapiens OX=9606 GN=LDHA PE=1 SV=2 | LDHA | _TLHPDLGTDK[Acetyl (K)]DKEQWK_ |
| P00505 | 396 | K | 0.561179028 | Down | Aspartate aminotransferase, mitochondrial OS=Homo sapiens OX=9606 GN=GOT2 PE=1 SV=3 | GOT2 | _LIK[Acetyl (K)]EFSIYMTK_ |
| P00558 | 48 | K | 0.363293134 | Down | Phosphoglycerate kinase 1 OS=Homo sapiens OX=9606 GN=PGK1 PE=1 SV=3 | PGK1 | _AAVPSIK[Acetyl (K)]FC[Carbamidomethyl (C)]LDNGAK_ |
| P00568 | 27 | K | 0.602623974 | Down | Adenylate kinase isoenzyme 1 OS=Homo sapiens OX=9606 GN=AK1 PE=1 SV=3 | AK1 | _GTQC[Carbamidomethyl (C)]EK[Acetyl (K)]IVQK_ |
| P02545 | 470 | K | 0.467794798 | Down | Prelamin-A/C OS=Homo sapiens OX=9606 GN=LMNA PE=1 SV=1 | LMNA | _SNEDQSMGNWQIK[Acetyl (K)]R_ |
| P02768 | 463 | K | 9.002135647 | Up | Albumin OS=Homo sapiens OX=9606 GN=ALB PE=1 SV=2 | ALB | _NLGKVGSKC[Carbamidomethyl (C)]C[Carbamidomethyl (C)]K[Acetyl (K)]HPEAK[Acetyl (K)]R_ |
| P02768 | 468 | K | 9.002135647 | Up | Albumin OS=Homo sapiens OX=9606 GN=ALB PE=1 SV=2 | ALB | _NLGKVGSKC[Carbamidomethyl (C)]C[Carbamidomethyl (C)]K[Acetyl (K)]HPEAK[Acetyl (K)]R_ |
| P02786 | 189 | K | 1.79171168 | Up | Transferrin receptor protein 1 OS=Homo sapiens OX=9606 GN=TFRC PE=1 SV=2 | TFRC | _DQHFVK[Acetyl (K)]IQVK_ |
| P02786 | 261 | K | 1.663501371 | Up | Transferrin receptor protein 1 OS=Homo sapiens OX=9606 GN=TFRC PE=1 SV=2 | TFRC | _AGK[Acetyl (K)]ITFAEK_ |
| P03928 | 57 | K | 0.647005542 | Down | ATP synthase protein 8 OS=Homo sapiens OX=9606 GN=MT-ATP8 PE=1 SV=1 | MT-ATP8 | _WTK[Acetyl (K)]IC[Carbamidomethyl (C)]SLHSLPPQS_ |
| P04181 | 386 | K | 0.347021593 | Down | Ornithine aminotransferase, mitochondrial OS=Homo sapiens OX=9606 GN=OAT PE=1 SV=1 | OAT | _ETK[Acetyl (K)]DWDAWK_ |
| P04406 | 139 | K | 2.546019381 | Up | Glyceraldehyde-3-phosphate dehydrogenase OS=Homo sapiens OX=9606 GN=GAPDH PE=1 SV=3 | GAPDH | _VIISAPSADAPMFVM[Oxidation (M)]GVNHEK[Acetyl (K)]YDNSLK_ |
| P04424 | 7 | K | 2.011893504 | Up | Argininosuccinate lyase OS=Homo sapiens OX=9606 GN=ASL PE=1 SV=4 | ASL | _[Acetyl (Protein N-term)]ASESGK[Acetyl (K)]LWGGR_ |
| P04818 | 107 | K | 1.53467639 | Up | Thymidylate synthase OS=Homo sapiens OX=9606 GN=TYMS PE=1 SV=3 | TYMS | _GVK[Acetyl (K)]IWDANGSR_ |
| P05023 | 444 | K | 0.665630312 | Down | Sodium/potassium-transporting ATPase subunit alpha-1 OS=Homo sapiens OX=9606 GN=ATP1A1 PE=1 SV=1 | ATP1A1 | _AVFQANQENLPILK[Acetyl (K)]R_ |
| P05023 | 671 | K | 0.421741722 | Down | Sodium/potassium-transporting ATPase subunit alpha-1 OS=Homo sapiens OX=9606 GN=ATP1A1 PE=1 SV=1 | ATP1A1 | _AC[Carbamidomethyl (C)]VVHGSDLK[Acetyl (K)]DMTSEQLDDILK_ |
| P05023 | 773 | K | 0.666540646 | Down | Sodium/potassium-transporting ATPase subunit alpha-1 OS=Homo sapiens OX=9606 GN=ATP1A1 PE=1 SV=1 | ATP1A1 | _LIFDNLK[Acetyl (K)]K_ |
| P05023 | 1019 | K | 0.482969655 | Down | Sodium/potassium-transporting ATPase subunit alpha-1 OS=Homo sapiens OX=9606 GN=ATP1A1 PE=1 SV=1 | ATP1A1 | _RPGGWVEK[Acetyl (K)]ETYY_ |
| P05026 | 216 | K | 0.530027404 | Down | Sodium/potassium-transporting ATPase subunit beta-1 OS=Homo sapiens OX=9606 GN=ATP1B1 PE=1 SV=1 | ATP1B1 | _YNPNVLPVQC[Carbamidomethyl (C)]TGK[Acetyl (K)]R_ |
| P05091 | 383 | K | 0.64284521 | Down | Aldehyde dehydrogenase, mitochondrial OS=Homo sapiens OX=9606 GN=ALDH2 PE=1 SV=2 | ALDH2 | _QEGAK[Acetyl (K)]LLC[Carbamidomethyl (C)]GGGIAADR_ |
| P05141 | 105 | K | 0.478675814 | Down | ADP/ATP translocase 2 OS=Homo sapiens OX=9606 GN=SLC25A5 PE=1 SV=7 | SLC25A5 | _QIFLGGVDK[Acetyl (K)]R_ |
| P05388 | 10 | K | 0.485944609 | Down | Large ribosomal subunit protein uL10 OS=Homo sapiens OX=9606 GN=RPLP0 PE=1 SV=1 | RPLP0 | _ATWK[Acetyl (K)]SNYFLK_ |
| P05388 | 146 | K | 0.435518884 | Down | Large ribosomal subunit protein uL10 OS=Homo sapiens OX=9606 GN=RPLP0 PE=1 SV=1 | RPLP0 | _TSFFQALGITTK[Acetyl (K)]ISR_ |
| P05455 | 360 | K | 0.577289595 | Down | Lupus La protein OS=Homo sapiens OX=9606 GN=SSB PE=1 SV=2 | SSB | _AAQPGSGK[Acetyl (K)]GK[Acetyl (K)]VQFQGK[Acetyl (K)]K_ |
| P05556 | 105 | K | 0.504292866 | Down | Integrin beta-1 OS=Homo sapiens OX=9606 GN=ITGB1 PE=1 SV=2 | ITGB1 | _GTAEK[Acetyl (K)]LKPEDITQIQPQQLVLR_ |
| P05556 | 134 | K | 0.464890325 | Down | Integrin beta-1 OS=Homo sapiens OX=9606 GN=ITGB1 PE=1 SV=2 | ITGB1 | _SGEPQTFTLK[Acetyl (K)]FK_ |
| P05787 | 8 | K | 0.578646228 | Down | Keratin, type II cytoskeletal 8 OS=Homo sapiens OX=9606 GN=KRT8 PE=1 SV=7 | KRT8 | _VTQK[Acetyl (K)]SYK[Acetyl (K)]VSTSGPR_ |
| P05787 | 11 | K | 0.578646228 | Down | Keratin, type II cytoskeletal 8 OS=Homo sapiens OX=9606 GN=KRT8 PE=1 SV=7 | KRT8 | _VTQK[Acetyl (K)]SYK[Acetyl (K)]VSTSGPR_ |
| P05787 | 101 | K | 0.565456907 | Down | Keratin, type II cytoskeletal 8 OS=Homo sapiens OX=9606 GN=KRT8 PE=1 SV=7 | KRT8 | _TLNNK[Acetyl (K)]FASFIDK_ |
| P05787 | 122 | K | 0.506936936 | Down | Keratin, type II cytoskeletal 8 OS=Homo sapiens OX=9606 GN=KRT8 PE=1 SV=7 | KRT8 | _MLETK[Acetyl (K)]WSLLQQQK_ |
| P05787 | 158 | K | 2.226724425 | Up | Keratin, type II cytoskeletal 8 OS=Homo sapiens OX=9606 GN=KRT8 PE=1 SV=7 | KRT8 | _QLETLGQEK[Acetyl (K)]LK_ |
| P06396 | 675 | K | 0.65174021 | Down | Gelsolin OS=Homo sapiens OX=9606 GN=GSN PE=1 SV=1 | GSN | _LFAC[Carbamidomethyl (C)]SNK[Acetyl (K)]IGR_ |
| P06400 | 847 | K | 0.663492744 | Down | Retinoblastoma-associated protein OS=Homo sapiens OX=9606 GN=RB1 PE=1 SV=2 | RB1 | _FQK[Acetyl (K)]INQMVC[Carbamidomethyl (C)]NSDR_ |
| P06400 | 900 | K | 0.652132375 | Down | Retinoblastoma-associated protein OS=Homo sapiens OX=9606 GN=RB1 PE=1 SV=2 | RB1 | _FQQK[Acetyl (K)]LAEMTSTR_ |
| P06493 | 33 | K | 0.553231353 | Down | Cyclin-dependent kinase 1 OS=Homo sapiens OX=9606 GN=CDK1 PE=1 SV=3 | CDK1 | _TTGQVVAMK[Acetyl (K)]K_ |
| P06493 | 274 | K | 0.491127142 | Down | Cyclin-dependent kinase 1 OS=Homo sapiens OX=9606 GN=CDK1 PE=1 SV=3 | CDK1 | _MLIYDPAK[Acetyl (K)]R_ |
| P06493 | 295 | K | 0.450601329 | Down | Cyclin-dependent kinase 1 OS=Homo sapiens OX=9606 GN=CDK1 PE=1 SV=3 | CDK1 | _MALNHPYFNDLDNQIK[Acetyl (K)]K_ |
| P06576 | 124 | K | 1.510163629 | Up | ATP synthase subunit beta, mitochondrial OS=Homo sapiens OX=9606 GN=ATP5F1B PE=1 SV=3 | ATP5F1B | _GQK[Acetyl (K)]VLDSGAPIK_ |
| P06576 | 198 | K | 0.487497105 | Down | ATP synthase subunit beta, mitochondrial OS=Homo sapiens OX=9606 GN=ATP5F1B PE=1 SV=3 | ATP5F1B | _VVDLLAPYAK[Acetyl (K)]GGK_ |
| P06733 | 343 | K | 0.451871601 | Down | Alpha-enolase OS=Homo sapiens OX=9606 GN=ENO1 PE=1 SV=2 | ENO1 | _SC[Carbamidomethyl (C)]NC[Carbamidomethyl (C)]LLLK[Acetyl (K)]VNQIGSVTESLQAC[Carbamidomethyl (C)]K_ |
| P06744 | 252 | K | 2.473444364 | Up | Glucose-6-phosphate isomerase OS=Homo sapiens OX=9606 GN=GPI PE=1 SV=4 | GPI | _HFVALSTNTTK[Acetyl (K)]VK_ |
| P06748 | 24 | K | 1.526387931 | Up | Nucleophosmin OS=Homo sapiens OX=9606 GN=NPM1 PE=1 SV=2 | NPM1 | _PQNYLFGC[Carbamidomethyl (C)]ELK[Acetyl (K)]ADK_ |
| P06748 | 27 | K | 1.672221992 | Up | Nucleophosmin OS=Homo sapiens OX=9606 GN=NPM1 PE=1 SV=2 | NPM1 | _ADK[Acetyl (K)]DYHFK_ |
| P06748 | 150 | K | 1.673548958 | Up | Nucleophosmin OS=Homo sapiens OX=9606 GN=NPM1 PE=1 SV=2 | NPM1 | _SAPGGGSK[Acetyl (K)]VPQK[Acetyl (K)]K_ |
| P06748 | 154 | K | 1.673548958 | Up | Nucleophosmin OS=Homo sapiens OX=9606 GN=NPM1 PE=1 SV=2 | NPM1 | _SAPGGGSK[Acetyl (K)]VPQK[Acetyl (K)]K_ |
| P06748 | 155 | K | 2.97530896 | Up | Nucleophosmin OS=Homo sapiens OX=9606 GN=NPM1 PE=1 SV=2 | NPM1 | _SAPGGGSK[Acetyl (K)]VPQK[Acetyl (K)]K[Acetyl (K)]VK_ |
| P06748 | 223 | K | 2.014957543 | Up | Nucleophosmin OS=Homo sapiens OX=9606 GN=NPM1 PE=1 SV=2 | NPM1 | _SK[Acetyl (K)]GQESFK[Acetyl (K)]K_ |
| P06748 | 229 | K | 2.014957543 | Up | Nucleophosmin OS=Homo sapiens OX=9606 GN=NPM1 PE=1 SV=2 | NPM1 | _SK[Acetyl (K)]GQESFK[Acetyl (K)]K_ |
| P06748 | 267 | K | 2.00433597 | Up | Nucleophosmin OS=Homo sapiens OX=9606 GN=NPM1 PE=1 SV=2 | NPM1 | _VEAK[Acetyl (K)]FINYVK_ |
| P06756 | 499 | K | 0.636977539 | Down | Integrin alpha-V OS=Homo sapiens OX=9606 GN=ITGAV PE=1 SV=2 | ITGAV | _TC[Carbamidomethyl (C)]SLPGTALK[Acetyl (K)]VSC[Carbamidomethyl (C)]FNVR_ |
| P07195 | 308 | K | 1.816942284 | Up | L-lactate dehydrogenase B chain OS=Homo sapiens OX=9606 GN=LDHB PE=1 SV=2 | LDHB | _GLTSVINQK[Acetyl (K)]LK_ |
| P07305 | 59 | K | 1.592050807 | Up | Histone H1.0 OS=Homo sapiens OX=9606 GN=H1-0 PE=1 SV=3 | H1-0 | _SHYK[Acetyl (K)]VGENADSQIK_ |
| P07339 | 28 | K | 0.60319261 | Down | Cathepsin D OS=Homo sapiens OX=9606 GN=CTSD PE=1 SV=1 | CTSD | _IPLHK[Acetyl (K)]FTSIR_ |
| P07355 | 313 | K | 0.632049712 | Down | Annexin A2 OS=Homo sapiens OX=9606 GN=ANXA2 PE=1 SV=2 | ANXA2 | _KYGK[Acetyl (K)]SLYYYIQQDTK_ |
| P07437 | 58 | K | 1.723206229 | Up | Tubulin beta chain OS=Homo sapiens OX=9606 GN=TUBB PE=1 SV=2 | TUBB | _ISVYYNEATGGK[Acetyl (K)]YVPR_ |
| P07814 | 435 | K | 0.637800314 | Down | Bifunctional glutamate/proline--tRNA ligase OS=Homo sapiens OX=9606 GN=EPRS1 PE=1 SV=5 | EPRS1 | _LNLNNTVLSK[Acetyl (K)]R_ |
| P07814 | 1503 | K | 0.463462633 | Down | Bifunctional glutamate/proline--tRNA ligase OS=Homo sapiens OX=9606 GN=EPRS1 PE=1 SV=5 | EPRS1 | _NPAK[Acetyl (K)]YYTLFGR_ |
| P07858 | 209 | K | 0.598515436 | Down | Cathepsin B OS=Homo sapiens OX=9606 GN=CTSB PE=1 SV=3 | CTSB | _C[Carbamidomethyl (C)]SK[Acetyl (K)]IC[Carbamidomethyl (C)]EPGYSPTYK_ |
| P07900 | 191 | K | 0.637171714 | Down | Heat shock protein HSP 90-alpha OS=Homo sapiens OX=9606 GN=HSP90AA1 PE=1 SV=5 | HSP90AA1 | _VILHLK[Acetyl (K)]EDQTEYLEER_ |
| P07900 | 362 | K | 0.577325677 | Down | Heat shock protein HSP 90-alpha OS=Homo sapiens OX=9606 GN=HSP90AA1 PE=1 SV=5 | HSP90AA1 | _NNIK[Acetyl (K)]LYVR_ |
| P07900 | 446 | K | 1.554005666 | Up | Heat shock protein HSP 90-alpha OS=Homo sapiens OX=9606 GN=HSP90AA1 PE=1 SV=5 | HSP90AA1 | _NIK[Acetyl (K)]LGIHEDSQNR_ |
| P07910 | 42 | K | 1.910113286 | Up | Heterogeneous nuclear ribonucleoproteins C1/C2 OS=Homo sapiens OX=9606 GN=HNRNPC PE=1 SV=4 | HNRNPC | _YGK[Acetyl (K)]IVGC[Carbamidomethyl (C)]SVHK_ |
| P08237 | 678 | K | 1.732653144 | Up | ATP-dependent 6-phosphofructokinase, muscle type OS=Homo sapiens OX=9606 GN=PFKM PE=1 SV=2 | PFKM | _NFATK[Acetyl (K)]MGAK_ |
| P08237 | 738 | K | 0.619050989 | Down | ATP-dependent 6-phosphofructokinase, muscle type OS=Homo sapiens OX=9606 GN=PFKM PE=1 SV=2 | PFKM | _IPK[Acetyl (K)]EQWWLK_ |
| P08238 | 72 | K | 0.554360738 | Down | Heat shock protein HSP 90-beta OS=Homo sapiens OX=9606 GN=HSP90AB1 PE=1 SV=4 | HSP90AB1 | _ELK[Acetyl (K)]IDIIPNPQER_ |
| P08238 | 347 | K | 0.444154343 | Down | Heat shock protein HSP 90-beta OS=Homo sapiens OX=9606 GN=HSP90AB1 PE=1 SV=4 | HSP90AB1 | _APFDLFENK[Acetyl (K)]K_ |
| P08574 | 318 | K | 0.623575185 | Down | Cytochrome c1, heme protein, mitochondrial OS=Homo sapiens OX=9606 GN=CYC1 PE=1 SV=3 | CYC1 | _K[Acetyl (K)]LAYRPPK_ |
| P08579 | 85 | K | 0.602599388 | Down | U2 small nuclear ribonucleoprotein B'' OS=Homo sapiens OX=9606 GN=SNRPB2 PE=1 SV=1 | SNRPB2 | _IQYAK[Acetyl (K)]TDSDIISK_ |
| P08579 | 219 | K | 0.649338493 | Down | U2 small nuclear ribonucleoprotein B'' OS=Homo sapiens OX=9606 GN=SNRPB2 PE=1 SV=1 | SNRPB2 | _ITPSHAMK[Acetyl (K)]ITYAK_ |
| P08708 | 72 | K | 1.666222955 | Up | Small ribosomal subunit protein eS17 OS=Homo sapiens OX=9606 GN=RPS17 PE=1 SV=2 | RPS17 | _GISIK[Acetyl (K)]LQEEER_ |
| P08727 | 215 | K | 2.141414739 | Up | Keratin, type I cytoskeletal 19 OS=Homo sapiens OX=9606 GN=KRT19 PE=1 SV=4 | KRT19 | _EELAYLK[Acetyl (K)]K_ |
| P08754 | 92 | K | 0.665187826 | Down | Guanine nucleotide-binding protein G(i) subunit alpha-3 OS=Homo sapiens OX=9606 GN=GNAI3 PE=1 SV=3 | GNAI3 | _LK[Acetyl (K)]IDFGEAAR_ |
| P08754 | 197 | K | 0.382958533 | Down | Guanine nucleotide-binding protein G(i) subunit alpha-3 OS=Homo sapiens OX=9606 GN=GNAI3 PE=1 SV=3 | GNAI3 | _DLYFK[Acetyl (K)]MFDVGGQR_ |
| P09012 | 276 | K | 1.532464036 | Up | U1 small nuclear ribonucleoprotein A OS=Homo sapiens OX=9606 GN=SNRPA PE=1 SV=3 | SNRPA | _ITQNNAMK[Acetyl (K)]ISFAK_ |
| P09429 | 12 | K | 0.301954606 | Down | High mobility group protein B1 OS=Homo sapiens OX=9606 GN=HMGB1 PE=1 SV=3 | HMGB1 | _GK[Acetyl (K)]MSSYAFFVQTC[Carbamidomethyl (C)]R_ |
| P09429 | 43 | K | 1.778459171 | Up | High mobility group protein B1 OS=Homo sapiens OX=9606 GN=HMGB1 PE=1 SV=3 | HMGB1 | _KHPDASVNFSEFSK[Acetyl (K)]K_ |
| P09525 | 253 | K | 1.56325713 | Up | Annexin A4 OS=Homo sapiens OX=9606 GN=ANXA4 PE=1 SV=4 | ANXA4 | _SAYFAEK[Acetyl (K)]LYK_ |
| P09543 | 371 | K | 2.538357911 | Up | 2',3'-cyclic-nucleotide 3'-phosphodiesterase OS=Homo sapiens OX=9606 GN=CNP PE=1 SV=2 | CNP | _GK[Acetyl (K)]LYSLGNGR_ |
| P09661 | 149 | K | 0.595983216 | Down | U2 small nuclear ribonucleoprotein A' OS=Homo sapiens OX=9606 GN=SNRPA1 PE=1 SV=2 | SNRPA1 | _VLDFQK[Acetyl (K)]VK_ |
| P09669 | 60 | K | 0.463539856 | Down | Cytochrome c oxidase subunit 6C OS=Homo sapiens OX=9606 GN=COX6C PE=1 SV=2 | COX6C | _NYDVMK[Acetyl (K)]DFEEMR_ |
| P09874 | 165 | K | 0.618004824 | Down | Poly [ADP-ribose] polymerase 1 OS=Homo sapiens OX=9606 GN=PARP1 PE=1 SV=4 | PARP1 | _WYHPGC[Carbamidomethyl (C)]FVK[Acetyl (K)]NR_ |
| P09874 | 400 | K | 1.59622287 | Up | Poly [ADP-ribose] polymerase 1 OS=Homo sapiens OX=9606 GN=PARP1 PE=1 SV=4 | PARP1 | _ILTLGK[Acetyl (K)]LSR_ |
| P09874 | 418 | K | 0.540969483 | Down | Poly [ADP-ribose] polymerase 1 OS=Homo sapiens OX=9606 GN=PARP1 PE=1 SV=4 | PARP1 | _LGGK[Acetyl (K)]LTGTANK_ |
| P09874 | 551 | K | 0.522754265 | Down | Poly [ADP-ribose] polymerase 1 OS=Homo sapiens OX=9606 GN=PARP1 PE=1 SV=4 | PARP1 | _GGK[Acetyl (K)]VFSATLGLVDIVK_ |
| P09960 | 58 | K | 0.384655242 | Down | Leukotriene A-4 hydrolase OS=Homo sapiens OX=9606 GN=LTA4H PE=1 SV=2 | LTA4H | _SLVLDTK[Acetyl (K)]DLTIEK_ |
| P09960 | 414 | K | 1.981228758 | Up | Leukotriene A-4 hydrolase OS=Homo sapiens OX=9606 GN=LTA4H PE=1 SV=2 | LTA4H | _AYVEK[Acetyl (K)]FSYK_ |
| P0C0S8 | 96 | K | 1.630825104 | Up | Histone H2A type 1 OS=Homo sapiens OX=9606 GN=H2AC11 PE=1 SV=2 | H2AC11 | _NDEELNK[Acetyl (K)]LLGK_ |
| P0DMV8 | 71 | K | 0.60465847 | Down | Heat shock 70 kDa protein 1A OS=Homo sapiens OX=9606 GN=HSPA1A PE=1 SV=1 | HSPA1A | _NQVALNPQNTVFDAK[Acetyl (K)]R_ |
| P0DP23 | 95 | K | 0.473688114 | Down | Calmodulin-1 OS=Homo sapiens OX=9606 GN=CALM1 PE=1 SV=1 | CALM1 | _VFDK[Acetyl (K)]DGNGYISAAELR_ |
| P10074 | 192 | K | 1.933822339 | Up | Telomere zinc finger-associated protein OS=Homo sapiens OX=9606 GN=ZBTB48 PE=1 SV=2 | ZBTB48 | _GSHSPQRPQLHSPAQSEGPSSLC[Carbamidomethyl (C)]GK[Acetyl (K)]LK_ |
| P10253 | 162 | K | 2.227053976 | Up | Lysosomal alpha-glucosidase OS=Homo sapiens OX=9606 GN=GAA PE=1 SV=4 | GAA | _TTPTFFPK[Acetyl (K)]DILTLR_ |
| P10301 | 31 | K | 0.487996408 | Down | Ras-related protein R-Ras OS=Homo sapiens OX=9606 GN=RRAS PE=1 SV=1 | RRAS | _GGGPGPGDPPPSETHK[Acetyl (K)]LVVVGGGGVGK_ |
| P10412 | 17 | K | 2.642804462 | Up | Histone H1.4 OS=Homo sapiens OX=9606 GN=H1-4 PE=1 SV=2 | H1-4 | _[Acetyl (Protein N-term)]SETAPAAPAAPAPAEK[Acetyl (K)]TPVK[Acetyl (K)]K[Acetyl (K)]K_ |
| P10412 | 21 | K | 2.642804462 | Up | Histone H1.4 OS=Homo sapiens OX=9606 GN=H1-4 PE=1 SV=2 | H1-4 | _[Acetyl (Protein N-term)]SETAPAAPAAPAPAEK[Acetyl (K)]TPVK[Acetyl (K)]K[Acetyl (K)]K_ |
| P10412 | 22 | K | 2.642804462 | Up | Histone H1.4 OS=Homo sapiens OX=9606 GN=H1-4 PE=1 SV=2 | H1-4 | _[Acetyl (Protein N-term)]SETAPAAPAAPAPAEK[Acetyl (K)]TPVK[Acetyl (K)]K[Acetyl (K)]K_ |
| P10412 | 106 | K | 0.3703055 | Down | Histone H1.4 OS=Homo sapiens OX=9606 GN=H1-4 PE=1 SV=2 | H1-4 | _GTGASGSFK[Acetyl (K)]LNK_ |
| P10515 | 362 | K | 0.500935912 | Down | Dihydrolipoyllysine-residue acetyltransferase component of pyruvate dehydrogenase complex, mitochondrial OS=Homo sapiens OX=9606 GN=DLAT PE=1 SV=3 | DLAT | _VFVSPLAK[Acetyl (K)]K_ |
| P10599 | 81 | K | 0.369589247 | Down | Thioredoxin OS=Homo sapiens OX=9606 GN=TXN PE=1 SV=3 | TXN | _C[Carbamidomethyl (C)]MPTFQFFK[Acetyl (K)]K_ |
| P10599 | 85 | K | 0.12455133 | Down | Thioredoxin OS=Homo sapiens OX=9606 GN=TXN PE=1 SV=3 | TXN | _GQK[Acetyl (K)]VGEFSGANK_ |
| P10644 | 222 | K | 0.611537045 | Down | cAMP-dependent protein kinase type I-alpha regulatory subunit OS=Homo sapiens OX=9606 GN=PRKAR1A PE=1 SV=1 | PRKAR1A | _TNVK[Acetyl (K)]LWGIDR_ |
| P10809 | 72 | K | 0.613890323 | Down | 60 kDa heat shock protein, mitochondrial OS=Homo sapiens OX=9606 GN=HSPD1 PE=1 SV=2 | HSPD1 | _TVIIEQSWGSPK[Acetyl (K)]VTK_ |
| P10809 | 352 | K | 0.643736476 | Down | 60 kDa heat shock protein, mitochondrial OS=Homo sapiens OX=9606 GN=HSPD1 PE=1 SV=2 | HSPD1 | _VGEVIVTK[Acetyl (K)]DDAMLLK_ |
| P11021 | 523 | K | 0.517953025 | Down | Endoplasmic reticulum chaperone BiP OS=Homo sapiens OX=9606 GN=HSPA5 PE=1 SV=2 | HSPA5 | _NK[Acetyl (K)]ITITNDQNR_ |
| P11142 | 71 | K | 0.49312996 | Down | Heat shock cognate 71 kDa protein OS=Homo sapiens OX=9606 GN=HSPA8 PE=1 SV=1 | HSPA8 | _NQVAMNPTNTVFDAK[Acetyl (K)]R_ |
| P11142 | 246 | K | 0.657997603 | Down | Heat shock cognate 71 kDa protein OS=Homo sapiens OX=9606 GN=HSPA8 PE=1 SV=1 | HSPA8 | _MVNHFIAEFK[Acetyl (K)]R_ |
| P11171 | 439 | K | 0.470267895 | Down | Protein 4.1 OS=Homo sapiens OX=9606 GN=EPB41 PE=1 SV=4 | EPB41 | _VLK[Acetyl (K)]ISYK_ |
| P11216 | 42 | K | 0.328489297 | Down | Glycogen phosphorylase, brain form OS=Homo sapiens OX=9606 GN=PYGB PE=1 SV=5 | PYGB | _HLHFTLVK[Acetyl (K)]DR_ |
| P11216 | 316 | K | 0.606397092 | Down | Glycogen phosphorylase, brain form OS=Homo sapiens OX=9606 GN=PYGB PE=1 SV=5 | PYGB | _SSK[Acetyl (K)]FGC[Carbamidomethyl (C)]R_ |
| P11233 | 166 | K | 0.425904863 | Down | Ras-related protein Ral-A OS=Homo sapiens OX=9606 GN=RALA PE=1 SV=1 | RALA | _ANVDK[Acetyl (K)]VFFDLMR_ |
| P11310 | 279 | K | 2.00187989 | Up | Medium-chain specific acyl-CoA dehydrogenase, mitochondrial OS=Homo sapiens OX=9606 GN=ACADM PE=1 SV=1 | ACADM | _VAMGAFDK[Acetyl (K)]TRPVVAAGAVGLAQR_ |
| P11387 | 218 | K | 0.429372508 | Down | DNA topoisomerase 1 OS=Homo sapiens OX=9606 GN=TOP1 PE=1 SV=2 | TOP1 | _WK[Acetyl (K)]FLEHK_ |
| P11387 | 245 | K | 1.596875832 | Up | DNA topoisomerase 1 OS=Homo sapiens OX=9606 GN=TOP1 PE=1 SV=2 | TOP1 | _FYYDGK[Acetyl (K)]VMK_ |
| P11387 | 540 | K | 1.969923949 | Up | DNA topoisomerase 1 OS=Homo sapiens OX=9606 GN=TOP1 PE=1 SV=2 | TOP1 | _YYNK[Acetyl (K)]VPVEK_ |
| P11388 | 1075 | K | 0.631289425 | Down | DNA topoisomerase 2-alpha OS=Homo sapiens OX=9606 GN=TOP2A PE=1 SV=3 | TOP2A | _ELIK[Acetyl (K)]VLIQR_ |
| P11441 | 66 | K | 1.701878952 | Up | Ubiquitin-like protein 4A OS=Homo sapiens OX=9606 GN=UBL4A PE=1 SV=1 | UBL4A | _LSDYSIGPNSK[Acetyl (K)]LNLVVKPLEK_ |
| P11474 | 403 | K | 0.123715856 | Down | Steroid hormone receptor ERR1 OS=Homo sapiens OX=9606 GN=ESRRA PE=1 SV=3 | ESRRA | _VLAHFYGVK[Acetyl (K)]LEGK_ |
| P11586 | 175 | K | 0.61567931 | Down | C-1-tetrahydrofolate synthase, cytoplasmic OS=Homo sapiens OX=9606 GN=MTHFD1 PE=1 SV=4 | MTHFD1 | _SK[Acetyl (K)]IVGAPMHDLLLWNNATVTTC[Carbamidomethyl (C)]HSK_ |
| P11717 | 1924 | K | 0.534219261 | Down | Cation-independent mannose-6-phosphate receptor OS=Homo sapiens OX=9606 GN=IGF2R PE=1 SV=3 | IGF2R | _AK[Acetyl (K)]LWC[Carbamidomethyl (C)]STTADYDR_ |
| P11926 | 57 | K | 1.516826868 | Up | Ornithine decarboxylase OS=Homo sapiens OX=9606 GN=ODC1 PE=1 SV=2 | ODC1 | _WLK[Acetyl (K)]ALPR_ |
| P12081 | 154 | K | 0.210746932 | Down | Histidine--tRNA ligase, cytoplasmic OS=Homo sapiens OX=9606 GN=HARS1 PE=1 SV=2 | HARS1 | _YHIAK[Acetyl (K)]VYR_ |
| P12236 | 105 | K | 0.547733341 | Down | ADP/ATP translocase 3 OS=Homo sapiens OX=9606 GN=SLC25A6 PE=1 SV=4 | SLC25A6 | _QIFLGGVDK[Acetyl (K)]HTQFWR_ |
| P12268 | 511 | K | 0.057488906 | Down | Inosine-5'-monophosphate dehydrogenase 2 OS=Homo sapiens OX=9606 GN=IMPDH2 PE=1 SV=2 | IMPDH2 | _TSSAQVEGGVHSLHSYEK[Acetyl (K)]R_ |
| P12270 | 1105 | K | 0.630194657 | Down | Nucleoprotein TPR OS=Homo sapiens OX=9606 GN=TPR PE=1 SV=3 | TPR | _EQVSK[Acetyl (K)]MASVR_ |
| P12270 | 1563 | K | 0.624766644 | Down | Nucleoprotein TPR OS=Homo sapiens OX=9606 GN=TPR PE=1 SV=3 | TPR | _SK[Acetyl (K)]IAHLAGVK_ |
| P12277 | 313 | K | 0.500038102 | Down | Creatine kinase B-type OS=Homo sapiens OX=9606 GN=CKB PE=1 SV=1 | CKB | _FSEVLK[Acetyl (K)]R_ |
| P12532 | 331 | K | 2.084708748 | Up | Creatine kinase U-type, mitochondrial OS=Homo sapiens OX=9606 GN=CKMT1A PE=1 SV=1 | CKMT1A | _AGVHIK[Acetyl (K)]LPLLSK_ |
| P12532 | 416 | K | 1.735153758 | Up | Creatine kinase U-type, mitochondrial OS=Homo sapiens OX=9606 GN=CKMT1A PE=1 SV=1 | CKMT1A | _IPTPVIHTK[Acetyl (K)]H_ |
| P12830 | 215 | K | 2.100230147 | Up | Cadherin-1 OS=Homo sapiens OX=9606 GN=CDH1 PE=1 SV=3 | CDH1 | _ETGWLK[Acetyl (K)]VTEPLDR_ |
| P12830 | 668 | K | 1.845101576 | Up | Cadherin-1 OS=Homo sapiens OX=9606 GN=CDH1 PE=1 SV=3 | CDH1 | _INLK[Acetyl (K)]LMDNQNK_ |
| P12883 | 657 | K | 2.84674202 | Up | Myosin-7 OS=Homo sapiens OX=9606 GN=MYH7 PE=1 SV=5 | MYH7 | _ENLNK[Acetyl (K)]LMTNLR_ |
| P12883 | 1305 | K | 1.76562975 | Up | Myosin-7 OS=Homo sapiens OX=9606 GN=MYH7 PE=1 SV=5 | MYH7 | _GK[Acetyl (K)]LTYTQQLEDLKR_ |
| P12956 | 114 | K | 0.338435183 | Down | X-ray repair cross-complementing protein 6 OS=Homo sapiens OX=9606 GN=XRCC6 PE=1 SV=2 | XRCC6 | _NIYVLQELDNPGAK[Acetyl (K)]R_ |
| P12956 | 317 | K | 0.600195409 | Down | X-ray repair cross-complementing protein 6 OS=Homo sapiens OX=9606 GN=XRCC6 PE=1 SV=2 | XRCC6 | _TFNTSTGGLLLPSDTK[Acetyl (K)]R_ |
| P12956 | 357 | K | 0.531951746 | Down | X-ray repair cross-complementing protein 6 OS=Homo sapiens OX=9606 GN=XRCC6 PE=1 SV=2 | XRCC6 | _PLVLLK[Acetyl (K)]K_ |
| P13010 | 347 | K | 0.510274489 | Down | X-ray repair cross-complementing protein 5 OS=Homo sapiens OX=9606 GN=XRCC5 PE=1 SV=3 | XRCC5 | _C[Carbamidomethyl (C)]FSVLGFC[Carbamidomethyl (C)]K[Acetyl (K)]SSQVQR_ |
| P13010 | 363 | K | 0.414839367 | Down | X-ray repair cross-complementing protein 5 OS=Homo sapiens OX=9606 GN=XRCC5 PE=1 SV=3 | XRCC5 | _FFMGNQVLK[Acetyl (K)]VFAAR_ |
| P13639 | 426 | K | 1.862314838 | Up | Elongation factor 2 OS=Homo sapiens OX=9606 GN=EEF2 PE=1 SV=4 | EEF2 | _VFSGLVSTGLK[Acetyl (K)]VR_ |
| P13639 | 438 | K | 0.458132962 | Down | Elongation factor 2 OS=Homo sapiens OX=9606 GN=EEF2 PE=1 SV=4 | EEF2 | _IMGPNYTPGK[Acetyl (K)]K_ |
| P13639 | 571 | K | 0.156833466 | Down | Elongation factor 2 OS=Homo sapiens OX=9606 GN=EEF2 PE=1 SV=4 | EEF2 | _DLEEDHAC[Carbamidomethyl (C)]IPIK[Acetyl (K)]K_ |
| P13667 | 103 | K | 0.649883598 | Down | Protein disulfide-isomerase A4 OS=Homo sapiens OX=9606 GN=PDIA4 PE=1 SV=2 | PDIA4 | _QFAPEYEK[Acetyl (K)]IANILK_ |
| P13693 | 93 | K | 4.766072139 | Up | Translationally-controlled tumor protein OS=Homo sapiens OX=9606 GN=TPT1 PE=1 SV=1 | TPT1 | _YIK[Acetyl (K)]DYMK_ |
| P13693 | 97 | K | 3.499345418 | Up | Translationally-controlled tumor protein OS=Homo sapiens OX=9606 GN=TPT1 PE=1 SV=1 | TPT1 | _DYMK[Acetyl (K)]SIK_ |
| P13995 | 152 | K | 0.653516934 | Down | Bifunctional methylenetetrahydrofolate dehydrogenase/cyclohydrolase, mitochondrial OS=Homo sapiens OX=9606 GN=MTHFD2 PE=1 SV=2 | MTHFD2 | _IC[Carbamidomethyl (C)]NAVSPDK[Acetyl (K)]DVDGFHVINVGR_ |
| P14618 | 141 | K | 0.46618888 | Down | Pyruvate kinase PKM OS=Homo sapiens OX=9606 GN=PKM PE=1 SV=4 | PKM | _GATLK[Acetyl (K)]ITLDNAYMEK_ |
| P14618 | 433 | K | 1.545467 | Up | Pyruvate kinase PKM OS=Homo sapiens OX=9606 GN=PKM PE=1 SV=4 | PKM | _C[Carbamidomethyl (C)]C[Carbamidomethyl (C)]SGAIIVLTK[Acetyl (K)]SGR_ |
| P14625 | 404 | K | 0.409249983 | Down | Endoplasmin OS=Homo sapiens OX=9606 GN=HSP90B1 PE=1 SV=1 | HSP90B1 | _GLFDEYGSK[Acetyl (K)]K_ |
| P14625 | 410 | K | 0.531738826 | Down | Endoplasmin OS=Homo sapiens OX=9606 GN=HSP90B1 PE=1 SV=1 | HSP90B1 | _SDYIK[Acetyl (K)]LYVR_ |
| P14625 | 473 | K | 0.498776582 | Down | Endoplasmin OS=Homo sapiens OX=9606 GN=HSP90B1 PE=1 SV=1 | HSP90B1 | _TLDMIK[Acetyl (K)]K_ |
| P14625 | 682 | K | 0.665683535 | Down | Endoplasmin OS=Homo sapiens OX=9606 GN=HSP90B1 PE=1 SV=1 | HSP90B1 | _DISTNYYASQK[Acetyl (K)]K_ |
| P14678 | 8 | K | 0.282656636 | Down | Small nuclear ribonucleoprotein-associated proteins B and B' OS=Homo sapiens OX=9606 GN=SNRPB PE=1 SV=2 | SNRPB | _SSK[Acetyl (K)]MLQHIDYR_ |
| P14678 | 36 | K | 0.149888853 | Down | Small nuclear ribonucleoprotein-associated proteins B and B' OS=Homo sapiens OX=9606 GN=SNRPB PE=1 SV=2 | SNRPB | _AFDK[Acetyl (K)]HMNLILC[Carbamidomethyl (C)]DC[Carbamidomethyl (C)]DEFR_ |
| P14866 | 34 | K | 0.654287445 | Down | Heterogeneous nuclear ribonucleoprotein L OS=Homo sapiens OX=9606 GN=HNRNPL PE=1 SV=2 | HNRNPL | _SGAMVK[Acetyl (K)]MAAAGGGGGGGR_ |
| P14866 | 269 | K | 0.485649586 | Down | Heterogeneous nuclear ribonucleoprotein L OS=Homo sapiens OX=9606 GN=HNRNPL PE=1 SV=2 | HNRNPL | _IEYAK[Acetyl (K)]PTR_ |
| P15531 | 39 | K | 0.65769189 | Down | Nucleoside diphosphate kinase A OS=Homo sapiens OX=9606 GN=NME1 PE=1 SV=1 | NME1 | _LVGLK[Acetyl (K)]FMQASEDLLK_ |
| P15924 | 154 | K | 0.525464041 | Down | Desmoplakin OS=Homo sapiens OX=9606 GN=DSP PE=1 SV=3 | DSP | _ALYK[Acetyl (K)]AISVPR_ |
| P15924 | 1818 | K | 0.663782844 | Down | Desmoplakin OS=Homo sapiens OX=9606 GN=DSP PE=1 SV=3 | DSP | _ESLLVK[Acetyl (K)]IK_ |
| P16144 | 46 | K | 0.427227309 | Down | Integrin beta-4 OS=Homo sapiens OX=9606 GN=ITGB4 PE=1 SV=5 | ITGB4 | _VDK[Acetyl (K)]DC[Carbamidomethyl (C)]AYC[Carbamidomethyl (C)]TDEMFR_ |
| P16219 | 306 | K | 1.673100972 | Up | Short-chain specific acyl-CoA dehydrogenase, mitochondrial OS=Homo sapiens OX=9606 GN=ACADS PE=1 SV=1 | ACADS | _MAFGAPLTK[Acetyl (K)]LQVIQFK_ |
| P16220 | 319 | K | 1.810633151 | Up | Cyclic AMP-responsive element-binding protein 1 OS=Homo sapiens OX=9606 GN=CREB1 PE=1 SV=3 | CREB1 | _ALK[Acetyl (K)]DLYC[Carbamidomethyl (C)]HK_ |
| P16278 | 376 | K | 2.154328388 | Up | Beta-galactosidase OS=Homo sapiens OX=9606 GN=GLB1 PE=1 SV=2 | GLB1 | _FAYGK[Acetyl (K)]VTLEK_ |
| P16435 | 176 | K | 0.520896451 | Down | NADPH--cytochrome P450 reductase OS=Homo sapiens OX=9606 GN=POR PE=1 SV=2 | POR | _FAVFGLGNK[Acetyl (K)]TYEHFNAMGK_ |
| P16615 | 533 | K | 3.551841008 | Up | Sarcoplasmic/endoplasmic reticulum calcium ATPase 2 OS=Homo sapiens OX=9606 GN=ATP2A2 PE=1 SV=1 | ATP2A2 | _VGSTK[Acetyl (K)]VPMTSGVK_ |
| P16949 | 52 | K | 0.414978845 | Down | Stathmin OS=Homo sapiens OX=9606 GN=STMN1 PE=1 SV=3 | STMN1 | _DLSLEEIQK[Acetyl (K)]K_ |
| P16949 | 100 | K | 2.770041808 | Up | Stathmin OS=Homo sapiens OX=9606 GN=STMN1 PE=1 SV=3 | STMN1 | _MAEEK[Acetyl (K)]LTHK_ |
| P16949 | 119 | K | 1.541292556 | Up | Stathmin OS=Homo sapiens OX=9606 GN=STMN1 PE=1 SV=3 | STMN1 | _EAQMAAK[Acetyl (K)]LER_ |
| P16989 | 90 | K | 1.502049345 | Up | Y-box-binding protein 3 OS=Homo sapiens OX=9606 GN=YBX3 PE=1 SV=4 | YBX3 | _VLATK[Acetyl (K)]VLGTVK_ |
| P17480 | 57 | K | 2.498785809 | Up | Nucleolar transcription factor 1 OS=Homo sapiens OX=9606 GN=UBTF PE=1 SV=1 | UBTF | _TTESHMDWEK[Acetyl (K)]VAFK_ |
| P17480 | 333 | K | 0.159432747 | Down | Nucleolar transcription factor 1 OS=Homo sapiens OX=9606 GN=UBTF PE=1 SV=1 | UBTF | _MVLC[Carbamidomethyl (C)]SQQWK[Acetyl (K)]LLSQK_ |
| P17844 | 284 | K | 0.515872969 | Down | Probable ATP-dependent RNA helicase DDX5 OS=Homo sapiens OX=9606 GN=DDX5 PE=1 SV=1 | DDX5 | _QTLM[Oxidation (M)]WSATWPK[Acetyl (K)]EVR_ |
| P17844 | 351 | K | 0.478550839 | Down | Probable ATP-dependent RNA helicase DDX5 OS=Homo sapiens OX=9606 GN=DDX5 PE=1 SV=1 | DDX5 | _TIVFVETK[Acetyl (K)]R_ |
| P17987 | 233 | K | 0.310760094 | Down | T-complex protein 1 subunit alpha OS=Homo sapiens OX=9606 GN=TCP1 PE=1 SV=1 | TCP1 | _IVNAK[Acetyl (K)]IAC[Carbamidomethyl (C)]LDFSLQK_ |
| P18077 | 8 | K | 0.512015487 | Down | Large ribosomal subunit protein eL33 OS=Homo sapiens OX=9606 GN=RPL35A PE=1 SV=2 | RPL35A | _LWSK[Acetyl (K)]AIFAGYK_ |
| P18077 | 73 | K | 1.769373721 | Up | Large ribosomal subunit protein eL33 OS=Homo sapiens OX=9606 GN=RPL35A PE=1 SV=2 | RPL35A | _VIWGK[Acetyl (K)]VTR_ |
| P18124 | 124 | K | 1.640805155 | Up | Large ribosomal subunit protein uL30 OS=Homo sapiens OX=9606 GN=RPL7 PE=1 SV=1 | RPL7 | _QIFNGTFVK[Acetyl (K)]LNK_ |
| P18669 | 113 | K | 0.543600387 | Down | Phosphoglycerate mutase 1 OS=Homo sapiens OX=9606 GN=PGAM1 PE=1 SV=2 | PGAM1 | _HGEAQVK[Acetyl (K)]IWR_ |
| P19338 | 116 | K | 0.161823443 | Down | Nucleolin OS=Homo sapiens OX=9606 GN=NCL PE=1 SV=3 | NCL | _GATPGK[Acetyl (K)]ALVATPGK_ |
| P19338 | 223 | K | 0.412232998 | Down | Nucleolin OS=Homo sapiens OX=9606 GN=NCL PE=1 SV=3 | NCL | _AAK[Acetyl (K)]VVPVK_ |
| P19338 | 398 | K | 1.508046344 | Up | Nucleolin OS=Homo sapiens OX=9606 GN=NCL PE=1 SV=3 | NCL | _TLLAK[Acetyl (K)]NLPYK_ |
| P19367 | 24 | K | 0.648615936 | Down | Hexokinase-1 OS=Homo sapiens OX=9606 GN=HK1 PE=1 SV=3 | HK1 | _KIDK[Acetyl (K)]YLYAMR_ |
| P19367 | 510 | K | 0.61580875 | Down | Hexokinase-1 OS=Homo sapiens OX=9606 GN=HK1 PE=1 SV=3 | HK1 | _QTHNNAVVK[Acetyl (K)]MLPSFVR_ |
| P19447 | 165 | K | 0.274741434 | Down | General transcription and DNA repair factor IIH helicase subunit XPB OS=Homo sapiens OX=9606 GN=ERCC3 PE=1 SV=1 | ERCC3 | _LC[Carbamidomethyl (C)]TVSYGK[Acetyl (K)]VK_ |
| P19838 | 251 | K | 1.553486479 | Up | Nuclear factor NF-kappa-B p105 subunit OS=Homo sapiens OX=9606 GN=NFKB1 PE=1 SV=2 | NFKB1 | _APNASNLK[Acetyl (K)]IVR_ |
| P20042 | 227 | K | 0.550989767 | Down | Eukaryotic translation initiation factor 2 subunit 2 OS=Homo sapiens OX=9606 GN=EIF2S2 PE=1 SV=2 | EIF2S2 | _TSFVNFTDIC[Carbamidomethyl (C)]K[Acetyl (K)]LLHR_ |
| P20042 | 324 | K | 0.442534619 | Down | Eukaryotic translation initiation factor 2 subunit 2 OS=Homo sapiens OX=9606 GN=EIF2S2 PE=1 SV=2 | EIF2S2 | _TGFQAVTGK[Acetyl (K)]R_ |
| P20290 | 57 | K | 1.716621742 | Up | Transcription factor BTF3 OS=Homo sapiens OX=9606 GN=BTF3 PE=1 SV=1 | BTF3 | _LAK[Acetyl (K)]LQAQVR_ |
| P20338 | 63 | K | 0.660800822 | Down | Ras-related protein Rab-4A OS=Homo sapiens OX=9606 GN=RAB4A PE=1 SV=3 | RAB4A | _YVK[Acetyl (K)]LQIWDTAGQER_ |
| P20339 | 22 | K | 0.432057113 | Down | Ras-related protein Rab-5A OS=Homo sapiens OX=9606 GN=RAB5A PE=1 SV=2 | RAB5A | _IC[Carbamidomethyl (C)]QFK[Acetyl (K)]LVLLGESAVGK_ |
| P20700 | 123 | K | 0.649474624 | Down | Lamin-B1 OS=Homo sapiens OX=9606 GN=LMNB1 PE=1 SV=2 | LMNB1 | _AEHDQLLLNYAK[Acetyl (K)]K_ |
| P20700 | 271 | K | 0.644514739 | Down | Lamin-B1 OS=Homo sapiens OX=9606 GN=LMNB1 PE=1 SV=2 | LMNB1 | _EELEQTYHAK[Acetyl (K)]LENAR_ |
| P21291 | 161 | K | 0.454331418 | Down | Cysteine and glycine-rich protein 1 OS=Homo sapiens OX=9606 GN=CSRP1 PE=1 SV=3 | CSRP1 | _GLESTTLADK[Acetyl (K)]DGEIYC[Carbamidomethyl (C)]K_ |
| P21291 | 173 | K | 1.602041501 | Up | Cysteine and glycine-rich protein 1 OS=Homo sapiens OX=9606 GN=CSRP1 PE=1 SV=3 | CSRP1 | _GC[Carbamidomethyl (C)]YAK[Acetyl (K)]NFGPK_ |
| P21333 | 1375 | K | 0.540344668 | Down | Filamin-A OS=Homo sapiens OX=9606 GN=FLNA PE=1 SV=4 | FLNA | _VHGPGIQSGTTNKPNK[Acetyl (K)]FTVETR_ |
| P21333 | 2621 | K | 0.655533914 | Down | Filamin-A OS=Homo sapiens OX=9606 GN=FLNA PE=1 SV=4 | FLNA | _LYSVSYLLK[Acetyl (K)]DK_ |
| P21397 | 379 | K | 1.528400473 | Up | Amine oxidase [flavin-containing] A OS=Homo sapiens OX=9606 GN=MAOA PE=1 SV=1 | MAOA | _IC[Carbamidomethyl (C)]ELYAK[Acetyl (K)]VLGSQEALHPVHYEEK_ |
| P21796 | 20 | K | 0.658578008 | Down | Voltage-dependent anion-selective channel protein 1 OS=Homo sapiens OX=9606 GN=VDAC1 PE=1 SV=2 | VDAC1 | _DVFTK[Acetyl (K)]GYGFGLIK_ |
| P21796 | 96 | K | 0.618097915 | Down | Voltage-dependent anion-selective channel protein 1 OS=Homo sapiens OX=9606 GN=VDAC1 PE=1 SV=2 | VDAC1 | _GLK[Acetyl (K)]LTFDSSFSPNTGK_ |
| P21796 | 274 | K | 0.622316077 | Down | Voltage-dependent anion-selective channel protein 1 OS=Homo sapiens OX=9606 GN=VDAC1 PE=1 SV=2 | VDAC1 | _NVNAGGHK[Acetyl (K)]LGLGLEFQA_ |
| P22102 | 20 | K | 0.448974546 | Down | Trifunctional purine biosynthetic protein adenosine-3 OS=Homo sapiens OX=9606 GN=GART PE=1 SV=1 | GART | _EHTLAWK[Acetyl (K)]LAQSHHVK_ |
| P22102 | 805 | K | 0.652546951 | Down | Trifunctional purine biosynthetic protein adenosine-3 OS=Homo sapiens OX=9606 GN=GART PE=1 SV=1 | GART | _NGSLTNHFSFEK[Acetyl (K)]K_ |
| P22102 | 866 | K | 2.165134235 | Up | Trifunctional purine biosynthetic protein adenosine-3 OS=Homo sapiens OX=9606 GN=GART PE=1 SV=1 | GART | _VINHK[Acetyl (K)]LYK_ |
| P22234 | 414 | K | 0.24559262 | Down | Bifunctional phosphoribosylaminoimidazole carboxylase/phosphoribosylaminoimidazole succinocarboxamide synthetase OS=Homo sapiens OX=9606 GN=PAICS PE=1 SV=3 | PAICS | _ASILNTWISLK[Acetyl (K)]QADK_ |
| P22314 | 185 | K | 0.298580891 | Down | Ubiquitin-like modifier-activating enzyme 1 OS=Homo sapiens OX=9606 GN=UBA1 PE=1 SV=3 | UBA1 | _GIK[Acetyl (K)]LVVADTR_ |
| P22570 | 400 | K | 0.221181758 | Down | NADPH:adrenodoxin oxidoreductase, mitochondrial OS=Homo sapiens OX=9606 GN=FDXR PE=1 SV=3 | FDXR | _VMDVPGLYC[Carbamidomethyl (C)]SGWVK[Acetyl (K)]R_ |
| P22695 | 98 | K | 0.384387078 | Down | Cytochrome b-c1 complex subunit 2, mitochondrial OS=Homo sapiens OX=9606 GN=UQCRC2 PE=1 SV=3 | UQCRC2 | _GASSFK[Acetyl (K)]ITR_ |
| P23396 | 75 | K | 0.51036397 | Down | Small ribosomal subunit protein uS3 OS=Homo sapiens OX=9606 GN=RPS3 PE=1 SV=2 | RPS3 | _ELTAVVQK[Acetyl (K)]R_ |
| P23396 | 108 | K | 0.375915692 | Down | Small ribosomal subunit protein uS3 OS=Homo sapiens OX=9606 GN=RPS3 PE=1 SV=2 | RPS3 | _YK[Acetyl (K)]LLGGLAVR_ |
| P23396 | 187 | K | 0.394235518 | Down | Small ribosomal subunit protein uS3 OS=Homo sapiens OX=9606 GN=RPS3 PE=1 SV=2 | RPS3 | _VK[Acetyl (K)]IMLPWDPTGK_ |
| P23396 | 197 | K | 0.314708809 | Down | Small ribosomal subunit protein uS3 OS=Homo sapiens OX=9606 GN=RPS3 PE=1 SV=2 | RPS3 | _IMLPWDPTGK[Acetyl (K)]IGPK_ |
| P23443 | 118 | K | 0.62030743 | Down | Ribosomal protein S6 kinase beta-1 OS=Homo sapiens OX=9606 GN=RPS6KB1 PE=1 SV=2 | RPS6KB1 | _VTGANTGK[Acetyl (K)]IFAMK_ |
| P23528 | 44 | K | 0.487816102 | Down | Cofilin-1 OS=Homo sapiens OX=9606 GN=CFL1 PE=1 SV=3 | CFL1 | _AVLFC[Carbamidomethyl (C)]LSEDK[Acetyl (K)]K_ |
| P23528 | 92 | K | 0.484737818 | Down | Cofilin-1 OS=Homo sapiens OX=9606 GN=CFL1 PE=1 SV=3 | CFL1 | _YALYDATYETK[Acetyl (K)]ESK_ |
| P23528 | 121 | K | 0.489715524 | Down | Cofilin-1 OS=Homo sapiens OX=9606 GN=CFL1 PE=1 SV=3 | CFL1 | _MIYASSK[Acetyl (K)]DAIK_ |
| P23528 | 144 | K | 0.65495202 | Down | Cofilin-1 OS=Homo sapiens OX=9606 GN=CFL1 PE=1 SV=3 | CFL1 | _HELQANC[Carbamidomethyl (C)]YEEVK[Acetyl (K)]DR_ |
| P23786 | 510 | K | 0.606615945 | Down | Carnitine O-palmitoyltransferase 2, mitochondrial OS=Homo sapiens OX=9606 GN=CPT2 PE=1 SV=2 | CPT2 | _PASVYTK[Acetyl (K)]R_ |
| P24534 | 185 | K | 0.556973374 | Down | Elongation factor 1-beta OS=Homo sapiens OX=9606 GN=EEF1B2 PE=1 SV=3 | EEF1B2 | _LVPVGYGIK[Acetyl (K)]K_ |
| P24752 | 124 | K | 0.595843925 | Down | Acetyl-CoA acetyltransferase, mitochondrial OS=Homo sapiens OX=9606 GN=ACAT1 PE=1 SV=1 | ACAT1 | _QAVLGAGLPISTPC[Carbamidomethyl (C)]TTINK[Acetyl (K)]VC[Carbamidomethyl (C)]ASGM[Oxidation (M)]K_ |
| P25205 | 413 | K | 0.31762067 | Down | DNA replication licensing factor MCM3 OS=Homo sapiens OX=9606 GN=MCM3 PE=1 SV=3 | MCM3 | _GVVC[Carbamidomethyl (C)]IDEFDK[Acetyl (K)]MSDMDR_ |
| P25398 | 112 | K | 0.603143443 | Down | Small ribosomal subunit protein eS12 OS=Homo sapiens OX=9606 GN=RPS12 PE=1 SV=3 | RPS12 | _VVGC[Carbamidomethyl (C)]SC[Carbamidomethyl (C)]VVVK[Acetyl (K)]DYGK_ |
| P25490 | 362 | K | 1.80959041 | Up | Transcriptional repressor protein YY1 OS=Homo sapiens OX=9606 GN=YY1 PE=1 SV=2 | YY1 | _PFQC[Carbamidomethyl (C)]TFEGC[Carbamidomethyl (C)]GK[Acetyl (K)]R_ |
| P25685 | 181 | K | 0.370861819 | Down | DnaJ homolog subfamily B member 1 OS=Homo sapiens OX=9606 GN=DNAJB1 PE=1 SV=4 | DNAJB1 | _VSLEEIYSGC[Carbamidomethyl (C)]TK[Acetyl (K)]K_ |
| P25705 | 161 | K | 0.272450316 | Down | ATP synthase subunit alpha, mitochondrial OS=Homo sapiens OX=9606 GN=ATP5F1A PE=1 SV=1 | ATP5F1A | _VVDALGNAIDGK[Acetyl (K)]GPIGSK_ |
| P25705 | 230 | K | 0.578628107 | Down | ATP synthase subunit alpha, mitochondrial OS=Homo sapiens OX=9606 GN=ATP5F1A PE=1 SV=1 | ATP5F1A | _TSIAIDTIINQK[Acetyl (K)]R_ |
| P25705 | 241 | K | 0.568118679 | Down | ATP synthase subunit alpha, mitochondrial OS=Homo sapiens OX=9606 GN=ATP5F1A PE=1 SV=1 | ATP5F1A | _K[Acetyl (K)]LYC[Carbamidomethyl (C)]IYVAIGQK_ |
| P25705 | 261 | K | 3.918356944 | Up | ATP synthase subunit alpha, mitochondrial OS=Homo sapiens OX=9606 GN=ATP5F1A PE=1 SV=1 | ATP5F1A | _STVAQLVK[Acetyl (K)]R_ |
| P25786 | 115 | K | 0.661268178 | Down | Proteasome subunit alpha type-1 OS=Homo sapiens OX=9606 GN=PSMA1 PE=1 SV=1 | PSMA1 | _LVSLIGSK[Acetyl (K)]TQIPTQR_ |
| P25788 | 43 | K | 1.557265703 | Up | Proteasome subunit alpha type-3 OS=Homo sapiens OX=9606 GN=PSMA3 PE=1 SV=2 | PSMA3 | _C[Carbamidomethyl (C)]K[Acetyl (K)]DGVVFGVEK_ |
| P25788 | 57 | K | 1.949462087 | Up | Proteasome subunit alpha type-3 OS=Homo sapiens OX=9606 GN=PSMA3 PE=1 SV=2 | PSMA3 | _LVLSK[Acetyl (K)]LYEEGSNK_ |
| P25788 | 179 | K | 1.545050936 | Up | Proteasome subunit alpha type-3 OS=Homo sapiens OX=9606 GN=PSMA3 PE=1 SV=2 | PSMA3 | _TEIEK[Acetyl (K)]LQMK_ |
| P25789 | 64 | K | 0.437585057 | Down | Proteasome subunit alpha type-4 OS=Homo sapiens OX=9606 GN=PSMA4 PE=1 SV=1 | PSMA4 | _LLDEVFFSEK[Acetyl (K)]IYK_ |
| P26006 | 638 | K | 0.333422754 | Down | Integrin alpha-3 OS=Homo sapiens OX=9606 GN=ITGA3 PE=1 SV=5 | ITGA3 | _AAFVSEQQQK[Acetyl (K)]LSR_ |
| P26038 | 60 | K | 0.376143728 | Down | Moesin OS=Homo sapiens OX=9606 GN=MSN PE=1 SV=3 | MSN | _GFSTWLK[Acetyl (K)]LNK_ |
| P26038 | 79 | K | 0.363625387 | Down | Moesin OS=Homo sapiens OX=9606 GN=MSN PE=1 SV=3 | MSN | _KESPLLFK[Acetyl (K)]FR_ |
| P26038 | 162 | K | 0.360130381 | Down | Moesin OS=Homo sapiens OX=9606 GN=MSN PE=1 SV=3 | MSN | _VLEQHK[Acetyl (K)]LNK_ |
| P26196 | 104 | K | 0.448456653 | Down | Probable ATP-dependent RNA helicase DDX6 OS=Homo sapiens OX=9606 GN=DDX6 PE=1 SV=2 | DDX6 | _GNEFEDYC[Carbamidomethyl (C)]LK[Acetyl (K)]R_ |
| P26358 | 675 | K | 1.859345302 | Up | DNA (cytosine-5)-methyltransferase 1 OS=Homo sapiens OX=9606 GN=DNMT1 PE=1 SV=2 | DNMT1 | _DMVK[Acetyl (K)]FGGSGR_ |
| P26447 | 57 | K | 0.599669027 | Down | Protein S100-A4 OS=Homo sapiens OX=9606 GN=S100A4 PE=1 SV=1 | S100A4 | _TDEAAFQK[Acetyl (K)]LM[Oxidation (M)]SNLDSNR_ |
| P26599 | 394 | K | 0.614803194 | Down | Polypyrimidine tract-binding protein 1 OS=Homo sapiens OX=9606 GN=PTBP1 PE=1 SV=2 | PTBP1 | _VK[Acetyl (K)]ILFNK_ |
| P26599 | 424 | K | 0.546156736 | Down | Polypyrimidine tract-binding protein 1 OS=Homo sapiens OX=9606 GN=PTBP1 PE=1 SV=2 | PTBP1 | _ENALVQMADGNQAQLAMSHLNGHK[Acetyl (K)]LHGK_ |
| P26599 | 508 | K | 0.558332835 | Down | Polypyrimidine tract-binding protein 1 OS=Homo sapiens OX=9606 GN=PTBP1 PE=1 SV=2 | PTBP1 | _VLFSSNGGVVK[Acetyl (K)]GFK_ |
| P26639 | 583 | K | 0.349518048 | Down | Threonine--tRNA ligase 1, cytoplasmic OS=Homo sapiens OX=9606 GN=TARS1 PE=1 SV=3 | TARS1 | _FNLTYVSHDGDDK[Acetyl (K)]K_ |
| P27635 | 74 | K | 0.410218684 | Down | Large ribosomal subunit protein uL16 OS=Homo sapiens OX=9606 GN=RPL10 PE=1 SV=5 | RPL10 | _IC[Carbamidomethyl (C)]ANK[Acetyl (K)]YMVK_ |
| P27635 | 188 | K | 0.44642156 | Down | Large ribosomal subunit protein uL16 OS=Homo sapiens OX=9606 GN=RPL10 PE=1 SV=5 | RPL10 | _FNADEFEDMVAEK[Acetyl (K)]R_ |
| P27635 | 198 | K | 0.362324247 | Down | Large ribosomal subunit protein uL16 OS=Homo sapiens OX=9606 GN=RPL10 PE=1 SV=5 | RPL10 | _LIPDGC[Carbamidomethyl (C)]GVK[Acetyl (K)]YIPSR_ |
| P27635 | 208 | K | 0.385906216 | Down | Large ribosomal subunit protein uL16 OS=Homo sapiens OX=9606 GN=RPL10 PE=1 SV=5 | RPL10 | _GPLDK[Acetyl (K)]WR_ |
| P27694 | 167 | K | 0.128069453 | Down | Replication protein A 70 kDa DNA-binding subunit OS=Homo sapiens OX=9606 GN=RPA1 PE=1 SV=2 | RPA1 | _TFGK[Acetyl (K)]AAGPSLSHTSGGTQSK_ |
| P27694 | 331 | K | 0.171487353 | Down | Replication protein A 70 kDa DNA-binding subunit OS=Homo sapiens OX=9606 GN=RPA1 PE=1 SV=2 | RPA1 | _SYEDATK[Acetyl (K)]ITVR_ |
| P27695 | 63 | K | 0.521454377 | Down | DNA-(apurinic or apyrimidinic site) endonuclease OS=Homo sapiens OX=9606 GN=APEX1 PE=1 SV=2 | APEX1 | _PATLK[Acetyl (K)]IC[Carbamidomethyl (C)]SWNVDGLR_ |
| P27708 | 1778 | K | 0.662200107 | Down | CAD protein OS=Homo sapiens OX=9606 GN=CAD PE=1 SV=3 | CAD | _AHWTPFEGQK[Acetyl (K)]VK_ |
| P27797 | 151 | K | 0.553550877 | Down | Calreticulin OS=Homo sapiens OX=9606 GN=CALR PE=1 SV=1 | CALR | _VHVIFNYK[Acetyl (K)]GK_ |
| P27797 | 159 | K | 0.448521274 | Down | Calreticulin OS=Homo sapiens OX=9606 GN=CALR PE=1 SV=1 | CALR | _NVLINK[Acetyl (K)]DIR_ |
| P27824 | 199 | K | 1.6788646 | Up | Calnexin OS=Homo sapiens OX=9606 GN=CANX PE=1 SV=2 | CANX | _C[Carbamidomethyl (C)]GEDYK[Acetyl (K)]LHFIFR_ |
| P27824 | 227 | K | 0.65728624 | Down | Calnexin OS=Homo sapiens OX=9606 GN=CANX PE=1 SV=2 | CANX | _RPDADLK[Acetyl (K)]TYFTDK_ |
| P27824 | 233 | K | 0.482501326 | Down | Calnexin OS=Homo sapiens OX=9606 GN=CANX PE=1 SV=2 | CANX | _TYFTDK[Acetyl (K)]K_ |
| P27824 | 458 | K | 0.51960051 | Down | Calnexin OS=Homo sapiens OX=9606 GN=CANX PE=1 SV=2 | CANX | _IVDDWANDGWGLK[Acetyl (K)]K_ |
| P28347 | 370 | K | 2.002980958 | Up | Transcriptional enhancer factor TEF-1 OS=Homo sapiens OX=9606 GN=TEAD1 PE=1 SV=2 | TEAD1 | _LK[Acetyl (K)]HLPEK_ |
| P28370 | 443 | K | 0.205776485 | Down | Probable global transcription activator SNF2L1 OS=Homo sapiens OX=9606 GN=SMARCA1 PE=1 SV=2 | SMARCA1 | _DIDVLNSSGK[Acetyl (K)]MDK_ |
| P28838 | 176 | K | 0.656117494 | Down | Cytosol aminopeptidase OS=Homo sapiens OX=9606 GN=LAP3 PE=1 SV=3 | LAP3 | _MAVSAK[Acetyl (K)]LYGSGDQEAWQK_ |
| P29372 | 220 | K | 0.602165823 | Down | DNA-3-methyladenine glycosylase OS=Homo sapiens OX=9606 GN=MPG PE=1 SV=3 | MPG | _ELC[Carbamidomethyl (C)]SGPSK[Acetyl (K)]LC[Carbamidomethyl (C)]QALAINK_ |
| P29373 | 99 | K | 0.503832326 | Down | Cellular retinoic acid-binding protein 2 OS=Homo sapiens OX=9606 GN=CRABP2 PE=1 SV=2 | CRABP2 | _MVC[Carbamidomethyl (C)]EQK[Acetyl (K)]LLK_ |
| P29401 | 603 | K | 0.433050907 | Down | Transketolase OS=Homo sapiens OX=9606 GN=TKT PE=1 SV=3 | TKT | _PAELLK[Acetyl (K)]MFGIDR_ |
| P29692 | 10 | K | 0.610457313 | Down | Elongation factor 1-delta OS=Homo sapiens OX=9606 GN=EEF1D PE=1 SV=5 | EEF1D | _[Acetyl (Protein N-term)]ATNFLAHEK[Acetyl (K)]IWFDK_ |
| P30040 | 48 | K | 0.364477446 | Down | Endoplasmic reticulum resident protein 29 OS=Homo sapiens OX=9606 GN=ERP29 PE=1 SV=4 | ERP29 | _GALPLDTVTFYK[Acetyl (K)]VIPK_ |
| P30040 | 112 | K | 0.64724341 | Down | Endoplasmic reticulum resident protein 29 OS=Homo sapiens OX=9606 GN=ERP29 PE=1 SV=4 | ERP29 | _LDK[Acetyl (K)]ESYPVFYLFR_ |
| P30041 | 144 | K | 3.204547368 | Up | Peroxiredoxin-6 OS=Homo sapiens OX=9606 GN=PRDX6 PE=1 SV=3 | PRDX6 | _LK[Acetyl (K)]LSILYPATTGR_ |
| P30050 | 40 | K | 0.611270981 | Down | Large ribosomal subunit protein uL11 OS=Homo sapiens OX=9606 GN=RPL12 PE=1 SV=1 | RPL12 | _IGPLGLSPK[Acetyl (K)]K_ |
| P30050 | 61 | K | 0.524022814 | Down | Large ribosomal subunit protein uL11 OS=Homo sapiens OX=9606 GN=RPL12 PE=1 SV=1 | RPL12 | _ITVK[Acetyl (K)]LTIQNR_ |
| P30101 | 226 | K | 0.666141451 | Down | Protein disulfide-isomerase A3 OS=Homo sapiens OX=9606 GN=PDIA3 PE=1 SV=4 | PDIA3 | _TVAYTEQK[Acetyl (K)]MTSGK_ |
| P30101 | 362 | K | 0.478822075 | Down | Protein disulfide-isomerase A3 OS=Homo sapiens OX=9606 GN=PDIA3 PE=1 SV=4 | PDIA3 | _FLQDYFDGNLK[Acetyl (K)]R_ |
| P31146 | 339 | K | 0.313367546 | Down | Coronin-1A OS=Homo sapiens OX=9606 GN=CORO1A PE=1 SV=4 | CORO1A | _FYK[Acetyl (K)]LHER_ |
| P31153 | 163 | K | 0.64952249 | Down | S-adenosylmethionine synthase isoform type-2 OS=Homo sapiens OX=9606 GN=MAT2A PE=1 SV=1 | MAT2A | _LNAK[Acetyl (K)]LAELR_ |
| P31930 | 134 | K | 0.665726975 | Down | Cytochrome b-c1 complex subunit 1, mitochondrial OS=Homo sapiens OX=9606 GN=UQCRC1 PE=1 SV=3 | UQCRC1 | _EHTAYYIK[Acetyl (K)]ALSK_ |
| P31943 | 35 | K | 0.499233376 | Down | Heterogeneous nuclear ribonucleoprotein H OS=Homo sapiens OX=9606 GN=HNRNPH1 PE=1 SV=4 | HNRNPH1 | _FFSDC[Carbamidomethyl (C)]K[Acetyl (K)]IQNGAQGIR_ |
| P31948 | 56 | K | 0.618202951 | Down | Stress-induced-phosphoprotein 1 OS=Homo sapiens OX=9606 GN=STIP1 PE=1 SV=1 | STIP1 | _KGDYQK[Acetyl (K)]AYEDGC[Carbamidomethyl (C)]K_ |
| P31948 | 388 | K | 2.794838994 | Up | Stress-induced-phosphoprotein 1 OS=Homo sapiens OX=9606 GN=STIP1 PE=1 SV=1 | STIP1 | _HYTEAIK[Acetyl (K)]R_ |
| P31948 | 462 | K | 0.499021198 | Down | Stress-induced-phosphoprotein 1 OS=Homo sapiens OX=9606 GN=STIP1 PE=1 SV=1 | STIP1 | _ALDLDSSC[Carbamidomethyl (C)]K[Acetyl (K)]EAADGYQR_ |
| P32119 | 196 | K | 0.192619816 | Down | Peroxiredoxin-2 OS=Homo sapiens OX=9606 GN=PRDX2 PE=1 SV=5 | PRDX2 | _EYFSK[Acetyl (K)]HN_ |
| P32969 | 121 | K | 0.518634434 | Down | Large ribosomal subunit protein uL6 OS=Homo sapiens OX=9606 GN=RPL9 PE=1 SV=1 | RPL9 | _NFLGEK[Acetyl (K)]YIR_ |
| P33121 | 419 | K | 2.466925749 | Up | Long-chain-fatty-acid--CoA ligase 1 OS=Homo sapiens OX=9606 GN=ACSL1 PE=1 SV=1 | ACSL1 | _LIFHK[Acetyl (K)]VQSSLGGR_ |
| P33176 | 690 | K | 0.574914503 | Down | Kinesin-1 heavy chain OS=Homo sapiens OX=9606 GN=KIF5B PE=1 SV=1 | KIF5B | _EHLNK[Acetyl (K)]VQTANEVK_ |
| P33992 | 54 | K | 0.657923001 | Down | DNA replication licensing factor MCM5 OS=Homo sapiens OX=9606 GN=MCM5 PE=1 SV=5 | MCM5 | _TGFTFK[Acetyl (K)]YR_ |
| P33992 | 256 | K | 0.496667847 | Down | DNA replication licensing factor MCM5 OS=Homo sapiens OX=9606 GN=MCM5 PE=1 SV=5 | MCM5 | _YLC[Carbamidomethyl (C)]DK[Acetyl (K)]VVPGNR_ |
| P33992 | 272 | K | 0.550228426 | Down | DNA replication licensing factor MCM5 OS=Homo sapiens OX=9606 GN=MCM5 PE=1 SV=5 | MCM5 | _VTIMGIYSIK[Acetyl (K)]K_ |
| P33993 | 159 | K | 1.578996874 | Up | DNA replication licensing factor MCM7 OS=Homo sapiens OX=9606 GN=MCM7 PE=1 SV=4 | MCM7 | _ADSVGK[Acetyl (K)]LVTVR_ |
| P34897 | 103 | K | 0.506538254 | Down | Serine hydroxymethyltransferase, mitochondrial OS=Homo sapiens OX=9606 GN=SHMT2 PE=1 SV=3 | SHMT2 | _YSEGYPGK[Acetyl (K)]R_ |
| P34897 | 461 | K | 1.906752107 | Up | Serine hydroxymethyltransferase, mitochondrial OS=Homo sapiens OX=9606 GN=SHMT2 PE=1 SV=3 | SHMT2 | _SK[Acetyl (K)]TAK[Acetyl (K)]LQDFK_ |
| P35221 | 120 | K | 0.511401095 | Down | Catenin alpha-1 OS=Homo sapiens OX=9606 GN=CTNNA1 PE=1 SV=1 | CTNNA1 | _AAAGEFADDPC[Carbamidomethyl (C)]SSVK[Acetyl (K)]R_ |
| P35221 | 493 | K | 2.445957248 | Up | Catenin alpha-1 OS=Homo sapiens OX=9606 GN=CTNNA1 PE=1 SV=1 | CTNNA1 | _EQWEK[Acetyl (K)]QVR_ |
| P35222 | 335 | K | 0.570754692 | Down | Catenin beta-1 OS=Homo sapiens OX=9606 GN=CTNNB1 PE=1 SV=1 | CTNNB1 | _TYTYEK[Acetyl (K)]LLWTTSR_ |
| P35232 | 4 | K | 0.425877818 | Down | Prohibitin 1 OS=Homo sapiens OX=9606 GN=PHB1 PE=1 SV=1 | PHB1 | _[Acetyl (Protein N-term)]AAK[Acetyl (K)]VFESIGK_ |
| P35241 | 60 | K | 0.360883456 | Down | Radixin OS=Homo sapiens OX=9606 GN=RDX PE=1 SV=1 | RDX | _GYSTWLK[Acetyl (K)]LNK_ |
| P35269 | 407 | K | 41.29475934 | Up | General transcription factor IIF subunit 1 OS=Homo sapiens OX=9606 GN=GTF2F1 PE=1 SV=2 | GTF2F1 | _AAASK[Acetyl (K)]LEQGK_ |
| P35270 | 240 | K | 1.533104183 | Up | Sepiapterin reductase OS=Homo sapiens OX=9606 GN=SPR PE=1 SV=1 | SPR | _VSAQK[Acetyl (K)]LLSLLEK_ |
| P35579 | 29 | K | 0.500325572 | Down | Myosin-9 OS=Homo sapiens OX=9606 GN=MYH9 PE=1 SV=4 | MYH9 | _NFINNPLAQADWAAK[Acetyl (K)]K_ |
| P35579 | 555 | K | 0.595577485 | Down | Myosin-9 OS=Homo sapiens OX=9606 GN=MYH9 PE=1 SV=4 | MYH9 | _VMQEQGTHPK[Acetyl (K)]FQKPK_ |
| P35579 | 1387 | K | 0.51097487 | Down | Myosin-9 OS=Homo sapiens OX=9606 GN=MYH9 PE=1 SV=4 | MYH9 | _MEDSVGC[Carbamidomethyl (C)]LETAEEVK[Acetyl (K)]R_ |
| P35637 | 365 | K | 0.65903574 | Down | RNA-binding protein FUS OS=Homo sapiens OX=9606 GN=FUS PE=1 SV=1 | FUS | _EFSGNPIK[Acetyl (K)]VSFATR_ |
| P35658 | 938 | K | 1.710521715 | Up | Nuclear pore complex protein Nup214 OS=Homo sapiens OX=9606 GN=NUP214 PE=1 SV=2 | NUP214 | _VPAK[Acetyl (K)]LSPMK_ |
| P35998 | 422 | K | 0.361515643 | Down | 26S proteasome regulatory subunit 7 OS=Homo sapiens OX=9606 GN=PSMC2 PE=1 SV=3 | PSMC2 | _SYAK[Acetyl (K)]FSATPR_ |
| P36578 | 283 | K | 0.295403707 | Down | Large ribosomal subunit protein uL4 OS=Homo sapiens OX=9606 GN=RPL4 PE=1 SV=5 | RPL4 | _SNYNLPMHK[Acetyl (K)]MINTDLSR_ |
| P36776 | 517 | K | 0.642541528 | Down | Lon protease homolog, mitochondrial OS=Homo sapiens OX=9606 GN=LONP1 PE=1 SV=2 | LONP1 | _GSTQGK[Acetyl (K)]ILC[Carbamidomethyl (C)]FYGPPGVGK_ |
| P36871 | 349 | K | 0.658650408 | Down | Phosphoglucomutase-1 OS=Homo sapiens OX=9606 GN=PGM1 PE=1 SV=3 | PGM1 | _VASATK[Acetyl (K)]IALYETPTGWK_ |
| P36873 | 141 | K | 0.638228589 | Down | Serine/threonine-protein phosphatase PP1-gamma catalytic subunit OS=Homo sapiens OX=9606 GN=PPP1CC PE=1 SV=1 | PPP1CC | _IYGFYDEC[Carbamidomethyl (C)]K[Acetyl (K)]R_ |
| P36915 | 301 | K | 0.579786753 | Down | Guanine nucleotide-binding protein-like 1 OS=Homo sapiens OX=9606 GN=GNL1 PE=1 SV=2 | GNL1 | _AC[Carbamidomethyl (C)]EAITVGK[Acetyl (K)]VDLSSWR_ |
| P37268 | 45 | K | 0.658311215 | Down | Squalene synthase OS=Homo sapiens OX=9606 GN=FDFT1 PE=1 SV=1 | FDFT1 | _TC[Carbamidomethyl (C)]YK[Acetyl (K)]YLNQTSR_ |
| P38159 | 9 | K | 0.621097071 | Down | RNA-binding motif protein, X chromosome OS=Homo sapiens OX=9606 GN=RBMX PE=1 SV=3 | RBMX | _PGK[Acetyl (K)]LFIGGLNTETNEK_ |
| P38159 | 30 | K | 1.603440905 | Up | RNA-binding motif protein, X chromosome OS=Homo sapiens OX=9606 GN=RBMX PE=1 SV=3 | RBMX | _ALEAVFGK[Acetyl (K)]YGR_ |
| P38646 | 121 | K | 0.466679414 | Down | Stress-70 protein, mitochondrial OS=Homo sapiens OX=9606 GN=HSPA9 PE=1 SV=2 | HSPA9 | _QAVTNPNNTFYATK[Acetyl (K)]R_ |
| P39019 | 23 | K | 2.453782974 | Up | Small ribosomal subunit protein eS19 OS=Homo sapiens OX=9606 GN=RPS19 PE=1 SV=2 | RPS19 | _ALAAFLK[Acetyl (K)]K_ |
| P39210 | 82 | K | 0.566167573 | Down | Protein Mpv17 OS=Homo sapiens OX=9606 GN=MPV17 PE=1 SV=1 | MPV17 | _FIPGTTK[Acetyl (K)]VDALK_ |
| P40227 | 55 | K | 0.535712558 | Down | T-complex protein 1 subunit zeta OS=Homo sapiens OX=9606 GN=CCT6A PE=1 SV=3 | CCT6A | _MLVSGAGDIK[Acetyl (K)]LTK_ |
| P40763 | 363 | K | 0.626847766 | Down | Signal transducer and activator of transcription 3 OS=Homo sapiens OX=9606 GN=STAT3 PE=1 SV=2 | STAT3 | _FPELNYQLK[Acetyl (K)]IK_ |
| P40925 | 170 | K | 0.559210388 | Down | Malate dehydrogenase, cytoplasmic OS=Homo sapiens OX=9606 GN=MDH1 PE=1 SV=4 | MDH1 | _AQIALK[Acetyl (K)]LGVTANDVK_ |
| P40939 | 60 | K | 2.137450509 | Up | Trifunctional enzyme subunit alpha, mitochondrial OS=Homo sapiens OX=9606 GN=HADHA PE=1 SV=2 | HADHA | _INSPNSK[Acetyl (K)]VNTLSK_ |
| P40939 | 540 | K | 0.428567558 | Down | Trifunctional enzyme subunit alpha, mitochondrial OS=Homo sapiens OX=9606 GN=HADHA PE=1 SV=2 | HADHA | _VIIVVK[Acetyl (K)]DGPGFYTTR_ |
| P41227 | 78 | K | 0.437354968 | Down | N-alpha-acetyltransferase 10 OS=Homo sapiens OX=9606 GN=NAA10 PE=1 SV=1 | NAA10 | _MEEDPDDVPHGHITSLAVK[Acetyl (K)]R_ |
| P41227 | 105 | K | 0.610170775 | Down | N-alpha-acetyltransferase 10 OS=Homo sapiens OX=9606 GN=NAA10 PE=1 SV=1 | NAA10 | _AMIENFNAK[Acetyl (K)]YVSLHVR_ |
| P41236 | 155 | K | 0.644452973 | Down | Protein phosphatase inhibitor 2 OS=Homo sapiens OX=9606 GN=PPP1R2 PE=1 SV=2 | PPP1R2 | _LHYNEGLNIK[Acetyl (K)]LAR_ |
| P41250 | 224 | K | 2.133543872 | Up | Glycine--tRNA ligase OS=Homo sapiens OX=9606 GN=GARS1 PE=1 SV=3 | GARS1 | _AHLQK[Acetyl (K)]LMSDK_ |
| P42166 | 667 | K | 2.25282713 | Up | Lamina-associated polypeptide 2, isoform alpha OS=Homo sapiens OX=9606 GN=TMPO PE=1 SV=2 | TMPO | _NK[Acetyl (K)]LASTPFK_ |
| P42224 | 359 | K | 2.103827398 | Up | Signal transducer and activator of transcription 1-alpha/beta OS=Homo sapiens OX=9606 GN=STAT1 PE=1 SV=2 | STAT1 | _LQELNYNLK[Acetyl (K)]VK_ |
| P42224 | 673 | K | 0.611291295 | Down | Signal transducer and activator of transcription 1-alpha/beta OS=Homo sapiens OX=9606 GN=STAT1 PE=1 SV=2 | STAT1 | _YLYPNIDK[Acetyl (K)]DHAFGK_ |
| P42285 | 78 | K | 1.631581664 | Up | Exosome RNA helicase MTR4 OS=Homo sapiens OX=9606 GN=MTREX PE=1 SV=3 | MTREX | _DVDFEGTDEPIFGK[Acetyl (K)]K_ |
| P42285 | 376 | K | 0.584915401 | Down | Exosome RNA helicase MTR4 OS=Homo sapiens OX=9606 GN=MTREX PE=1 SV=3 | MTREX | _GPSNVFK[Acetyl (K)]IVK_ |
| P42330 | 201 | K | 0.462345605 | Down | Aldo-keto reductase family 1 member C3 OS=Homo sapiens OX=9606 GN=AKR1C3 PE=1 SV=4 | AKR1C3 | _SK[Acetyl (K)]LLDFC[Carbamidomethyl (C)]K_ |
| P42704 | 107 | K | 0.64331891 | Down | Leucine-rich PPR motif-containing protein, mitochondrial OS=Homo sapiens OX=9606 GN=LRPPRC PE=1 SV=3 | LRPPRC | _LLQK[Acetyl (K)]VFNDTC[Carbamidomethyl (C)]R_ |
| P42704 | 395 | K | 4.970150748 | Up | Leucine-rich PPR motif-containing protein, mitochondrial OS=Homo sapiens OX=9606 GN=LRPPRC PE=1 SV=3 | LRPPRC | _LTDYC[Carbamidomethyl (C)]K[Acetyl (K)]K_ |
| P42704 | 1357 | K | 0.603374555 | Down | Leucine-rich PPR motif-containing protein, mitochondrial OS=Homo sapiens OX=9606 GN=LRPPRC PE=1 SV=3 | LRPPRC | _LDDLFLK[Acetyl (K)]R_ |
| P42766 | 43 | K | 8.002742246 | Up | Large ribosomal subunit protein uL29 OS=Homo sapiens OX=9606 GN=RPL35 PE=1 SV=2 | RPL35 | _VTGGAASK[Acetyl (K)]LSK_ |
| P43304 | 128 | K | 0.426424893 | Down | Glycerol-3-phosphate dehydrogenase, mitochondrial OS=Homo sapiens OX=9606 GN=GPD2 PE=1 SV=3 | GPD2 | _AIMK[Acetyl (K)]LDIEQYR_ |
| P43304 | 409 | K | 0.517222065 | Down | Glycerol-3-phosphate dehydrogenase, mitochondrial OS=Homo sapiens OX=9606 GN=GPD2 PE=1 SV=3 | GPD2 | _PLVTDPK[Acetyl (K)]SADTQSISR_ |
| P43487 | 76 | K | 2.095632337 | Up | Ran-specific GTPase-activating protein OS=Homo sapiens OX=9606 GN=RANBP1 PE=1 SV=1 | RANBP1 | _GTGDVK[Acetyl (K)]LLK_ |
| P43490 | 330 | K | 0.365301054 | Down | Nicotinamide phosphoribosyltransferase OS=Homo sapiens OX=9606 GN=NAMPT PE=1 SV=1 | NAMPT | _VLEILGK[Acetyl (K)]K_ |
| P43490 | 342 | K | 0.554486936 | Down | Nicotinamide phosphoribosyltransferase OS=Homo sapiens OX=9606 GN=NAMPT PE=1 SV=1 | NAMPT | _GYK[Acetyl (K)]LLPPYLR_ |
| P43686 | 212 | K | 0.466556647 | Down | 26S proteasome regulatory subunit 6B OS=Homo sapiens OX=9606 GN=PSMC4 PE=1 SV=2 | PSMC4 | _GVLMYGPPGC[Carbamidomethyl (C)]GK[Acetyl (K)]TMLAK_ |
| P45880 | 31 | K | 0.5748311 | Down | Voltage-dependent anion-selective channel protein 2 OS=Homo sapiens OX=9606 GN=VDAC2 PE=1 SV=2 | VDAC2 | _DIFNK[Acetyl (K)]GFGFGLVK_ |
| P45880 | 39 | K | 0.659816181 | Down | Voltage-dependent anion-selective channel protein 2 OS=Homo sapiens OX=9606 GN=VDAC2 PE=1 SV=2 | VDAC2 | _GFGFGLVK[Acetyl (K)]LDVK_ |
| P45974 | 585 | K | 5.128317688 | Up | Ubiquitin carboxyl-terminal hydrolase 5 OS=Homo sapiens OX=9606 GN=USP5 PE=1 SV=2 | USP5 | _FTFGLDWVPK[Acetyl (K)]K_ |
| P46013 | 1491 | K | 0.620959807 | Down | Proliferation marker protein Ki-67 OS=Homo sapiens OX=9606 GN=MKI67 PE=1 SV=2 | MKI67 | _TTK[Acetyl (K)]IAC[Carbamidomethyl (C)]R_ |
| P46013 | 1639 | K | 1.823624039 | Up | Proliferation marker protein Ki-67 OS=Homo sapiens OX=9606 GN=MKI67 PE=1 SV=2 | MKI67 | _TSLGK[Acetyl (K)]VGVK_ |
| P46013 | 2005 | K | 2.031747217 | Up | Proliferation marker protein Ki-67 OS=Homo sapiens OX=9606 GN=MKI67 PE=1 SV=2 | MKI67 | _ISLGK[Acetyl (K)]VGVK_ |
| P46013 | 2218 | K | 0.620959807 | Down | Proliferation marker protein Ki-67 OS=Homo sapiens OX=9606 GN=MKI67 PE=1 SV=2 | MKI67 | _TTK[Acetyl (K)]IAC[Carbamidomethyl (C)]R_ |
| P46013 | 2500 | K | 3.202037789 | Up | Proliferation marker protein Ki-67 OS=Homo sapiens OX=9606 GN=MKI67 PE=1 SV=2 | MKI67 | _EEPLAVSK[Acetyl (K)]LTR_ |
| P46087 | 76 | K | 1.621243476 | Up | Probable 28S rRNA (cytosine(4447)-C(5))-methyltransferase OS=Homo sapiens OX=9606 GN=NOP2 PE=1 SV=2 | NOP2 | _PLPGK[Acetyl (K)]LPK_ |
| P46087 | 649 | K | 0.600439171 | Down | Probable 28S rRNA (cytosine(4447)-C(5))-methyltransferase OS=Homo sapiens OX=9606 GN=NOP2 PE=1 SV=2 | NOP2 | _ASFQK[Acetyl (K)]LNGISK_ |
| P46199 | 597 | K | 2.052524922 | Up | Translation initiation factor IF-2, mitochondrial OS=Homo sapiens OX=9606 GN=MTIF2 PE=1 SV=2 | MTIF2 | _LHK[Acetyl (K)]IIYR_ |
| P46199 | 715 | K | 0.503160147 | Down | Translation initiation factor IF-2, mitochondrial OS=Homo sapiens OX=9606 GN=MTIF2 PE=1 SV=2 | MTIF2 | _IVC[Carbamidomethyl (C)]YEEK[Acetyl (K)]QIQAK_ |
| P46459 | 708 | K | 0.623913757 | Down | Vesicle-fusing ATPase OS=Homo sapiens OX=9606 GN=NSF PE=1 SV=3 | NSF | _VWIGIK[Acetyl (K)]K_ |
| P46776 | 110 | K | 0.619281938 | Down | Large ribosomal subunit protein uL15 OS=Homo sapiens OX=9606 GN=RPL27A PE=1 SV=2 | RPL27A | _SGYYK[Acetyl (K)]VLGK_ |
| P46777 | 48 | K | 0.54671432 | Down | Large ribosomal subunit protein uL18 OS=Homo sapiens OX=9606 GN=RPL5 PE=1 SV=3 | RPL5 | _YNTPK[Acetyl (K)]YR_ |
| P46777 | 241 | K | 0.475378479 | Down | Large ribosomal subunit protein uL18 OS=Homo sapiens OX=9606 GN=RPL5 PE=1 SV=3 | RPL5 | _NSVTPDMMEEMYK[Acetyl (K)]K_ |
| P46778 | 129 | K | 0.143932707 | Down | Large ribosomal subunit protein eL21 OS=Homo sapiens OX=9606 GN=RPL21 PE=1 SV=2 | RPL21 | _GTWVQLK[Acetyl (K)]R_ |
| P46779 | 19 | K | 0.077306515 | Down | Large ribosomal subunit protein eL28 OS=Homo sapiens OX=9606 GN=RPL28 PE=1 SV=3 | RPL28 | _NC[Carbamidomethyl (C)]SSFLIK[Acetyl (K)]R_ |
| P46781 | 52 | K | 0.113155631 | Down | Small ribosomal subunit protein uS4 OS=Homo sapiens OX=9606 GN=RPS9 PE=1 SV=3 | RPS9 | _FTLAK[Acetyl (K)]IR_ |
| P46783 | 59 | K | 2.910209641 | Up | Small ribosomal subunit protein eS10 OS=Homo sapiens OX=9606 GN=RPS10 PE=1 SV=1 | RPS10 | _GYVK[Acetyl (K)]EQFAWR_ |
| P46940 | 811 | K | 0.465775903 | Down | Ras GTPase-activating-like protein IQGAP1 OS=Homo sapiens OX=9606 GN=IQGAP1 PE=1 SV=1 | IQGAP1 | _SHKDEVVK[Acetyl (K)]IQSLAR_ |
| P46940 | 1505 | K | 0.610762748 | Down | Ras GTPase-activating-like protein IQGAP1 OS=Homo sapiens OX=9606 GN=IQGAP1 PE=1 SV=1 | IQGAP1 | _AELVK[Acetyl (K)]LQQTYAALNSK_ |
| P46940 | 1528 | K | 0.541779739 | Down | Ras GTPase-activating-like protein IQGAP1 OS=Homo sapiens OX=9606 GN=IQGAP1 PE=1 SV=1 | IQGAP1 | _ATFYGEQVDYYK[Acetyl (K)]SYIK_ |
| P47813 | 56 | K | 0.421318003 | Down | Eukaryotic translation initiation factor 1A, X-chromosomal OS=Homo sapiens OX=9606 GN=EIF1AX PE=1 SV=2 | EIF1AX | _LEAMC[Carbamidomethyl (C)]FDGVK[Acetyl (K)]R_ |
| P48059 | 290 | K | 0.462385036 | Down | LIM and senescent cell antigen-like-containing domain protein 1 OS=Homo sapiens OX=9606 GN=LIMS1 PE=1 SV=4 | LIMS1 | _NK[Acetyl (K)]FVEFDMKPVC[Carbamidomethyl (C)]K_ |
| P48200 | 877 | K | 2.082928384 | Up | Iron-responsive element-binding protein 2 OS=Homo sapiens OX=9606 GN=IREB2 PE=1 SV=4 | IREB2 | _AVLAESYEK[Acetyl (K)]IHK_ |
| P48382 | 564 | K | 0.641590187 | Down | DNA-binding protein RFX5 OS=Homo sapiens OX=9606 GN=RFX5 PE=1 SV=1 | RFX5 | _IPLVPSK[Acetyl (K)]VSVIK_ |
| P48444 | 38 | K | 0.56657391 | Down | Coatomer subunit delta OS=Homo sapiens OX=9606 GN=ARCN1 PE=1 SV=1 | ARCN1 | _IEGLLAAFPK[Acetyl (K)]LMNTGK_ |
| P48444 | 277 | K | 1.606224821 | Up | Coatomer subunit delta OS=Homo sapiens OX=9606 GN=ARCN1 PE=1 SV=1 | ARCN1 | _MHAPPINMESVHMK[Acetyl (K)]IEEK_ |
| P48507 | 94 | K | 0.615234701 | Down | Glutamate--cysteine ligase regulatory subunit OS=Homo sapiens OX=9606 GN=GCLM PE=1 SV=1 | GCLM | _VSAK[Acetyl (K)]LFIVESNSSSSTR_ |
| P48643 | 232 | K | 0.465570072 | Down | T-complex protein 1 subunit epsilon OS=Homo sapiens OX=9606 GN=CCT5 PE=1 SV=1 | CCT5 | _GVIVDK[Acetyl (K)]DFSHPQMPK_ |
| P48643 | 247 | K | 0.609641033 | Down | T-complex protein 1 subunit epsilon OS=Homo sapiens OX=9606 GN=CCT5 PE=1 SV=1 | CCT5 | _KVEDAK[Acetyl (K)]IAILTC[Carbamidomethyl (C)]PFEPPKPK_ |
| P48651 | 18 | K | 0.348630892 | Down | Phosphatidylserine synthase 1 OS=Homo sapiens OX=9606 GN=PTDSS1 PE=1 SV=1 | PTDSS1 | _DDVNYK[Acetyl (K)]MHFR_ |
| P48730 | 122 | K | 0.571791674 | Down | Casein kinase I isoform delta OS=Homo sapiens OX=9606 GN=CSNK1D PE=1 SV=2 | CSNK1D | _IEYIHSK[Acetyl (K)]NFIHR_ |
| P48735 | 67 | K | 0.573501012 | Down | Isocitrate dehydrogenase [NADP], mitochondrial OS=Homo sapiens OX=9606 GN=IDH2 PE=1 SV=2 | IDH2 | _IIWQFIK[Acetyl (K)]EK_ |
| P48735 | 193 | K | 0.577367542 | Down | Isocitrate dehydrogenase [NADP], mitochondrial OS=Homo sapiens OX=9606 GN=IDH2 PE=1 SV=2 | IDH2 | _AGTFK[Acetyl (K)]MVFTPK_ |
| P48735 | 282 | K | 0.490581167 | Down | Isocitrate dehydrogenase [NADP], mitochondrial OS=Homo sapiens OX=9606 GN=IDH2 PE=1 SV=2 | IDH2 | _NK[Acetyl (K)]IWYEHR_ |
| P48739 | 60 | K | 0.226471106 | Down | Phosphatidylinositol transfer protein beta isoform OS=Homo sapiens OX=9606 GN=PITPNB PE=1 SV=2 | PITPNB | _GQYTHK[Acetyl (K)]IYHLK_ |
| P49189 | 366 | K | 0.626680771 | Down | 4-trimethylaminobutyraldehyde dehydrogenase OS=Homo sapiens OX=9606 GN=ALDH9A1 PE=1 SV=3 | ALDH9A1 | _VLC[Carbamidomethyl (C)]GGDIYVPEDPK[Acetyl (K)]LK_ |
| P49327 | 41 | K | 0.269332441 | Down | Fatty acid synthase OS=Homo sapiens OX=9606 GN=FASN PE=1 SV=3 | FASN | _WK[Acetyl (K)]AGLYGLPR_ |
| P49327 | 1151 | K | 0.615864038 | Down | Fatty acid synthase OS=Homo sapiens OX=9606 GN=FASN PE=1 SV=3 | FASN | _GLVQALQTK[Acetyl (K)]VTQQGLK_ |
| P49327 | 1878 | K | 0.478552779 | Down | Fatty acid synthase OS=Homo sapiens OX=9606 GN=FASN PE=1 SV=3 | FASN | _LMSAISK[Acetyl (K)]TFC[Carbamidomethyl (C)]PAHK_ |
| P49327 | 1995 | K | 0.458066306 | Down | Fatty acid synthase OS=Homo sapiens OX=9606 GN=FASN PE=1 SV=3 | FASN | _PK[Acetyl (K)]YSGTLNLDR_ |
| P49327 | 2426 | K | 0.408170285 | Down | Fatty acid synthase OS=Homo sapiens OX=9606 GN=FASN PE=1 SV=3 | FASN | _SFYYK[Acetyl (K)]LR_ |
| P49368 | 370 | K | 0.602883553 | Down | T-complex protein 1 subunit gamma OS=Homo sapiens OX=9606 GN=CCT3 PE=1 SV=4 | CCT3 | _DPK[Acetyl (K)]AC[Carbamidomethyl (C)]TILLR_ |
| P49368 | 502 | K | 0.659680171 | Down | T-complex protein 1 subunit gamma OS=Homo sapiens OX=9606 GN=CCT3 PE=1 SV=4 | CCT3 | _ELGIWEPLAVK[Acetyl (K)]LQTYK_ |
| P49406 | 277 | K | 1.541426497 | Up | Large ribosomal subunit protein bL19m OS=Homo sapiens OX=9606 GN=MRPL19 PE=1 SV=2 | MRPL19 | _EYDTSK[Acetyl (K)]IEAAIWK_ |
| P49458 | 64 | K | 1.54547858 | Up | Signal recognition particle 9 kDa protein OS=Homo sapiens OX=9606 GN=SRP9 PE=1 SV=2 | SRP9 | _KIEK[Acetyl (K)]FHSQLMR_ |
| P49589 | 339 | K | 1.753723699 | Up | Cysteine--tRNA ligase, cytoplasmic OS=Homo sapiens OX=9606 GN=CARS1 PE=1 SV=3 | CARS1 | _PGEPSWPC[Carbamidomethyl (C)]PWGK[Acetyl (K)]GR_ |
| P49590 | 155 | K | 0.119451391 | Down | Histidine--tRNA ligase, mitochondrial OS=Homo sapiens OX=9606 GN=HARS2 PE=1 SV=1 | HARS2 | _YHVGK[Acetyl (K)]VWR_ |
| P49711 | 436 | K | 2.174051659 | Up | Transcriptional repressor CTCF OS=Homo sapiens OX=9606 GN=CTCF PE=1 SV=1 | CTCF | _HTENVAK[Acetyl (K)]FHC[Carbamidomethyl (C)]PHC[Carbamidomethyl (C)]DTVIAR_ |
| P49736 | 534 | K | 1.688984172 | Up | DNA replication licensing factor MCM2 OS=Homo sapiens OX=9606 GN=MCM2 PE=1 SV=4 | MCM2 | _SQFLK[Acetyl (K)]YIEK_ |
| P49790 | 294 | K | 0.332118943 | Down | Nuclear pore complex protein Nup153 OS=Homo sapiens OX=9606 GN=NUP153 PE=1 SV=2 | NUP153 | _AK[Acetyl (K)]QLSAQSYGVTSSTAR_ |
| P49790 | 685 | K | 0.514142241 | Down | Nuclear pore complex protein Nup153 OS=Homo sapiens OX=9606 GN=NUP153 PE=1 SV=2 | NUP153 | _C[Carbamidomethyl (C)]IAC[Carbamidomethyl (C)]QAAK[Acetyl (K)]LSPR_ |
| P49792 | 1220 | K | 3.39299644 | Up | E3 SUMO-protein ligase RanBP2 OS=Homo sapiens OX=9606 GN=RANBP2 PE=1 SV=2 | RANBP2 | _GIGNVK[Acetyl (K)]ILR_ |
| P49792 | 2366 | K | 1.762799105 | Up | E3 SUMO-protein ligase RanBP2 OS=Homo sapiens OX=9606 GN=RANBP2 PE=1 SV=2 | RANBP2 | _ILQNYDNK[Acetyl (K)]QVR_ |
| P49792 | 2966 | K | 0.290329124 | Down | E3 SUMO-protein ligase RanBP2 OS=Homo sapiens OX=9606 GN=RANBP2 PE=1 SV=2 | RANBP2 | _ILWHTMK[Acetyl (K)]NYYR_ |
| P49792 | 2980 | K | 0.477074678 | Down | E3 SUMO-protein ligase RanBP2 OS=Homo sapiens OX=9606 GN=RANBP2 PE=1 SV=2 | RANBP2 | _DQVFK[Acetyl (K)]VC[Carbamidomethyl (C)]ANHVITK_ |
| P49916 | 201 | K | 0.566920621 | Down | DNA ligase 3 OS=Homo sapiens OX=9606 GN=LIG3 PE=1 SV=2 | LIG3 | _AVVQAK[Acetyl (K)]LTTTGQVTSPVK_ |
| P49918 | 278 | K | 0.514044929 | Down | Cyclin-dependent kinase inhibitor 1C OS=Homo sapiens OX=9606 GN=CDKN1C PE=1 SV=1 | CDKN1C | _LSGPLISDFFAK[Acetyl (K)]R_ |
| P49959 | 384 | K | 0.600339573 | Down | Double-strand break repair protein MRE11 OS=Homo sapiens OX=9606 GN=MRE11 PE=1 SV=3 | MRE11 | _FSQK[Acetyl (K)]FVDR_ |
| P50213 | 77 | K | 0.524287102 | Down | Isocitrate dehydrogenase [NAD] subunit alpha, mitochondrial OS=Homo sapiens OX=9606 GN=IDH3A PE=1 SV=1 | IDH3A | _NVTAIQGPGGK[Acetyl (K)]WMIPSEAK_ |
| P50395 | 435 | K | 0.553020454 | Down | Rab GDP dissociation inhibitor beta OS=Homo sapiens OX=9606 GN=GDI2 PE=1 SV=2 | GDI2 | _MTGSEFDFEEMK[Acetyl (K)]R_ |
| P50416 | 40 | K | 1.548490347 | Up | Carnitine O-palmitoyltransferase 1, liver isoform OS=Homo sapiens OX=9606 GN=CPT1A PE=1 SV=2 | CPT1A | _QIYLSGLHSWK[Acetyl (K)]K_ |
| P50416 | 344 | K | 1.658675873 | Up | Carnitine O-palmitoyltransferase 1, liver isoform OS=Homo sapiens OX=9606 GN=CPT1A PE=1 SV=2 | CPT1A | _YFK[Acetyl (K)]VWLYHDGR_ |
| P50454 | 283 | K | 1.755570704 | Up | Serpin H1 OS=Homo sapiens OX=9606 GN=SERPINH1 PE=1 SV=2 | SERPINH1 | _LEK[Acetyl (K)]LLTK_ |
| P50502 | 153 | K | 0.610479032 | Down | Hsc70-interacting protein OS=Homo sapiens OX=9606 GN=ST13 PE=1 SV=2 | ST13 | _LAILYAK[Acetyl (K)]R_ |
| P50552 | 21 | K | 0.373899672 | Down | Vasodilator-stimulated phosphoprotein OS=Homo sapiens OX=9606 GN=VASP PE=1 SV=3 | VASP | _ATVMLYDDGNK[Acetyl (K)]R_ |
| P50579 | 385 | K | 2.252007691 | Up | Methionine aminopeptidase 2 OS=Homo sapiens OX=9606 GN=METAP2 PE=1 SV=1 | METAP2 | _GVVHDDMEC[Carbamidomethyl (C)]SHYMK[Acetyl (K)]NFDVGHVPIR_ |
| P50748 | 521 | K | 1.566196425 | Up | Kinetochore-associated protein 1 OS=Homo sapiens OX=9606 GN=KNTC1 PE=1 SV=1 | KNTC1 | _AHAK[Acetyl (K)]LTTFYGAFGPEK_ |
| P50914 | 177 | K | 2.977812794 | Up | Large ribosomal subunit protein eL14 OS=Homo sapiens OX=9606 GN=RPL14 PE=1 SV=4 | RPL14 | _APAQK[Acetyl (K)]VPAQK[Acetyl (K)]ATGQK_ |
| P50914 | 182 | K | 2.977812794 | Up | Large ribosomal subunit protein eL14 OS=Homo sapiens OX=9606 GN=RPL14 PE=1 SV=4 | RPL14 | _APAQK[Acetyl (K)]VPAQK[Acetyl (K)]ATGQK_ |
| P50990 | 307 | K | 0.581005963 | Down | T-complex protein 1 subunit theta OS=Homo sapiens OX=9606 GN=CCT8 PE=1 SV=4 | CCT8 | _VADMALHYANK[Acetyl (K)]YNIMLVR_ |
| P50991 | 321 | K | 0.572212229 | Down | T-complex protein 1 subunit delta OS=Homo sapiens OX=9606 GN=CCT4 PE=1 SV=4 | CCT4 | _MK[Acetyl (K)]IM[Oxidation (M)]VIK_ |
| P51003 | 650 | K | 0.439131342 | Down | Poly(A) polymerase alpha OS=Homo sapiens OX=9606 GN=PAPOLA PE=1 SV=4 | PAPOLA | _IPTPIVGVK[Acetyl (K)]R_ |
| P51114 | 295 | K | 1.753509532 | Up | RNA-binding protein FXR1 OS=Homo sapiens OX=9606 GN=FXR1 PE=1 SV=3 | FXR1 | _NLVGK[Acetyl (K)]VIGK_ |
| P51398 | 175 | K | 8.91653583 | Up | Small ribosomal subunit protein mS29 OS=Homo sapiens OX=9606 GN=DAP3 PE=1 SV=1 | DAP3 | _DLLQSSYNK[Acetyl (K)]QR_ |
| P51398 | 207 | K | 0.622027416 | Down | Small ribosomal subunit protein mS29 OS=Homo sapiens OX=9606 GN=DAP3 PE=1 SV=1 | DAP3 | _VQEK[Acetyl (K)]YVWNK_ |
| P51452 | 53 | K | 1.548826918 | Up | Dual specificity protein phosphatase 3 OS=Homo sapiens OX=9606 GN=DUSP3 PE=1 SV=1 | DUSP3 | _LQK[Acetyl (K)]LGITHVLNAAEGR_ |
| P51532 | 892 | K | 3.427500162 | Up | Transcription activator BRG1 OS=Homo sapiens OX=9606 GN=SMARCA4 PE=1 SV=2 | SMARCA4 | _NHHC[Carbamidomethyl (C)]K[Acetyl (K)]LTQVLNTHYVAPR_ |
| P51532 | 1450 | K | 5.9752168 | Up | Transcription activator BRG1 OS=Homo sapiens OX=9606 GN=SMARCA4 PE=1 SV=2 | SMARCA4 | _GRPPAEK[Acetyl (K)]LSPNPPNLTK_ |
| P51608 | 219 | K | 2.833183027 | Up | Methyl-CpG-binding protein 2 OS=Homo sapiens OX=9606 GN=MECP2 PE=1 SV=1 | MECP2 | _SPGK[Acetyl (K)]LLVK_ |
| P51608 | 249 | K | 2.246858023 | Up | Methyl-CpG-binding protein 2 OS=Homo sapiens OX=9606 GN=MECP2 PE=1 SV=1 | MECP2 | _AEGGGATTSTQVMVIK[Acetyl (K)]R_ |
| P51610 | 813 | K | 0.647379714 | Down | Host cell factor 1 OS=Homo sapiens OX=9606 GN=HCFC1 PE=1 SV=2 | HCFC1 | _VM[Oxidation (M)]TSGTGAPAK[Acetyl (K)]IITAVPK_ |
| P51688 | 425 | K | 0.658038795 | Down | N-sulphoglucosamine sulphohydrolase OS=Homo sapiens OX=9606 GN=SGSH PE=1 SV=1 | SGSH | _TTAGQPTGWYK[Acetyl (K)]DLR_ |
| P51790 | 797 | K | 0.550968363 | Down | H(+)/Cl(-) exchange transporter 3 OS=Homo sapiens OX=9606 GN=CLCN3 PE=1 SV=2 | CLCN3 | _LLGIITK[Acetyl (K)]K_ |
| P51991 | 126 | K | 0.553668185 | Down | Heterogeneous nuclear ribonucleoprotein A3 OS=Homo sapiens OX=9606 GN=HNRNPA3 PE=1 SV=2 | HNRNPA3 | _EDSVKPGAHLTVK[Acetyl (K)]K_ |
| P51991 | 134 | K | 0.474310547 | Down | Heterogeneous nuclear ribonucleoprotein A3 OS=Homo sapiens OX=9606 GN=HNRNPA3 PE=1 SV=2 | HNRNPA3 | _IFVGGIK[Acetyl (K)]EDTEEYNLR_ |
| P51991 | 187 | K | 0.248474872 | Down | Heterogeneous nuclear ribonucleoprotein A3 OS=Homo sapiens OX=9606 GN=HNRNPA3 PE=1 SV=2 | HNRNPA3 | _IVVQK[Acetyl (K)]YHTINGHNC[Carbamidomethyl (C)]EVK_ |
| P52272 | 69 | K | 0.326545729 | Down | Heterogeneous nuclear ribonucleoprotein M OS=Homo sapiens OX=9606 GN=HNRNPM PE=1 SV=3 | HNRNPM | _FEPYANPTK[Acetyl (K)]R_ |
| P52272 | 83 | K | 0.237698431 | Down | Heterogeneous nuclear ribonucleoprotein M OS=Homo sapiens OX=9606 GN=HNRNPM PE=1 SV=3 | HNRNPM | _AFITNIPFDVK[Acetyl (K)]WQSLK_ |
| P52272 | 88 | K | 0.500313437 | Down | Heterogeneous nuclear ribonucleoprotein M OS=Homo sapiens OX=9606 GN=HNRNPM PE=1 SV=3 | HNRNPM | _WQSLK[Acetyl (K)]DLVK_ |
| P52272 | 667 | K | 0.259735874 | Down | Heterogeneous nuclear ribonucleoprotein M OS=Homo sapiens OX=9606 GN=HNRNPM PE=1 SV=3 | HNRNPM | _NLPFDFTWK[Acetyl (K)]MLK_ |
| P52594 | 313 | K | 0.459749405 | Down | Arf-GAP domain and FG repeat-containing protein 1 OS=Homo sapiens OX=9606 GN=AGFG1 PE=1 SV=2 | AGFG1 | _SSSADFGTFNTSQSHQTASAVSK[Acetyl (K)]VSTNK_ |
| P52655 | 49 | K | 0.607491898 | Down | Transcription initiation factor IIA subunit 1 OS=Homo sapiens OX=9606 GN=GTF2A1 PE=1 SV=1 | GTF2A1 | _TLWENK[Acetyl (K)]LMQSR_ |
| P52907 | 278 | K | 0.490958741 | Down | F-actin-capping protein subunit alpha-1 OS=Homo sapiens OX=9606 GN=CAPZA1 PE=1 SV=3 | CAPZA1 | _ILSYK[Acetyl (K)]IGK_ |
| P52948 | 551 | K | 0.243540905 | Down | Nuclear pore complex protein Nup98-Nup96 OS=Homo sapiens OX=9606 GN=NUP98 PE=1 SV=4 | NUP98 | _ALTTPTHYK[Acetyl (K)]LTPR_ |
| P53396 | 498 | K | 0.290880214 | Down | ATP-citrate synthase OS=Homo sapiens OX=9606 GN=ACLY PE=1 SV=3 | ACLY | _HTK[Acetyl (K)]AIVWGMQTR_ |
| P53396 | 991 | K | 0.550113179 | Down | ATP-citrate synthase OS=Homo sapiens OX=9606 GN=ACLY PE=1 SV=3 | ACLY | _VQILK[Acetyl (K)]DYVR_ |
| P53582 | 298 | K | 1.771784779 | Up | Methionine aminopeptidase 1 OS=Homo sapiens OX=9606 GN=METAP1 PE=1 SV=2 | METAP1 | _SYC[Carbamidomethyl (C)]GHGIHK[Acetyl (K)]LFHTAPNVPHYAK_ |
| P53597 | 81 | K | 0.511627019 | Down | Succinate--CoA ligase [ADP/GDP-forming] subunit alpha, mitochondrial OS=Homo sapiens OX=9606 GN=SUCLG1 PE=1 SV=4 | SUCLG1 | _QGTFHSQQALEYGTK[Acetyl (K)]LVGGTTPGK_ |
| P53611 | 124 | K | 1.628159285 | Up | Geranylgeranyl transferase type-2 subunit beta OS=Homo sapiens OX=9606 GN=RABGGTB PE=1 SV=2 | RABGGTB | _VVEYVK[Acetyl (K)]GLQK_ |
| P53680 | 56 | K | 2.289379105 | Up | AP-2 complex subunit sigma OS=Homo sapiens OX=9606 GN=AP2S1 PE=1 SV=2 | AP2S1 | _NFK[Acetyl (K)]IIYR_ |
| P53990 | 134 | K | 0.119972635 | Down | IST1 homolog OS=Homo sapiens OX=9606 GN=IST1 PE=1 SV=1 | IST1 | _EYGK[Acetyl (K)]LC[Carbamidomethyl (C)]R_ |
| P54274 | 213 | K | 1.555117552 | Up | Telomeric repeat-binding factor 1 OS=Homo sapiens OX=9606 GN=TERF1 PE=1 SV=3 | TERF1 | _SK[Acetyl (K)]LLMIISQK_ |
| P54578 | 441 | K | 0.539471346 | Down | Ubiquitin carboxyl-terminal hydrolase 14 OS=Homo sapiens OX=9606 GN=USP14 PE=1 SV=3 | USP14 | _SSSSGHYVSWVK[Acetyl (K)]R_ |
| P55010 | 248 | K | 0.639132596 | Down | Eukaryotic translation initiation factor 5 OS=Homo sapiens OX=9606 GN=EIF5 PE=1 SV=2 | EIF5 | _VNILFDFVK[Acetyl (K)]K_ |
| P55081 | 357 | K | 0.648753982 | Down | Microfibrillar-associated protein 1 OS=Homo sapiens OX=9606 GN=MFAP1 PE=1 SV=2 | MFAP1 | _GAFFMDEDEEVYK[Acetyl (K)]R_ |
| P55084 | 201 | K | 0.175281656 | Down | Trifunctional enzyme subunit beta, mitochondrial OS=Homo sapiens OX=9606 GN=HADHB PE=1 SV=3 | HADHB | _LSLISK[Acetyl (K)]FR_ |
| P55199 | 355 | K | 0.410510819 | Down | RNA polymerase II elongation factor ELL OS=Homo sapiens OX=9606 GN=ELL PE=1 SV=1 | ELL | _AQPAVNGK[Acetyl (K)]LGVPNGR_ |
| P55201 | 896 | K | 0.618548219 | Down | Peregrin OS=Homo sapiens OX=9606 GN=BRPF1 PE=1 SV=2 | BRPF1 | _TSVLFSK[Acetyl (K)]K_ |
| P55209 | 116 | K | 0.660452504 | Down | Nucleosome assembly protein 1-like 1 OS=Homo sapiens OX=9606 GN=NAP1L1 PE=1 SV=1 | NAP1L1 | _YAVLYQPLFDK[Acetyl (K)]R_ |
| P55210 | 66 | K | 0.522972391 | Down | Caspase-7 OS=Homo sapiens OX=9606 GN=CASP7 PE=1 SV=1 | CASP7 | _DRVPTYQYNMNFEK[Acetyl (K)]LGK_ |
| P55854 | 41 | K | 1.75598629 | Up | Small ubiquitin-related modifier 3 OS=Homo sapiens OX=9606 GN=SUMO3 PE=1 SV=2 | SUMO3 | _HTPLSK[Acetyl (K)]LM[Oxidation (M)]K_ |
| P56385 | 34 | K | 2.550628737 | Up | ATP synthase subunit e, mitochondrial OS=Homo sapiens OX=9606 GN=ATP5ME PE=1 SV=2 | ATP5ME | _YNYLK[Acetyl (K)]PR_ |
| P56589 | 189 | K | 3.089593348 | Up | Peroxisomal biogenesis factor 3 OS=Homo sapiens OX=9606 GN=PEX3 PE=1 SV=1 | PEX3 | _QAVQK[Acetyl (K)]VLGSVSLK_ |
| P58107 | 2242 | K | 0.574511324 | Down | Epiplakin OS=Homo sapiens OX=9606 GN=EPPK1 PE=1 SV=3 | EPPK1 | _QEK[Acetyl (K)]MSIYQAMWK_ |
| P58107 | 2778 | K | 0.574511324 | Down | Epiplakin OS=Homo sapiens OX=9606 GN=EPPK1 PE=1 SV=3 | EPPK1 | _QEK[Acetyl (K)]MSIYQAMWK_ |
| P58107 | 3309 | K | 0.574511324 | Down | Epiplakin OS=Homo sapiens OX=9606 GN=EPPK1 PE=1 SV=3 | EPPK1 | _QEK[Acetyl (K)]MSIYQAMWK_ |
| P58107 | 3843 | K | 0.574511324 | Down | Epiplakin OS=Homo sapiens OX=9606 GN=EPPK1 PE=1 SV=3 | EPPK1 | _QEK[Acetyl (K)]MSIYQAMWK_ |
| P58107 | 4377 | K | 0.574511324 | Down | Epiplakin OS=Homo sapiens OX=9606 GN=EPPK1 PE=1 SV=3 | EPPK1 | _QEK[Acetyl (K)]MSIYQAMWK_ |
| P58107 | 4910 | K | 0.574511324 | Down | Epiplakin OS=Homo sapiens OX=9606 GN=EPPK1 PE=1 SV=3 | EPPK1 | _QEK[Acetyl (K)]MSIYQAMWK_ |
| P60033 | 193 | K | 2.023207485 | Up | CD81 antigen OS=Homo sapiens OX=9606 GN=CD81 PE=1 SV=1 | CD81 | _EDC[Carbamidomethyl (C)]HQK[Acetyl (K)]IDDLFSGK_ |
| P60174 | 6 | K | 0.431608764 | Down | Triosephosphate isomerase OS=Homo sapiens OX=9606 GN=TPI1 PE=1 SV=4 | TPI1 | _K[Acetyl (K)]FFVGGNWK_ |
| P60174 | 188 | K | 0.283298695 | Down | Triosephosphate isomerase OS=Homo sapiens OX=9606 GN=TPI1 PE=1 SV=4 | TPI1 | _TATPQQAQEVHEK[Acetyl (K)]LR_ |
| P60228 | 265 | K | 0.630521332 | Down | Eukaryotic translation initiation factor 3 subunit E OS=Homo sapiens OX=9606 GN=EIF3E PE=1 SV=1 | EIF3E | _YLTTAVITNK[Acetyl (K)]DVR_ |
| P60660 | 50 | K | 0.634695509 | Down | Myosin light polypeptide 6 OS=Homo sapiens OX=9606 GN=MYL6 PE=1 SV=2 | MYL6 | _ALGQNPTNAEVLK[Acetyl (K)]VLGNPK_ |
| P60709 | 326 | K | 1.570861091 | Up | Actin, cytoplasmic 1 OS=Homo sapiens OX=9606 GN=ACTB PE=1 SV=1 | ACTB | _EITALAPSTMK[Acetyl (K)]IK_ |
| P60842 | 54 | K | 0.504548338 | Down | Eukaryotic initiation factor 4A-I OS=Homo sapiens OX=9606 GN=EIF4A1 PE=1 SV=1 | EIF4A1 | _GIYAYGFEK[Acetyl (K)]PSAIQQR_ |
| P60981 | 19 | K | 0.63675948 | Down | Destrin OS=Homo sapiens OX=9606 GN=DSTN PE=1 SV=3 | DSTN | _IFYDMK[Acetyl (K)]VR_ |
| P60981 | 92 | K | 0.373818287 | Down | Destrin OS=Homo sapiens OX=9606 GN=DSTN PE=1 SV=3 | DSTN | _YALYDASFETK[Acetyl (K)]ESR_ |
| P60983 | 137 | K | 1.565227996 | Up | Glia maturation factor beta OS=Homo sapiens OX=9606 GN=GMFB PE=1 SV=2 | GMFB | _EK[Acetyl (K)]LGFFH_ |
| P61011 | 120 | K | 0.583328155 | Down | Signal recognition particle subunit SRP54 OS=Homo sapiens OX=9606 GN=SRP54 PE=1 SV=1 | SRP54 | _TTTC[Carbamidomethyl (C)]SK[Acetyl (K)]LAYYYQR_ |
| P61158 | 317 | K | 0.57605975 | Down | Actin-related protein 3 OS=Homo sapiens OX=9606 GN=ACTR3 PE=1 SV=3 | ACTR3 | _RPLYK[Acetyl (K)]NIVLSGGSTMFR_ |
| P61160 | 368 | K | 0.599879786 | Down | Actin-related protein 2 OS=Homo sapiens OX=9606 GN=ACTR2 PE=1 SV=1 | ACTR2 | _DK[Acetyl (K)]DNFWMTR_ |
| P61201 | 77 | K | 0.382875779 | Down | COP9 signalosome complex subunit 2 OS=Homo sapiens OX=9606 GN=COPS2 PE=1 SV=1 | COPS2 | _QMIK[Acetyl (K)]INFK_ |
| P61201 | 243 | K | 0.554487317 | Down | COP9 signalosome complex subunit 2 OS=Homo sapiens OX=9606 GN=COPS2 PE=1 SV=1 | COPS2 | _EC[Carbamidomethyl (C)]GGK[Acetyl (K)]MHLR_ |
| P61247 | 115 | K | 2.555214182 | Up | Small ribosomal subunit protein eS1 OS=Homo sapiens OX=9606 GN=RPS3A PE=1 SV=2 | RPS3A | _MC[Carbamidomethyl (C)]SMVK[Acetyl (K)]K_ |
| P61247 | 227 | K | 0.572861594 | Down | Small ribosomal subunit protein eS1 OS=Homo sapiens OX=9606 GN=RPS3A PE=1 SV=2 | RPS3A | _FELGK[Acetyl (K)]LMELHGEGSSSGK_ |
| P61254 | 2 | K | 0.58463115 | Down | Large ribosomal subunit protein uL24 OS=Homo sapiens OX=9606 GN=RPL26 PE=1 SV=1 | RPL26 | _MK[Acetyl (K)]FNPFVTSDR_ |
| P61289 | 14 | K | 4.968212807 | Up | Proteasome activator complex subunit 3 OS=Homo sapiens OX=9606 GN=PSME3 PE=1 SV=1 | PSME3 | _LK[Acetyl (K)]VDSFR_ |
| P61353 | 3 | K | 2.061198131 | Up | Large ribosomal subunit protein eL27 OS=Homo sapiens OX=9606 GN=RPL27 PE=1 SV=2 | RPL27 | _[Acetyl (Protein N-term)]GK[Acetyl (K)]FMKPGK_ |
| P61353 | 9 | K | 1.767303143 | Up | Large ribosomal subunit protein eL27 OS=Homo sapiens OX=9606 GN=RPL27 PE=1 SV=2 | RPL27 | _PGK[Acetyl (K)]VVLVLAGR_ |
| P61513 | 36 | K | 1.730413809 | Up | Large ribosomal subunit protein eL43 OS=Homo sapiens OX=9606 GN=RPL37A PE=1 SV=2 | RPL37A | _IEISQHAK[Acetyl (K)]YTC[Carbamidomethyl (C)]SFC[Carbamidomethyl (C)]GK_ |
| P61586 | 118 | K | 0.436409127 | Down | Transforming protein RhoA OS=Homo sapiens OX=9606 GN=RHOA PE=1 SV=1 | RHOA | _HFC[Carbamidomethyl (C)]PNVPIILVGNK[Acetyl (K)]K_ |
| P61586 | 162 | K | 0.4374909 | Down | Transforming protein RhoA OS=Homo sapiens OX=9606 GN=RHOA PE=1 SV=1 | RHOA | _IGAFGYMEC[Carbamidomethyl (C)]SAK[Acetyl (K)]TK_ |
| P61764 | 339 | K | 0.305346154 | Down | Syntaxin-binding protein 1 OS=Homo sapiens OX=9606 GN=STXBP1 PE=1 SV=1 | STXBP1 | _MPQYQK[Acetyl (K)]ELSK_ |
| P61923 | 51 | K | 0.605841338 | Down | Coatomer subunit zeta-1 OS=Homo sapiens OX=9606 GN=COPZ1 PE=1 SV=1 | COPZ1 | _NIFNK[Acetyl (K)]THR_ |
| P61956 | 42 | K | 4.033202531 | Up | Small ubiquitin-related modifier 2 OS=Homo sapiens OX=9606 GN=SUMO2 PE=1 SV=3 | SUMO2 | _HTPLSK[Acetyl (K)]LM[Oxidation (M)]K_ |
| P61956 | 45 | K | 2.143429448 | Up | Small ubiquitin-related modifier 2 OS=Homo sapiens OX=9606 GN=SUMO2 PE=1 SV=3 | SUMO2 | _LMK[Acetyl (K)]AYC[Carbamidomethyl (C)]ER_ |
| P62081 | 74 | K | 12.62555958 | Up | Small ribosomal subunit protein eS7 OS=Homo sapiens OX=9606 GN=RPS7 PE=1 SV=1 | RPS7 | _SFQK[Acetyl (K)]IQVR_ |
| P62081 | 155 | K | 1.5286103 | Up | Small ribosomal subunit protein eS7 OS=Homo sapiens OX=9606 GN=RPS7 PE=1 SV=1 | RPS7 | _LIK[Acetyl (K)]VHLDK_ |
| P62081 | 178 | K | 2.581945453 | Up | Small ribosomal subunit protein eS7 OS=Homo sapiens OX=9606 GN=RPS7 PE=1 SV=1 | RPS7 | _VETFSGVYK[Acetyl (K)]K_ |
| P62136 | 141 | K | 0.564001661 | Down | Serine/threonine-protein phosphatase PP1-alpha catalytic subunit OS=Homo sapiens OX=9606 GN=PPP1CA PE=1 SV=1 | PPP1CA | _IYGFYDEC[Carbamidomethyl (C)]K[Acetyl (K)]R_ |
| P62166 | 25 | K | 0.624292457 | Down | Neuronal calcium sensor 1 OS=Homo sapiens OX=9606 GN=NCS1 PE=1 SV=2 | NCS1 | _TYFTEK[Acetyl (K)]EVQQWYK_ |
| P62244 | 124 | K | 0.5953253 | Down | Small ribosomal subunit protein uS8 OS=Homo sapiens OX=9606 GN=RPS15A PE=1 SV=2 | RPS15A | _HTGGK[Acetyl (K)]ILGFFF_ |
| P62249 | 50 | K | 0.19732027 | Down | Small ribosomal subunit protein uS9 OS=Homo sapiens OX=9606 GN=RPS16 PE=1 SV=2 | RPS16 | _TLQYK[Acetyl (K)]LLEPVLLLGK_ |
| P62273 | 48 | K | 19.68368694 | Up | Small ribosomal subunit protein uS14 OS=Homo sapiens OX=9606 GN=RPS29 PE=1 SV=2 | RPS29 | _QYAK[Acetyl (K)]DIGFIK_ |
| P62280 | 144 | K | 0.100353704 | Down | Small ribosomal subunit protein uS17 OS=Homo sapiens OX=9606 GN=RPS11 PE=1 SV=3 | RPS11 | _FNVLK[Acetyl (K)]VTK_ |
| P62312 | 23 | K | 2.567111246 | Up | U6 snRNA-associated Sm-like protein LSm6 OS=Homo sapiens OX=9606 GN=LSM6 PE=1 SV=1 | LSM6 | _PVVVK[Acetyl (K)]LNSGVDYR_ |
| P62312 | 77 | K | 2.063113834 | Up | U6 snRNA-associated Sm-like protein LSm6 OS=Homo sapiens OX=9606 GN=LSM6 PE=1 SV=1 | LSM6 | _GNNVLYISTQK[Acetyl (K)]R_ |
| P62333 | 383 | K | 0.383733375 | Down | 26S proteasome regulatory subunit 10B OS=Homo sapiens OX=9606 GN=PSMC6 PE=1 SV=1 | PSMC6 | _KLESK[Acetyl (K)]LDYKPV_ |
| P62424 | 20 | K | 0.2396066 | Down | Large ribosomal subunit protein eL8 OS=Homo sapiens OX=9606 GN=RPL7A PE=1 SV=2 | RPL7A | _VAPAPAVVK[Acetyl (K)]K_ |
| P62701 | 22 | K | 0.089641187 | Down | Small ribosomal subunit protein eS4, X isoform OS=Homo sapiens OX=9606 GN=RPS4X PE=1 SV=2 | RPS4X | _HWMLDK[Acetyl (K)]LTGVFAPR_ |
| P62753 | 143 | K | 0.342371459 | Down | Small ribosomal subunit protein eS6 OS=Homo sapiens OX=9606 GN=RPS6 PE=1 SV=1 | RPS6 | _K[Acetyl (K)]LFNLSK_ |
| P62753 | 211 | K | 0.371060263 | Down | Small ribosomal subunit protein eS6 OS=Homo sapiens OX=9606 GN=RPS6 PE=1 SV=1 | RPS6 | _NKEEAAEYAK[Acetyl (K)]LLAK_ |
| P62805 | 17 | K | 3.672888087 | Up | Histone H4 OS=Homo sapiens OX=9606 GN=H4C1 PE=1 SV=2 | H4C1 | _GGKGLGK[Acetyl (K)]GGAK[Acetyl (K)]R_ |
| P62805 | 60 | K | 0.508382844 | Down | Histone H4 OS=Homo sapiens OX=9606 GN=H4C1 PE=1 SV=2 | H4C1 | _GVLK[Acetyl (K)]VFLENVIR_ |
| P62820 | 61 | K | 1.906494809 | Up | Ras-related protein Rab-1A OS=Homo sapiens OX=9606 GN=RAB1A PE=1 SV=3 | RAB1A | _TIK[Acetyl (K)]LQIWDTAGQER_ |
| P62820 | 191 | K | 0.666408728 | Down | Ras-related protein Rab-1A OS=Homo sapiens OX=9606 GN=RAB1A PE=1 SV=3 | RAB1A | _SNVK[Acetyl (K)]IQSTPVK_ |
| P62851 | 57 | K | 0.397712386 | Down | Small ribosomal subunit protein eS25 OS=Homo sapiens OX=9606 GN=RPS25 PE=1 SV=1 | RPS25 | _ATYDK[Acetyl (K)]LC[Carbamidomethyl (C)]K_ |
| P62854 | 70 | K | 3.11030294 | Up | Small ribosomal subunit protein eS26 OS=Homo sapiens OX=9606 GN=RPS26 PE=1 SV=3 | RPS26 | _LYVK[Acetyl (K)]LHYC[Carbamidomethyl (C)]VSC[Carbamidomethyl (C)]AIHSK_ |
| P62854 | 82 | K | 5.759801974 | Up | Small ribosomal subunit protein eS26 OS=Homo sapiens OX=9606 GN=RPS26 PE=1 SV=3 | RPS26 | _LHYC[Carbamidomethyl (C)]VSC[Carbamidomethyl (C)]AIHSK[Acetyl (K)]VVR_ |
| P62857 | 10 | K | 2.959757353 | Up | Small ribosomal subunit protein eS28 OS=Homo sapiens OX=9606 GN=RPS28 PE=1 SV=1 | RPS28 | _VQPIK[Acetyl (K)]LAR_ |
| P62861 | 125 | K | 2.389196261 | Up | Ubiquitin-like FUBI-ribosomal protein eS30 fusion protein OS=Homo sapiens OX=9606 GN=FAU PE=1 SV=2 | FAU | _FVNVVPTFGK[Acetyl (K)]K_ |
| P62888 | 42 | K | 86.4794415 | Up | Large ribosomal subunit protein eL30 OS=Homo sapiens OX=9606 GN=RPL30 PE=1 SV=2 | RPL30 | _QGK[Acetyl (K)]AK[Acetyl (K)]LVILANNC[Carbamidomethyl (C)]PALR_ |
| P62888 | 44 | K | 3.193492127 | Up | Large ribosomal subunit protein eL30 OS=Homo sapiens OX=9606 GN=RPL30 PE=1 SV=2 | RPL30 | _AK[Acetyl (K)]LVILANNC[Carbamidomethyl (C)]PALR_ |
| P62888 | 87 | K | 2.369821812 | Up | Large ribosomal subunit protein eL30 OS=Homo sapiens OX=9606 GN=RPL30 PE=1 SV=2 | RPL30 | _TGVHHYSGNNIELGTAC[Carbamidomethyl (C)]GK[Acetyl (K)]YYR_ |
| P62899 | 75 | K | 0.545030113 | Down | Large ribosomal subunit protein eL31 OS=Homo sapiens OX=9606 GN=RPL31 PE=1 SV=1 | RPL31 | _AVWAK[Acetyl (K)]GIR_ |
| P62913 | 38 | K | 0.348981488 | Down | Large ribosomal subunit protein uL5 OS=Homo sapiens OX=9606 GN=RPL11 PE=1 SV=2 | RPL11 | _AAK[Acetyl (K)]VLEQLTGQTPVFSK_ |
| P62913 | 67 | K | 0.106076774 | Down | Large ribosomal subunit protein uL5 OS=Homo sapiens OX=9606 GN=RPL11 PE=1 SV=2 | RPL11 | _NEK[Acetyl (K)]IAVHC[Carbamidomethyl (C)]TVR_ |
| P62913 | 169 | K | 0.370235263 | Down | Large ribosomal subunit protein uL5 OS=Homo sapiens OX=9606 GN=RPL11 PE=1 SV=2 | RPL11 | _WFQQK[Acetyl (K)]YDGIILPGK_ |
| P62917 | 42 | K | 0.348519323 | Down | Large ribosomal subunit protein uL2 OS=Homo sapiens OX=9606 GN=RPL8 PE=1 SV=2 | RPL8 | _HGYIK[Acetyl (K)]GIVK_ |
| P62917 | 60 | K | 0.65216518 | Down | Large ribosomal subunit protein uL2 OS=Homo sapiens OX=9606 GN=RPL8 PE=1 SV=2 | RPL8 | _GAPLAK[Acetyl (K)]VVFR_ |
| P62937 | 131 | K | 1.555644062 | Up | Peptidyl-prolyl cis-trans isomerase A OS=Homo sapiens OX=9606 GN=PPIA PE=1 SV=2 | PPIA | _HVVFGK[Acetyl (K)]VK_ |
| P62979 | 6 | K | 1.59431637 | Up | Ubiquitin-ribosomal protein eS31 fusion protein OS=Homo sapiens OX=9606 GN=RPS27A PE=1 SV=2 | RPS27A | _MQIFVK[Acetyl (K)]TLTGK_ |
| P62979 | 48 | K | 1.949489042 | Up | Ubiquitin-ribosomal protein eS31 fusion protein OS=Homo sapiens OX=9606 GN=RPS27A PE=1 SV=2 | RPS27A | _LIFAGK[Acetyl (K)]QLEDGR_ |
| P62979 | 104 | K | 0.205529011 | Down | Ubiquitin-ribosomal protein eS31 fusion protein OS=Homo sapiens OX=9606 GN=RPS27A PE=1 SV=2 | RPS27A | _LAVLK[Acetyl (K)]YYK_ |
| P62979 | 143 | K | 0.444274029 | Down | Ubiquitin-ribosomal protein eS31 fusion protein OS=Homo sapiens OX=9606 GN=RPS27A PE=1 SV=2 | RPS27A | _HYC[Carbamidomethyl (C)]GK[Acetyl (K)]C[Carbamidomethyl (C)]C[Carbamidomethyl (C)]LTYC[Carbamidomethyl (C)]FNKPEDK_ |
| P62979 | 152 | K | 0.244341353 | Down | Ubiquitin-ribosomal protein eS31 fusion protein OS=Homo sapiens OX=9606 GN=RPS27A PE=1 SV=2 | RPS27A | _C[Carbamidomethyl (C)]C[Carbamidomethyl (C)]LTYC[Carbamidomethyl (C)]FNK[Acetyl (K)]PEDK_ |
| P63010 | 265 | K | 1.895118711 | Up | AP-2 complex subunit beta OS=Homo sapiens OX=9606 GN=AP2B1 PE=1 SV=1 | AP2B1 | _VLMK[Acetyl (K)]FLELLPK_ |
| P63096 | 197 | K | 1.767229379 | Up | Guanine nucleotide-binding protein G(i) subunit alpha-1 OS=Homo sapiens OX=9606 GN=GNAI1 PE=1 SV=2 | GNAI1 | _DLHFK[Acetyl (K)]MFDVGGQR_ |
| P63241 | 67 | K | 0.482106478 | Down | Eukaryotic translation initiation factor 5A-1 OS=Homo sapiens OX=9606 GN=EIF5A PE=1 SV=2 | EIF5A | _VHLVGIDIFTGK[Acetyl (K)]K_ |
| P63244 | 44 | K | 0.256108784 | Down | Small ribosomal subunit protein RACK1 OS=Homo sapiens OX=9606 GN=RACK1 PE=1 SV=3 | RACK1 | _TIIM[Oxidation (M)]WK[Acetyl (K)]LTR_ |
| P63244 | 175 | K | 0.418102965 | Down | Small ribosomal subunit protein RACK1 OS=Homo sapiens OX=9606 GN=RACK1 PE=1 SV=3 | RACK1 | _LVK[Acetyl (K)]VWNLANC[Carbamidomethyl (C)]K_ |
| P63244 | 183 | K | 0.477118226 | Down | Small ribosomal subunit protein RACK1 OS=Homo sapiens OX=9606 GN=RACK1 PE=1 SV=3 | RACK1 | _VWNLANC[Carbamidomethyl (C)]K[Acetyl (K)]LK_ |
| P67809 | 64 | K | 0.526254797 | Down | Y-box-binding protein 1 OS=Homo sapiens OX=9606 GN=YBX1 PE=1 SV=3 | YBX1 | _VLGTVK[Acetyl (K)]WFNVR_ |
| P68104 | 55 | K | 0.547011437 | Down | Elongation factor 1-alpha 1 OS=Homo sapiens OX=9606 GN=EEF1A1 PE=1 SV=1 | EEF1A1 | _GSFK[Acetyl (K)]YAWVLDK_ |
| P68104 | 450 | K | 0.245474184 | Down | Elongation factor 1-alpha 1 OS=Homo sapiens OX=9606 GN=EEF1A1 PE=1 SV=1 | EEF1A1 | _AAGAGK[Acetyl (K)]VTK[Acetyl (K)]SAQK[Acetyl (K)]AQK_ |
| P68104 | 453 | K | 0.245474184 | Down | Elongation factor 1-alpha 1 OS=Homo sapiens OX=9606 GN=EEF1A1 PE=1 SV=1 | EEF1A1 | _AAGAGK[Acetyl (K)]VTK[Acetyl (K)]SAQK[Acetyl (K)]AQK_ |
| P68104 | 457 | K | 0.245474184 | Down | Elongation factor 1-alpha 1 OS=Homo sapiens OX=9606 GN=EEF1A1 PE=1 SV=1 | EEF1A1 | _AAGAGK[Acetyl (K)]VTK[Acetyl (K)]SAQK[Acetyl (K)]AQK_ |
| P68104 | 460 | K | 0.47698668 | Down | Elongation factor 1-alpha 1 OS=Homo sapiens OX=9606 GN=EEF1A1 PE=1 SV=1 | EEF1A1 | _AAGAGK[Acetyl (K)]VTK[Acetyl (K)]SAQK[Acetyl (K)]AQK[Acetyl (K)]AK_ |
| P68431 | 19 | K | 6.023692701 | Up | Histone H3.1 OS=Homo sapiens OX=9606 GN=H3C1 PE=1 SV=2 | H3C1 | _K[Acetyl (K)]QLATK[Acetyl (K)]AAR_ |
| P68431 | 24 | K | 6.079123174 | Up | Histone H3.1 OS=Homo sapiens OX=9606 GN=H3C1 PE=1 SV=2 | H3C1 | _K[Acetyl (K)]QLATK[Acetyl (K)]AAR_ |
| P68431 | 57 | K | 0.610290398 | Down | Histone H3.1 OS=Homo sapiens OX=9606 GN=H3C1 PE=1 SV=2 | H3C1 | _YQK[Acetyl (K)]STELLIR_ |
| P78347 | 526 | K | 0.482436115 | Down | General transcription factor II-I OS=Homo sapiens OX=9606 GN=GTF2I PE=1 SV=2 | GTF2I | _LEK[Acetyl (K)]IIQVGNR_ |
| P78347 | 660 | K | 0.610110677 | Down | General transcription factor II-I OS=Homo sapiens OX=9606 GN=GTF2I PE=1 SV=2 | GTF2I | _KPELVISYLPPGMASK[Acetyl (K)]INTK_ |
| P78371 | 284 | K | 0.636272658 | Down | T-complex protein 1 subunit beta OS=Homo sapiens OX=9606 GN=CCT2 PE=1 SV=4 | CCT2 | _ILK[Acetyl (K)]HGINC[Carbamidomethyl (C)]FINR_ |
| P78406 | 137 | K | 0.576357184 | Down | mRNA export factor RAE1 OS=Homo sapiens OX=9606 GN=RAE1 PE=1 SV=1 | RAE1 | _TIHWIK[Acetyl (K)]APNYSC[Carbamidomethyl (C)]VMTGSWDK_ |
| P78417 | 114 | K | 0.658221598 | Down | Glutathione S-transferase omega-1 OS=Homo sapiens OX=9606 GN=GSTO1 PE=1 SV=2 | GSTO1 | _AC[Carbamidomethyl (C)]QK[Acetyl (K)]MILELFSK_ |
| P78417 | 152 | K | 2.030227221 | Up | Glutathione S-transferase omega-1 OS=Homo sapiens OX=9606 GN=GSTO1 PE=1 SV=2 | GSTO1 | _EFTK[Acetyl (K)]LEEVLTNK_ |
| P78527 | 1744 | K | 0.554111185 | Down | DNA-dependent protein kinase catalytic subunit OS=Homo sapiens OX=9606 GN=PRKDC PE=1 SV=3 | PRKDC | _FNNYVDC[Carbamidomethyl (C)]MK[Acetyl (K)]K_ |
| P78527 | 2702 | K | 0.341684962 | Down | DNA-dependent protein kinase catalytic subunit OS=Homo sapiens OX=9606 GN=PRKDC PE=1 SV=3 | PRKDC | _SVGPDFGK[Acetyl (K)]K_ |
| P78527 | 2738 | K | 0.313616725 | Down | DNA-dependent protein kinase catalytic subunit OS=Homo sapiens OX=9606 GN=PRKDC PE=1 SV=3 | PRKDC | _DQEK[Acetyl (K)]LSLMYAR_ |
| P78527 | 3840 | K | 0.370396002 | Down | DNA-dependent protein kinase catalytic subunit OS=Homo sapiens OX=9606 GN=PRKDC PE=1 SV=3 | PRKDC | _APPC[Carbamidomethyl (C)]EYK[Acetyl (K)]DWLTK_ |
| P82650 | 255 | K | 0.329584994 | Down | Small ribosomal subunit protein mS22 OS=Homo sapiens OX=9606 GN=MRPS22 PE=1 SV=1 | MRPS22 | _TYEDIDK[Acetyl (K)]R_ |
| P83881 | 61 | K | 0.499330084 | Down | Large ribosomal subunit protein eL42 OS=Homo sapiens OX=9606 GN=RPL36A PE=1 SV=2 | RPL36A | _AK[Acetyl (K)]TTK[Acetyl (K)]K[Acetyl (K)]IVLR_ |
| P83881 | 64 | K | 0.499330084 | Down | Large ribosomal subunit protein eL42 OS=Homo sapiens OX=9606 GN=RPL36A PE=1 SV=2 | RPL36A | _AK[Acetyl (K)]TTK[Acetyl (K)]K[Acetyl (K)]IVLR_ |
| P83881 | 65 | K | 0.499330084 | Down | Large ribosomal subunit protein eL42 OS=Homo sapiens OX=9606 GN=RPL36A PE=1 SV=2 | RPL36A | _AK[Acetyl (K)]TTK[Acetyl (K)]K[Acetyl (K)]IVLR_ |
| P84022 | 378 | K | 3.132923333 | Up | Mothers against decapentaplegic homolog 3 OS=Homo sapiens OX=9606 GN=SMAD3 PE=1 SV=1 | SMAD3 | _MSFVK[Acetyl (K)]GWGAEYR_ |
| P84098 | 144 | K | 0.228107203 | Down | Large ribosomal subunit protein eL19 OS=Homo sapiens OX=9606 GN=RPL19 PE=1 SV=1 | RPL19 | _ILMEHIHK[Acetyl (K)]LK_ |
| P84157 | 147 | K | 0.525928901 | Down | Matrix-remodeling-associated protein 7 OS=Homo sapiens OX=9606 GN=MXRA7 PE=1 SV=1 | MXRA7 | _YSPGK[Acetyl (K)]LR_ |
| P85037 | 365 | K | 0.639584259 | Down | Forkhead box protein K1 OS=Homo sapiens OX=9606 GN=FOXK1 PE=1 SV=1 | FOXK1 | _YFIK[Acetyl (K)]VPR_ |
| P99999 | 87 | K | 2.885939309 | Up | Cytochrome c OS=Homo sapiens OX=9606 GN=CYCS PE=1 SV=2 | CYCS | _MIFVGIK[Acetyl (K)]K_ |
| P99999 | 100 | K | 1.931548442 | Up | Cytochrome c OS=Homo sapiens OX=9606 GN=CYCS PE=1 SV=2 | CYCS | _ADLIAYLK[Acetyl (K)]K_ |
| Q00169 | 60 | K | 1.651411574 | Up | Phosphatidylinositol transfer protein alpha isoform OS=Homo sapiens OX=9606 GN=PITPNA PE=1 SV=2 | PITPNA | _GQYTHK[Acetyl (K)]IYHLQSK_ |
| Q00325 | 355 | K | 0.625094567 | Down | Solute carrier family 25 member 3 OS=Homo sapiens OX=9606 GN=SLC25A3 PE=1 SV=2 | SLC25A3 | _LPRPPPPEMPESLK[Acetyl (K)]K_ |
| Q00577 | 266 | K | 5.165243478 | Up | Transcriptional activator protein Pur-alpha OS=Homo sapiens OX=9606 GN=PURA PE=1 SV=2 | PURA | _VWAK[Acetyl (K)]FGHTFC[Carbamidomethyl (C)]K_ |
| Q00610 | 78 | K | 0.6304029 | Down | Clathrin heavy chain 1 OS=Homo sapiens OX=9606 GN=CLTC PE=1 SV=5 | CLTC | _RPISADSAIMNPASK[Acetyl (K)]VIALK_ |
| Q00610 | 798 | K | 0.521264237 | Down | Clathrin heavy chain 1 OS=Homo sapiens OX=9606 GN=CLTC PE=1 SV=5 | CLTC | _NNLQK[Acetyl (K)]YIEIYVQK_ |
| Q00610 | 856 | K | 1.961754253 | Up | Clathrin heavy chain 1 OS=Homo sapiens OX=9606 GN=CLTC PE=1 SV=5 | CLTC | _LK[Acetyl (K)]LLLPWLEAR_ |
| Q00610 | 1209 | K | 0.539820146 | Down | Clathrin heavy chain 1 OS=Homo sapiens OX=9606 GN=CLTC PE=1 SV=5 | CLTC | _C[Carbamidomethyl (C)]YDEK[Acetyl (K)]MYDAAK_ |
| Q00610 | 1441 | K | 2.923039278 | Up | Clathrin heavy chain 1 OS=Homo sapiens OX=9606 GN=CLTC PE=1 SV=5 | CLTC | _AVNYFSK[Acetyl (K)]VK_ |
| Q00610 | 1516 | K | 0.504604583 | Down | Clathrin heavy chain 1 OS=Homo sapiens OX=9606 GN=CLTC PE=1 SV=5 | CLTC | _IAAYLFK[Acetyl (K)]GNNR_ |
| Q00688 | 170 | K | 1.522048777 | Up | Peptidyl-prolyl cis-trans isomerase FKBP3 OS=Homo sapiens OX=9606 GN=FKBP3 PE=1 SV=1 | FKBP3 | _VGVGK[Acetyl (K)]VIR_ |
| Q00765 | 147 | K | 1.697151622 | Up | Receptor expression-enhancing protein 5 OS=Homo sapiens OX=9606 GN=REEP5 PE=1 SV=3 | REEP5 | _PFFLK[Acetyl (K)]HESQMDSVVK_ |
| Q00839 | 9 | K | 2.301777761 | Up | Heterogeneous nuclear ribonucleoprotein U OS=Homo sapiens OX=9606 GN=HNRNPU PE=1 SV=6 | HNRNPU | _[Acetyl (Protein N-term)]SSSPVNVK[Acetyl (K)]K_ |
| Q00839 | 339 | K | 0.657888628 | Down | Heterogeneous nuclear ribonucleoprotein U OS=Homo sapiens OX=9606 GN=HNRNPU PE=1 SV=6 | HNRNPU | _VC[Carbamidomethyl (C)]FEMK[Acetyl (K)]VTEK_ |
| Q00839 | 352 | K | 1.78155565 | Up | Heterogeneous nuclear ribonucleoprotein U OS=Homo sapiens OX=9606 GN=HNRNPU PE=1 SV=6 | HNRNPU | _HLYTK[Acetyl (K)]DIDIHEVR_ |
| Q00839 | 551 | K | 1.727432418 | Up | Heterogeneous nuclear ribonucleoprotein U OS=Homo sapiens OX=9606 GN=HNRNPU PE=1 SV=6 | HNRNPU | _QMADTGK[Acetyl (K)]LNTLLQR_ |
| Q00839 | 626 | K | 0.478970589 | Down | Heterogeneous nuclear ribonucleoprotein U OS=Homo sapiens OX=9606 GN=HNRNPU PE=1 SV=6 | HNRNPU | _AEVEGK[Acetyl (K)]DLPEHAVLK_ |
| Q01082 | 2362 | K | 0.381960248 | Down | Spectrin beta chain, non-erythrocytic 1 OS=Homo sapiens OX=9606 GN=SPTBN1 PE=1 SV=2 | SPTBN1 | _FSLFGK[Acetyl (K)]K_ |
| Q01130 | 36 | K | 2.571609633 | Up | Serine/arginine-rich splicing factor 2 OS=Homo sapiens OX=9606 GN=SRSF2 PE=1 SV=4 | SRSF2 | _VFEK[Acetyl (K)]YGR_ |
| Q01518 | 465 | K | 0.30209206 | Down | Adenylyl cyclase-associated protein 1 OS=Homo sapiens OX=9606 GN=CAP1 PE=1 SV=5 | CAP1 | _TLWNGQK[Acetyl (K)]LVTTVTEIAG_ |
| Q01581 | 55 | K | 0.438287318 | Down | Hydroxymethylglutaryl-CoA synthase, cytoplasmic OS=Homo sapiens OX=9606 GN=HMGCS1 PE=1 SV=2 | HMGCS1 | _YTIGLGQAK[Acetyl (K)]MGFC[Carbamidomethyl (C)]TDR_ |
| Q01780 | 408 | K | 0.596637391 | Down | Exosome complex component 10 OS=Homo sapiens OX=9606 GN=EXOSC10 PE=1 SV=2 | EXOSC10 | _HSLDHLLK[Acetyl (K)]LYC[Carbamidomethyl (C)]NVDSNK_ |
| Q01813 | 688 | K | 1.76087646 | Up | ATP-dependent 6-phosphofructokinase, platelet type OS=Homo sapiens OX=9606 GN=PFKP PE=1 SV=2 | PFKP | _NFGTK[Acetyl (K)]ISAR_ |
| Q01813 | 700 | K | 2.51064651 | Up | ATP-dependent 6-phosphofructokinase, platelet type OS=Homo sapiens OX=9606 GN=PFKP PE=1 SV=2 | PFKP | _AMEWITAK[Acetyl (K)]LK_ |
| Q01844 | 439 | K | 2.790276675 | Up | RNA-binding protein EWS OS=Homo sapiens OX=9606 GN=EWSR1 PE=1 SV=1 | EWSR1 | _DFQGSK[Acetyl (K)]LK_ |
| Q02241 | 384 | K | 0.413569367 | Down | Kinesin-like protein KIF23 OS=Homo sapiens OX=9606 GN=KIF23 PE=1 SV=3 | KIF23 | _ENQMYGTNK[Acetyl (K)]MVPYR_ |
| Q02241 | 392 | K | 2.401868801 | Up | Kinesin-like protein KIF23 OS=Homo sapiens OX=9606 GN=KIF23 PE=1 SV=3 | KIF23 | _DSK[Acetyl (K)]LTHLFK_ |
| Q02878 | 192 | K | 1.857865526 | Up | Large ribosomal subunit protein eL6 OS=Homo sapiens OX=9606 GN=RPL6 PE=1 SV=3 | RPL6 | _THQK[Acetyl (K)]FVIATSTK_ |
| Q02878 | 200 | K | 0.388270493 | Down | Large ribosomal subunit protein eL6 OS=Homo sapiens OX=9606 GN=RPL6 PE=1 SV=3 | RPL6 | _FVIATSTK[Acetyl (K)]IDISNVK_ |
| Q02952 | 177 | K | 0.61973261 | Down | A-kinase anchor protein 12 OS=Homo sapiens OX=9606 GN=AKAP12 PE=1 SV=4 | AKAP12 | _VFK[Acetyl (K)]FVGFK_ |
| Q02952 | 182 | K | 0.514476745 | Down | A-kinase anchor protein 12 OS=Homo sapiens OX=9606 GN=AKAP12 PE=1 SV=4 | AKAP12 | _FVGFK[Acetyl (K)]FTVK_ |
| Q03164 | 2521 | K | 0.614697914 | Down | Histone-lysine N-methyltransferase 2A OS=Homo sapiens OX=9606 GN=KMT2A PE=1 SV=5 | KMT2A | _TVK[Acetyl (K)]VTLTPLK_ |
| Q03188 | 119 | K | 1.651537163 | Up | Centromere protein C OS=Homo sapiens OX=9606 GN=CENPC PE=1 SV=2 | CENPC | _SVQAHEVHQK[Acetyl (K)]ILATDVSSK_ |
| Q03252 | 77 | K | 1.672438637 | Up | Lamin-B2 OS=Homo sapiens OX=9606 GN=LMNB2 PE=1 SV=4 | LMNB2 | _LLLK[Acetyl (K)]ISEK_ |
| Q04637 | 1019 | K | 0.637444744 | Down | Eukaryotic translation initiation factor 4 gamma 1 OS=Homo sapiens OX=9606 GN=EIF4G1 PE=1 SV=4 | EIF4G1 | _EHIK[Acetyl (K)]VQQLMAK_ |
| Q04724 | 508 | K | 2.136472128 | Up | Transducin-like enhancer protein 1 OS=Homo sapiens OX=9606 GN=TLE1 PE=1 SV=2 | TLE1 | _GC[Carbamidomethyl (C)]VK[Acetyl (K)]VWDISHPGNK_ |
| Q04837 | 103 | K | 0.47144132 | Down | Single-stranded DNA-binding protein, mitochondrial OS=Homo sapiens OX=9606 GN=SSBP1 PE=1 SV=1 | SSBP1 | _DVAYQYVK[Acetyl (K)]K_ |
| Q06203 | 81 | K | 2.076507873 | Up | Amidophosphoribosyltransferase OS=Homo sapiens OX=9606 GN=PPAT PE=1 SV=1 | PPAT | _K[Acetyl (K)]LYVSNLGIGHTR_ |
| Q06203 | 403 | K | 0.6110721 | Down | Amidophosphoribosyltransferase OS=Homo sapiens OX=9606 GN=PPAT PE=1 SV=1 | PPAT | _GNTISPIIK[Acetyl (K)]LLK_ |
| Q06265 | 101 | K | 2.134725143 | Up | Exosome complex component RRP45 OS=Homo sapiens OX=9606 GN=EXOSC9 PE=1 SV=3 | EXOSC9 | _QSDLLVK[Acetyl (K)]LNR_ |
| Q06830 | 7 | K | 0.471067572 | Down | Peroxiredoxin-1 OS=Homo sapiens OX=9606 GN=PRDX1 PE=1 SV=1 | PRDX1 | _[Acetyl (Protein N-term)]SSGNAK[Acetyl (K)]IGHPAPNFK_ |
| Q06830 | 92 | K | 0.594539298 | Down | Peroxiredoxin-1 OS=Homo sapiens OX=9606 GN=PRDX1 PE=1 SV=1 | PRDX1 | _LNC[Carbamidomethyl (C)]QVIGASVDSHFC[Carbamidomethyl (C)]HLAWVNTPK[Acetyl (K)]K_ |
| Q07666 | 139 | K | 0.47524752 | Down | KH domain-containing, RNA-binding, signal transduction-associated protein 1 OS=Homo sapiens OX=9606 GN=KHDRBS1 PE=1 SV=1 | KHDRBS1 | _K[Acetyl (K)]DDEENYLDLFSHK_ |
| Q07866 | 454 | K | 0.487539494 | Down | Kinesin light chain 1 OS=Homo sapiens OX=9606 GN=KLC1 PE=1 SV=2 | KLC1 | _DGTSFGEYGGWYK[Acetyl (K)]AC[Carbamidomethyl (C)]K_ |
| Q07955 | 30 | K | 0.530736017 | Down | Serine/arginine-rich splicing factor 1 OS=Homo sapiens OX=9606 GN=SRSF1 PE=1 SV=2 | SRSF1 | _TK[Acetyl (K)]DIEDVFYK_ |
| Q07955 | 138 | K | 0.651488055 | Down | Serine/arginine-rich splicing factor 1 OS=Homo sapiens OX=9606 GN=SRSF1 PE=1 SV=2 | SRSF1 | _VVVSGLPPSGSWQDLK[Acetyl (K)]DHMR_ |
| Q08170 | 23 | K | 3.766797119 | Up | Serine/arginine-rich splicing factor 4 OS=Homo sapiens OX=9606 GN=SRSF4 PE=1 SV=2 | SRSF4 | _FFK[Acetyl (K)]GYGK_ |
| Q08380 | 229 | K | 2.387004554 | Up | Galectin-3-binding protein OS=Homo sapiens OX=9606 GN=LGALS3BP PE=1 SV=1 | LGALS3BP | _C[Carbamidomethyl (C)]FHK[Acetyl (K)]LASAYGAR_ |
| Q08752 | 321 | K | 1.652189259 | Up | Peptidyl-prolyl cis-trans isomerase D OS=Homo sapiens OX=9606 GN=PPID PE=1 SV=3 | PPID | _AQGWQGLK[Acetyl (K)]EYDQALADLKK_ |
| Q08945 | 73 | K | 1.546303771 | Up | FACT complex subunit SSRP1 OS=Homo sapiens OX=9606 GN=SSRP1 PE=1 SV=1 | SSRP1 | _NGHVYK[Acetyl (K)]YDGFR_ |
| Q08J23 | 179 | K | 1.942906222 | Up | RNA cytosine C(5)-methyltransferase NSUN2 OS=Homo sapiens OX=9606 GN=NSUN2 PE=1 SV=2 | NSUN2 | _PHHK[Acetyl (K)]ILDMC[Carbamidomethyl (C)]AAPGSK_ |
| Q09028 | 4 | K | 0.559616454 | Down | Histone-binding protein RBBP4 OS=Homo sapiens OX=9606 GN=RBBP4 PE=1 SV=3 | RBBP4 | _[Acetyl (Protein N-term)]ADK[Acetyl (K)]EAAFDDAVEER_ |
| Q09472 | 418 | K | 0.210177836 | Down | Histone acetyltransferase p300 OS=Homo sapiens OX=9606 GN=EP300 PE=1 SV=2 | EP300 | _HDC[Carbamidomethyl (C)]PVC[Carbamidomethyl (C)]LPLK[Acetyl (K)]NAGDK[Acetyl (K)]R_ |
| Q09472 | 423 | K | 0.210177836 | Down | Histone acetyltransferase p300 OS=Homo sapiens OX=9606 GN=EP300 PE=1 SV=2 | EP300 | _HDC[Carbamidomethyl (C)]PVC[Carbamidomethyl (C)]LPLK[Acetyl (K)]NAGDK[Acetyl (K)]R_ |
| Q09472 | 1554 | K | 0.04306008 | Down | Histone acetyltransferase p300 OS=Homo sapiens OX=9606 GN=EP300 PE=1 SV=2 | EP300 | _NNK[Acetyl (K)]K[Acetyl (K)]TSK[Acetyl (K)]NK[Acetyl (K)]SSLSR_ |
| Q09472 | 1555 | K | 0.04306008 | Down | Histone acetyltransferase p300 OS=Homo sapiens OX=9606 GN=EP300 PE=1 SV=2 | EP300 | _NNK[Acetyl (K)]K[Acetyl (K)]TSK[Acetyl (K)]NK[Acetyl (K)]SSLSR_ |
| Q09472 | 1558 | K | 0.07829209 | Down | Histone acetyltransferase p300 OS=Homo sapiens OX=9606 GN=EP300 PE=1 SV=2 | EP300 | _NNKKTSK[Acetyl (K)]NK[Acetyl (K)]SSLSR_ |
| Q09472 | 1560 | K | 0.07829209 | Down | Histone acetyltransferase p300 OS=Homo sapiens OX=9606 GN=EP300 PE=1 SV=2 | EP300 | _NNKKTSK[Acetyl (K)]NK[Acetyl (K)]SSLSR_ |
| Q09472 | 1583 | K | 0.314434028 | Down | Histone acetyltransferase p300 OS=Homo sapiens OX=9606 GN=EP300 PE=1 SV=2 | EP300 | _KPGMPNVSNDLSQK[Acetyl (K)]LYATMEK_ |
| Q09472 | 1590 | K | 0.585272767 | Down | Histone acetyltransferase p300 OS=Homo sapiens OX=9606 GN=EP300 PE=1 SV=2 | EP300 | _KPGMPNVSNDLSQK[Acetyl (K)]LYATMEK[Acetyl (K)]HK_ |
| Q09472 | 1760 | K | 0.547182411 | Down | Histone acetyltransferase p300 OS=Homo sapiens OX=9606 GN=EP300 PE=1 SV=2 | EP300 | _NANC[Carbamidomethyl (C)]SLPSC[Carbamidomethyl (C)]QK[Acetyl (K)]MK_ |
| Q09472 | 1762 | K | 0.307602202 | Down | Histone acetyltransferase p300 OS=Homo sapiens OX=9606 GN=EP300 PE=1 SV=2 | EP300 | _NANC[Carbamidomethyl (C)]SLPSC[Carbamidomethyl (C)]QK[Acetyl (K)]MK[Acetyl (K)]R_ |
| Q09666 | 303 | K | 1.507748304 | Up | Neuroblast differentiation-associated protein AHNAK OS=Homo sapiens OX=9606 GN=AHNAK PE=1 SV=2 | AHNAK | _AVEVQGPSLESGDHGK[Acetyl (K)]IK_ |
| Q09666 | 4898 | K | 0.582805495 | Down | Neuroblast differentiation-associated protein AHNAK OS=Homo sapiens OX=9606 GN=AHNAK PE=1 SV=2 | AHNAK | _LDFEGPDAK[Acetyl (K)]LSGPSLK_ |
| Q09666 | 5235 | K | 2.016311264 | Up | Neuroblast differentiation-associated protein AHNAK OS=Homo sapiens OX=9606 GN=AHNAK PE=1 SV=2 | AHNAK | _FPK[Acetyl (K)]FSMPK_ |
| Q09666 | 5858 | K | 0.493869511 | Down | Neuroblast differentiation-associated protein AHNAK OS=Homo sapiens OX=9606 GN=AHNAK PE=1 SV=2 | AHNAK | _LQGSGVSLASK[Acetyl (K)]K_ |
| Q0JRZ9 | 737 | K | 2.058336866 | Up | F-BAR domain only protein 2 OS=Homo sapiens OX=9606 GN=FCHO2 PE=1 SV=2 | FCHO2 | _AFWK[Acetyl (K)]LSSISEK_ |
| Q10567 | 67 | K | 0.548893332 | Down | AP-1 complex subunit beta-1 OS=Homo sapiens OX=9606 GN=AP1B1 PE=1 SV=3 | AP1B1 | _K[Acetyl (K)]LVYLYLMNYAK_ |
| Q10567 | 265 | K | 0.627545604 | Down | AP-1 complex subunit beta-1 OS=Homo sapiens OX=9606 GN=AP1B1 PE=1 SV=3 | AP1B1 | _VLMK[Acetyl (K)]FMEMLSK_ |
| Q12830 | 1285 | K | 1.752526449 | Up | Nucleosome-remodeling factor subunit BPTF OS=Homo sapiens OX=9606 GN=BPTF PE=1 SV=3 | BPTF | _MSDPSHTTNK[Acetyl (K)]LYPK_ |
| Q12830 | 2736 | K | 1.867686807 | Up | Nucleosome-remodeling factor subunit BPTF OS=Homo sapiens OX=9606 GN=BPTF PE=1 SV=3 | BPTF | _QNATK[Acetyl (K)]LSALLFK_ |
| Q12872 | 935 | K | 0.434179987 | Down | Splicing factor, suppressor of white-apricot homolog OS=Homo sapiens OX=9606 GN=SFSWAP PE=1 SV=3 | SFSWAP | _ITQDLMAK[Acetyl (K)]VR_ |
| Q12906 | 214 | K | 1.770033872 | Up | Interleukin enhancer-binding factor 3 OS=Homo sapiens OX=9606 GN=ILF3 PE=1 SV=3 | ILF3 | _HAK[Acetyl (K)]WFQAR_ |
| Q12906 | 332 | K | 0.608806435 | Down | Interleukin enhancer-binding factor 3 OS=Homo sapiens OX=9606 GN=ILF3 PE=1 SV=3 | ILF3 | _LAAFGQLHK[Acetyl (K)]VLGM[Oxidation (M)]DPLPSK_ |
| Q12906 | 454 | K | 1.863797481 | Up | Interleukin enhancer-binding factor 3 OS=Homo sapiens OX=9606 GN=ILF3 PE=1 SV=3 | ILF3 | _TAK[Acetyl (K)]LHVAVK_ |
| Q13033 | 511 | K | 0.414644861 | Down | Striatin-3 OS=Homo sapiens OX=9606 GN=STRN3 PE=1 SV=3 | STRN3 | _LWNLQK[Acetyl (K)]TVPAK_ |
| Q13042 | 67 | K | 1.608307855 | Up | Cell division cycle protein 16 homolog OS=Homo sapiens OX=9606 GN=CDC16 PE=1 SV=2 | CDC16 | _KLDK[Acetyl (K)]LYEAC[Carbamidomethyl (C)]R_ |
| Q13042 | 138 | K | 0.505872168 | Down | Cell division cycle protein 16 homolog OS=Homo sapiens OX=9606 GN=CDC16 PE=1 SV=2 | CDC16 | _GK[Acetyl (K)]IYDALDNR_ |
| Q13042 | 208 | K | 0.644667574 | Down | Cell division cycle protein 16 homolog OS=Homo sapiens OX=9606 GN=CDC16 PE=1 SV=2 | CDC16 | _FLFENK[Acetyl (K)]LK_ |
| Q13042 | 248 | K | 1.759455812 | Up | Cell division cycle protein 16 homolog OS=Homo sapiens OX=9606 GN=CDC16 PE=1 SV=2 | CDC16 | _HYYNC[Carbamidomethyl (C)]DFK[Acetyl (K)]MC[Carbamidomethyl (C)]YK_ |
| Q13085 | 1334 | K | 0.461734563 | Down | Acetyl-CoA carboxylase 1 OS=Homo sapiens OX=9606 GN=ACACA PE=1 SV=2 | ACACA | _LTFLVAQK[Acetyl (K)]DFR_ |
| Q13085 | 1354 | K | 0.58690665 | Down | Acetyl-CoA carboxylase 1 OS=Homo sapiens OX=9606 GN=ACACA PE=1 SV=2 | ACACA | _EFPK[Acetyl (K)]FFTFR_ |
| Q13085 | 2302 | K | 0.446933393 | Down | Acetyl-CoA carboxylase 1 OS=Homo sapiens OX=9606 GN=ACACA PE=1 SV=2 | ACACA | _DYVLK[Acetyl (K)]QIR_ |
| Q13098 | 337 | K | 0.615169185 | Down | COP9 signalosome complex subunit 1 OS=Homo sapiens OX=9606 GN=GPS1 PE=1 SV=4 | GPS1 | _FYESK[Acetyl (K)]YASC[Carbamidomethyl (C)]LK_ |
| Q13111 | 558 | K | 0.298734012 | Down | Chromatin assembly factor 1 subunit A OS=Homo sapiens OX=9606 GN=CHAF1A PE=1 SV=3 | CHAF1A | _MK[Acetyl (K)]LLQFC[Carbamidomethyl (C)]ENHR_ |
| Q13112 | 163 | K | 1.538322163 | Up | Chromatin assembly factor 1 subunit B OS=Homo sapiens OX=9606 GN=CHAF1B PE=1 SV=1 | CHAF1B | _GQK[Acetyl (K)]ISIFNEHK_ |
| Q13112 | 494 | K | 0.374870516 | Down | Chromatin assembly factor 1 subunit B OS=Homo sapiens OX=9606 GN=CHAF1B PE=1 SV=1 | CHAF1B | _VTLNTLQAWSK[Acetyl (K)]TTPR_ |
| Q13131 | 80 | K | 0.592711552 | Down | 5'-AMP-activated protein kinase catalytic subunit alpha-1 OS=Homo sapiens OX=9606 GN=PRKAA1 PE=1 SV=4 | PRKAA1 | _EIQNLK[Acetyl (K)]LFR_ |
| Q13148 | 160 | K | 0.262701796 | Down | TAR DNA-binding protein 43 OS=Homo sapiens OX=9606 GN=TARDBP PE=1 SV=1 | TARDBP | _FTEYETQVK[Acetyl (K)]VMSQR_ |
| Q13151 | 99 | K | 0.644106876 | Down | Heterogeneous nuclear ribonucleoprotein A0 OS=Homo sapiens OX=9606 GN=HNRNPA0 PE=1 SV=1 | HNRNPA0 | _K[Acetyl (K)]LFVGGLK_ |
| Q13185 | 44 | K | 1.638617731 | Up | Chromobox protein homolog 3 OS=Homo sapiens OX=9606 GN=CBX3 PE=1 SV=4 | CBX3 | _VVNGK[Acetyl (K)]VEYFLK_ |
| Q13263 | 337 | K | 0.289533632 | Down | Transcription intermediary factor 1-beta OS=Homo sapiens OX=9606 GN=TRIM28 PE=1 SV=5 | TRIM28 | _QHWTMTK[Acetyl (K)]IQK_ |
| Q13263 | 366 | K | 0.38777052 | Down | Transcription intermediary factor 1-beta OS=Homo sapiens OX=9606 GN=TRIM28 PE=1 SV=5 | TRIM28 | _K[Acetyl (K)]LIYFQLHR_ |
| Q13263 | 774 | K | 1.609190747 | Up | Transcription intermediary factor 1-beta OS=Homo sapiens OX=9606 GN=TRIM28 PE=1 SV=5 | TRIM28 | _QFNK[Acetyl (K)]LTEDK_ |
| Q13268 | 79 | K | 0.628770301 | Down | Dehydrogenase/reductase SDR family member 2, mitochondrial OS=Homo sapiens OX=9606 GN=DHRS2 PE=1 SV=4 | DHRS2 | _AMAK[Acetyl (K)]LQGEGLSVAGIVC[Carbamidomethyl (C)]HVGK_ |
| Q13308 | 852 | K | 1.558138548 | Up | Inactive tyrosine-protein kinase 7 OS=Homo sapiens OX=9606 GN=PTK7 PE=1 SV=2 | PTK7 | _ELEMFGK[Acetyl (K)]LNHANVVR_ |
| Q13308 | 1035 | K | 0.629397917 | Down | Inactive tyrosine-protein kinase 7 OS=Homo sapiens OX=9606 GN=PTK7 PE=1 SV=2 | PTK7 | _LPQPEGC[Carbamidomethyl (C)]PSK[Acetyl (K)]LYR_ |
| Q13315 | 2440 | K | 2.138525087 | Up | Serine-protein kinase ATM OS=Homo sapiens OX=9606 GN=ATM PE=1 SV=4 | ATM | _YTVK[Acetyl (K)]VQR_ |
| Q13325 | 257 | K | 0.645855411 | Down | Interferon-induced protein with tetratricopeptide repeats 5 OS=Homo sapiens OX=9606 GN=IFIT5 PE=1 SV=1 | IFIT5 | _YAAK[Acetyl (K)]FYR_ |
| Q13415 | 282 | K | 2.533893448 | Up | Origin recognition complex subunit 1 OS=Homo sapiens OX=9606 GN=ORC1 PE=1 SV=2 | ORC1 | _SQPDK[Acetyl (K)]LQTLSPALK_ |
| Q13416 | 47 | K | 1.684738035 | Up | Origin recognition complex subunit 2 OS=Homo sapiens OX=9606 GN=ORC2 PE=1 SV=2 | ORC2 | _AQLLVNPK[Acetyl (K)]K_ |
| Q13426 | 187 | K | 3.369224212 | Up | DNA repair protein XRCC4 OS=Homo sapiens OX=9606 GN=XRCC4 PE=1 SV=2 | XRCC4 | _FILVLNEK[Acetyl (K)]K_ |
| Q13428 | 296 | K | 1.817454083 | Up | Treacle protein OS=Homo sapiens OX=9606 GN=TCOF1 PE=1 SV=3 | TCOF1 | _ASEK[Acetyl (K)]ILQVR_ |
| Q13428 | 308 | K | 5.733029532 | Up | Treacle protein OS=Homo sapiens OX=9606 GN=TCOF1 PE=1 SV=3 | TCOF1 | _AASAPAK[Acetyl (K)]GTPGK[Acetyl (K)]GATPAPPGK_ |
| Q13428 | 313 | K | 5.733029532 | Up | Treacle protein OS=Homo sapiens OX=9606 GN=TCOF1 PE=1 SV=3 | TCOF1 | _AASAPAK[Acetyl (K)]GTPGK[Acetyl (K)]GATPAPPGK_ |
| Q13428 | 637 | K | 2.111374632 | Up | Treacle protein OS=Homo sapiens OX=9606 GN=TCOF1 PE=1 SV=3 | TCOF1 | _PALK[Acetyl (K)]IPQTK_ |
| Q13435 | 577 | K | 0.475129099 | Down | Splicing factor 3B subunit 2 OS=Homo sapiens OX=9606 GN=SF3B2 PE=1 SV=2 | SF3B2 | _LHDAFFK[Acetyl (K)]WQTK_ |
| Q13442 | 126 | K | 0.501453985 | Down | 28 kDa heat- and acid-stable phosphoprotein OS=Homo sapiens OX=9606 GN=PDAP1 PE=1 SV=1 | PDAP1 | _YMK[Acetyl (K)]MHLAGK_ |
| Q13459 | 201 | K | 0.442786525 | Down | Unconventional myosin-IXb OS=Homo sapiens OX=9606 GN=MYO9B PE=1 SV=3 | MYO9B | _YVK[Acetyl (K)]MYENQQLGK_ |
| Q13485 | 113 | K | 0.636816185 | Down | Mothers against decapentaplegic homolog 4 OS=Homo sapiens OX=9606 GN=SMAD4 PE=1 SV=1 | SMAD4 | _HVK[Acetyl (K)]YC[Carbamidomethyl (C)]QYAFDLK_ |
| Q13496 | 516 | K | 0.660763145 | Down | Myotubularin OS=Homo sapiens OX=9606 GN=MTM1 PE=1 SV=2 | MTM1 | _NPFYTK[Acetyl (K)]EINR_ |
| Q13501 | 165 | K | 16.14332552 | Up | Sequestosome-1 OS=Homo sapiens OX=9606 GN=SQSTM1 PE=1 SV=1 | SQSTM1 | _GHTK[Acetyl (K)]LAFPSPFGHLSEGFSHSR_ |
| Q13509 | 58 | K | 0.434716429 | Down | Tubulin beta-3 chain OS=Homo sapiens OX=9606 GN=TUBB3 PE=1 SV=2 | TUBB3 | _ISVYYNEASSHK[Acetyl (K)]YVPR_ |
| Q13557 | 251 | K | 0.502482865 | Down | Calcium/calmodulin-dependent protein kinase type II subunit delta OS=Homo sapiens OX=9606 GN=CAMK2D PE=1 SV=3 | CAMK2D | _DLINK[Acetyl (K)]MLTINPAK_ |
| Q13618 | 235 | K | 0.603236879 | Down | Cullin-3 OS=Homo sapiens OX=9606 GN=CUL3 PE=1 SV=2 | CUL3 | _FLAENSASVYIK[Acetyl (K)]K_ |
| Q13618 | 542 | K | 0.386650471 | Down | Cullin-3 OS=Homo sapiens OX=9606 GN=CUL3 PE=1 SV=2 | CUL3 | _FYLAK[Acetyl (K)]HSGR_ |
| Q13642 | 124 | K | 0.433569808 | Down | Four and a half LIM domains protein 1 OS=Homo sapiens OX=9606 GN=FHL1 PE=1 SV=4 | FHL1 | _GTVWHK[Acetyl (K)]DC[Carbamidomethyl (C)]FTC[Carbamidomethyl (C)]SNC[Carbamidomethyl (C)]K_ |
| Q13643 | 235 | K | 1.568369332 | Up | Four and a half LIM domains protein 3 OS=Homo sapiens OX=9606 GN=FHL3 PE=1 SV=4 | FHL3 | _RPIVGLGGGK[Acetyl (K)]YVSFEDR_ |
| Q13686 | 120 | K | 12.28601967 | Up | Nucleic acid dioxygenase ALKBH1 OS=Homo sapiens OX=9606 GN=ALKBH1 PE=1 SV=2 | ALKBH1 | _QC[Carbamidomethyl (C)]LK[Acetyl (K)]LYSQKPNVC[Carbamidomethyl (C)]NLDK_ |
| Q13813 | 864 | K | 0.333339264 | Down | Spectrin alpha chain, non-erythrocytic 1 OS=Homo sapiens OX=9606 GN=SPTAN1 PE=1 SV=3 | SPTAN1 | _AK[Acetyl (K)]LHELNQK_ |
| Q13887 | 382 | K | 0.641255384 | Down | Krueppel-like factor 5 OS=Homo sapiens OX=9606 GN=KLF5 PE=1 SV=2 | KLF5 | _IHYC[Carbamidomethyl (C)]DYPGC[Carbamidomethyl (C)]TK[Acetyl (K)]VYTK_ |
| Q13907 | 192 | K | 0.53004211 | Down | Isopentenyl-diphosphate Delta-isomerase 1 OS=Homo sapiens OX=9606 GN=IDI1 PE=1 SV=2 | IDI1 | _AASGEIK[Acetyl (K)]ITPWFK_ |
| Q14008 | 1463 | K | 0.597740426 | Down | Cytoskeleton-associated protein 5 OS=Homo sapiens OX=9606 GN=CKAP5 PE=1 SV=3 | CKAP5 | _GPAEDMSSK[Acetyl (K)]LNQAR_ |
| Q14119 | 100 | K | 2.743537878 | Up | Vascular endothelial zinc finger 1 OS=Homo sapiens OX=9606 GN=VEZF1 PE=1 SV=2 | VEZF1 | _HESC[Carbamidomethyl (C)]HTGIK[Acetyl (K)]LVSR_ |
| Q14149 | 231 | K | 0.59064396 | Down | MORC family CW-type zinc finger protein 3 OS=Homo sapiens OX=9606 GN=MORC3 PE=1 SV=3 | MORC3 | _IPEDLDEITGK[Acetyl (K)]K_ |
| Q14151 | 437 | K | 0.404775649 | Down | Scaffold attachment factor B2 OS=Homo sapiens OX=9606 GN=SAFB2 PE=1 SV=1 | SAFB2 | _VVGAK[Acetyl (K)]VVTNAR_ |
| Q14152 | 285 | K | 0.436583278 | Down | Eukaryotic translation initiation factor 3 subunit A OS=Homo sapiens OX=9606 GN=EIF3A PE=1 SV=1 | EIF3A | _VSTVFWK[Acetyl (K)]SGNALFHASTLHR_ |
| Q14160 | 313 | K | 0.601680391 | Down | Protein scribble homolog OS=Homo sapiens OX=9606 GN=SCRIB PE=1 SV=5 | SCRIB | _LTK[Acetyl (K)]LTNLNVDR_ |
| Q14165 | 85 | K | 0.535781339 | Down | Malectin OS=Homo sapiens OX=9606 GN=MLEC PE=1 SV=1 | MLEC | _ASDYGMK[Acetyl (K)]LPILR_ |
| Q14166 | 451 | K | 0.534013215 | Down | Tubulin--tyrosine ligase-like protein 12 OS=Homo sapiens OX=9606 GN=TTLL12 PE=1 SV=2 | TTLL12 | _VVSK[Acetyl (K)]YIESPVLFLR_ |
| Q14195 | 368 | K | 2.090486506 | Up | Dihydropyrimidinase-related protein 3 OS=Homo sapiens OX=9606 GN=DPYSL3 PE=1 SV=1 | DPYSL3 | _MSVIWDK[Acetyl (K)]AVATGK_ |
| Q14202 | 439 | K | 2.511022862 | Up | Zinc finger MYM-type protein 3 OS=Homo sapiens OX=9606 GN=ZMYM3 PE=1 SV=2 | ZMYM3 | _LC[Carbamidomethyl (C)]SDSC[Carbamidomethyl (C)]FSK[Acetyl (K)]FR_ |
| Q14202 | 489 | K | 1.94637527 | Up | Zinc finger MYM-type protein 3 OS=Homo sapiens OX=9606 GN=ZMYM3 PE=1 SV=2 | ZMYM3 | _FC[Carbamidomethyl (C)]NTTC[Carbamidomethyl (C)]LGAYK[Acetyl (K)]K_ |
| Q14204 | 447 | K | 0.259204315 | Down | Cytoplasmic dynein 1 heavy chain 1 OS=Homo sapiens OX=9606 GN=DYNC1H1 PE=1 SV=5 | DYNC1H1 | _EENLK[Acetyl (K)]MVWR_ |
| Q14204 | 734 | K | 0.560719218 | Down | Cytoplasmic dynein 1 heavy chain 1 OS=Homo sapiens OX=9606 GN=DYNC1H1 PE=1 SV=5 | DYNC1H1 | _TGNVLK[Acetyl (K)]LK_ |
| Q14204 | 4594 | K | 0.309756739 | Down | Cytoplasmic dynein 1 heavy chain 1 OS=Homo sapiens OX=9606 GN=DYNC1H1 PE=1 SV=5 | DYNC1H1 | _WVK[Acetyl (K)]QTNTEK_ |
| Q14232 | 11 | K | 1.506963003 | Up | Translation initiation factor eIF-2B subunit alpha OS=Homo sapiens OX=9606 GN=EIF2B1 PE=1 SV=1 | EIF2B1 | _ELIEYFK[Acetyl (K)]SQMK_ |
| Q14247 | 230 | K | 0.64968553 | Down | Src substrate cortactin OS=Homo sapiens OX=9606 GN=CTTN PE=1 SV=2 | CTTN | _DYVK[Acetyl (K)]GFGGK_ |
| Q14258 | 439 | K | 0.531888058 | Down | E3 ubiquitin/ISG15 ligase TRIM25 OS=Homo sapiens OX=9606 GN=TRIM25 PE=1 SV=2 | TRIM25 | _AK[Acetyl (K)]VLETFLAK_ |
| Q14344 | 66 | K | 2.090632111 | Up | Guanine nucleotide-binding protein subunit alpha-13 OS=Homo sapiens OX=9606 GN=GNA13 PE=1 SV=2 | GNA13 | _STFLK[Acetyl (K)]QMR_ |
| Q14353 | 109 | K | 0.389854549 | Down | Guanidinoacetate N-methyltransferase OS=Homo sapiens OX=9606 GN=GAMT PE=1 SV=1 | GAMT | _QTHK[Acetyl (K)]VIPLK_ |
| Q14493 | 188 | K | 0.657649614 | Down | Histone RNA hairpin-binding protein OS=Homo sapiens OX=9606 GN=SLBP PE=1 SV=1 | SLBP | _SWDQQIK[Acetyl (K)]LWK_ |
| Q14517 | 58 | K | 2.42285599 | Up | Protocadherin Fat 1 OS=Homo sapiens OX=9606 GN=FAT1 PE=1 SV=2 | FAT1 | _TYVGHPVK[Acetyl (K)]MGVYITHPAWEVR_ |
| Q14517 | 230 | K | 17.63657205 | Up | Protocadherin Fat 1 OS=Homo sapiens OX=9606 GN=FAT1 PE=1 SV=2 | FAT1 | _GMK[Acetyl (K)]LYGSSGISSMAK_ |
| Q14517 | 4094 | K | 0.399221584 | Down | Protocadherin Fat 1 OS=Homo sapiens OX=9606 GN=FAT1 PE=1 SV=2 | FAT1 | _C[Carbamidomethyl (C)]QLSPYC[Carbamidomethyl (C)]K[Acetyl (K)]DEPC[Carbamidomethyl (C)]K_ |
| Q14517 | 4236 | K | 1.819686577 | Up | Protocadherin Fat 1 OS=Homo sapiens OX=9606 GN=FAT1 PE=1 SV=2 | FAT1 | _PYFDSK[Acetyl (K)]LNK_ |
| Q14566 | 588 | K | 0.412952459 | Down | DNA replication licensing factor MCM6 OS=Homo sapiens OX=9606 GN=MCM6 PE=1 SV=1 | MCM6 | _ISK[Acetyl (K)]ESEDFIVEQYK_ |
| Q14573 | 1234 | K | 0.609864039 | Down | Inositol 1,4,5-trisphosphate receptor type 3 OS=Homo sapiens OX=9606 GN=ITPR3 PE=1 SV=2 | ITPR3 | _YTHQFLQK[Acetyl (K)]FC[Carbamidomethyl (C)]AGNPGNQALLHK_ |
| Q14573 | 2628 | K | 4.074442247 | Up | Inositol 1,4,5-trisphosphate receptor type 3 OS=Homo sapiens OX=9606 GN=ITPR3 PE=1 SV=2 | ITPR3 | _ILQDK[Acetyl (K)]LNSTMK_ |
| Q14653 | 366 | K | 0.450690183 | Down | Interferon regulatory factor 3 OS=Homo sapiens OX=9606 GN=IRF3 PE=1 SV=1 | IRF3 | _LVMVK[Acetyl (K)]VVPTC[Carbamidomethyl (C)]LR_ |
| Q14669 | 1399 | K | 0.548229689 | Down | E3 ubiquitin-protein ligase TRIP12 OS=Homo sapiens OX=9606 GN=TRIP12 PE=1 SV=1 | TRIP12 | _THTIWYK[Acetyl (K)]PVR_ |
| Q14676 | 812 | K | 2.159899565 | Up | Mediator of DNA damage checkpoint protein 1 OS=Homo sapiens OX=9606 GN=MDC1 PE=1 SV=3 | MDC1 | _QTVDK[Acetyl (K)]VMGIPK_ |
| Q14690 | 471 | K | 0.530867365 | Down | Protein RRP5 homolog OS=Homo sapiens OX=9606 GN=PDCD11 PE=1 SV=3 | PDCD11 | _SYGMLVK[Acetyl (K)]VGEQMR_ |
| Q14690 | 522 | K | 0.524366657 | Down | Protein RRP5 homolog OS=Homo sapiens OX=9606 GN=PDCD11 PE=1 SV=3 | PDCD11 | _LMMTLK[Acetyl (K)]K_ |
| Q14690 | 1102 | K | 0.393325556 | Down | Protein RRP5 homolog OS=Homo sapiens OX=9606 GN=PDCD11 PE=1 SV=3 | PDCD11 | _TFK[Acetyl (K)]YLPISHPR_ |
| Q14690 | 1157 | K | 0.614939243 | Down | Protein RRP5 homolog OS=Homo sapiens OX=9606 GN=PDCD11 PE=1 SV=3 | PDCD11 | _QYQAGQTVTC[Carbamidomethyl (C)]FLK[Acetyl (K)]K_ |
| Q14690 | 1369 | K | 0.470634835 | Down | Protein RRP5 homolog OS=Homo sapiens OX=9606 GN=PDCD11 PE=1 SV=3 | PDCD11 | _ALYNK[Acetyl (K)]HLPEGK_ |
| Q14692 | 1028 | K | 0.143638714 | Down | Ribosome biogenesis protein BMS1 homolog OS=Homo sapiens OX=9606 GN=BMS1 PE=1 SV=1 | BMS1 | _LK[Acetyl (K)]LTGFPYK_ |
| Q14738 | 82 | K | 0.413702483 | Down | Serine/threonine-protein phosphatase 2A 56 kDa regulatory subunit delta isoform OS=Homo sapiens OX=9606 GN=PPP2R5D PE=1 SV=1 | PPP2R5D | _YSGGPQIVK[Acetyl (K)]K_ |
| Q14739 | 601 | K | 1.825381622 | Up | Delta(14)-sterol reductase LBR OS=Homo sapiens OX=9606 GN=LBR PE=1 SV=2 | LBR | _YGVAWEK[Acetyl (K)]YC[Carbamidomethyl (C)]QR_ |
| Q14746 | 195 | K | 0.626465937 | Down | Conserved oligomeric Golgi complex subunit 2 OS=Homo sapiens OX=9606 GN=COG2 PE=1 SV=1 | COG2 | _GMPLLDK[Acetyl (K)]VRPR_ |
| Q147X3 | 233 | K | 0.435299401 | Down | N-alpha-acetyltransferase 30 OS=Homo sapiens OX=9606 GN=NAA30 PE=1 SV=1 | NAA30 | _LITK[Acetyl (K)]DLSEPYSIYTYR_ |
| Q14839 | 504 | K | 0.435199664 | Down | Chromodomain-helicase-DNA-binding protein 4 OS=Homo sapiens OX=9606 GN=CHD4 PE=1 SV=2 | CHD4 | _VQK[Acetyl (K)]ILIWK_ |
| Q14839 | 604 | K | 0.633551808 | Down | Chromodomain-helicase-DNA-binding protein 4 OS=Homo sapiens OX=9606 GN=CHD4 PE=1 SV=2 | CHD4 | _NKDPK[Acetyl (K)]FAEMEER_ |
| Q14847 | 10 | K | 1.577885778 | Up | LIM and SH3 domain protein 1 OS=Homo sapiens OX=9606 GN=LASP1 PE=1 SV=2 | LASP1 | _C[Carbamidomethyl (C)]GK[Acetyl (K)]IVYPTEK_ |
| Q14914 | 178 | K | 0.544912518 | Down | Prostaglandin reductase 1 OS=Homo sapiens OX=9606 GN=PTGR1 PE=1 SV=2 | PTGR1 | _VVGAVGSDEK[Acetyl (K)]VAYLQK_ |
| Q14914 | 295 | K | 1.791514717 | Up | Prostaglandin reductase 1 OS=Homo sapiens OX=9606 GN=PTGR1 PE=1 SV=2 | PTGR1 | _WVLEGK[Acetyl (K)]IQYK_ |
| Q14966 | 1455 | K | 0.605579342 | Down | Zinc finger protein 638 OS=Homo sapiens OX=9606 GN=ZNF638 PE=1 SV=2 | ZNF638 | _SGLAESSSK[Acetyl (K)]FKPTQSSLTR_ |
| Q14966 | 1477 | K | 0.513782518 | Down | Zinc finger protein 638 OS=Homo sapiens OX=9606 GN=ZNF638 PE=1 SV=2 | ZNF638 | _ISALQGK[Acetyl (K)]LSK_ |
| Q14966 | 1480 | K | 1.553932387 | Up | Zinc finger protein 638 OS=Homo sapiens OX=9606 GN=ZNF638 PE=1 SV=2 | ZNF638 | _LSK[Acetyl (K)]LDYR_ |
| Q14966 | 1860 | K | 0.607090252 | Down | Zinc finger protein 638 OS=Homo sapiens OX=9606 GN=ZNF638 PE=1 SV=2 | ZNF638 | _IGK[Acetyl (K)]TLPSEK_ |
| Q14978 | 415 | K | 2.141692381 | Up | Nucleolar and coiled-body phosphoprotein 1 OS=Homo sapiens OX=9606 GN=NOLC1 PE=1 SV=2 | NOLC1 | _QPVGGGQK[Acetyl (K)]LLTR_ |
| Q14980 | 1611 | K | 0.216821566 | Down | Nuclear mitotic apparatus protein 1 OS=Homo sapiens OX=9606 GN=NUMA1 PE=1 SV=2 | NUMA1 | _EQAAEHYK[Acetyl (K)]LQMEK_ |
| Q14997 | 36 | K | 1.52805381 | Up | Proteasome activator complex subunit 4 OS=Homo sapiens OX=9606 GN=PSME4 PE=1 SV=2 | PSME4 | _EIVYNK[Acetyl (K)]LLPYAER_ |
| Q14997 | 1397 | K | 0.468990132 | Down | Proteasome activator complex subunit 4 OS=Homo sapiens OX=9606 GN=PSME4 PE=1 SV=2 | PSME4 | _HWTFEK[Acetyl (K)]VEK_ |
| Q14CS0 | 83 | K | 1.718182461 | Up | UBX domain-containing protein 2B OS=Homo sapiens OX=9606 GN=UBXN2B PE=1 SV=1 | UBXN2B | _PSTGK[Acetyl (K)]IVNELFK_ |
| Q14CX7 | 435 | K | 0.626676101 | Down | N-alpha-acetyltransferase 25, NatB auxiliary subunit OS=Homo sapiens OX=9606 GN=NAA25 PE=1 SV=1 | NAA25 | _NQK[Acetyl (K)]LSVVR_ |
| Q15005 | 71 | K | 1.670168014 | Up | Signal peptidase complex subunit 2 OS=Homo sapiens OX=9606 GN=SPCS2 PE=1 SV=3 | SPCS2 | _VLLEK[Acetyl (K)]YK_ |
| Q15006 | 255 | K | 0.190764626 | Down | ER membrane protein complex subunit 2 OS=Homo sapiens OX=9606 GN=EMC2 PE=1 SV=1 | EMC2 | _DNMK[Acetyl (K)]YASWAASQINR_ |
| Q15008 | 130 | K | 0.661149215 | Down | 26S proteasome non-ATPase regulatory subunit 6 OS=Homo sapiens OX=9606 GN=PSMD6 PE=1 SV=1 | PSMD6 | _TYDK[Acetyl (K)]TVALGHR_ |
| Q15020 | 958 | K | 0.099023833 | Down | Squamous cell carcinoma antigen recognized by T-cells 3 OS=Homo sapiens OX=9606 GN=SART3 PE=1 SV=1 | SART3 | _MSNADFAK[Acetyl (K)]LFLR_ |
| Q15036 | 435 | K | 0.623902492 | Down | Sorting nexin-17 OS=Homo sapiens OX=9606 GN=SNX17 PE=1 SV=1 | SNX17 | _LSSK[Acetyl (K)]LSAVSLR_ |
| Q15046 | 153 | K | 0.654827196 | Down | Lysine--tRNA ligase OS=Homo sapiens OX=9606 GN=KARS1 PE=1 SV=3 | KARS1 | _GEGVK[Acetyl (K)]LQVMANSR_ |
| Q15050 | 226 | K | 0.451875653 | Down | Ribosome biogenesis regulatory protein homolog OS=Homo sapiens OX=9606 GN=RRS1 PE=1 SV=2 | RRS1 | _AMQVAK[Acetyl (K)]VSTASVGR_ |
| Q15050 | 341 | K | 1.788000443 | Up | Ribosome biogenesis regulatory protein homolog OS=Homo sapiens OX=9606 GN=RRS1 PE=1 SV=2 | RRS1 | _GGLGGK[Acetyl (K)]MNSGPPGLGGK_ |
| Q15054 | 390 | K | 1.69854577 | Up | DNA polymerase delta subunit 3 OS=Homo sapiens OX=9606 GN=POLD3 PE=1 SV=2 | POLD3 | _SK[Acetyl (K)]TYLDGEGC[Carbamidomethyl (C)]IVTEK_ |
| Q15061 | 564 | K | 28.45445465 | Up | WD repeat-containing protein 43 OS=Homo sapiens OX=9606 GN=WDR43 PE=1 SV=3 | WDR43 | _TFQK[Acetyl (K)]LSHLHGK_ |
| Q15067 | 198 | K | 0.497861377 | Down | Peroxisomal acyl-coenzyme A oxidase 1 OS=Homo sapiens OX=9606 GN=ACOX1 PE=1 SV=3 | ACOX1 | _GK[Acetyl (K)]C[Carbamidomethyl (C)]YGLHAFIVPIR_ |
| Q15070 | 343 | K | 1.870908763 | Up | Mitochondrial inner membrane protein OXA1L OS=Homo sapiens OX=9606 GN=OXA1L PE=1 SV=3 | OXA1L | _TVLK[Acetyl (K)]IPQR_ |
| Q15084 | 241 | K | 0.465546978 | Down | Protein disulfide-isomerase A6 OS=Homo sapiens OX=9606 GN=PDIA6 PE=1 SV=1 | PDIA6 | _GFPTIK[Acetyl (K)]IFQK_ |
| Q15149 | 191 | K | 2.549686473 | Up | Plectin OS=Homo sapiens OX=9606 GN=PLEC PE=1 SV=3 | PLEC | _WVNK[Acetyl (K)]HLIK_ |
| Q15149 | 567 | K | 1.842837537 | Up | Plectin OS=Homo sapiens OX=9606 GN=PLEC PE=1 SV=3 | PLEC | _LLAAGK[Acetyl (K)]VPQR_ |
| Q15149 | 740 | K | 0.46095889 | Down | Plectin OS=Homo sapiens OX=9606 GN=PLEC PE=1 SV=3 | PLEC | _LDLQYAK[Acetyl (K)]LLNSSK_ |
| Q15154 | 1647 | K | 0.515024537 | Down | Pericentriolar material 1 protein OS=Homo sapiens OX=9606 GN=PCM1 PE=1 SV=5 | PCM1 | _FFHK[Acetyl (K)]QLGSILQDSLAK_ |
| Q15172 | 208 | K | 0.638403221 | Down | Serine/threonine-protein phosphatase 2A 56 kDa regulatory subunit alpha isoform OS=Homo sapiens OX=9606 GN=PPP2R5A PE=1 SV=1 | PPP2R5A | _DFLK[Acetyl (K)]TVLHR_ |
| Q15181 | 288 | K | 0.237726399 | Down | Inorganic pyrophosphatase OS=Homo sapiens OX=9606 GN=PPA1 PE=1 SV=2 | PPA1 | _WFHHQK[Acetyl (K)]N_ |
| Q15185 | 7 | K | 0.517203414 | Down | Prostaglandin E synthase 3 OS=Homo sapiens OX=9606 GN=PTGES3 PE=1 SV=1 | PTGES3 | _MQPASAK[Acetyl (K)]WYDR_ |
| Q15233 | 96 | K | 1.551639371 | Up | Non-POU domain-containing octamer-binding protein OS=Homo sapiens OX=9606 GN=NONO PE=1 SV=4 | NONO | _KLFEK[Acetyl (K)]YGK_ |
| Q15233 | 467 | K | 2.343958157 | Up | Non-POU domain-containing octamer-binding protein OS=Homo sapiens OX=9606 GN=NONO PE=1 SV=4 | NONO | _AAPGAEFAPNK[Acetyl (K)]R_ |
| Q15269 | 523 | K | 0.665531874 | Down | Periodic tryptophan protein 2 homolog OS=Homo sapiens OX=9606 GN=PWP2 PE=1 SV=2 | PWP2 | _SVLASASWDK[Acetyl (K)]TVR_ |
| Q15269 | 596 | K | 2.05249953 | Up | Periodic tryptophan protein 2 homolog OS=Homo sapiens OX=9606 GN=PWP2 PE=1 SV=2 | PWP2 | _ELDK[Acetyl (K)]ITAK_ |
| Q15287 | 186 | K | 0.651384204 | Down | RNA-binding protein with serine-rich domain 1 OS=Homo sapiens OX=9606 GN=RNPS1 PE=1 SV=1 | RNPS1 | _DHIMEIFSTYGK[Acetyl (K)]IK_ |
| Q15366 | 23 | K | 0.588938049 | Down | Poly(rC)-binding protein 2 OS=Homo sapiens OX=9606 GN=PCBP2 PE=1 SV=1 | PCBP2 | _LLMHGK[Acetyl (K)]EVGSIIGK_ |
| Q15392 | 313 | K | 0.390278208 | Down | Delta(24)-sterol reductase OS=Homo sapiens OX=9606 GN=DHCR24 PE=1 SV=2 | DHCR24 | _HVENYLK[Acetyl (K)]TNR_ |
| Q15393 | 36 | K | 0.612076752 | Down | Splicing factor 3B subunit 3 OS=Homo sapiens OX=9606 GN=SF3B3 PE=1 SV=4 | SF3B3 | _GK[Acetyl (K)]ILELLRPDPNTGK_ |
| Q15393 | 929 | K | 0.342834296 | Down | Splicing factor 3B subunit 3 OS=Homo sapiens OX=9606 GN=SF3B3 PE=1 SV=4 | SF3B3 | _SVAGGFVYTYK[Acetyl (K)]LVNNGEK_ |
| Q15424 | 607 | K | 1.744898417 | Up | Scaffold attachment factor B1 OS=Homo sapiens OX=9606 GN=SAFB PE=1 SV=4 | SAFB | _SVVSFDK[Acetyl (K)]VK_ |
| Q15428 | 194 | K | 0.334385451 | Down | Splicing factor 3A subunit 2 OS=Homo sapiens OX=9606 GN=SF3A2 PE=1 SV=2 | SF3A2 | _AEGK[Acetyl (K)]FWTHWNR_ |
| Q15436 | 81 | K | 0.347622205 | Down | Protein transport protein Sec23A OS=Homo sapiens OX=9606 GN=SEC23A PE=1 SV=2 | SEC23A | _AK[Acetyl (K)]LWAC[Carbamidomethyl (C)]NFC[Carbamidomethyl (C)]YQR_ |
| Q15436 | 392 | K | 0.650982671 | Down | Protein transport protein Sec23A OS=Homo sapiens OX=9606 GN=SEC23A PE=1 SV=2 | SEC23A | _VFTK[Acetyl (K)]DMHGQFK_ |
| Q15437 | 394 | K | 0.580024714 | Down | Protein transport protein Sec23B OS=Homo sapiens OX=9606 GN=SEC23B PE=1 SV=2 | SEC23B | _IFTK[Acetyl (K)]DFNGDFR_ |
| Q15437 | 558 | K | 0.290719828 | Down | Protein transport protein Sec23B OS=Homo sapiens OX=9606 GN=SEC23B PE=1 SV=2 | SEC23B | _LC[Carbamidomethyl (C)]QK[Acetyl (K)]FGQYNK_ |
| Q15459 | 424 | K | 1.6360251 | Up | Splicing factor 3A subunit 1 OS=Homo sapiens OX=9606 GN=SF3A1 PE=1 SV=1 | SF3A1 | _IPASK[Acetyl (K)]MQEHMR_ |
| Q15459 | 756 | K | 2.074433318 | Up | Splicing factor 3A subunit 1 OS=Homo sapiens OX=9606 GN=SF3A1 PE=1 SV=1 | SF3A1 | _QK[Acetyl (K)]LQYEGIFIK_ |
| Q15464 | 218 | K | 2.144294872 | Up | SH2 domain-containing adapter protein B OS=Homo sapiens OX=9606 GN=SHB PE=1 SV=2 | SHB | _TWSPTAC[Carbamidomethyl (C)]GGK[Acetyl (K)]K[Acetyl (K)]LLNK_ |
| Q15464 | 219 | K | 2.144294872 | Up | SH2 domain-containing adapter protein B OS=Homo sapiens OX=9606 GN=SHB PE=1 SV=2 | SHB | _TWSPTAC[Carbamidomethyl (C)]GGK[Acetyl (K)]K[Acetyl (K)]LLNK_ |
| Q15555 | 271 | K | 1.711107649 | Up | Microtubule-associated protein RP/EB family member 2 OS=Homo sapiens OX=9606 GN=MAPRE2 PE=1 SV=1 | MAPRE2 | _DFYFGK[Acetyl (K)]LR_ |
| Q15561 | 378 | K | 1.819859568 | Up | Transcriptional enhancer factor TEF-3 OS=Homo sapiens OX=9606 GN=TEAD4 PE=1 SV=3 | TEAD4 | _LK[Acetyl (K)]HLPEK_ |
| Q15648 | 1126 | K | 0.227009801 | Down | Mediator of RNA polymerase II transcription subunit 1 OS=Homo sapiens OX=9606 GN=MED1 PE=1 SV=4 | MED1 | _SEGSSSSK[Acetyl (K)]LSSSMYSSQGSSGSSQSK_ |
| Q15648 | 1391 | K | 0.473711715 | Down | Mediator of RNA polymerase II transcription subunit 1 OS=Homo sapiens OX=9606 GN=MED1 PE=1 SV=4 | MED1 | _NVGSTGVAK[Acetyl (K)]IIISK_ |
| Q15691 | 66 | K | 0.207335972 | Down | Microtubule-associated protein RP/EB family member 1 OS=Homo sapiens OX=9606 GN=MAPRE1 PE=1 SV=3 | MAPRE1 | _FQAK[Acetyl (K)]LEHEYIQNFK_ |
| Q15691 | 76 | K | 0.539801586 | Down | Microtubule-associated protein RP/EB family member 1 OS=Homo sapiens OX=9606 GN=MAPRE1 PE=1 SV=3 | MAPRE1 | _LEHEYIQNFK[Acetyl (K)]ILQAGFK_ |
| Q15717 | 55 | K | 2.382007105 | Up | ELAV-like protein 1 OS=Homo sapiens OX=9606 GN=ELAVL1 PE=1 SV=2 | ELAVL1 | _DK[Acetyl (K)]VAGHSLGYGFVNYVTAK_ |
| Q15717 | 92 | K | 1.618207929 | Up | ELAV-like protein 1 OS=Homo sapiens OX=9606 GN=ELAVL1 PE=1 SV=2 | ELAVL1 | _TIK[Acetyl (K)]VSYAR_ |
| Q15796 | 420 | K | 1.526360047 | Up | Mothers against decapentaplegic homolog 2 OS=Homo sapiens OX=9606 GN=SMAD2 PE=1 SV=1 | SMAD2 | _MSFVK[Acetyl (K)]GWGAEYR_ |
| Q15811 | 967 | K | 1.873238256 | Up | Intersectin-1 OS=Homo sapiens OX=9606 GN=ITSN1 PE=1 SV=3 | ITSN1 | _SYVK[Acetyl (K)]LISGPIR_ |
| Q15819 | 66 | K | 0.636797775 | Down | Ubiquitin-conjugating enzyme E2 variant 2 OS=Homo sapiens OX=9606 GN=UBE2V2 PE=1 SV=4 | UBE2V2 | _IYSLK[Acetyl (K)]VEC[Carbamidomethyl (C)]GPK_ |
| Q15847 | 70 | K | 0.536045258 | Down | Adipogenesis regulatory factor OS=Homo sapiens OX=9606 GN=ADIRF PE=1 SV=1 | ADIRF | _TANQASDTFSGIGK[Acetyl (K)]K_ |
| Q15942 | 24 | K | 0.385186889 | Down | Zyxin OS=Homo sapiens OX=9606 GN=ZYX PE=1 SV=1 | ZYX | _PSPAISVSVSAPAFYAPQK[Acetyl (K)]K_ |
| Q16181 | 298 | K | 0.593910458 | Down | Septin-7 OS=Homo sapiens OX=9606 GN=SEPTIN7 PE=1 SV=2 | SEPTIN7 | _THMQDLK[Acetyl (K)]DVTNNVHYENYR_ |
| Q16186 | 97 | K | 0.422909706 | Down | Proteasomal ubiquitin receptor ADRM1 OS=Homo sapiens OX=9606 GN=ADRM1 PE=1 SV=2 | ADRM1 | _VYVLK[Acetyl (K)]FK_ |
| Q16512 | 628 | K | 4.129759278 | Up | Serine/threonine-protein kinase N1 OS=Homo sapiens OX=9606 GN=PKN1 PE=1 SV=2 | PKN1 | _GHFGK[Acetyl (K)]VLLSEFR_ |
| Q16531 | 857 | K | 0.227997696 | Down | DNA damage-binding protein 1 OS=Homo sapiens OX=9606 GN=DDB1 PE=1 SV=1 | DDB1 | _IVVFQYSDGK[Acetyl (K)]LQTVAEK_ |
| Q16576 | 4 | K | 0.627257386 | Down | Histone-binding protein RBBP7 OS=Homo sapiens OX=9606 GN=RBBP7 PE=1 SV=1 | RBBP7 | _[Acetyl (Protein N-term)]ASK[Acetyl (K)]EMFEDTVEER_ |
| Q16629 | 70 | K | 0.578853841 | Down | Serine/arginine-rich splicing factor 7 OS=Homo sapiens OX=9606 GN=SRSF7 PE=1 SV=1 | SRSF7 | _GLDGK[Acetyl (K)]VIC[Carbamidomethyl (C)]GSR_ |
| Q16658 | 241 | K | 0.632379064 | Down | Fascin OS=Homo sapiens OX=9606 GN=FSCN1 PE=1 SV=3 | FSCN1 | _YLAPSGPSGTLK[Acetyl (K)]AGK_ |
| Q16658 | 313 | K | 0.613214807 | Down | Fascin OS=Homo sapiens OX=9606 GN=FSCN1 PE=1 SV=3 | FSCN1 | _THTGK[Acetyl (K)]YWTLTATGGVQSTASSK_ |
| Q16822 | 491 | K | 1.582997927 | Up | Phosphoenolpyruvate carboxykinase [GTP], mitochondrial OS=Homo sapiens OX=9606 GN=PCK2 PE=1 SV=4 | PCK2 | _GK[Acetyl (K)]IIMHDPFAMR_ |
| Q16836 | 76 | K | 0.164564036 | Down | Hydroxyacyl-coenzyme A dehydrogenase, mitochondrial OS=Homo sapiens OX=9606 GN=HADH PE=1 SV=3 | HADH | _KGIEESLRK[Acetyl (K)]VAK[Acetyl (K)]K_ |
| Q16836 | 79 | K | 0.164564036 | Down | Hydroxyacyl-coenzyme A dehydrogenase, mitochondrial OS=Homo sapiens OX=9606 GN=HADH PE=1 SV=3 | HADH | _KGIEESLRK[Acetyl (K)]VAK[Acetyl (K)]K_ |
| Q16851 | 177 | K | 3.996789335 | Up | UTP--glucose-1-phosphate uridylyltransferase OS=Homo sapiens OX=9606 GN=UGP2 PE=1 SV=5 | UGP2 | _ILQK[Acetyl (K)]YNHC[Carbamidomethyl (C)]R_ |
| Q16851 | 301 | K | 1.667715295 | Up | UTP--glucose-1-phosphate uridylyltransferase OS=Homo sapiens OX=9606 GN=UGP2 PE=1 SV=5 | UGP2 | _GGTLTQYEGK[Acetyl (K)]LR_ |
| Q16854 | 56 | K | 1.531938352 | Up | Deoxyguanosine kinase, mitochondrial OS=Homo sapiens OX=9606 GN=DGUOK PE=1 SV=2 | DGUOK | _STFVK[Acetyl (K)]LLTK_ |
| Q16891 | 640 | K | 0.614148042 | Down | MICOS complex subunit MIC60 OS=Homo sapiens OX=9606 GN=IMMT PE=1 SV=1 | IMMT | _FYAVQK[Acetyl (K)]LAR_ |
| Q1KMD3 | 297 | K | 0.60934819 | Down | Heterogeneous nuclear ribonucleoprotein U-like protein 2 OS=Homo sapiens OX=9606 GN=HNRNPUL2 PE=1 SV=1 | HNRNPUL2 | _VC[Carbamidomethyl (C)]FEAK[Acetyl (K)]VTQNLPMK_ |
| Q29980 | 190 | K | 0.651146739 | Down | MHC class I polypeptide-related sequence B OS=Homo sapiens OX=9606 GN=MICB PE=1 SV=2 | MICB | _AMQADC[Carbamidomethyl (C)]LQK[Acetyl (K)]LQR_ |
| Q2TAK8 | 642 | K | 1.671148686 | Up | PWWP domain-containing DNA repair factor 3A OS=Homo sapiens OX=9606 GN=PWWP3A PE=1 SV=3 | PWWP3A | _YLQGVYQEVGAK[Acetyl (K)]VLQR_ |
| Q2TAY7 | 292 | K | 0.469490324 | Down | WD40 repeat-containing protein SMU1 OS=Homo sapiens OX=9606 GN=SMU1 PE=1 SV=2 | SMU1 | _VWK[Acetyl (K)]IQSGQC[Carbamidomethyl (C)]LR_ |
| Q2TAY7 | 379 | K | 0.463105897 | Down | WD40 repeat-containing protein SMU1 OS=Homo sapiens OX=9606 GN=SMU1 PE=1 SV=2 | SMU1 | _IWNMK[Acetyl (K)]TTEC[Carbamidomethyl (C)]SNTFK_ |
| Q49A26 | 15 | K | 1.729450659 | Up | Cytokine-like nuclear factor N-PAC OS=Homo sapiens OX=9606 GN=GLYR1 PE=1 SV=4 | GLYR1 | _LGDLVWGK[Acetyl (K)]LGR_ |
| Q49A26 | 25 | K | 2.491648457 | Up | Cytokine-like nuclear factor N-PAC OS=Homo sapiens OX=9606 GN=GLYR1 PE=1 SV=4 | GLYR1 | _YPPWPGK[Acetyl (K)]IVNPPK_ |
| Q49A26 | 237 | K | 1.972439576 | Up | Cytokine-like nuclear factor N-PAC OS=Homo sapiens OX=9606 GN=GLYR1 PE=1 SV=4 | GLYR1 | _PAVC[Carbamidomethyl (C)]YQAITK[Acetyl (K)]K_ |
| Q4ADV7 | 503 | K | 2.613531402 | Up | Guanine nucleotide exchange factor subunit RIC1 OS=Homo sapiens OX=9606 GN=RIC1 PE=1 SV=2 | RIC1 | _FSAIDK[Acetyl (K)]LGQNIAVVGK_ |
| Q4LE39 | 349 | K | 0.539438175 | Down | AT-rich interactive domain-containing protein 4B OS=Homo sapiens OX=9606 GN=ARID4B PE=1 SV=2 | ARID4B | _LVHK[Acetyl (K)]LGGFDNIESGAVWK_ |
| Q53EP0 | 440 | K | 0.303500941 | Down | Fibronectin type III domain-containing protein 3B OS=Homo sapiens OX=9606 GN=FNDC3B PE=1 SV=2 | FNDC3B | _LTK[Acetyl (K)]LC[Carbamidomethyl (C)]PAMGYTFR_ |
| Q53F19 | 282 | K | 0.532891097 | Down | Nuclear cap-binding protein subunit 3 OS=Homo sapiens OX=9606 GN=NCBP3 PE=1 SV=2 | NCBP3 | _SQYYMK[Acetyl (K)]YGNPNYGGMK_ |
| Q53R41 | 482 | K | 0.398131089 | Down | FAST kinase domain-containing protein 1, mitochondrial OS=Homo sapiens OX=9606 GN=FASTKD1 PE=1 SV=1 | FASTKD1 | _LLQK[Acetyl (K)]LDHYGR_ |
| Q5BJF2 | 48 | K | 0.534660788 | Down | Sigma intracellular receptor 2 OS=Homo sapiens OX=9606 GN=TMEM97 PE=1 SV=1 | TMEM97 | _NLLK[Acetyl (K)]WYAK_ |
| Q5BKZ1 | 264 | K | 1.541873819 | Up | DBIRD complex subunit ZNF326 OS=Homo sapiens OX=9606 GN=ZNF326 PE=1 SV=2 | ZNF326 | _PMEK[Acetyl (K)]ISLSK_ |
| Q5C9Z4 | 641 | K | 1.975585079 | Up | Nucleolar MIF4G domain-containing protein 1 OS=Homo sapiens OX=9606 GN=NOM1 PE=1 SV=1 | NOM1 | _QLVGTVSSK[Acetyl (K)]ILELAR_ |
| Q5GLZ8 | 606 | K | 0.573555369 | Down | Probable E3 ubiquitin-protein ligase HERC4 OS=Homo sapiens OX=9606 GN=HERC4 PE=1 SV=1 | HERC4 | _VNEK[Acetyl (K)]MGQIIQYDK_ |
| Q5H9R7 | 430 | K | 0.564476943 | Down | Serine/threonine-protein phosphatase 6 regulatory subunit 3 OS=Homo sapiens OX=9606 GN=PPP6R3 PE=1 SV=2 | PPP6R3 | _HLFQK[Acetyl (K)]C[Carbamidomethyl (C)]QLIER_ |
| Q5HYI8 | 202 | K | 1.586272489 | Up | Rab-like protein 3 OS=Homo sapiens OX=9606 GN=RABL3 PE=1 SV=1 | RABL3 | _FFDK[Acetyl (K)]VIEK_ |
| Q5JRA6 | 1625 | K | 0.470758558 | Down | Transport and Golgi organization protein 1 homolog OS=Homo sapiens OX=9606 GN=MIA3 PE=1 SV=1 | MIA3 | _HK[Acetyl (K)]LLELTQK_ |
| Q5JSZ5 | 50 | K | 0.555186861 | Down | Protein PRRC2B OS=Homo sapiens OX=9606 GN=PRRC2B PE=1 SV=2 | PRRC2B | _HGLQSLGK[Acetyl (K)]VAAAR_ |
| Q5JTH9 | 14 | K | 0.374597182 | Down | RRP12-like protein OS=Homo sapiens OX=9606 GN=RRP12 PE=1 SV=2 | RRP12 | _LPSGVSAK[Acetyl (K)]LK_ |
| Q5JTV8 | 578 | K | 0.568276116 | Down | Torsin-1A-interacting protein 1 OS=Homo sapiens OX=9606 GN=TOR1AIP1 PE=1 SV=2 | TOR1AIP1 | _ISHLVLPVQPENALK[Acetyl (K)]R_ |
| Q5JVF3 | 201 | K | 0.503117546 | Down | PCI domain-containing protein 2 OS=Homo sapiens OX=9606 GN=PCID2 PE=1 SV=2 | PCID2 | _AIDSSNLK[Acetyl (K)]DDYSTAQR_ |
| Q5SNT2 | 176 | K | 4.477403431 | Up | Transmembrane protein 201 OS=Homo sapiens OX=9606 GN=TMEM201 PE=1 SV=1 | TMEM201 | _LC[Carbamidomethyl (C)]RPC[Carbamidomethyl (C)]QAAVEYYIK[Acetyl (K)]HQNR_ |
| Q5ST30 | 748 | K | 2.716101874 | Up | Valine--tRNA ligase, mitochondrial OS=Homo sapiens OX=9606 GN=VARS2 PE=1 SV=2 | VARS2 | _HFC[Carbamidomethyl (C)]NK[Acetyl (K)]IWNALR_ |
| Q5SW79 | 747 | K | 1.539107245 | Up | Centrosomal protein of 170 kDa OS=Homo sapiens OX=9606 GN=CEP170 PE=1 SV=1 | CEP170 | _QTLAK[Acetyl (K)]LQQQEQR_ |
| Q5SWX8 | 238 | K | 0.439013477 | Down | Protein odr-4 homolog OS=Homo sapiens OX=9606 GN=ODR4 PE=1 SV=1 | ODR4 | _DEDC[Carbamidomethyl (C)]DLLEGQK[Acetyl (K)]K_ |
| Q5T200 | 47 | K | 0.633764696 | Down | Zinc finger CCCH domain-containing protein 13 OS=Homo sapiens OX=9606 GN=ZC3H13 PE=1 SV=1 | ZC3H13 | _NWLK[Acetyl (K)]TGNC[Carbamidomethyl (C)]LYGNTC[Carbamidomethyl (C)]R_ |
| Q5T2T1 | 56 | K | 0.657790134 | Down | MAGUK p55 subfamily member 7 OS=Homo sapiens OX=9606 GN=MPP7 PE=1 SV=1 | MPP7 | _IHEK[Acetyl (K)]LHYYEK_ |
| Q5T3I0 | 111 | K | 3.089181849 | Up | G patch domain-containing protein 4 OS=Homo sapiens OX=9606 GN=GPATCH4 PE=1 SV=2 | GPATCH4 | _FVK[Acetyl (K)]MATLTSGGEKPNK_ |
| Q5T3J3 | 134 | K | 2.210842601 | Up | Ligand-dependent nuclear receptor-interacting factor 1 OS=Homo sapiens OX=9606 GN=LRIF1 PE=1 SV=1 | LRIF1 | _VTSVGTGNFSSSVSK[Acetyl (K)]VQSHGVK_ |
| Q5T3J3 | 316 | K | 2.108114817 | Up | Ligand-dependent nuclear receptor-interacting factor 1 OS=Homo sapiens OX=9606 GN=LRIF1 PE=1 SV=1 | LRIF1 | _SSNNVASK[Acetyl (K)]ILK_ |
| Q5T5C0 | 924 | K | 0.527894229 | Down | Syntaxin-binding protein 5 OS=Homo sapiens OX=9606 GN=STXBP5 PE=1 SV=1 | STXBP5 | _QAK[Acetyl (K)]VISLPTQNC[Carbamidomethyl (C)]AYK_ |
| Q5T8P6 | 41 | K | 0.60514725 | Down | RNA-binding protein 26 OS=Homo sapiens OX=9606 GN=RBM26 PE=1 SV=3 | RBM26 | _YVLALVK[Acetyl (K)]K_ |
| Q5T8P6 | 600 | K | 1.75084353 | Up | RNA-binding protein 26 OS=Homo sapiens OX=9606 GN=RBM26 PE=1 SV=3 | RBM26 | _FIK[Acetyl (K)]VYWHR_ |
| Q5T9L3 | 73 | K | 0.40811528 | Down | Protein wntless homolog OS=Homo sapiens OX=9606 GN=WLS PE=1 SV=2 | WLS | _WFVPWGPNHC[Carbamidomethyl (C)]DK[Acetyl (K)]IR_ |
| Q5TA45 | 362 | K | 0.487016019 | Down | Integrator complex subunit 11 OS=Homo sapiens OX=9606 GN=INTS11 PE=1 SV=2 | INTS11 | _NMVIMPGYC[Carbamidomethyl (C)]VQGTVGHK[Acetyl (K)]ILSGQR_ |
| Q5TGY3 | 494 | K | 0.604431798 | Down | Transcription factor Gibbin OS=Homo sapiens OX=9606 GN=AHDC1 PE=1 SV=1 | AHDC1 | _TTYK[Acetyl (K)]VSSLSSSLSVEGK_ |
| Q5TH69 | 2024 | K | 0.606343868 | Down | Brefeldin A-inhibited guanine nucleotide-exchange protein 3 OS=Homo sapiens OX=9606 GN=ARFGEF3 PE=1 SV=3 | ARFGEF3 | _TISK[Acetyl (K)]LMTEYK_ |
| Q5VUA4 | 1866 | K | 1.515962386 | Up | Zinc finger protein 318 OS=Homo sapiens OX=9606 GN=ZNF318 PE=1 SV=2 | ZNF318 | _AK[Acetyl (K)]LDSFLSEAR_ |
| Q5VWN6 | 694 | K | 0.619243727 | Down | Protein TASOR 2 OS=Homo sapiens OX=9606 GN=TASOR2 PE=1 SV=1 | TASOR2 | _SPEAATPVGK[Acetyl (K)]VMPFR_ |
| Q5VYK3 | 1442 | K | 1.567627543 | Up | Proteasome adapter and scaffold protein ECM29 OS=Homo sapiens OX=9606 GN=ECPAS PE=1 SV=2 | ECPAS | _LLQK[Acetyl (K)]LNGWYMEK_ |
| Q5VYS8 | 1156 | K | 0.518883628 | Down | Terminal uridylyltransferase 7 OS=Homo sapiens OX=9606 GN=TUT7 PE=1 SV=1 | TUT7 | _VFTK[Acetyl (K)]MC[Carbamidomethyl (C)]DIGDASR_ |
| Q5VZK9 | 1302 | K | 1.529820054 | Up | F-actin-uncapping protein LRRC16A OS=Homo sapiens OX=9606 GN=CARMIL1 PE=1 SV=1 | CARMIL1 | _VALLPPVLK[Acetyl (K)]K_ |
| Q5VZL5 | 757 | K | 1.524010444 | Up | Zinc finger MYM-type protein 4 OS=Homo sapiens OX=9606 GN=ZMYM4 PE=1 SV=1 | ZMYM4 | _SFC[Carbamidomethyl (C)]SEGC[Carbamidomethyl (C)]K[Acetyl (K)]LLYK_ |
| Q63HN8 | 4297 | K | 2.878808992 | Up | E3 ubiquitin-protein ligase RNF213 OS=Homo sapiens OX=9606 GN=RNF213 PE=1 SV=3 | RNF213 | _GMEFVQGLSK[Acetyl (K)]PGRPHQWVFPK_ |
| Q66K74 | 277 | K | 0.273942192 | Down | Microtubule-associated protein 1S OS=Homo sapiens OX=9606 GN=MAP1S PE=1 SV=2 | MAP1S | _SSFWK[Acetyl (K)]LVR_ |
| Q68CP9 | 1076 | K | 0.543692441 | Down | AT-rich interactive domain-containing protein 2 OS=Homo sapiens OX=9606 GN=ARID2 PE=1 SV=2 | ARID2 | _RGPSTPGGK[Acetyl (K)]LILPAPQIPPPNNAR_ |
| Q6DD87 | 80 | K | 1.989739127 | Up | Zinc finger protein 787 OS=Homo sapiens OX=9606 GN=ZNF787 PE=1 SV=4 | ZNF787 | _SFSHWSK[Acetyl (K)]LTR_ |
| Q6DKI1 | 138 | K | 0.229718642 | Down | Ribosomal protein uL30-like OS=Homo sapiens OX=9606 GN=RPL7L1 PE=1 SV=2 | RPL7L1 | _VTPQNLK[Acetyl (K)]MLR_ |
| Q6H8Q1 | 185 | K | 0.493496328 | Down | Actin-binding LIM protein 2 OS=Homo sapiens OX=9606 GN=ABLIM2 PE=1 SV=2 | ABLIM2 | _SC[Carbamidomethyl (C)]GK[Acetyl (K)]LLNAEYISK_ |
| Q6I9Y2 | 98 | K | 1.609963432 | Up | THO complex subunit 7 homolog OS=Homo sapiens OX=9606 GN=THOC7 PE=1 SV=3 | THOC7 | _EIEC[Carbamidomethyl (C)]SIAGAHEK[Acetyl (K)]IAEC[Carbamidomethyl (C)]K_ |
| Q6IBS0 | 19 | K | 1.639290975 | Up | Twinfilin-2 OS=Homo sapiens OX=9606 GN=TWF2 PE=1 SV=2 | TWF2 | _EFFAK[Acetyl (K)]AR_ |
| Q6IE81 | 302 | K | 1.503122545 | Up | Protein Jade-1 OS=Homo sapiens OX=9606 GN=JADE1 PE=1 SV=1 | JADE1 | _MEPITK[Acetyl (K)]VSHIPSSR_ |
| Q6IE81 | 521 | K | 2.9792685 | Up | Protein Jade-1 OS=Homo sapiens OX=9606 GN=JADE1 PE=1 SV=1 | JADE1 | _SVC[Carbamidomethyl (C)]K[Acetyl (K)]VQEQIFNLYTK_ |
| Q6IQ32 | 83 | K | 3.807627418 | Up | Activity-dependent neuroprotector homeobox protein 2 OS=Homo sapiens OX=9606 GN=ADNP2 PE=1 SV=1 | ADNP2 | _YSTK[Acetyl (K)]VLTSFK_ |
| Q6KC79 | 2787 | K | 1.738835935 | Up | Nipped-B-like protein OS=Homo sapiens OX=9606 GN=NIPBL PE=1 SV=2 | NIPBL | _LTNK[Acetyl (K)]VVQTLR_ |
| Q6MZP7 | 249 | K | 2.682541976 | Up | Protein lin-54 homolog OS=Homo sapiens OX=9606 GN=LIN54 PE=1 SV=3 | LIN54 | _LIFAK[Acetyl (K)]PINSK_ |
| Q6NSJ5 | 487 | K | 1.650114004 | Up | Volume-regulated anion channel subunit LRRC8E OS=Homo sapiens OX=9606 GN=LRRC8E PE=1 SV=2 | LRRC8E | _DHLK[Acetyl (K)]VMR_ |
| Q6NTE8 | 58 | K | 4.108714018 | Up | MRN complex-interacting protein OS=Homo sapiens OX=9606 GN=MRNIP PE=1 SV=2 | MRNIP | _HVQK[Acetyl (K)]LNLLQGQVSELPLR_ |
| Q6NUK1 | 332 | K | 0.523230178 | Down | Mitochondrial adenyl nucleotide antiporter SLC25A24 OS=Homo sapiens OX=9606 GN=SLC25A24 PE=1 SV=2 | SLC25A24 | _TGQYSGIYDC[Carbamidomethyl (C)]AK[Acetyl (K)]K_ |
| Q6NUQ1 | 728 | K | 16.31476185 | Up | RAD50-interacting protein 1 OS=Homo sapiens OX=9606 GN=RINT1 PE=1 SV=1 | RINT1 | _RPENYFK[Acetyl (K)]HIK_ |
| Q6NZI2 | 109 | K | 2.143865704 | Up | Caveolae-associated protein 1 OS=Homo sapiens OX=9606 GN=CAVIN1 PE=1 SV=1 | CAVIN1 | _AHATTSNTVSK[Acetyl (K)]LLEK_ |
| Q6NZI2 | 165 | K | 1.807484249 | Up | Caveolae-associated protein 1 OS=Homo sapiens OX=9606 GN=CAVIN1 PE=1 SV=1 | CAVIN1 | _LPAK[Acetyl (K)]LSISK_ |
| Q6P1A2 | 232 | K | 0.607283338 | Down | Lysophospholipid acyltransferase 5 OS=Homo sapiens OX=9606 GN=LPCAT3 PE=1 SV=1 | LPCAT3 | _IPNSIIPALK[Acetyl (K)]R_ |
| Q6P1M3 | 834 | K | 0.656718065 | Down | LLGL scribble cell polarity complex component 2 OS=Homo sapiens OX=9606 GN=LLGL2 PE=1 SV=2 | LLGL2 | _LK[Acetyl (K)]LTALEGSR_ |
| Q6P2E9 | 1087 | K | 2.2437918 | Up | Enhancer of mRNA-decapping protein 4 OS=Homo sapiens OX=9606 GN=EDC4 PE=1 SV=1 | EDC4 | _ENISK[Acetyl (K)]LLK_ |
| Q6P2Q9 | 746 | K | 0.587006736 | Down | Pre-mRNA-processing-splicing factor 8 OS=Homo sapiens OX=9606 GN=PRPF8 PE=1 SV=2 | PRPF8 | _AK[Acetyl (K)]ADWWTNTAHYNR_ |
| Q6P2Q9 | 1158 | K | 0.505608033 | Down | Pre-mRNA-processing-splicing factor 8 OS=Homo sapiens OX=9606 GN=PRPF8 PE=1 SV=2 | PRPF8 | _AVFWDIK[Acetyl (K)]NR_ |
| Q6P2Q9 | 1392 | K | 0.501702455 | Down | Pre-mRNA-processing-splicing factor 8 OS=Homo sapiens OX=9606 GN=PRPF8 PE=1 SV=2 | PRPF8 | _VWAEYALK[Acetyl (K)]R_ |
| Q6P2Q9 | 1831 | K | 3.182240502 | Up | Pre-mRNA-processing-splicing factor 8 OS=Homo sapiens OX=9606 GN=PRPF8 PE=1 SV=2 | PRPF8 | _IIHTSVWAGQK[Acetyl (K)]R_ |
| Q6P4A7 | 197 | K | 2.11517716 | Up | Sideroflexin-4 OS=Homo sapiens OX=9606 GN=SFXN4 PE=1 SV=1 | SFXN4 | _YGLTGPWIK[Acetyl (K)]R_ |
| Q6P587 | 151 | K | 0.343476236 | Down | Acylpyruvase FAHD1, mitochondrial OS=Homo sapiens OX=9606 GN=FAHD1 PE=1 SV=2 | FAHD1 | _LWLK[Acetyl (K)]VNGELR_ |
| Q6P5R6 | 63 | K | 3.206788022 | Up | Ribosomal protein eL22-like OS=Homo sapiens OX=9606 GN=RPL22L1 PE=1 SV=2 | RPL22L1 | _NK[Acetyl (K)]ITVVSEK_ |
| Q6PCB5 | 28 | K | 0.477026096 | Down | Lysine-specific demethylase RSBN1L OS=Homo sapiens OX=9606 GN=RSBN1L PE=1 SV=2 | RSBN1L | _EPFGK[Acetyl (K)]LQLSSR_ |
| Q6PI48 | 454 | K | 1.531521782 | Up | Aspartate--tRNA ligase, mitochondrial OS=Homo sapiens OX=9606 GN=DARS2 PE=1 SV=1 | DARS2 | _AC[Carbamidomethyl (C)]SLLGK[Acetyl (K)]LR_ |
| Q6PIW4 | 339 | K | 0.416893269 | Down | Fidgetin-like protein 1 OS=Homo sapiens OX=9606 GN=FIGNL1 PE=1 SV=2 | FIGNL1 | _GILGK[Acetyl (K)]FVPPIPK_ |
| Q6PIY7 | 312 | K | 1.684879596 | Up | Poly(A) RNA polymerase GLD2 OS=Homo sapiens OX=9606 GN=TENT2 PE=1 SV=1 | TENT2 | _VRPLVLVIK[Acetyl (K)]K_ |
| Q6PKG0 | 964 | K | 2.026965649 | Up | La-related protein 1 OS=Homo sapiens OX=9606 GN=LARP1 PE=1 SV=2 | LARP1 | _YYSYGLEK[Acetyl (K)]K_ |
| Q6PML9 | 124 | K | 0.232225026 | Down | Proton-coupled zinc antiporter SLC30A9, mitochondrial OS=Homo sapiens OX=9606 GN=SLC30A9 PE=1 SV=1 | SLC30A9 | _EYGSK[Acetyl (K)]YTQNNFITGVR_ |
| Q6UW63 | 202 | K | 1.66077834 | Up | Protein O-glucosyltransferase 2 OS=Homo sapiens OX=9606 GN=POGLUT2 PE=1 SV=1 | POGLUT2 | _VYIK[Acetyl (K)]THGEHVGFR_ |
| Q6UW68 | 149 | K | 0.65274407 | Down | Transmembrane protein 205 OS=Homo sapiens OX=9606 GN=TMEM205 PE=1 SV=1 | TMEM205 | _EKDPK[Acetyl (K)]YSALR_ |
| Q6UXN9 | 2 | K | 0.46414564 | Down | WD repeat-containing protein 82 OS=Homo sapiens OX=9606 GN=WDR82 PE=1 SV=1 | WDR82 | _MK[Acetyl (K)]LTDSVLR_ |
| Q6XZF7 | 661 | K | 0.374370954 | Down | Dynamin-binding protein OS=Homo sapiens OX=9606 GN=DNMBP PE=1 SV=1 | DNMBP | _TNAVSPK[Acetyl (K)]LLSR_ |
| Q6Y7W6 | 1123 | K | 0.486690433 | Down | GRB10-interacting GYF protein 2 OS=Homo sapiens OX=9606 GN=GIGYF2 PE=1 SV=1 | GIGYF2 | _LLK[Acetyl (K)]LFQGVNK_ |
| Q6ZNB6 | 740 | K | 1.520925268 | Up | NF-X1-type zinc finger protein NFXL1 OS=Homo sapiens OX=9606 GN=NFXL1 PE=1 SV=2 | NFXL1 | _C[Carbamidomethyl (C)]HC[Carbamidomethyl (C)]K[Acetyl (K)]ITSLYVEC[Carbamidomethyl (C)]R_ |
| Q6ZRP7 | 150 | K | 0.62315748 | Down | Sulfhydryl oxidase 2 OS=Homo sapiens OX=9606 GN=QSOX2 PE=1 SV=3 | QSOX2 | _AFTK[Acetyl (K)]EFTTGENFK_ |
| Q6ZRP7 | 414 | K | 0.607740147 | Down | Sulfhydryl oxidase 2 OS=Homo sapiens OX=9606 GN=QSOX2 PE=1 SV=3 | QSOX2 | _ISGIFLTNHIK[Acetyl (K)]WVGC[Carbamidomethyl (C)]QGSR_ |
| Q6ZSZ5 | 1058 | K | 0.560305753 | Down | Rho guanine nucleotide exchange factor 18 OS=Homo sapiens OX=9606 GN=ARHGEF18 PE=1 SV=4 | ARHGEF18 | _AALEK[Acetyl (K)]LQSQLR_ |
| Q6ZSZ5 | 1238 | K | 0.368334863 | Down | Rho guanine nucleotide exchange factor 18 OS=Homo sapiens OX=9606 GN=ARHGEF18 PE=1 SV=4 | ARHGEF18 | _QAAVQQQIPTK[Acetyl (K)]LAASTK_ |
| Q6ZU65 | 1074 | K | 0.582308843 | Down | Ubinuclein-2 OS=Homo sapiens OX=9606 GN=UBN2 PE=1 SV=2 | UBN2 | _PSVSTK[Acetyl (K)]LISK_ |
| Q6ZU65 | 1148 | K | 0.529837687 | Down | Ubinuclein-2 OS=Homo sapiens OX=9606 GN=UBN2 PE=1 SV=2 | UBN2 | _NLQAPSK[Acetyl (K)]LTNSSSTGTVGK_ |
| Q7KZ85 | 748 | K | 0.54386507 | Down | Transcription elongation factor SPT6 OS=Homo sapiens OX=9606 GN=SUPT6H PE=1 SV=2 | SUPT6H | _K[Acetyl (K)]LYNWLR_ |
| Q7KZI7 | 637 | K | 0.450103978 | Down | Serine/threonine-protein kinase MARK2 OS=Homo sapiens OX=9606 GN=MARK2 PE=1 SV=2 | MARK2 | _GASGSIFSK[Acetyl (K)]FTSK_ |
| Q7L014 | 907 | K | 4.782585217 | Up | Probable ATP-dependent RNA helicase DDX46 OS=Homo sapiens OX=9606 GN=DDX46 PE=1 SV=2 | DDX46 | _INAK[Acetyl (K)]LNYVPLEK_ |
| Q7L0Y3 | 268 | K | 1.629341416 | Up | tRNA methyltransferase 10 homolog C OS=Homo sapiens OX=9606 GN=TRMT10C PE=1 SV=2 | TRMT10C | _WDK[Acetyl (K)]LLLTSTEK_ |
| Q7L1W4 | 632 | K | 3.202819616 | Up | Volume-regulated anion channel subunit LRRC8D OS=Homo sapiens OX=9606 GN=LRRC8D PE=1 SV=1 | LRRC8D | _LLVLNSLK[Acetyl (K)]K_ |
| Q7L2E3 | 73 | K | 0.290884998 | Down | ATP-dependent RNA helicase DHX30 OS=Homo sapiens OX=9606 GN=DHX30 PE=1 SV=1 | DHX30 | _DK[Acetyl (K)]LVYVHTNGPK_ |
| Q7L2E3 | 762 | K | 0.41169075 | Down | ATP-dependent RNA helicase DHX30 OS=Homo sapiens OX=9606 GN=DHX30 PE=1 SV=1 | DHX30 | _TK[Acetyl (K)]VSC[Carbamidomethyl (C)]LETVWVSR_ |
| Q7L4E1 | 375 | K | 0.649748984 | Down | Mitoguardin 2 OS=Homo sapiens OX=9606 GN=MIGA2 PE=1 SV=1 | MIGA2 | _SNQLFFGK[Acetyl (K)]VGR_ |
| Q7L4I2 | 342 | K | 2.281460854 | Up | Arginine/serine-rich coiled-coil protein 2 OS=Homo sapiens OX=9606 GN=RSRC2 PE=1 SV=1 | RSRC2 | _MLWQGK[Acetyl (K)]K_ |
| Q7L576 | 426 | K | 0.09585292 | Down | Cytoplasmic FMR1-interacting protein 1 OS=Homo sapiens OX=9606 GN=CYFIP1 PE=1 SV=1 | CYFIP1 | _YSNK[Acetyl (K)]DC[Carbamidomethyl (C)]PDSAEEYER_ |
| Q7L5Y9 | 144 | K | 0.548065476 | Down | E3 ubiquitin-protein transferase MAEA OS=Homo sapiens OX=9606 GN=MAEA PE=1 SV=1 | MAEA | _C[Carbamidomethyl (C)]GYYNTAVK[Acetyl (K)]LAR_ |
| Q7RTS9 | 213 | K | 2.078697381 | Up | Dymeclin OS=Homo sapiens OX=9606 GN=DYM PE=1 SV=1 | DYM | _GPC[Carbamidomethyl (C)]LPYTSK[Acetyl (K)]LVK_ |
| Q7Z309 | 165 | K | 0.464900632 | Down | PABIR family member 2 OS=Homo sapiens OX=9606 GN=PABIR2 PE=1 SV=2 | PABIR2 | _PSVLGPLK[Acetyl (K)]R_ |
| Q7Z3B3 | 331 | K | 5.025480703 | Up | KAT8 regulatory NSL complex subunit 1 OS=Homo sapiens OX=9606 GN=KANSL1 PE=1 SV=3 | KANSL1 | _TLSK[Acetyl (K)]LPNLESLRPR_ |
| Q7Z3B3 | 785 | K | 1.858779349 | Up | KAT8 regulatory NSL complex subunit 1 OS=Homo sapiens OX=9606 GN=KANSL1 PE=1 SV=3 | KANSL1 | _LLNPPPPVHDPNHSK[Acetyl (K)]MR_ |
| Q7Z3B4 | 371 | K | 0.342528252 | Down | Nucleoporin p54 OS=Homo sapiens OX=9606 GN=NUP54 PE=1 SV=2 | NUP54 | _NQTTSVAK[Acetyl (K)]IAQYK_ |
| Q7Z417 | 361 | K | 0.349900689 | Down | FMR1-interacting protein NUFIP2 OS=Homo sapiens OX=9606 GN=NUFIP2 PE=1 SV=1 | NUFIP2 | _ISYASK[Acetyl (K)]VK_ |
| Q7Z478 | 401 | K | 0.393747591 | Down | ATP-dependent RNA helicase DHX29 OS=Homo sapiens OX=9606 GN=DHX29 PE=1 SV=2 | DHX29 | _SPNPSFEK[Acetyl (K)]VPVGR_ |
| Q7Z4V5 | 163 | K | 2.006850904 | Up | Hepatoma-derived growth factor-related protein 2 OS=Homo sapiens OX=9606 GN=HDGFL2 PE=1 SV=1 | HDGFL2 | _TPALK[Acetyl (K)]MSVSK_ |
| Q7Z589 | 650 | K | 0.61973764 | Down | BRCA2-interacting transcriptional repressor EMSY OS=Homo sapiens OX=9606 GN=EMSY PE=1 SV=2 | EMSY | _GIGSTVQPAAK[Acetyl (K)]IIPTK_ |
| Q7Z5J4 | 1328 | K | 2.304854488 | Up | Retinoic acid-induced protein 1 OS=Homo sapiens OX=9606 GN=RAI1 PE=1 SV=2 | RAI1 | _GLK[Acetyl (K)]LEAIVQK_ |
| Q7Z6E9 | 901 | K | 0.554007726 | Down | E3 ubiquitin-protein ligase RBBP6 OS=Homo sapiens OX=9606 GN=RBBP6 PE=1 SV=1 | RBBP6 | _NIGSNYPEK[Acetyl (K)]LSAR_ |
| Q7Z6E9 | 1199 | K | 2.038761351 | Up | E3 ubiquitin-protein ligase RBBP6 OS=Homo sapiens OX=9606 GN=RBBP6 PE=1 SV=1 | RBBP6 | _DK[Acetyl (K)]ISLSAPAK[Acetyl (K)]K_ |
| Q7Z6E9 | 1207 | K | 2.066143594 | Up | E3 ubiquitin-protein ligase RBBP6 OS=Homo sapiens OX=9606 GN=RBBP6 PE=1 SV=1 | RBBP6 | _ISLSAPAK[Acetyl (K)]K_ |
| Q7Z6M1 | 49 | K | 0.637035979 | Down | Rab9 effector protein with kelch motifs OS=Homo sapiens OX=9606 GN=RABEPK PE=1 SV=1 | RABEPK | _GK[Acetyl (K)]VFIVGGANPNR_ |
| Q7Z739 | 446 | K | 0.662953428 | Down | YTH domain-containing family protein 3 OS=Homo sapiens OX=9606 GN=YTHDF3 PE=1 SV=1 | YTHDF3 | _YSIWC[Carbamidomethyl (C)]STEHGNK[Acetyl (K)]R_ |
| Q7Z739 | 509 | K | 0.59027572 | Down | YTH domain-containing family protein 3 OS=Homo sapiens OX=9606 GN=YTHDF3 PE=1 SV=1 | YTHDF3 | _WIFVK[Acetyl (K)]DVPNNQLR_ |
| Q86TG7 | 136 | K | 0.511477561 | Down | Retrotransposon-derived protein PEG10 OS=Homo sapiens OX=9606 GN=PEG10 PE=1 SV=2 | PEG10 | _WASAK[Acetyl (K)]LER_ |
| Q86TI2 | 318 | K | 0.641554987 | Down | Dipeptidyl peptidase 9 OS=Homo sapiens OX=9606 GN=DPP9 PE=1 SV=3 | DPP9 | _IALK[Acetyl (K)]LAEFQTDSQGK_ |
| Q86TI2 | 456 | K | 0.498358864 | Down | Dipeptidyl peptidase 9 OS=Homo sapiens OX=9606 GN=DPP9 PE=1 SV=3 | DPP9 | _TGFC[Carbamidomethyl (C)]HLYK[Acetyl (K)]VTAVLK_ |
| Q86U42 | 207 | K | 3.151228789 | Up | Polyadenylate-binding protein 2 OS=Homo sapiens OX=9606 GN=PABPN1 PE=1 SV=3 | PABPN1 | _VTILC[Carbamidomethyl (C)]DK[Acetyl (K)]FSGHPK_ |
| Q86U70 | 277 | K | 0.529657389 | Down | LIM domain-binding protein 1 OS=Homo sapiens OX=9606 GN=LDB1 PE=1 SV=2 | LDB1 | _TC[Carbamidomethyl (C)]LFQK[Acetyl (K)]WQR_ |
| Q86UT6 | 431 | K | 1.528753576 | Up | NLR family member X1 OS=Homo sapiens OX=9606 GN=NLRX1 PE=1 SV=1 | NLRX1 | _TMGK[Acetyl (K)]LAYEGVSSR_ |
| Q86UU0 | 108 | K | 2.460539914 | Up | B-cell CLL/lymphoma 9-like protein OS=Homo sapiens OX=9606 GN=BCL9L PE=1 SV=1 | BCL9L | _NPQAGVPPFSSLK[Acetyl (K)]GK[Acetyl (K)]VK_ |
| Q86UU0 | 110 | K | 2.460539914 | Up | B-cell CLL/lymphoma 9-like protein OS=Homo sapiens OX=9606 GN=BCL9L PE=1 SV=1 | BCL9L | _NPQAGVPPFSSLK[Acetyl (K)]GK[Acetyl (K)]VK_ |
| Q86VP6 | 971 | K | 0.597876748 | Down | Cullin-associated NEDD8-dissociated protein 1 OS=Homo sapiens OX=9606 GN=CAND1 PE=1 SV=2 | CAND1 | _LK[Acetyl (K)]GYLISGSSYAR_ |
| Q86XI2 | 835 | K | 2.240331273 | Up | Condensin-2 complex subunit G2 OS=Homo sapiens OX=9606 GN=NCAPG2 PE=1 SV=1 | NCAPG2 | _LSIHLQHK[Acetyl (K)]FC[Carbamidomethyl (C)]SEGK_ |
| Q86XP3 | 25 | K | 0.58938504 | Down | ATP-dependent RNA helicase DDX42 OS=Homo sapiens OX=9606 GN=DDX42 PE=1 SV=1 | DDX42 | _GFGFGGFAISAGK[Acetyl (K)]K_ |
| Q86YP4 | 349 | K | 0.456091498 | Down | Transcriptional repressor p66-alpha OS=Homo sapiens OX=9606 GN=GATAD2A PE=1 SV=1 | GATAD2A | _QAAAK[Acetyl (K)]LALR_ |
| Q8IVS2 | 254 | K | 0.376218864 | Down | Malonyl-CoA-acyl carrier protein transacylase, mitochondrial OS=Homo sapiens OX=9606 GN=MCAT PE=1 SV=2 | MCAT | _NSSK[Acetyl (K)]FHFR_ |
| Q8IWI9 | 3020 | K | 0.553900066 | Down | MAX gene-associated protein OS=Homo sapiens OX=9606 GN=MGA PE=1 SV=4 | MGA | _VMPC[Carbamidomethyl (C)]LAPIAAK[Acetyl (K)]VGSVGHK_ |
| Q8IWS0 | 312 | K | 0.497059667 | Down | PHD finger protein 6 OS=Homo sapiens OX=9606 GN=PHF6 PE=1 SV=1 | PHF6 | _AK[Acetyl (K)]YIENMSR_ |
| Q8IWW6 | 93 | K | 0.612605734 | Down | Rho GTPase-activating protein 12 OS=Homo sapiens OX=9606 GN=ARHGAP12 PE=1 SV=1 | ARHGAP12 | _QVAGLPNNSTK[Acetyl (K)]IMQSLHLQR_ |
| Q8IX01 | 243 | K | 6.824335119 | Up | SURP and G-patch domain-containing protein 2 OS=Homo sapiens OX=9606 GN=SUGP2 PE=1 SV=2 | SUGP2 | _GGVGK[Acetyl (K)]LVTLR_ |
| Q8IX12 | 967 | K | 1.580257784 | Up | Cell division cycle and apoptosis regulator protein 1 OS=Homo sapiens OX=9606 GN=CCAR1 PE=1 SV=2 | CCAR1 | _LLNK[Acetyl (K)]VVLR_ |
| Q8IX18 | 129 | K | 0.603529793 | Down | Probable ATP-dependent RNA helicase DHX40 OS=Homo sapiens OX=9606 GN=DHX40 PE=1 SV=2 | DHX40 | _C[Carbamidomethyl (C)]TLGSK[Acetyl (K)]VGYQVR_ |
| Q8IX18 | 726 | K | 0.340369821 | Down | Probable ATP-dependent RNA helicase DHX40 OS=Homo sapiens OX=9606 GN=DHX40 PE=1 SV=2 | DHX40 | _WTNK[Acetyl (K)]ENVK[Acetyl (K)]QLK_ |
| Q8IX18 | 730 | K | 0.340369821 | Down | Probable ATP-dependent RNA helicase DHX40 OS=Homo sapiens OX=9606 GN=DHX40 PE=1 SV=2 | DHX40 | _WTNK[Acetyl (K)]ENVK[Acetyl (K)]QLK_ |
| Q8IXB1 | 61 | K | 1.500748675 | Up | DnaJ homolog subfamily C member 10 OS=Homo sapiens OX=9606 GN=DNAJC10 PE=1 SV=2 | DNAJC10 | _LALK[Acetyl (K)]LHPDK_ |
| Q8IXT5 | 916 | K | 0.459908728 | Down | RNA-binding protein 12B OS=Homo sapiens OX=9606 GN=RBM12B PE=1 SV=2 | RBM12B | _FMPDPK[Acetyl (K)]INC[Carbamidomethyl (C)]GSGR_ |
| Q8IXT5 | 928 | K | 0.601298773 | Down | RNA-binding protein 12B OS=Homo sapiens OX=9606 GN=RBM12B PE=1 SV=2 | RBM12B | _VTPIK[Acetyl (K)]IMNLPFK_ |
| Q8IY18 | 115 | K | 0.630270834 | Down | Structural maintenance of chromosomes protein 5 OS=Homo sapiens OX=9606 GN=SMC5 PE=1 SV=2 | SMC5 | _VGFFVK[Acetyl (K)]R_ |
| Q8IY81 | 20 | K | 0.271551111 | Down | pre-rRNA 2'-O-ribose RNA methyltransferase FTSJ3 OS=Homo sapiens OX=9606 GN=FTSJ3 PE=1 SV=2 | FTSJ3 | _FYHLAK[Acetyl (K)]ETGYR_ |
| Q8IY81 | 31 | K | 0.310146568 | Down | pre-rRNA 2'-O-ribose RNA methyltransferase FTSJ3 OS=Homo sapiens OX=9606 GN=FTSJ3 PE=1 SV=2 | FTSJ3 | _SAFK[Acetyl (K)]LIQLNR_ |
| Q8IYB3 | 140 | K | 2.589113612 | Up | Serine/arginine repetitive matrix protein 1 OS=Homo sapiens OX=9606 GN=SRRM1 PE=1 SV=2 | SRRM1 | _QIEQEK[Acetyl (K)]LASMK_ |
| Q8IYB7 | 491 | K | 0.090829872 | Down | DIS3-like exonuclease 2 OS=Homo sapiens OX=9606 GN=DIS3L2 PE=1 SV=4 | DIS3L2 | _SC[Carbamidomethyl (C)]TK[Acetyl (K)]LSYEHAQSMIESPTEK_ |
| Q8IYI6 | 29 | K | 0.611094432 | Down | Exocyst complex component 8 OS=Homo sapiens OX=9606 GN=EXOC8 PE=1 SV=2 | EXOC8 | _LYVK[Acetyl (K)]QLSQQSDGDR_ |
| Q8IYT2 | 97 | K | 0.461792704 | Down | Cap-specific mRNA (nucleoside-2'-O-)-methyltransferase 2 OS=Homo sapiens OX=9606 GN=CMTR2 PE=1 SV=2 | CMTR2 | _AGK[Acetyl (K)]IISHVR_ |
| Q8IYU8 | 17 | K | 0.664406454 | Down | Calcium uptake protein 2, mitochondrial OS=Homo sapiens OX=9606 GN=MICU2 PE=1 SV=2 | MICU2 | _VAAWGGK[Acetyl (K)]LR_ |
| Q8IYU8 | 199 | K | 0.356903312 | Down | Calcium uptake protein 2, mitochondrial OS=Homo sapiens OX=9606 GN=MICU2 PE=1 SV=2 | MICU2 | _EFFK[Acetyl (K)]LQK_ |
| Q8IZL8 | 216 | K | 0.393346974 | Down | Proline-, glutamic acid- and leucine-rich protein 1 OS=Homo sapiens OX=9606 GN=PELP1 PE=1 SV=2 | PELP1 | _GK[Acetyl (K)]LASFFLSR_ |
| Q8IZL8 | 490 | K | 0.621451686 | Down | Proline-, glutamic acid- and leucine-rich protein 1 OS=Homo sapiens OX=9606 GN=PELP1 PE=1 SV=2 | PELP1 | _GSPDGSLQTGK[Acetyl (K)]PSAPK[Acetyl (K)]K_ |
| Q8IZL8 | 495 | K | 0.621451686 | Down | Proline-, glutamic acid- and leucine-rich protein 1 OS=Homo sapiens OX=9606 GN=PELP1 PE=1 SV=2 | PELP1 | _GSPDGSLQTGK[Acetyl (K)]PSAPK[Acetyl (K)]K_ |
| Q8IZQ5 | 20 | K | 1.963447068 | Up | Selenoprotein H OS=Homo sapiens OX=9606 GN=SELENOH PE=1 SV=2 | SELENOH | _KAEAAVVAVAEK[Acetyl (K)]R_ |
| Q8N163 | 215 | K | 1.600936269 | Up | Cell cycle and apoptosis regulator protein 2 OS=Homo sapiens OX=9606 GN=CCAR2 PE=1 SV=2 | CCAR2 | _AGGEPWGAK[Acetyl (K)]K_ |
| Q8N183 | 31 | K | 1.948128861 | Up | NADH dehydrogenase [ubiquinone] 1 alpha subcomplex assembly factor 2 OS=Homo sapiens OX=9606 GN=NDUFAF2 PE=1 SV=1 | NDUFAF2 | _EHVGTDQFGNK[Acetyl (K)]YYYIPQYK_ |
| Q8N1G2 | 97 | K | 2.222469118 | Up | Cap-specific mRNA (nucleoside-2'-O-)-methyltransferase 1 OS=Homo sapiens OX=9606 GN=CMTR1 PE=1 SV=1 | CMTR1 | _LMAK[Acetyl (K)]MGFR_ |
| Q8N3C0 | 399 | K | 1.561647646 | Up | Activating signal cointegrator 1 complex subunit 3 OS=Homo sapiens OX=9606 GN=ASCC3 PE=1 SV=3 | ASCC3 | _DADVEK[Acetyl (K)]IHYPHVYDSQAEAMK_ |
| Q8N3C0 | 860 | K | 2.480859052 | Up | Activating signal cointegrator 1 complex subunit 3 OS=Homo sapiens OX=9606 GN=ASCC3 PE=1 SV=3 | ASCC3 | _AGRPQFDK[Acetyl (K)]FGEGIIITTHDK_ |
| Q8N3F8 | 289 | K | 2.198994348 | Up | MICAL-like protein 1 OS=Homo sapiens OX=9606 GN=MICALL1 PE=1 SV=2 | MICALL1 | _VPGK[Acetyl (K)]LQELASPPAGR_ |
| Q8N465 | 101 | K | 2.864160778 | Up | D-2-hydroxyglutarate dehydrogenase, mitochondrial OS=Homo sapiens OX=9606 GN=D2HGDH PE=1 SV=3 | D2HGDH | _GC[Carbamidomethyl (C)]SK[Acetyl (K)]VLLR_ |
| Q8N4N3 | 379 | K | 0.571006361 | Down | Kelch-like protein 36 OS=Homo sapiens OX=9606 GN=KLHL36 PE=1 SV=1 | KLHL36 | _QWIK[Acetyl (K)]VASMNQR_ |
| Q8N531 | 213 | K | 0.656477674 | Down | F-box/LRR-repeat protein 6 OS=Homo sapiens OX=9606 GN=FBXL6 PE=2 SV=2 | FBXL6 | _SQVHPVLK[Acetyl (K)]LVGEC[Carbamidomethyl (C)]C[Carbamidomethyl (C)]PR_ |
| Q8N5I2 | 207 | K | 0.25825736 | Down | Arrestin domain-containing protein 1 OS=Homo sapiens OX=9606 GN=ARRDC1 PE=1 SV=1 | ARRDC1 | _DTSPVVASLLQK[Acetyl (K)]VSYK_ |
| Q8N680 | 154 | K | 1.509037424 | Up | Zinc finger and BTB domain-containing protein 2 OS=Homo sapiens OX=9606 GN=ZBTB2 PE=1 SV=1 | ZBTB2 | _IASAPEK[Acetyl (K)]LGR_ |
| Q8N8A2 | 171 | K | 2.076858917 | Up | Serine/threonine-protein phosphatase 6 regulatory ankyrin repeat subunit B OS=Homo sapiens OX=9606 GN=ANKRD44 PE=1 SV=3 | ANKRD44 | _GANINAFDK[Acetyl (K)]K_ |
| Q8N9M1 | 166 | K | 2.20457557 | Up | Uncharacterized protein C19orf47 OS=Homo sapiens OX=9606 GN=C19orf47 PE=1 SV=1 | C19orf47 | _RPDTSTSK[Acetyl (K)]ISVTVSNK_ |
| Q8N9M1 | 349 | K | 1.534172532 | Up | Uncharacterized protein C19orf47 OS=Homo sapiens OX=9606 GN=C19orf47 PE=1 SV=1 | C19orf47 | _KPESLSK[Acetyl (K)]VSIIK_ |
| Q8N9T8 | 83 | K | 0.581958072 | Down | Protein KRI1 homolog OS=Homo sapiens OX=9606 GN=KRI1 PE=1 SV=3 | KRI1 | _IYQK[Acetyl (K)]DATFYNR_ |
| Q8N9T8 | 225 | K | 0.616521476 | Down | Protein KRI1 homolog OS=Homo sapiens OX=9606 GN=KRI1 PE=1 SV=3 | KRI1 | _ELTHLK[Acetyl (K)]EYWNDPELDEGER_ |
| Q8NB78 | 109 | K | 0.580349928 | Down | Lysine-specific histone demethylase 2 OS=Homo sapiens OX=9606 GN=KDM1B PE=1 SV=3 | KDM1B | _DGYDK[Acetyl (K)]YTTWK_ |
| Q8NB78 | 250 | K | 0.650081991 | Down | Lysine-specific histone demethylase 2 OS=Homo sapiens OX=9606 GN=KDM1B PE=1 SV=3 | KDM1B | _AAATGNASPGK[Acetyl (K)]LEHSK_ |
| Q8NB90 | 535 | K | 0.276307339 | Down | ATPase family gene 2 protein homolog A OS=Homo sapiens OX=9606 GN=AFG2A PE=1 SV=3 | AFG2A | _LDILQK[Acetyl (K)]LLR_ |
| Q8NB90 | 674 | K | 0.505908844 | Down | ATPase family gene 2 protein homolog A OS=Homo sapiens OX=9606 GN=AFG2A PE=1 SV=3 | AFG2A | _GVLLYGPPGC[Carbamidomethyl (C)]SK[Acetyl (K)]TMIAK_ |
| Q8NBI5 | 182 | K | 4.207470087 | Up | Equilibrative nucleobase transporter 1 OS=Homo sapiens OX=9606 GN=SLC43A3 PE=1 SV=2 | SLC43A3 | _LLYEK[Acetyl (K)]GISLR_ |
| Q8NBJ5 | 145 | K | 1.826816533 | Up | Procollagen galactosyltransferase 1 OS=Homo sapiens OX=9606 GN=COLGALT1 PE=1 SV=1 | COLGALT1 | _YEHVMK[Acetyl (K)]LR_ |
| Q8NBU5 | 112 | K | 0.394119129 | Down | Outer mitochondrial transmembrane helix translocase OS=Homo sapiens OX=9606 GN=ATAD1 PE=1 SV=1 | ATAD1 | _DTVILPIK[Acetyl (K)]K_ |
| Q8NBU5 | 139 | K | 0.456849883 | Down | Outer mitochondrial transmembrane helix translocase OS=Homo sapiens OX=9606 GN=ATAD1 PE=1 SV=1 | ATAD1 | _GVLLYGPPGC[Carbamidomethyl (C)]GK[Acetyl (K)]TLIAK_ |
| Q8NDX5 | 222 | K | 1.616454564 | Up | Polyhomeotic-like protein 3 OS=Homo sapiens OX=9606 GN=PHC3 PE=1 SV=1 | PHC3 | _SQK[Acetyl (K)]LGVLSSSQNGPPK_ |
| Q8NEF9 | 423 | K | 0.553016832 | Down | Serum response factor-binding protein 1 OS=Homo sapiens OX=9606 GN=SRFBP1 PE=1 SV=1 | SRFBP1 | _EQQSNIAVFQGK[Acetyl (K)]K_ |
| Q8NEU8 | 406 | K | 0.552940926 | Down | DCC-interacting protein 13-beta OS=Homo sapiens OX=9606 GN=APPL2 PE=1 SV=3 | APPL2 | _LNQTALQAVTPITSFGK[Acetyl (K)]K_ |
| Q8NFD5 | 1652 | K | 0.630811852 | Down | AT-rich interactive domain-containing protein 1B OS=Homo sapiens OX=9606 GN=ARID1B PE=1 SV=3 | ARID1B | _MQK[Acetyl (K)]VMPTVPTSQVTGPPPQPPPIR_ |
| Q8NG31 | 757 | K | 0.328260241 | Down | Kinetochore scaffold 1 OS=Homo sapiens OX=9606 GN=KNL1 PE=1 SV=3 | KNL1 | _SVLGQNSK[Acetyl (K)]LAEPLR_ |
| Q8NG31 | 2022 | K | 1.75209065 | Up | Kinetochore scaffold 1 OS=Homo sapiens OX=9606 GN=KNL1 PE=1 SV=3 | KNL1 | _VALYGK[Acetyl (K)]LVQSAQNER_ |
| Q8NHH9 | 346 | K | 0.65191568 | Down | Atlastin-2 OS=Homo sapiens OX=9606 GN=ATL2 PE=1 SV=2 | ATL2 | _EISGSK[Acetyl (K)]VTC[Carbamidomethyl (C)]R_ |
| Q8NHH9 | 361 | K | 5.148932506 | Up | Atlastin-2 OS=Homo sapiens OX=9606 GN=ATL2 PE=1 SV=2 | ATL2 | _AYIK[Acetyl (K)]IYQGEELPHPK_ |
| Q8NHQ9 | 295 | K | 0.497602061 | Down | ATP-dependent RNA helicase DDX55 OS=Homo sapiens OX=9606 GN=DDX55 PE=1 SV=3 | DDX55 | _GVK[Acetyl (K)]IMC[Carbamidomethyl (C)]IHGK_ |
| Q8TAA5 | 142 | K | 1.824811469 | Up | GrpE protein homolog 2, mitochondrial OS=Homo sapiens OX=9606 GN=GRPEL2 PE=1 SV=1 | GRPEL2 | _LTLEK[Acetyl (K)]VFR_ |
| Q8TAQ2 | 622 | K | 0.293223197 | Down | SWI/SNF complex subunit SMARCC2 OS=Homo sapiens OX=9606 GN=SMARCC2 PE=1 SV=1 | SMARCC2 | _DDWNK[Acetyl (K)]VSEHVGSR_ |
| Q8TBB5 | 169 | K | 0.542766029 | Down | Kelch domain-containing protein 4 OS=Homo sapiens OX=9606 GN=KLHDC4 PE=1 SV=1 | KLHDC4 | _TWEQVK[Acetyl (K)]STGGPSGR_ |
| Q8TC12 | 194 | K | 2.855784437 | Up | Retinol dehydrogenase 11 OS=Homo sapiens OX=9606 GN=RDH11 PE=1 SV=2 | RDH11 | _IHFHNLQGEK[Acetyl (K)]FYNAGLAYC[Carbamidomethyl (C)]HSK_ |
| Q8TCS8 | 357 | K | 0.623613196 | Down | Polyribonucleotide nucleotidyltransferase 1, mitochondrial OS=Homo sapiens OX=9606 GN=PNPT1 PE=1 SV=2 | PNPT1 | _SIVLNEYK[Acetyl (K)]R_ |
| Q8TD19 | 327 | K | 1.577255883 | Up | Serine/threonine-protein kinase Nek9 OS=Homo sapiens OX=9606 GN=NEK9 PE=1 SV=2 | NEK9 | _VTLLNAPTK[Acetyl (K)]R_ |
| Q8TDM6 | 960 | K | 0.479680994 | Down | Disks large homolog 5 OS=Homo sapiens OX=9606 GN=DLG5 PE=1 SV=4 | DLG5 | _AMLSSTAVPEK[Acetyl (K)]LSVYK_ |
| Q8TED0 | 80 | K | 0.525537271 | Down | U3 small nucleolar RNA-associated protein 15 homolog OS=Homo sapiens OX=9606 GN=UTP15 PE=1 SV=3 | UTP15 | _FK[Acetyl (K)]DTAYC[Carbamidomethyl (C)]ATFR_ |
| Q8TEH3 | 723 | K | 1.588005259 | Up | DENN domain-containing protein 1A OS=Homo sapiens OX=9606 GN=DENND1A PE=1 SV=2 | DENND1A | _PAK[Acetyl (K)]LQAAGAALGDVSER_ |
| Q8TEQ6 | 980 | K | 0.483186397 | Down | Gem-associated protein 5 OS=Homo sapiens OX=9606 GN=GEMIN5 PE=1 SV=3 | GEMIN5 | _QLC[Carbamidomethyl (C)]FQDQYVK[Acetyl (K)]AASHLLSIHK_ |
| Q8TEQ6 | 1187 | K | 0.429926952 | Down | Gem-associated protein 5 OS=Homo sapiens OX=9606 GN=GEMIN5 PE=1 SV=3 | GEMIN5 | _LQNIK[Acetyl (K)]YPSATNNTPAK_ |
| Q8TEV9 | 781 | K | 0.444618091 | Down | Guanine nucleotide exchange protein SMCR8 OS=Homo sapiens OX=9606 GN=SMCR8 PE=1 SV=2 | SMCR8 | _WK[Acetyl (K)]LIGLQR_ |
| Q8TEY7 | 517 | K | 2.240173872 | Up | Ubiquitin carboxyl-terminal hydrolase 33 OS=Homo sapiens OX=9606 GN=USP33 PE=1 SV=2 | USP33 | _EDLAK[Acetyl (K)]LHSSSHPTSIVK_ |
| Q8TF01 | 730 | K | 2.097467533 | Up | Arginine/serine-rich protein PNISR OS=Homo sapiens OX=9606 GN=PNISR PE=1 SV=2 | PNISR | _SGSISVK[Acetyl (K)]IIR_ |
| Q8WTT2 | 393 | K | 2.806646748 | Up | Nucleolar complex protein 3 homolog OS=Homo sapiens OX=9606 GN=NOC3L PE=1 SV=1 | NOC3L | _QDK[Acetyl (K)]LGQASLGVIK_ |
| Q8WTT2 | 628 | K | 1.892289572 | Up | Nucleolar complex protein 3 homolog OS=Homo sapiens OX=9606 GN=NOC3L PE=1 SV=1 | NOC3L | _ALAFIK[Acetyl (K)]R_ |
| Q8WU90 | 93 | K | 0.556953854 | Down | Zinc finger CCCH domain-containing protein 15 OS=Homo sapiens OX=9606 GN=ZC3H15 PE=1 SV=1 | ZC3H15 | _PVVAAQK[Acetyl (K)]ISK_ |
| Q8WUA2 | 30 | K | 1.596233718 | Up | Peptidyl-prolyl cis-trans isomerase-like 4 OS=Homo sapiens OX=9606 GN=PPIL4 PE=1 SV=1 | PPIL4 | _AC[Carbamidomethyl (C)]LNFLK[Acetyl (K)]LC[Carbamidomethyl (C)]K_ |
| Q8WUM0 | 135 | K | 1.782265616 | Up | Nuclear pore complex protein Nup133 OS=Homo sapiens OX=9606 GN=NUP133 PE=1 SV=2 | NUP133 | _IALSPITK[Acetyl (K)]LSVC[Carbamidomethyl (C)]K_ |
| Q8WVC6 | 128 | K | 0.604541676 | Down | Dephospho-CoA kinase domain-containing protein OS=Homo sapiens OX=9606 GN=DCAKD PE=1 SV=1 | DCAKD | _YMK[Acetyl (K)]HTVVVYC[Carbamidomethyl (C)]DR_ |
| Q8WVV9 | 71 | K | 2.32296072 | Up | Heterogeneous nuclear ribonucleoprotein L-like OS=Homo sapiens OX=9606 GN=HNRNPLL PE=1 SV=1 | HNRNPLL | _SFSQPEAGGSHHK[Acetyl (K)]VSVSPVVHVR_ |
| Q8WVV9 | 396 | K | 2.20369982 | Up | Heterogeneous nuclear ribonucleoprotein L-like OS=Homo sapiens OX=9606 GN=HNRNPLL PE=1 SV=1 | HNRNPLL | _AVTHLNNVK[Acetyl (K)]LFGK_ |
| Q8WW12 | 160 | K | 1.615334328 | Up | PEST proteolytic signal-containing nuclear protein OS=Homo sapiens OX=9606 GN=PCNP PE=1 SV=2 | PCNP | _HGFSDNQK[Acetyl (K)]LWER_ |
| Q8WX92 | 146 | K | 0.569369685 | Down | Negative elongation factor B OS=Homo sapiens OX=9606 GN=NELFB PE=1 SV=1 | NELFB | _AC[Carbamidomethyl (C)]AVEVK[Acetyl (K)]R_ |
| Q8WXH0 | 4832 | K | 1.529078641 | Up | Nesprin-2 OS=Homo sapiens OX=9606 GN=SYNE2 PE=1 SV=3 | SYNE2 | _QC[Carbamidomethyl (C)]GMK[Acetyl (K)]LQSLLQK_ |
| Q8WXH0 | 6140 | K | 1.705599095 | Up | Nesprin-2 OS=Homo sapiens OX=9606 GN=SYNE2 PE=1 SV=3 | SYNE2 | _LWQK[Acetyl (K)]FLDDYSR_ |
| Q8WYH8 | 224 | K | 3.541677263 | Up | Inhibitor of growth protein 5 OS=Homo sapiens OX=9606 GN=ING5 PE=1 SV=1 | ING5 | _PK[Acetyl (K)]GK[Acetyl (K)]WFC[Carbamidomethyl (C)]PR_ |
| Q8WYH8 | 226 | K | 1.677475097 | Up | Inhibitor of growth protein 5 OS=Homo sapiens OX=9606 GN=ING5 PE=1 SV=1 | ING5 | _GK[Acetyl (K)]WFC[Carbamidomethyl (C)]PR_ |
| Q92522 | 69 | K | 1.926323606 | Up | Histone H1.10 OS=Homo sapiens OX=9606 GN=H1-10 PE=1 SV=1 | H1-10 | _NGSSLAK[Acetyl (K)]IYTEAK_ |
| Q92613 | 299 | K | 2.024191795 | Up | Protein Jade-3 OS=Homo sapiens OX=9606 GN=JADE3 PE=1 SV=1 | JADE3 | _MEPITK[Acetyl (K)]ISHIPPSR_ |
| Q92625 | 991 | K | 0.656381668 | Down | Ankyrin repeat and SAM domain-containing protein 1A OS=Homo sapiens OX=9606 GN=ANKS1A PE=1 SV=4 | ANKS1A | _GVK[Acetyl (K)]FIDASNK_ |
| Q92759 | 452 | K | 0.627743691 | Down | General transcription factor IIH subunit 4 OS=Homo sapiens OX=9606 GN=GTF2H4 PE=1 SV=1 | GTF2H4 | _LMVVTPAGHSDVK[Acetyl (K)]R_ |
| Q92769 | 201 | K | 0.646927777 | Down | Histone deacetylase 2 OS=Homo sapiens OX=9606 GN=HDAC2 PE=1 SV=2 | HDAC2 | _VMTVSFHK[Acetyl (K)]YGEYFPGTGDLR_ |
| Q92793 | 1583 | K | 3.193895422 | Up | CREB-binding protein OS=Homo sapiens OX=9606 GN=CREBBP PE=1 SV=3 | CREBBP | _EESTAASETTEGSQGDSK[Acetyl (K)]NAK[Acetyl (K)]K[Acetyl (K)]K[Acetyl (K)]NNK_ |
| Q92793 | 1586 | K | 3.193895422 | Up | CREB-binding protein OS=Homo sapiens OX=9606 GN=CREBBP PE=1 SV=3 | CREBBP | _EESTAASETTEGSQGDSK[Acetyl (K)]NAK[Acetyl (K)]K[Acetyl (K)]K[Acetyl (K)]NNK_ |
| Q92793 | 1587 | K | 3.193895422 | Up | CREB-binding protein OS=Homo sapiens OX=9606 GN=CREBBP PE=1 SV=3 | CREBBP | _EESTAASETTEGSQGDSK[Acetyl (K)]NAK[Acetyl (K)]K[Acetyl (K)]K[Acetyl (K)]NNK_ |
| Q92793 | 1588 | K | 1.711053324 | Up | CREB-binding protein OS=Homo sapiens OX=9606 GN=CREBBP PE=1 SV=3 | CREBBP | _EESTAASETTEGSQGDSK[Acetyl (K)]NAK[Acetyl (K)]K[Acetyl (K)]K[Acetyl (K)]NNK_ |
| Q92793 | 1591 | K | 0.086268053 | Down | CREB-binding protein OS=Homo sapiens OX=9606 GN=CREBBP PE=1 SV=3 | CREBBP | _NNK[Acetyl (K)]K[Acetyl (K)]TNK[Acetyl (K)]NK[Acetyl (K)]SSISR_ |
| Q92793 | 1592 | K | 0.086268053 | Down | CREB-binding protein OS=Homo sapiens OX=9606 GN=CREBBP PE=1 SV=3 | CREBBP | _NNK[Acetyl (K)]K[Acetyl (K)]TNK[Acetyl (K)]NK[Acetyl (K)]SSISR_ |
| Q92793 | 1595 | K | 0.086268053 | Down | CREB-binding protein OS=Homo sapiens OX=9606 GN=CREBBP PE=1 SV=3 | CREBBP | _NNK[Acetyl (K)]K[Acetyl (K)]TNK[Acetyl (K)]NK[Acetyl (K)]SSISR_ |
| Q92793 | 1597 | K | 0.028111808 | Down | CREB-binding protein OS=Homo sapiens OX=9606 GN=CREBBP PE=1 SV=3 | CREBBP | _NNK[Acetyl (K)]K[Acetyl (K)]TNK[Acetyl (K)]NK[Acetyl (K)]SSISR_ |
| Q92804 | 277 | K | 2.083376623 | Up | TATA-binding protein-associated factor 2N OS=Homo sapiens OX=9606 GN=TAF15 PE=1 SV=1 | TAF15 | _PMINLYTDK[Acetyl (K)]DTGKPK_ |
| Q92804 | 281 | K | 1.697295056 | Up | TATA-binding protein-associated factor 2N OS=Homo sapiens OX=9606 GN=TAF15 PE=1 SV=1 | TAF15 | _TGKPM[Oxidation (M)]INLYTDKDTGK[Acetyl (K)]PK_ |
| Q92828 | 227 | K | 2.543950455 | Up | Coronin-2A OS=Homo sapiens OX=9606 GN=CORO2A PE=1 SV=2 | CORO2A | _ASK[Acetyl (K)]VLFLGNLK_ |
| Q92841 | 428 | K | 2.100172744 | Up | Probable ATP-dependent RNA helicase DDX17 OS=Homo sapiens OX=9606 GN=DDX17 PE=1 SV=2 | DDX17 | _TIIFVETK[Acetyl (K)]R_ |
| Q92887 | 1329 | K | 0.536051303 | Down | ATP-binding cassette sub-family C member 2 OS=Homo sapiens OX=9606 GN=ABCC2 PE=1 SV=3 | ABCC2 | _GITC[Carbamidomethyl (C)]DIGSMEK[Acetyl (K)]IGVVGR_ |
| Q92890 | 264 | K | 1.539143034 | Up | Ubiquitin recognition factor in ER-associated degradation protein 1 OS=Homo sapiens OX=9606 GN=UFD1 PE=1 SV=3 | UFD1 | _GIPNYEFK[Acetyl (K)]LGK_ |
| Q92896 | 367 | K | 1.702244851 | Up | Golgi apparatus protein 1 OS=Homo sapiens OX=9606 GN=GLG1 PE=1 SV=2 | GLG1 | _LIAQDYK[Acetyl (K)]VSYSLAK_ |
| Q92922 | 61 | K | 0.361349801 | Down | SWI/SNF complex subunit SMARCC1 OS=Homo sapiens OX=9606 GN=SMARCC1 PE=1 SV=3 | SMARCC1 | _VWLGK[Acetyl (K)]HYK_ |
| Q92922 | 103 | K | 0.469027742 | Down | SWI/SNF complex subunit SMARCC1 OS=Homo sapiens OX=9606 GN=SMARCC1 PE=1 SV=3 | SMARCC1 | _HVTNPAFTK[Acetyl (K)]LPAK_ |
| Q92945 | 87 | K | 0.648139315 | Down | Far upstream element-binding protein 2 OS=Homo sapiens OX=9606 GN=KHSRP PE=1 SV=4 | KHSRP | _QIAAK[Acetyl (K)]IGGDAATTVNNSTPDFGFGGQK_ |
| Q92973 | 192 | K | 0.445853201 | Down | Transportin-1 OS=Homo sapiens OX=9606 GN=TNPO1 PE=1 SV=2 | TNPO1 | _FLQFFK[Acetyl (K)]HSSPK_ |
| Q92979 | 37 | K | 3.686548409 | Up | Ribosomal RNA small subunit methyltransferase NEP1 OS=Homo sapiens OX=9606 GN=EMG1 PE=1 SV=4 | EMG1 | _LGAGNK[Acetyl (K)]IGGR_ |
| Q92993 | 52 | K | 2.695044193 | Up | Histone acetyltransferase KAT5 OS=Homo sapiens OX=9606 GN=KAT5 PE=1 SV=2 | KAT5 | _LFYVHYIDFNK[Acetyl (K)]R_ |
| Q93009 | 98 | K | 0.594950572 | Down | Ubiquitin carboxyl-terminal hydrolase 7 OS=Homo sapiens OX=9606 GN=USP7 PE=1 SV=2 | USP7 | _NLPWK[Acetyl (K)]IMVMPR_ |
| Q93009 | 595 | K | 0.423905676 | Down | Ubiquitin carboxyl-terminal hydrolase 7 OS=Homo sapiens OX=9606 GN=USP7 PE=1 SV=2 | USP7 | _YTVFK[Acetyl (K)]VLK_ |
| Q969M1 | 38 | K | 3.001849212 | Up | Mitochondrial import receptor subunit TOM40B OS=Homo sapiens OX=9606 GN=TOMM40L PE=1 SV=1 | TOMM40L | _LC[Carbamidomethyl (C)]K[Acetyl (K)]DVFPAQMEGVK_ |
| Q969Q0 | 61 | K | 0.561308755 | Down | Ribosomal protein eL42-like OS=Homo sapiens OX=9606 GN=RPL36AL PE=1 SV=3 | RPL36AL | _AK[Acetyl (K)]TTK[Acetyl (K)]K[Acetyl (K)]IVLR_ |
| Q969Q0 | 64 | K | 0.561308755 | Down | Ribosomal protein eL42-like OS=Homo sapiens OX=9606 GN=RPL36AL PE=1 SV=3 | RPL36AL | _AK[Acetyl (K)]TTK[Acetyl (K)]K[Acetyl (K)]IVLR_ |
| Q969Q0 | 65 | K | 0.561308755 | Down | Ribosomal protein eL42-like OS=Homo sapiens OX=9606 GN=RPL36AL PE=1 SV=3 | RPL36AL | _AK[Acetyl (K)]TTK[Acetyl (K)]K[Acetyl (K)]IVLR_ |
| Q969X5 | 288 | K | 0.404183038 | Down | Endoplasmic reticulum-Golgi intermediate compartment protein 1 OS=Homo sapiens OX=9606 GN=ERGIC1 PE=1 SV=1 | ERGIC1 | _IQLGK[Acetyl (K)]MH_ |
| Q96A33 | 419 | K | 0.632067585 | Down | PAT complex subunit CCDC47 OS=Homo sapiens OX=9606 GN=CCDC47 PE=1 SV=1 | CCDC47 | _VEENFLK[Acetyl (K)]LTHVQR_ |
| Q96AE4 | 44 | K | 0.395020879 | Down | Far upstream element-binding protein 1 OS=Homo sapiens OX=9606 GN=FUBP1 PE=1 SV=3 | FUBP1 | _QIAAK[Acetyl (K)]IGGDAGTSLNSNDYGYGGQK_ |
| Q96AG4 | 143 | K | 0.297921455 | Down | Leucine-rich repeat-containing protein 59 OS=Homo sapiens OX=9606 GN=LRRC59 PE=1 SV=1 | LRRC59 | _QC[Carbamidomethyl (C)]ANK[Acetyl (K)]VLQHMK_ |
| Q96AX1 | 429 | K | 1.750206527 | Up | Vacuolar protein sorting-associated protein 33A OS=Homo sapiens OX=9606 GN=VPS33A PE=1 SV=1 | VPS33A | _VLDYYK[Acetyl (K)]R_ |
| Q96AY2 | 103 | K | 1.96706643 | Up | Crossover junction endonuclease EME1 OS=Homo sapiens OX=9606 GN=EME1 PE=1 SV=2 | EME1 | _LTC[Carbamidomethyl (C)]K[Acetyl (K)]FLTHK_ |
| Q96B77 | 184 | K | 0.615242655 | Down | Transmembrane protein 186 OS=Homo sapiens OX=9606 GN=TMEM186 PE=1 SV=1 | TMEM186 | _YSGK[Acetyl (K)]QTFYVTLR_ |
| Q96BK5 | 57 | K | 1.973209065 | Up | PIN2/TERF1-interacting telomerase inhibitor 1 OS=Homo sapiens OX=9606 GN=PINX1 PE=1 SV=2 | PINX1 | _GLGAQEQGATDHIK[Acetyl (K)]VQVK_ |
| Q96BP3 | 220 | K | 1.731603962 | Up | Peptidylprolyl isomerase domain and WD repeat-containing protein 1 OS=Homo sapiens OX=9606 GN=PPWD1 PE=1 SV=1 | PPWD1 | _GDNQPLHIFDK[Acetyl (K)]LHTSPLTQIR_ |
| Q96BW9 | 77 | K | 0.470635897 | Down | Phosphatidate cytidylyltransferase, mitochondrial OS=Homo sapiens OX=9606 GN=TAMM41 PE=1 SV=2 | TAMM41 | _NWSHYSFLK[Acetyl (K)]VLGPK_ |
| Q96C19 | 123 | K | 1.513378136 | Up | EF-hand domain-containing protein D2 OS=Homo sapiens OX=9606 GN=EFHD2 PE=1 SV=1 | EFHD2 | _LMMEK[Acetyl (K)]LGAPQTHLGLK_ |
| Q96CB8 | 85 | K | 1.742322977 | Up | Integrator complex subunit 12 OS=Homo sapiens OX=9606 GN=INTS12 PE=1 SV=1 | INTS12 | _ISSSLPSGNNNGK[Acetyl (K)]VLTTEK_ |
| Q96CB8 | 347 | K | 0.604861852 | Down | Integrator complex subunit 12 OS=Homo sapiens OX=9606 GN=INTS12 PE=1 SV=1 | INTS12 | _GGIGSK[Acetyl (K)]IGSNNSTTPTVPLKPPPPLTLGK_ |
| Q96CG3 | 48 | K | 0.232637847 | Down | TRAF-interacting protein with FHA domain-containing protein A OS=Homo sapiens OX=9606 GN=TIFA PE=1 SV=1 | TIFA | _LPSSEVVK[Acetyl (K)]FGR_ |
| Q96CS3 | 167 | K | 0.586457514 | Down | FAS-associated factor 2 OS=Homo sapiens OX=9606 GN=FAF2 PE=1 SV=2 | FAF2 | _AHPVFYQGTYSQALNDAK[Acetyl (K)]R_ |
| Q96DH6 | 94 | K | 0.58985702 | Down | RNA-binding protein Musashi homolog 2 OS=Homo sapiens OX=9606 GN=MSI2 PE=1 SV=1 | MSI2 | _TIDPK[Acetyl (K)]VAFPR_ |
| Q96DT7 | 497 | K | 0.498195187 | Down | Zinc finger and BTB domain-containing protein 10 OS=Homo sapiens OX=9606 GN=ZBTB10 PE=1 SV=2 | ZBTB10 | _DQK[Acetyl (K)]IASFWATR_ |
| Q96EE3 | 105 | K | 1.751912144 | Up | Nucleoporin SEH1 OS=Homo sapiens OX=9606 GN=SEH1L PE=1 SV=3 | SEH1L | _GQSHWVK[Acetyl (K)]R_ |
| Q96EY7 | 315 | K | 2.27610588 | Up | Small ribosomal subunit protein mS39 OS=Homo sapiens OX=9606 GN=PTCD3 PE=1 SV=3 | PTCD3 | _WSK[Acetyl (K)]ILELLR_ |
| Q96FV9 | 300 | K | 1.568297775 | Up | THO complex subunit 1 OS=Homo sapiens OX=9606 GN=THOC1 PE=1 SV=1 | THOC1 | _TGGEHVYFAK[Acetyl (K)]FLTSEK_ |
| Q96G01 | 787 | K | 1.583507748 | Up | Protein bicaudal D homolog 1 OS=Homo sapiens OX=9606 GN=BICD1 PE=1 SV=3 | BICD1 | _MAIQQK[Acetyl (K)]LALTQR_ |
| Q96G21 | 105 | K | 1.697408224 | Up | U3 small nucleolar ribonucleoprotein protein IMP4 OS=Homo sapiens OX=9606 GN=IMP4 PE=1 SV=1 | IMP4 | _ELK[Acetyl (K)]LVFPGAQR_ |
| Q96HE7 | 67 | K | 0.633988612 | Down | ERO1-like protein alpha OS=Homo sapiens OX=9606 GN=ERO1A PE=1 SV=2 | ERO1A | _LQK[Acetyl (K)]LLESDYFR_ |
| Q96HJ9 | 40 | K | 0.247404999 | Down | Protein FMC1 homolog OS=Homo sapiens OX=9606 GN=FMC1 PE=1 SV=2 | FMC1 | _YLVK[Acetyl (K)]AFR_ |
| Q96HJ9 | 51 | K | 0.296155343 | Down | Protein FMC1 homolog OS=Homo sapiens OX=9606 GN=FMC1 PE=1 SV=2 | FMC1 | _VTSEK[Acetyl (K)]LC[Carbamidomethyl (C)]R_ |
| Q96I25 | 21 | K | 0.597802759 | Down | Splicing factor 45 OS=Homo sapiens OX=9606 GN=RBM17 PE=1 SV=1 | RBM17 | _TEGWSK[Acetyl (K)]NFK_ |
| Q96I51 | 138 | K | 0.453199545 | Down | RCC1-like G exchanging factor-like protein OS=Homo sapiens OX=9606 GN=RCC1L PE=1 SV=3 | RCC1L | _VWGMGLNK[Acetyl (K)]DSQLGFHR_ |
| Q96IJ6 | 379 | K | 0.55811458 | Down | Mannose-1-phosphate guanyltransferase alpha OS=Homo sapiens OX=9606 GN=GMPPA PE=1 SV=1 | GMPPA | _DGK[Acetyl (K)]LLPAITILGC[Carbamidomethyl (C)]R_ |
| Q96JH7 | 1005 | K | 0.636051703 | Down | Deubiquitinating protein VCPIP1 OS=Homo sapiens OX=9606 GN=VCPIP1 PE=1 SV=2 | VCPIP1 | _ESSPSHGLLK[Acetyl (K)]LGSGGVVK_ |
| Q96JM7 | 452 | K | 14.88202746 | Up | Lethal(3)malignant brain tumor-like protein 3 OS=Homo sapiens OX=9606 GN=L3MBTL3 PE=1 SV=2 | L3MBTL3 | _HFSWDK[Acetyl (K)]YLEETNSLPAPAR_ |
| Q96JP0 | 608 | K | 2.256497983 | Up | Protein fem-1 homolog C OS=Homo sapiens OX=9606 GN=FEM1C PE=1 SV=1 | FEM1C | _GHIPEK[Acetyl (K)]LETFVSLHR_ |
| Q96JY6 | 282 | K | 0.311614182 | Down | PDZ and LIM domain protein 2 OS=Homo sapiens OX=9606 GN=PDLIM2 PE=1 SV=1 | PDLIM2 | _ALATPPK[Acetyl (K)]LHTC[Carbamidomethyl (C)]EK_ |
| Q96KB5 | 64 | K | 0.270870462 | Down | Lymphokine-activated killer T-cell-originated protein kinase OS=Homo sapiens OX=9606 GN=PBK PE=1 SV=3 | PBK | _GLSHSPWAVK[Acetyl (K)]K_ |
| Q96KQ7 | 354 | K | 1.846000971 | Up | Histone-lysine N-methyltransferase EHMT2 OS=Homo sapiens OX=9606 GN=EHMT2 PE=1 SV=3 | EHMT2 | _KDSPWVK[Acetyl (K)]PSR_ |
| Q96KR1 | 484 | K | 0.560047199 | Down | Zinc finger RNA-binding protein OS=Homo sapiens OX=9606 GN=ZFR PE=1 SV=2 | ZFR | _GLTTTGNSSLNSTSNTK[Acetyl (K)]VSAVPTNMAAK_ |
| Q96KR1 | 585 | K | 11.14077204 | Up | Zinc finger RNA-binding protein OS=Homo sapiens OX=9606 GN=ZFR PE=1 SV=2 | ZFR | _FHC[Carbamidomethyl (C)]K[Acetyl (K)]LC[Carbamidomethyl (C)]EC[Carbamidomethyl (C)]SFNDPNAK_ |
| Q96LT9 | 364 | K | 2.099551747 | Up | RNA-binding region-containing protein 3 OS=Homo sapiens OX=9606 GN=RNPC3 PE=1 SV=1 | RNPC3 | _NHDLPATEVDASNIGFGK[Acetyl (K)]IFPK_ |
| Q96NB2 | 258 | K | 2.029267637 | Up | Sideroflexin-2 OS=Homo sapiens OX=9606 GN=SFXN2 PE=1 SV=2 | SFXN2 | _LHFMQK[Acetyl (K)]VK_ |
| Q96P11 | 262 | K | 0.601519769 | Down | 28S rRNA (cytosine-C(5))-methyltransferase OS=Homo sapiens OX=9606 GN=NSUN5 PE=1 SV=2 | NSUN5 | _IFAFDLDAK[Acetyl (K)]R_ |
| Q96PV6 | 400 | K | 0.567955743 | Down | Leukocyte receptor cluster member 8 OS=Homo sapiens OX=9606 GN=LENG8 PE=1 SV=3 | LENG8 | _GNSFTK[Acetyl (K)]FGNR_ |
| Q96Q05 | 98 | K | 0.520037712 | Down | Trafficking protein particle complex subunit 9 OS=Homo sapiens OX=9606 GN=TRAPPC9 PE=1 SV=2 | TRAPPC9 | _DWPQTFEK[Acetyl (K)]FHVQK_ |
| Q96QC0 | 609 | K | 1.569319479 | Up | Serine/threonine-protein phosphatase 1 regulatory subunit 10 OS=Homo sapiens OX=9606 GN=PPP1R10 PE=1 SV=1 | PPP1R10 | _QPDYSDK[Acetyl (K)]IK_ |
| Q96QE5 | 321 | K | 1.977846173 | Up | Transcription elongation factor, mitochondrial OS=Homo sapiens OX=9606 GN=TEFM PE=1 SV=1 | TEFM | _VFFPSDK[Acetyl (K)]IVHYR_ |
| Q96QK1 | 555 | K | 0.555076913 | Down | Vacuolar protein sorting-associated protein 35 OS=Homo sapiens OX=9606 GN=VPS35 PE=1 SV=2 | VPS35 | _VDDKWEK[Acetyl (K)]K_ |
| Q96QR8 | 267 | K | 0.551613156 | Down | Transcriptional activator protein Pur-beta OS=Homo sapiens OX=9606 GN=PURB PE=1 SV=3 | PURB | _AWGK[Acetyl (K)]FGGAFC[Carbamidomethyl (C)]R_ |
| Q96R05 | 64 | K | 2.546335844 | Up | Retinoid-binding protein 7 OS=Homo sapiens OX=9606 GN=RBP7 PE=1 SV=1 | RBP7 | _NYFVK[Acetyl (K)]FK_ |
| Q96RT1 | 162 | K | 16.78552226 | Up | Erbin OS=Homo sapiens OX=9606 GN=ERBIN PE=1 SV=2 | ERBIN | _LTK[Acetyl (K)]LQILELR_ |
| Q96RT1 | 468 | K | 0.142808815 | Down | Erbin OS=Homo sapiens OX=9606 GN=ERBIN PE=1 SV=2 | ERBIN | _AQVAFEC[Carbamidomethyl (C)]DEDK[Acetyl (K)]DER_ |
| Q96SK2 | 304 | K | 0.288211058 | Down | Transmembrane protein 209 OS=Homo sapiens OX=9606 GN=TMEM209 PE=1 SV=2 | TMEM209 | _SQAPC[Carbamidomethyl (C)]ANK[Acetyl (K)]DEADLSSK_ |
| Q96ST3 | 1164 | K | 0.647229044 | Down | Paired amphipathic helix protein Sin3a OS=Homo sapiens OX=9606 GN=SIN3A PE=1 SV=2 | SIN3A | _TMENVDSLDK[Acetyl (K)]LEC[Carbamidomethyl (C)]R_ |
| Q96SW2 | 300 | K | 2.333765025 | Up | Protein cereblon OS=Homo sapiens OX=9606 GN=CRBN PE=1 SV=1 | CRBN | _IQLLK[Acetyl (K)]IGSAIQR_ |
| Q96T37 | 420 | K | 0.536333002 | Down | RNA-binding protein 15 OS=Homo sapiens OX=9606 GN=RBM15 PE=1 SV=2 | RBM15 | _GQTSTYGFLK[Acetyl (K)]FENLDMSHR_ |
| Q96T58 | 2497 | K | 1.76478507 | Up | Msx2-interacting protein OS=Homo sapiens OX=9606 GN=SPEN PE=1 SV=1 | SPEN | _LSPPVASGGIPHQSPPTK[Acetyl (K)]VTEWITR_ |
| Q96T58 | 3020 | K | 1.79106797 | Up | Msx2-interacting protein OS=Homo sapiens OX=9606 GN=SPEN PE=1 SV=1 | SPEN | _TVSHLAAAK[Acetyl (K)]LDAHSPR_ |
| Q96T88 | 745 | K | 1.934933304 | Up | E3 ubiquitin-protein ligase UHRF1 OS=Homo sapiens OX=9606 GN=UHRF1 PE=1 SV=1 | UHRF1 | _PITTVC[Carbamidomethyl (C)]QHNVC[Carbamidomethyl (C)]K[Acetyl (K)]DC[Carbamidomethyl (C)]LDR_ |
| Q99439 | 234 | K | 0.640164624 | Down | Calponin-2 OS=Homo sapiens OX=9606 GN=CNN2 PE=1 SV=4 | CNN2 | _HIYDTK[Acetyl (K)]LGTDK_ |
| Q99459 | 76 | K | 0.363076757 | Down | Cell division cycle 5-like protein OS=Homo sapiens OX=9606 GN=CDC5L PE=1 SV=2 | CDC5L | _LLHLAK[Acetyl (K)]LMPTQWR_ |
| Q99460 | 136 | K | 0.637966435 | Down | 26S proteasome non-ATPase regulatory subunit 1 OS=Homo sapiens OX=9606 GN=PSMD1 PE=1 SV=2 | PSMD1 | _LEGIVNK[Acetyl (K)]MFQR_ |
| Q99569 | 601 | K | 1.913698026 | Up | Plakophilin-4 OS=Homo sapiens OX=9606 GN=PKP4 PE=1 SV=2 | PKP4 | _NLVFGK[Acetyl (K)]STDENK_ |
| Q99594 | 379 | K | 1.806495665 | Up | Transcriptional enhancer factor TEF-5 OS=Homo sapiens OX=9606 GN=TEAD3 PE=1 SV=2 | TEAD3 | _LK[Acetyl (K)]HLPEK_ |
| Q99598 | 252 | K | 0.449779474 | Down | Translin-associated protein X OS=Homo sapiens OX=9606 GN=TSNAX PE=1 SV=1 | TSNAX | _QSLAK[Acetyl (K)]VENAC[Carbamidomethyl (C)]YALK_ |
| Q99615 | 353 | K | 1.534951055 | Up | DnaJ homolog subfamily C member 7 OS=Homo sapiens OX=9606 GN=DNAJC7 PE=1 SV=2 | DNAJC7 | _DYEK[Acetyl (K)]VYQTEK_ |
| Q99623 | 147 | K | 0.540158175 | Down | Prohibitin-2 OS=Homo sapiens OX=9606 GN=PHB2 PE=1 SV=2 | PHB2 | _SVVAK[Acetyl (K)]FNASQLITQR_ |
| Q99643 | 44 | K | 4.235648601 | Up | Succinate dehydrogenase cytochrome b560 subunit, mitochondrial OS=Homo sapiens OX=9606 GN=SDHC PE=1 SV=1 | SDHC | _FWNK[Acetyl (K)]NIGSNR_ |
| Q99729 | 91 | K | 0.477724989 | Down | Heterogeneous nuclear ribonucleoprotein A/B OS=Homo sapiens OX=9606 GN=HNRNPAB PE=1 SV=2 | HNRNPAB | _DYFTK[Acetyl (K)]FGEVVDC[Carbamidomethyl (C)]TIK_ |
| Q99832 | 172 | K | 0.625392192 | Down | T-complex protein 1 subunit eta OS=Homo sapiens OX=9606 GN=CCT7 PE=1 SV=2 | CCT7 | _LISQQK[Acetyl (K)]AFFAK_ |
| Q99848 | 149 | K | 0.429474637 | Down | Probable rRNA-processing protein EBP2 OS=Homo sapiens OX=9606 GN=EBNA1BP2 PE=1 SV=2 | EBNA1BP2 | _SDLQMQK[Acetyl (K)]IR_ |
| Q9BPW8 | 141 | K | 0.435106917 | Down | Protein NipSnap homolog 1 OS=Homo sapiens OX=9606 GN=NIPSNAP1 PE=1 SV=1 | NIPSNAP1 | _FSGGYPALMDC[Carbamidomethyl (C)]MNK[Acetyl (K)]LK_ |
| Q9BPW8 | 191 | K | 0.202344751 | Down | Protein NipSnap homolog 1 OS=Homo sapiens OX=9606 GN=NIPSNAP1 PE=1 SV=1 | NIPSNAP1 | _TYK[Acetyl (K)]LKPGTMIEWGNNWAR_ |
| Q9BQ13 | 231 | K | 1.509893893 | Up | BTB/POZ domain-containing protein KCTD14 OS=Homo sapiens OX=9606 GN=KCTD14 PE=1 SV=2 | KCTD14 | _VFSK[Acetyl (K)]FYLTYPTK_ |
| Q9BQ13 | 239 | K | 1.744852952 | Up | BTB/POZ domain-containing protein KCTD14 OS=Homo sapiens OX=9606 GN=KCTD14 PE=1 SV=2 | KCTD14 | _FYLTYPTK[Acetyl (K)]R_ |
| Q9BQ69 | 117 | K | 0.605568063 | Down | ADP-ribose glycohydrolase MACROD1 OS=Homo sapiens OX=9606 GN=MACROD1 PE=1 SV=2 | MACROD1 | _EEHYFC[Carbamidomethyl (C)]K[Acetyl (K)]DFVR_ |
| Q9BQE3 | 311 | K | 0.56062808 | Down | Tubulin alpha-1C chain OS=Homo sapiens OX=9606 GN=TUBA1C PE=1 SV=1 | TUBA1C | _HGK[Acetyl (K)]YMAC[Carbamidomethyl (C)]C[Carbamidomethyl (C)]LLYR_ |
| Q9BQE3 | 370 | K | 0.563087208 | Down | Tubulin alpha-1C chain OS=Homo sapiens OX=9606 GN=TUBA1C PE=1 SV=1 | TUBA1C | _VGINYQPPTVVPGGDLAK[Acetyl (K)]VQR_ |
| Q9BQE3 | 401 | K | 0.662193409 | Down | Tubulin alpha-1C chain OS=Homo sapiens OX=9606 GN=TUBA1C PE=1 SV=1 | TUBA1C | _FDLMYAK[Acetyl (K)]R_ |
| Q9BRD0 | 499 | K | 0.208006689 | Down | BUD13 homolog OS=Homo sapiens OX=9606 GN=BUD13 PE=1 SV=1 | BUD13 | _DSERDELYAQWGK[Acetyl (K)]GLAQSR_ |
| Q9BRJ2 | 264 | K | 0.411971807 | Down | Large ribosomal subunit protein mL45 OS=Homo sapiens OX=9606 GN=MRPL45 PE=1 SV=2 | MRPL45 | _MHTK[Acetyl (K)]IVPPWAPPK_ |
| Q9BRJ7 | 151 | K | 0.514675208 | Down | Tudor-interacting repair regulator protein OS=Homo sapiens OX=9606 GN=NUDT16L1 PE=1 SV=1 | NUDT16L1 | _VPLYTQK[Acetyl (K)]DR_ |
| Q9BRQ8 | 225 | K | 1.575913434 | Up | Ferroptosis suppressor protein 1 OS=Homo sapiens OX=9606 GN=AIFM2 PE=1 SV=1 | AIFM2 | _EYIK[Acetyl (K)]VQTDK_ |
| Q9BRR3 | 244 | K | 2.121403891 | Up | Post-GPI attachment to proteins factor 4 OS=Homo sapiens OX=9606 GN=PGAP4 PE=1 SV=1 | PGAP4 | _DALYLK[Acetyl (K)]LYHPER_ |
| Q9BRX2 | 297 | K | 0.612063291 | Down | Protein pelota homolog OS=Homo sapiens OX=9606 GN=PELO PE=1 SV=2 | PELO | _AFYGLK[Acetyl (K)]QVEK_ |
| Q9BS26 | 345 | K | 0.561689193 | Down | Endoplasmic reticulum resident protein 44 OS=Homo sapiens OX=9606 GN=ERP44 PE=1 SV=1 | ERP44 | _LK[Acetyl (K)]QFVFDLHSGK_ |
| Q9BSH4 | 231 | K | 1.541784166 | Up | Translational activator of cytochrome c oxidase 1 OS=Homo sapiens OX=9606 GN=TACO1 PE=1 SV=1 | TACO1 | _NVFK[Acetyl (K)]FIC[Carbamidomethyl (C)]DASSLHQVR_ |
| Q9BT22 | 236 | K | 17.08430623 | Up | Chitobiosyldiphosphodolichol beta-mannosyltransferase OS=Homo sapiens OX=9606 GN=ALG1 PE=1 SV=2 | ALG1 | _LFMK[Acetyl (K)]LGSMHSPFR_ |
| Q9BTE7 | 124 | K | 1.950602882 | Up | DCN1-like protein 5 OS=Homo sapiens OX=9606 GN=DCUN1D5 PE=1 SV=1 | DCUN1D5 | _LQNK[Acetyl (K)]FDFLR_ |
| Q9BTU6 | 172 | K | 2.033974317 | Up | Phosphatidylinositol 4-kinase type 2-alpha OS=Homo sapiens OX=9606 GN=PI4K2A PE=1 SV=1 | PI4K2A | _WLQK[Acetyl (K)]LC[Carbamidomethyl (C)]C[Carbamidomethyl (C)]PC[Carbamidomethyl (C)]C[Carbamidomethyl (C)]FGR_ |
| Q9BUQ8 | 686 | K | 0.643688267 | Down | Probable ATP-dependent RNA helicase DDX23 OS=Homo sapiens OX=9606 GN=DDX23 PE=1 SV=3 | DDX23 | _SLEK[Acetyl (K)]MGYNAC[Carbamidomethyl (C)]TLHGGK_ |
| Q9BUT9 | 10 | K | 0.289370911 | Down | MAPK regulated corepressor interacting protein 2 OS=Homo sapiens OX=9606 GN=MCRIP2 PE=1 SV=2 | MCRIP2 | _GPSK[Acetyl (K)]LVAQR_ |
| Q9BVC5 | 72 | K | 2.598187869 | Up | Ashwin OS=Homo sapiens OX=9606 GN=C2orf49 PE=1 SV=1 | C2orf49 | _WGK[Acetyl (K)]MMEK_ |
| Q9BVI4 | 232 | K | 0.632919522 | Down | Nucleolar complex protein 4 homolog OS=Homo sapiens OX=9606 GN=NOC4L PE=1 SV=1 | NOC4L | _REPTVSSFYVK[Acetyl (K)]R_ |
| Q9BVJ6 | 45 | K | 1.690719086 | Up | U3 small nucleolar RNA-associated protein 14 homolog A OS=Homo sapiens OX=9606 GN=UTP14A PE=1 SV=1 | UTP14A | _HQK[Acetyl (K)]LLEAISSLDGK_ |
| Q9BVJ6 | 466 | K | 0.630257231 | Down | U3 small nucleolar RNA-associated protein 14 homolog A OS=Homo sapiens OX=9606 GN=UTP14A PE=1 SV=1 | UTP14A | _VLSQK[Acetyl (K)]LK_ |
| Q9BVK6 | 71 | K | 1.819814609 | Up | Transmembrane emp24 domain-containing protein 9 OS=Homo sapiens OX=9606 GN=TMED9 PE=1 SV=2 | TMED9 | _TQLYDK[Acetyl (K)]QR_ |
| Q9BVP2 | 135 | K | 0.622905134 | Down | Guanine nucleotide-binding protein-like 3 OS=Homo sapiens OX=9606 GN=GNL3 PE=1 SV=2 | GNL3 | _LYC[Carbamidomethyl (C)]QELK[Acetyl (K)]K_ |
| Q9BVQ7 | 511 | K | 0.638681795 | Down | ATPase family gene 2 protein homolog B OS=Homo sapiens OX=9606 GN=AFG2B PE=1 SV=2 | AFG2B | _GVLLYGPPGC[Carbamidomethyl (C)]AK[Acetyl (K)]TTLVR_ |
| Q9BWJ5 | 28 | K | 0.569478667 | Down | Splicing factor 3B subunit 5 OS=Homo sapiens OX=9606 GN=SF3B5 PE=1 SV=1 | SF3B5 | _YIGTGHADTTK[Acetyl (K)]WEWLVNQHR_ |
| Q9BWN1 | 256 | K | 0.358800972 | Down | Proline-rich protein 14 OS=Homo sapiens OX=9606 GN=PRR14 PE=1 SV=1 | PRR14 | _SK[Acetyl (K)]LESFADIFLTPNK_ |
| Q9BXP5 | 419 | K | 0.287652943 | Down | Serrate RNA effector molecule homolog OS=Homo sapiens OX=9606 GN=SRRT PE=1 SV=1 | SRRT | _PLHK[Acetyl (K)]TC[Carbamidomethyl (C)]SLFMR_ |
| Q9BXY0 | 115 | K | 0.184915015 | Down | Protein MAK16 homolog OS=Homo sapiens OX=9606 GN=MAK16 PE=1 SV=2 | MAK16 | _FTK[Acetyl (K)]ITQYLIR_ |
| Q9BYD2 | 249 | K | 0.556800468 | Down | Large ribosomal subunit protein bL9m OS=Homo sapiens OX=9606 GN=MRPL9 PE=1 SV=2 | MRPL9 | _YK[Acetyl (K)]YWLAQQAAK_ |
| Q9BYW2 | 141 | K | 2.145733224 | Up | Histone-lysine N-methyltransferase SETD2 OS=Homo sapiens OX=9606 GN=SETD2 PE=1 SV=3 | SETD2 | _VELGK[Acetyl (K)]IHFK_ |
| Q9BZE4 | 142 | K | 1.687042844 | Up | GTP-binding protein 4 OS=Homo sapiens OX=9606 GN=GTPBP4 PE=1 SV=3 | GTPBP4 | _MC[Carbamidomethyl (C)]TVIK[Acetyl (K)]R_ |
| Q9BZK7 | 102 | K | 0.599661137 | Down | F-box-like/WD repeat-containing protein TBL1XR1 OS=Homo sapiens OX=9606 GN=TBL1XR1 PE=1 SV=1 | TBL1XR1 | _DK[Acetyl (K)]LAQQQAAAAAAAAAAASQQGSAK_ |
| Q9C0H5 | 624 | K | 2.892971856 | Up | Rho GTPase-activating protein 39 OS=Homo sapiens OX=9606 GN=ARHGAP39 PE=1 SV=2 | ARHGAP39 | _GTFEK[Acetyl (K)]LGFPQILLEK_ |
| Q9C0J8 | 46 | K | 0.627121631 | Down | pre-mRNA 3' end processing protein WDR33 OS=Homo sapiens OX=9606 GN=WDR33 PE=1 SV=2 | WDR33 | _RPDFAQQQAMQQLTFDGK[Acetyl (K)]R_ |
| Q9GZN8 | 59 | K | 2.055011569 | Up | Adipose-secreted signaling protein OS=Homo sapiens OX=9606 GN=ADISSP PE=1 SV=3 | ADISSP | _VGFLK[Acetyl (K)]ILHR_ |
| Q9GZR2 | 273 | K | 1.655574859 | Up | RNA exonuclease 4 OS=Homo sapiens OX=9606 GN=REXO4 PE=1 SV=2 | REXO4 | _VSIVNQYGK[Acetyl (K)]C[Carbamidomethyl (C)]VYDK_ |
| Q9GZR7 | 541 | K | 5.177056914 | Up | ATP-dependent RNA helicase DDX24 OS=Homo sapiens OX=9606 GN=DDX24 PE=1 SV=1 | DDX24 | _LDLLMQK[Acetyl (K)]IGM[Oxidation (M)]R_ |
| Q9GZT3 | 36 | K | 1.676268206 | Up | SRA stem-loop-interacting RNA-binding protein, mitochondrial OS=Homo sapiens OX=9606 GN=SLIRP PE=1 SV=1 | SLIRP | _IPWTAASSQLK[Acetyl (K)]EHFAQFGHVR_ |
| Q9GZV4 | 39 | K | 0.294757644 | Down | Eukaryotic translation initiation factor 5A-2 OS=Homo sapiens OX=9606 GN=EIF5A2 PE=1 SV=3 | EIF5A2 | _GRPC[Carbamidomethyl (C)]K[Acetyl (K)]IVEMSTSK_ |
| Q9GZV4 | 67 | K | 0.204738612 | Down | Eukaryotic translation initiation factor 5A-2 OS=Homo sapiens OX=9606 GN=EIF5A2 PE=1 SV=3 | EIF5A2 | _VHLVGIDIFTGK[Acetyl (K)]K_ |
| Q9H0A0 | 426 | K | 2.260900673 | Up | RNA cytidine acetyltransferase OS=Homo sapiens OX=9606 GN=NAT10 PE=1 SV=2 | NAT10 | _SLSLK[Acetyl (K)]LIQQLR_ |
| Q9H0H0 | 1165 | K | 0.666161081 | Down | Integrator complex subunit 2 OS=Homo sapiens OX=9606 GN=INTS2 PE=1 SV=2 | INTS2 | _EKPSGWSQIC[Carbamidomethyl (C)]K[Acetyl (K)]DSSYK_ |
| Q9H0N5 | 86 | K | 2.025597172 | Up | Pterin-4-alpha-carbinolamine dehydratase 2 OS=Homo sapiens OX=9606 GN=PCBD2 PE=1 SV=4 | PCBD2 | _VALQAEK[Acetyl (K)]MNHHPEWFNVYNK_ |
| Q9H0P0 | 252 | K | 0.655697427 | Down | Cytosolic 5'-nucleotidase 3A OS=Homo sapiens OX=9606 GN=NT5C3A PE=1 SV=3 | NT5C3A | _GELIHVFNK[Acetyl (K)]HDGALR_ |
| Q9H0X4 | 295 | K | 1.59367114 | Up | Protein FAM234A OS=Homo sapiens OX=9606 GN=FAM234A PE=1 SV=1 | FAM234A | _GLYEK[Acetyl (K)]VTGSGGPFK_ |
| Q9H267 | 470 | K | 1.700737156 | Up | Vacuolar protein sorting-associated protein 33B OS=Homo sapiens OX=9606 GN=VPS33B PE=1 SV=2 | VPS33B | _AAGK[Acetyl (K)]ITDAFSSLAK_ |
| Q9H270 | 417 | K | 1.668976254 | Up | Vacuolar protein sorting-associated protein 11 homolog OS=Homo sapiens OX=9606 GN=VPS11 PE=1 SV=1 | VPS11 | _TIGK[Acetyl (K)]LEPSYVIR_ |
| Q9H2G2 | 1223 | K | 0.569980041 | Down | STE20-like serine/threonine-protein kinase OS=Homo sapiens OX=9606 GN=SLK PE=1 SV=1 | SLK | _ISK[Acetyl (K)]FYPIPSLHSTGS_ |
| Q9H2P0 | 490 | K | 0.472022715 | Down | Activity-dependent neuroprotector homeobox protein OS=Homo sapiens OX=9606 GN=ADNP PE=1 SV=1 | ADNP | _IHNFTSK[Acetyl (K)]C[Carbamidomethyl (C)]LYC[Carbamidomethyl (C)]NR_ |
| Q9H2P0 | 716 | K | 1.507467056 | Up | Activity-dependent neuroprotector homeobox protein OS=Homo sapiens OX=9606 GN=ADNP PE=1 SV=1 | ADNP | _LNQSPSLAPVK[Acetyl (K)]R_ |
| Q9H2W6 | 132 | K | 0.551623026 | Down | Large ribosomal subunit protein mL46 OS=Homo sapiens OX=9606 GN=MRPL46 PE=1 SV=1 | MRPL46 | _FLQFK[Acetyl (K)]LGAR_ |
| Q9H3F6 | 171 | K | 0.554094646 | Down | BTB/POZ domain-containing adapter for CUL3-mediated RhoA degradation protein 3 OS=Homo sapiens OX=9606 GN=KCTD10 PE=1 SV=1 | KCTD10 | _SNNK[Acetyl (K)]YSYTSNSDDNMLK_ |
| Q9H3K2 | 63 | K | 0.414929202 | Down | Growth hormone-inducible transmembrane protein OS=Homo sapiens OX=9606 GN=GHITM PE=1 SV=2 | GHITM | _TGQELK[Acetyl (K)]EAALEPSMEK_ |
| Q9H3U1 | 411 | K | 0.358669874 | Down | Protein unc-45 homolog A OS=Homo sapiens OX=9606 GN=UNC45A PE=1 SV=1 | UNC45A | _SWFEGQGLAGK[Acetyl (K)]LR_ |
| Q9H4G0 | 392 | K | 0.348928928 | Down | Band 4.1-like protein 1 OS=Homo sapiens OX=9606 GN=EPB41L1 PE=1 SV=2 | EPB41L1 | _GFLVMGSK[Acetyl (K)]FR_ |
| Q9H4H8 | 244 | K | 2.044570461 | Up | Protein FAM83D OS=Homo sapiens OX=9606 GN=FAM83D PE=1 SV=3 | FAM83D | _VHEK[Acetyl (K)]FTLIDGIR_ |
| Q9H4L5 | 13 | K | 0.384574995 | Down | Oxysterol-binding protein-related protein 3 OS=Homo sapiens OX=9606 GN=OSBPL3 PE=1 SV=1 | OSBPL3 | _NLGVSQK[Acetyl (K)]LVSPSR_ |
| Q9H4L7 | 621 | K | 1.675342562 | Up | SWI/SNF-related matrix-associated actin-dependent regulator of chromatin subfamily A containing DEAD/H box 1 OS=Homo sapiens OX=9606 GN=SMARCAD1 PE=1 SV=2 | SMARCAD1 | _LK[Acetyl (K)]LNYAIFDEGHMLK_ |
| Q9H501 | 48 | K | 0.620735575 | Down | ESF1 homolog OS=Homo sapiens OX=9606 GN=ESF1 PE=1 SV=1 | ESF1 | _FK[Acetyl (K)]LNYAVDK_ |
| Q9H501 | 103 | K | 0.484277299 | Down | ESF1 homolog OS=Homo sapiens OX=9606 GN=ESF1 PE=1 SV=1 | ESF1 | _K[Acetyl (K)]EIDSK[Acetyl (K)]NLVEK_ |
| Q9H501 | 108 | K | 0.484277299 | Down | ESF1 homolog OS=Homo sapiens OX=9606 GN=ESF1 PE=1 SV=1 | ESF1 | _K[Acetyl (K)]EIDSK[Acetyl (K)]NLVEK_ |
| Q9H501 | 138 | K | 0.502787347 | Down | ESF1 homolog OS=Homo sapiens OX=9606 GN=ESF1 PE=1 SV=1 | ESF1 | _TDLDNSIGIK[Acetyl (K)]K_ |
| Q9H6R4 | 54 | K | 2.999908889 | Up | Nucleolar protein 6 OS=Homo sapiens OX=9606 GN=NOL6 PE=1 SV=2 | NOL6 | _GLLQPVK[Acetyl (K)]LSR_ |
| Q9H6Y2 | 85 | K | 0.485302124 | Down | WD repeat-containing protein 55 OS=Homo sapiens OX=9606 GN=WDR55 PE=1 SV=2 | WDR55 | _ELWSSGHHLK[Acetyl (K)]AC[Carbamidomethyl (C)]R_ |
| Q9H777 | 218 | K | 2.226561339 | Up | Zinc phosphodiesterase ELAC protein 1 OS=Homo sapiens OX=9606 GN=ELAC1 PE=1 SV=2 | ELAC1 | _DLGVPPGPAYGK[Acetyl (K)]LK_ |
| Q9H7B2 | 47 | K | 1.627465622 | Up | Ribosome production factor 2 homolog OS=Homo sapiens OX=9606 GN=RPF2 PE=1 SV=2 | RPF2 | _GGNANATVTK[Acetyl (K)]VLK_ |
| Q9H7B2 | 275 | K | 0.59616673 | Down | Ribosome production factor 2 homolog OS=Homo sapiens OX=9606 GN=RPF2 PE=1 SV=2 | RPF2 | _QDLSK[Acetyl (K)]LQTR_ |
| Q9H845 | 47 | K | 2.010149129 | Up | Complex I assembly factor ACAD9, mitochondrial OS=Homo sapiens OX=9606 GN=ACAD9 PE=1 SV=1 | ACAD9 | _ELFLGK[Acetyl (K)]IK_ |
| Q9H8E8 | 292 | K | 1.861341506 | Up | Cysteine-rich protein 2-binding protein OS=Homo sapiens OX=9606 GN=KAT14 PE=1 SV=3 | KAT14 | _STSSTPVK[Acetyl (K)]FISR_ |
| Q9H8K7 | 147 | K | 0.502312546 | Down | ATPase PAAT OS=Homo sapiens OX=9606 GN=PAAT PE=1 SV=2 | PAAT | _IK[Acetyl (K)]LLSFGER_ |
| Q9H8Y8 | 162 | K | 1.6075047 | Up | Golgi reassembly-stacking protein 2 OS=Homo sapiens OX=9606 GN=GORASP2 PE=1 SV=3 | GORASP2 | _PLK[Acetyl (K)]LYVYNTDTDNC[Carbamidomethyl (C)]R_ |
| Q9H974 | 7 | K | 0.63349544 | Down | Queuine tRNA-ribosyltransferase accessory subunit 2 OS=Homo sapiens OX=9606 GN=QTRT2 PE=1 SV=1 | QTRT2 | _LSLTK[Acetyl (K)]VVNGC[Carbamidomethyl (C)]R_ |
| Q9H981 | 141 | K | 0.551572166 | Down | Actin-related protein 8 OS=Homo sapiens OX=9606 GN=ACTR8 PE=1 SV=2 | ACTR8 | _SYNK[Acetyl (K)]QMR_ |
| Q9H9A6 | 132 | K | 2.101324405 | Up | Leucine-rich repeat-containing protein 40 OS=Homo sapiens OX=9606 GN=LRRC40 PE=1 SV=1 | LRRC40 | _ELENLQK[Acetyl (K)]LNVSHNK_ |
| Q9H9B1 | 439 | K | 2.251563666 | Up | Histone-lysine N-methyltransferase EHMT1 OS=Homo sapiens OX=9606 GN=EHMT1 PE=1 SV=4 | EHMT1 | _TDSPWIK[Acetyl (K)]PAR_ |
| Q9HAF1 | 6 | K | 1.503321626 | Up | Chromatin modification-related protein MEAF6 OS=Homo sapiens OX=9606 GN=MEAF6 PE=1 SV=1 | MEAF6 | _[Acetyl (Protein N-term)]AMHNK[Acetyl (K)]AAPPQIPDTR_ |
| Q9HAV7 | 162 | K | 1.518885149 | Up | GrpE protein homolog 1, mitochondrial OS=Homo sapiens OX=9606 GN=GRPEL1 PE=1 SV=2 | GRPEL1 | _HGLLK[Acetyl (K)]LNPVGAK_ |
| Q9HAV7 | 196 | K | 0.369265678 | Down | GrpE protein homolog 1, mitochondrial OS=Homo sapiens OX=9606 GN=GRPEL1 PE=1 SV=2 | GRPEL1 | _EPGTVALVSK[Acetyl (K)]VGYK_ |
| Q9HB07 | 40 | K | 3.159424863 | Up | MYG1 exonuclease OS=Homo sapiens OX=9606 GN=MYG1 PE=1 SV=3 | MYG1 | _SK[Acetyl (K)]LMAPPR_ |
| Q9HB71 | 85 | K | 0.451100717 | Down | Calcyclin-binding protein OS=Homo sapiens OX=9606 GN=CACYBP PE=1 SV=2 | CACYBP | _ISNYGWDQSDK[Acetyl (K)]FVK_ |
| Q9HBI6 | 177 | K | 0.661149928 | Down | Cytochrome P450 4F11 OS=Homo sapiens OX=9606 GN=CYP4F11 PE=1 SV=3 | CYP4F11 | _SVNIMHDK[Acetyl (K)]WQR_ |
| Q9HC35 | 203 | K | 0.549195705 | Down | Echinoderm microtubule-associated protein-like 4 OS=Homo sapiens OX=9606 GN=EML4 PE=1 SV=3 | EML4 | _IPSTPK[Acetyl (K)]LIPK_ |
| Q9HC36 | 153 | K | 1.996076847 | Up | rRNA methyltransferase 3, mitochondrial OS=Homo sapiens OX=9606 GN=MRM3 PE=1 SV=2 | MRM3 | _LEYLK[Acetyl (K)]ELPVDK_ |
| Q9HC38 | 12 | K | 0.499641906 | Down | Glyoxalase domain-containing protein 4 OS=Homo sapiens OX=9606 GN=GLOD4 PE=1 SV=1 | GLOD4 | _ALHFVFK[Acetyl (K)]VGNR_ |
| Q9HC38 | 305 | K | 0.432704452 | Down | Glyoxalase domain-containing protein 4 OS=Homo sapiens OX=9606 GN=GLOD4 PE=1 SV=1 | GLOD4 | _SDEWFAK[Acetyl (K)]HNKPK_ |
| Q9HCK8 | 542 | K | 1.913849717 | Up | Chromodomain-helicase-DNA-binding protein 8 OS=Homo sapiens OX=9606 GN=CHD8 PE=1 SV=5 | CHD8 | _LNTITPVVGK[Acetyl (K)]K_ |
| Q9HCM4 | 223 | K | 0.593415827 | Down | Band 4.1-like protein 5 OS=Homo sapiens OX=9606 GN=EPB41L5 PE=1 SV=3 | EPB41L5 | _GQTPAQAETNYLNK[Acetyl (K)]AK_ |
| Q9HCM4 | 350 | K | 0.312284109 | Down | Band 4.1-like protein 5 OS=Homo sapiens OX=9606 GN=EPB41L5 PE=1 SV=3 | EPB41L5 | _YSGK[Acetyl (K)]TEYQTTK_ |
| Q9HCN8 | 43 | K | 0.38576707 | Down | Stromal cell-derived factor 2-like protein 1 OS=Homo sapiens OX=9606 GN=SDF2L1 PE=1 SV=2 | SDF2L1 | _TGAELVTC[Carbamidomethyl (C)]GSVLK[Acetyl (K)]LLNTHHR_ |
| Q9HD33 | 144 | K | 0.379559989 | Down | Large ribosomal subunit protein uL29m OS=Homo sapiens OX=9606 GN=MRPL47 PE=1 SV=2 | MRPL47 | _VVDSMDALDK[Acetyl (K)]VVQER_ |
| Q9HD42 | 107 | K | 0.578425195 | Down | Charged multivesicular body protein 1a OS=Homo sapiens OX=9606 GN=CHMP1A PE=1 SV=1 | CHMP1A | _ALSTMDLQK[Acetyl (K)]VSSVMDR_ |
| Q9HD45 | 257 | K | 0.429590057 | Down | Transmembrane 9 superfamily member 3 OS=Homo sapiens OX=9606 GN=TM9SF3 PE=1 SV=2 | TM9SF3 | _YSK[Acetyl (K)]EEEMDDMDR_ |
| Q9HDC9 | 347 | K | 0.56435228 | Down | Adipocyte plasma membrane-associated protein OS=Homo sapiens OX=9606 GN=APMAP PE=1 SV=2 | APMAP | _MIFK[Acetyl (K)]LFSQETVMK_ |
| Q9NP64 | 70 | K | 1.725166383 | Up | Zinc finger CCHC domain-containing protein 17 OS=Homo sapiens OX=9606 GN=ZCCHC17 PE=1 SV=1 | ZCCHC17 | _VWVK[Acetyl (K)]LIGR_ |
| Q9NP64 | 144 | K | 0.234747454 | Down | Zinc finger CCHC domain-containing protein 17 OS=Homo sapiens OX=9606 GN=ZCCHC17 PE=1 SV=1 | ZCCHC17 | _GHFAK[Acetyl (K)]DC[Carbamidomethyl (C)]FMQPGGTK_ |
| Q9NP79 | 300 | K | 0.654448544 | Down | Vacuolar protein sorting-associated protein VTA1 homolog OS=Homo sapiens OX=9606 GN=VTA1 PE=1 SV=1 | VTA1 | _ALK[Acetyl (K)]LLTTGR_ |
| Q9NPA8 | 19 | K | 2.819876915 | Up | Transcription and mRNA export factor ENY2 OS=Homo sapiens OX=9606 GN=ENY2 PE=1 SV=1 | ENY2 | _AAINQK[Acetyl (K)]LIETGER_ |
| Q9NPJ3 | 27 | K | 3.339718418 | Up | Acyl-coenzyme A thioesterase 13 OS=Homo sapiens OX=9606 GN=ACOT13 PE=1 SV=1 | ACOT13 | _VLGK[Acetyl (K)]ITLVSAAPGK_ |
| Q9NPQ8 | 482 | K | 0.496900413 | Down | Synembryn-A OS=Homo sapiens OX=9606 GN=RIC8A PE=1 SV=3 | RIC8A | _EHEAMK[Acetyl (K)]LVTMFDK_ |
| Q9NQ29 | 357 | K | 0.516972657 | Down | Putative RNA-binding protein Luc7-like 1 OS=Homo sapiens OX=9606 GN=LUC7L PE=1 SV=1 | LUC7L | _LESSNGK[Acetyl (K)]MASR_ |
| Q9NQ34 | 180 | K | 0.586277255 | Down | Transmembrane protein 9B OS=Homo sapiens OX=9606 GN=TMEM9B PE=1 SV=1 | TMEM9B | _WK[Acetyl (K)]LQVQEQR_ |
| Q9NQ55 | 98 | K | 0.252340234 | Down | Suppressor of SWI4 1 homolog OS=Homo sapiens OX=9606 GN=PPAN PE=1 SV=1 | PPAN | _TETNVYFK[Acetyl (K)]LMR_ |
| Q9NQ88 | 77 | K | 1.77523535 | Up | Fructose-2,6-bisphosphatase TIGAR OS=Homo sapiens OX=9606 GN=TIGAR PE=1 SV=1 | TIGAR | _FC[Carbamidomethyl (C)]K[Acetyl (K)]DMTVK_ |
| Q9NQC1 | 32 | K | 1.889330135 | Up | E3 ubiquitin-protein ligase Jade-2 OS=Homo sapiens OX=9606 GN=JADE2 PE=1 SV=2 | JADE2 | _C[Carbamidomethyl (C)]SK[Acetyl (K)]LPSSTK[Acetyl (K)]SGWPR_ |
| Q9NQC1 | 38 | K | 1.796372953 | Up | E3 ubiquitin-protein ligase Jade-2 OS=Homo sapiens OX=9606 GN=JADE2 PE=1 SV=2 | JADE2 | _LPSSTK[Acetyl (K)]SGWPR_ |
| Q9NQC1 | 298 | K | 3.013434708 | Up | E3 ubiquitin-protein ligase Jade-2 OS=Homo sapiens OX=9606 GN=JADE2 PE=1 SV=2 | JADE2 | _MEPITK[Acetyl (K)]ISHIPASR_ |
| Q9NQC3 | 1179 | K | 0.664753818 | Down | Reticulon-4 OS=Homo sapiens OX=9606 GN=RTN4 PE=1 SV=2 | RTN4 | _DAM[Oxidation (M)]AK[Acetyl (K)]IQAK_ |
| Q9NQY0 | 30 | K | 2.978612372 | Up | Bridging integrator 3 OS=Homo sapiens OX=9606 GN=BIN3 PE=1 SV=1 | BIN3 | _EYGK[Acetyl (K)]LQQLEEQTR_ |
| Q9NQZ2 | 310 | K | 0.383128841 | Down | Something about silencing protein 10 OS=Homo sapiens OX=9606 GN=UTP3 PE=1 SV=1 | UTP3 | _NLINK[Acetyl (K)]LSVVDQK_ |
| Q9NR19 | 405 | K | 0.650848551 | Down | Acetyl-coenzyme A synthetase, cytoplasmic OS=Homo sapiens OX=9606 GN=ACSS2 PE=1 SV=1 | ACSS2 | _VTK[Acetyl (K)]FYTAPTAIR_ |
| Q9NR30 | 224 | K | 0.628628852 | Down | Nucleolar RNA helicase 2 OS=Homo sapiens OX=9606 GN=DDX21 PE=1 SV=5 | DDX21 | _TFHHVYSGK[Acetyl (K)]DLIAQAR_ |
| Q9NR45 | 290 | K | 0.61307256 | Down | Sialic acid synthase OS=Homo sapiens OX=9606 GN=NANS PE=1 SV=2 | NANS | _QLLPC[Carbamidomethyl (C)]EMAC[Carbamidomethyl (C)]NEK[Acetyl (K)]LGK_ |
| Q9NR48 | 1815 | K | 3.812564727 | Up | Histone-lysine N-methyltransferase ASH1L OS=Homo sapiens OX=9606 GN=ASH1L PE=1 SV=2 | ASH1L | _MC[Carbamidomethyl (C)]NYDK[Acetyl (K)]ILATK_ |
| Q9NR48 | 1820 | K | 8.100800082 | Up | Histone-lysine N-methyltransferase ASH1L OS=Homo sapiens OX=9606 GN=ASH1L PE=1 SV=2 | ASH1L | _MC[Carbamidomethyl (C)]NYDK[Acetyl (K)]ILATK[Acetyl (K)]K_ |
| Q9NRF8 | 360 | K | 0.203146834 | Down | CTP synthase 2 OS=Homo sapiens OX=9606 GN=CTPS2 PE=1 SV=1 | CTPS2 | _FHEAWQK[Acetyl (K)]LC[Carbamidomethyl (C)]K_ |
| Q9NRL2 | 229 | K | 0.611803707 | Down | Bromodomain adjacent to zinc finger domain protein 1A OS=Homo sapiens OX=9606 GN=BAZ1A PE=1 SV=2 | BAZ1A | _LFLK[Acetyl (K)]QHC[Carbamidomethyl (C)]EPQDGVIK_ |
| Q9NRL2 | 1396 | K | 2.640491525 | Up | Bromodomain adjacent to zinc finger domain protein 1A OS=Homo sapiens OX=9606 GN=BAZ1A PE=1 SV=2 | BAZ1A | _SVNIASK[Acetyl (K)]LSLQESESK_ |
| Q9NRL3 | 469 | K | 0.358564545 | Down | Striatin-4 OS=Homo sapiens OX=9606 GN=STRN4 PE=1 SV=2 | STRN4 | _LWNLQK[Acetyl (K)]AVTAK_ |
| Q9NRZ5 | 281 | K | 0.500653754 | Down | 1-acyl-sn-glycerol-3-phosphate acyltransferase delta OS=Homo sapiens OX=9606 GN=AGPAT4 PE=1 SV=1 | AGPAT4 | _LYQEK[Acetyl (K)]DAFQEEYYR_ |
| Q9NRZ9 | 349 | K | 1.720743282 | Up | Lymphoid-specific helicase OS=Homo sapiens OX=9606 GN=HELLS PE=1 SV=1 | HELLS | _NALQHC[Carbamidomethyl (C)]YWK[Acetyl (K)]YLIVDEGHR_ |
| Q9NSE4 | 233 | K | 0.49859511 | Down | Isoleucine--tRNA ligase, mitochondrial OS=Homo sapiens OX=9606 GN=IARS2 PE=1 SV=2 | IARS2 | _TFYQMYDK[Acetyl (K)]GLVYR_ |
| Q9NSU2 | 66 | K | 1.558272859 | Up | Three-prime repair exonuclease 1 OS=Homo sapiens OX=9606 GN=TREX1 PE=1 SV=2 | TREX1 | _VVDK[Acetyl (K)]LSLC[Carbamidomethyl (C)]VAPGK_ |
| Q9NTI5 | 335 | K | 1.590186868 | Up | Sister chromatid cohesion protein PDS5 homolog B OS=Homo sapiens OX=9606 GN=PDS5B PE=1 SV=1 | PDS5B | _LEC[Carbamidomethyl (C)]VK[Acetyl (K)]FASHC[Carbamidomethyl (C)]LMNHPDLAK_ |
| Q9NTJ3 | 898 | K | 0.600649861 | Down | Structural maintenance of chromosomes protein 4 OS=Homo sapiens OX=9606 GN=SMC4 PE=1 SV=2 | SMC4 | _LHNTIVEINNHK[Acetyl (K)]LK_ |
| Q9NTK5 | 79 | K | 0.529779595 | Down | Obg-like ATPase 1 OS=Homo sapiens OX=9606 GN=OLA1 PE=1 SV=2 | OLA1 | _FDFLC[Carbamidomethyl (C)]QYHK[Acetyl (K)]PASK_ |
| Q9NTK5 | 333 | K | 2.484735897 | Up | Obg-like ATPase 1 OS=Homo sapiens OX=9606 GN=OLA1 PE=1 SV=2 | OLA1 | _APQAAGK[Acetyl (K)]IHTDFEK_ |
| Q9NUQ9 | 89 | K | 0.598480457 | Down | CYFIP-related Rac1 interactor B OS=Homo sapiens OX=9606 GN=CYRIB PE=1 SV=1 | CYRIB | _AWGAVVPLVGK[Acetyl (K)]LK_ |
| Q9NUQ9 | 92 | K | 0.493115173 | Down | CYFIP-related Rac1 interactor B OS=Homo sapiens OX=9606 GN=CYRIB PE=1 SV=1 | CYRIB | _K[Acetyl (K)]FYEFSQR_ |
| Q9NUQ9 | 286 | K | 0.665643853 | Down | CYFIP-related Rac1 interactor B OS=Homo sapiens OX=9606 GN=CYRIB PE=1 SV=1 | CYRIB | _GC[Carbamidomethyl (C)]IK[Acetyl (K)]VLK_ |
| Q9NUQ9 | 308 | K | 0.239474977 | Down | CYFIP-related Rac1 interactor B OS=Homo sapiens OX=9606 GN=CYRIB PE=1 SV=1 | CYRIB | _YTTK[Acetyl (K)]HLNDETTSK_ |
| Q9NUU7 | 460 | K | 0.283110954 | Down | ATP-dependent RNA helicase DDX19A OS=Homo sapiens OX=9606 GN=DDX19A PE=1 SV=1 | DDX19A | _IQEHFNK[Acetyl (K)]K_ |
| Q9NV06 | 49 | K | 3.292919765 | Up | DDB1- and CUL4-associated factor 13 OS=Homo sapiens OX=9606 GN=DCAF13 PE=1 SV=2 | DCAF13 | _ALNATK[Acetyl (K)]LER_ |
| Q9NV06 | 359 | K | 0.629552827 | Down | DDB1- and CUL4-associated factor 13 OS=Homo sapiens OX=9606 GN=DCAF13 PE=1 SV=2 | DCAF13 | _ANASEK[Acetyl (K)]LGVLTSR_ |
| Q9NV56 | 185 | K | 0.413612957 | Down | MRG/MORF4L-binding protein OS=Homo sapiens OX=9606 GN=MRGBP PE=1 SV=1 | MRGBP | _VTDK[Acetyl (K)]VLTANSNPSSPSAAK_ |
| Q9NV66 | 190 | K | 0.532678502 | Down | S-adenosyl-L-methionine-dependent tRNA 4-demethylwyosine synthase TYW1 OS=Homo sapiens OX=9606 GN=TYW1 PE=2 SV=2 | TYW1 | _YAVFGLGNSAYASHFNK[Acetyl (K)]VGK_ |
| Q9NV66 | 396 | K | 1.516470203 | Up | S-adenosyl-L-methionine-dependent tRNA 4-demethylwyosine synthase TYW1 OS=Homo sapiens OX=9606 GN=TYW1 PE=2 SV=2 | TYW1 | _GGC[Carbamidomethyl (C)]YK[Acetyl (K)]HTFYGIESHR_ |
| Q9NVF7 | 263 | K | 2.07278903 | Up | F-box only protein 28 OS=Homo sapiens OX=9606 GN=FBXO28 PE=1 SV=1 | FBXO28 | _QEVTK[Acetyl (K)]LQQQVK_ |
| Q9NVH6 | 236 | K | 2.382245903 | Up | Trimethyllysine dioxygenase, mitochondrial OS=Homo sapiens OX=9606 GN=TMLHE PE=1 SV=1 | TMLHE | _GDTAYTK[Acetyl (K)]LALDR_ |
| Q9NVH6 | 373 | K | 1.691827808 | Up | Trimethyllysine dioxygenase, mitochondrial OS=Homo sapiens OX=9606 GN=TMLHE PE=1 SV=1 | TMLHE | _RPENEFWVK[Acetyl (K)]LKPGR_ |
| Q9NVI7 | 539 | K | 1.504008645 | Up | ATPase family AAA domain-containing protein 3A OS=Homo sapiens OX=9606 GN=ATAD3A PE=1 SV=2 | ATAD3A | _MYFDK[Acetyl (K)]YVLKPATEGK_ |
| Q9NVI7 | 616 | K | 0.611623469 | Down | ATPase family AAA domain-containing protein 3A OS=Homo sapiens OX=9606 GN=ATAD3A PE=1 SV=2 | ATAD3A | _VQDAVQQHQQK[Acetyl (K)]MC[Carbamidomethyl (C)]WLK_ |
| Q9NVV4 | 152 | K | 0.524537953 | Down | Poly(A) RNA polymerase, mitochondrial OS=Homo sapiens OX=9606 GN=MTPAP PE=1 SV=1 | MTPAP | _FFNLK[Acetyl (K)]LK_ |
| Q9NW64 | 76 | K | 0.191216293 | Down | Pre-mRNA-splicing factor RBM22 OS=Homo sapiens OX=9606 GN=RBM22 PE=1 SV=1 | RBM22 | _TEVC[Carbamidomethyl (C)]QTC[Carbamidomethyl (C)]SK[Acetyl (K)]LK_ |
| Q9NW68 | 193 | K | 1.69261344 | Up | BSD domain-containing protein 1 OS=Homo sapiens OX=9606 GN=BSDC1 PE=1 SV=1 | BSDC1 | _YFYK[Acetyl (K)]VHQLEQEQAR_ |
| Q9NW82 | 368 | K | 1.520888293 | Up | WD repeat-containing protein 70 OS=Homo sapiens OX=9606 GN=WDR70 PE=1 SV=1 | WDR70 | _NLTVHPK[Acetyl (K)]FHYK_ |
| Q9NW82 | 509 | K | 1.825298431 | Up | WD repeat-containing protein 70 OS=Homo sapiens OX=9606 GN=WDR70 PE=1 SV=1 | WDR70 | _GAK[Acetyl (K)]LC[Carbamidomethyl (C)]VVK_ |
| Q9NWR8 | 140 | K | 2.404902426 | Up | Calcium uniporter regulatory subunit MCUb, mitochondrial OS=Homo sapiens OX=9606 GN=MCUB PE=1 SV=2 | MCUB | _LVINK[Acetyl (K)]IAYDVQC[Carbamidomethyl (C)]PK_ |
| Q9NWR8 | 304 | K | 2.001727768 | Up | Calcium uniporter regulatory subunit MCUb, mitochondrial OS=Homo sapiens OX=9606 GN=MCUB PE=1 SV=2 | MCUB | _QQHFDVQQYNK[Acetyl (K)]LK_ |
| Q9NWU5 | 50 | K | 0.488250597 | Down | Large ribosomal subunit protein uL22m OS=Homo sapiens OX=9606 GN=MRPL22 PE=1 SV=1 | MRPL22 | _NK[Acetyl (K)]IVYPPQLPGEPR_ |
| Q9NX58 | 14 | K | 0.64385798 | Down | Cell growth-regulating nucleolar protein OS=Homo sapiens OX=9606 GN=LYAR PE=1 SV=3 | LYAR | _VFFTC[Carbamidomethyl (C)]NAC[Carbamidomethyl (C)]GESVK[Acetyl (K)]K_ |
| Q9NX58 | 113 | K | 0.481019023 | Down | Cell growth-regulating nucleolar protein OS=Homo sapiens OX=9606 GN=LYAR PE=1 SV=3 | LYAR | _AK[Acetyl (K)]FQNWMK_ |
| Q9NX63 | 63 | K | 0.539831409 | Down | MICOS complex subunit MIC19 OS=Homo sapiens OX=9606 GN=CHCHD3 PE=1 SV=1 | CHCHD3 | _YSGAYGASVSDEELK[Acetyl (K)]R_ |
| Q9NXC5 | 272 | K | 0.618417056 | Down | GATOR2 complex protein MIOS OS=Homo sapiens OX=9606 GN=MIOS PE=1 SV=2 | MIOS | _PLTK[Acetyl (K)]VAWC[Carbamidomethyl (C)]PTR_ |
| Q9NXV6 | 533 | K | 0.519713003 | Down | CDKN2A-interacting protein OS=Homo sapiens OX=9606 GN=CDKN2AIP PE=1 SV=3 | CDKN2AIP | _EALK[Acetyl (K)]LFLK_ |
| Q9NXW2 | 103 | K | 0.651838285 | Down | DnaJ homolog subfamily B member 12 OS=Homo sapiens OX=9606 GN=DNAJB12 PE=1 SV=5 | DNAJB12 | _GYTAEQVAAVK[Acetyl (K)]R_ |
| Q9NXW2 | 136 | K | 1.74712584 | Up | DnaJ homolog subfamily B member 12 OS=Homo sapiens OX=9606 GN=DNAJB12 PE=1 SV=5 | DNAJB12 | _LALK[Acetyl (K)]FHPDK_ |
| Q9NY12 | 143 | K | 0.267356571 | Down | H/ACA ribonucleoprotein complex subunit 1 OS=Homo sapiens OX=9606 GN=GAR1 PE=1 SV=1 | GAR1 | _LQK[Acetyl (K)]FYIDPYK_ |
| Q9NY61 | 496 | K | 0.664884235 | Down | Protein AATF OS=Homo sapiens OX=9606 GN=AATF PE=1 SV=1 | AATF | _QWLAIQK[Acetyl (K)]LR_ |
| Q9NYB0 | 208 | K | 1.999036669 | Up | Telomeric repeat-binding factor 2-interacting protein 1 OS=Homo sapiens OX=9606 GN=TERF2IP PE=1 SV=1 | TERF2IP | _YLLGDAPVSPSSQK[Acetyl (K)]LK_ |
| Q9NYB9 | 123 | K | 1.778649226 | Up | Abl interactor 2 OS=Homo sapiens OX=9606 GN=ABI2 PE=1 SV=1 | ABI2 | _THK[Acetyl (K)]IIAPANLER_ |
| Q9NYK5 | 234 | K | 1.66416614 | Up | Large ribosomal subunit protein mL39 OS=Homo sapiens OX=9606 GN=MRPL39 PE=1 SV=3 | MRPL39 | _VALEIFQHSK[Acetyl (K)]YK_ |
| Q9NYT0 | 323 | K | 0.641114562 | Down | Pleckstrin-2 OS=Homo sapiens OX=9606 GN=PLEK2 PE=1 SV=1 | PLEK2 | _GNVQGNLFK[Acetyl (K)]VITK_ |
| Q9NYU2 | 1027 | K | 1.54277805 | Up | UDP-glucose:glycoprotein glucosyltransferase 1 OS=Homo sapiens OX=9606 GN=UGGT1 PE=1 SV=3 | UGGT1 | _VFMNC[Carbamidomethyl (C)]QSK[Acetyl (K)]LSDMPLK_ |
| Q9NZI5 | 418 | K | 0.343006516 | Down | Grainyhead-like protein 1 homolog OS=Homo sapiens OX=9606 GN=GRHL1 PE=1 SV=2 | GRHL1 | _AYC[Carbamidomethyl (C)]QIK[Acetyl (K)]VFC[Carbamidomethyl (C)]DK_ |
| Q9NZM1 | 437 | K | 1.790664802 | Up | Myoferlin OS=Homo sapiens OX=9606 GN=MYOF PE=1 SV=1 | MYOF | _FPSVC[Carbamidomethyl (C)]EK[Acetyl (K)]IK_ |
| Q9NZM1 | 1956 | K | 0.535725561 | Down | Myoferlin OS=Homo sapiens OX=9606 GN=MYOF PE=1 SV=1 | MYOF | _GWWPC[Carbamidomethyl (C)]YAEK[Acetyl (K)]DGAR_ |
| Q9NZM3 | 335 | K | 1.585613192 | Up | Intersectin-2 OS=Homo sapiens OX=9606 GN=ITSN2 PE=1 SV=3 | ITSN2 | _GGK[Acetyl (K)]QIDSINGTLPSYQK_ |
| Q9P0L0 | 38 | K | 0.620124661 | Down | Vesicle-associated membrane protein-associated protein A OS=Homo sapiens OX=9606 GN=VAPA PE=1 SV=3 | VAPA | _GPFTDVVTTNLK[Acetyl (K)]LR_ |
| Q9P0M6 | 323 | K | 0.64016582 | Down | Core histone macro-H2A.2 OS=Homo sapiens OX=9606 GN=MACROH2A2 PE=1 SV=3 | MACROH2A2 | _NC[Carbamidomethyl (C)]FPK[Acetyl (K)]QTAAQVTLK_ |
| Q9P0U4 | 250 | K | 1.857119663 | Up | CXXC-type zinc finger protein 1 OS=Homo sapiens OX=9606 GN=CXXC1 PE=1 SV=2 | CXXC1 | _RPLPTQQQPQPSQK[Acetyl (K)]LGR_ |
| Q9P265 | 276 | K | 1.507163243 | Up | Disco-interacting protein 2 homolog B OS=Homo sapiens OX=9606 GN=DIP2B PE=1 SV=3 | DIP2B | _VSTK[Acetyl (K)]IQQLLNTLK_ |
| Q9P275 | 39 | K | 2.022596723 | Up | Ubiquitin carboxyl-terminal hydrolase 36 OS=Homo sapiens OX=9606 GN=USP36 PE=1 SV=4 | USP36 | _VLLQK[Acetyl (K)]IEFEPASK_ |
| Q9P275 | 230 | K | 1.510931209 | Up | Ubiquitin carboxyl-terminal hydrolase 36 OS=Homo sapiens OX=9606 GN=USP36 PE=1 SV=4 | USP36 | _AC[Carbamidomethyl (C)]LNGC[Carbamidomethyl (C)]AK[Acetyl (K)]LDR_ |
| Q9P275 | 338 | K | 1.741814658 | Up | Ubiquitin carboxyl-terminal hydrolase 36 OS=Homo sapiens OX=9606 GN=USP36 PE=1 SV=4 | USP36 | _FANFSGGK[Acetyl (K)]ITK_ |
| Q9P2J5 | 198 | K | 0.596605786 | Down | Leucine--tRNA ligase, cytoplasmic OS=Homo sapiens OX=9606 GN=LARS1 PE=1 SV=2 | LARS1 | _MGLK[Acetyl (K)]VDWR_ |
| Q9P2J5 | 270 | K | 0.640338683 | Down | Leucine--tRNA ligase, cytoplasmic OS=Homo sapiens OX=9606 GN=LARS1 PE=1 SV=2 | LARS1 | _LK[Acetyl (K)]VLEPYPSK_ |
| Q9P2K8 | 610 | K | 0.537169487 | Down | eIF-2-alpha kinase GCN2 OS=Homo sapiens OX=9606 GN=EIF2AK4 PE=1 SV=3 | EIF2AK4 | _VQNK[Acetyl (K)]LDGC[Carbamidomethyl (C)]C[Carbamidomethyl (C)]YAVK_ |
| Q9P2N5 | 601 | K | 2.193281815 | Up | RNA-binding protein 27 OS=Homo sapiens OX=9606 GN=RBM27 PE=1 SV=2 | RBM27 | _NQYTNTK[Acetyl (K)]LEVK_ |
| Q9P2N5 | 616 | K | 2.455310352 | Up | RNA-binding protein 27 OS=Homo sapiens OX=9606 GN=RBM27 PE=1 SV=2 | RBM27 | _IPQELNNITK[Acetyl (K)]LNEHFSK_ |
| Q9P2W1 | 99 | K | 1.984286262 | Up | Homologous-pairing protein 2 homolog OS=Homo sapiens OX=9606 GN=PSMC3IP PE=1 SV=1 | PSMC3IP | _IVALTAK[Acetyl (K)]VQSLQQSC[Carbamidomethyl (C)]R_ |
| Q9UBF6 | 28 | K | 0.465820988 | Down | RING-box protein 2 OS=Homo sapiens OX=9606 GN=RNF7 PE=1 SV=1 | RNF7 | _SGGDK[Acetyl (K)]MFSLK_ |
| Q9UBH6 | 68 | K | 0.467335989 | Down | Solute carrier family 53 member 1 OS=Homo sapiens OX=9606 GN=XPR1 PE=1 SV=1 | XPR1 | _ELAK[Acetyl (K)]INTFYSEK_ |
| Q9UBH6 | 76 | K | 0.376287587 | Down | Solute carrier family 53 member 1 OS=Homo sapiens OX=9606 GN=XPR1 PE=1 SV=1 | XPR1 | _INTFYSEK[Acetyl (K)]LAEAQR_ |
| Q9UBL3 | 216 | K | 2.011967947 | Up | Set1/Ash2 histone methyltransferase complex subunit ASH2 OS=Homo sapiens OX=9606 GN=ASH2L PE=1 SV=1 | ASH2L | _QRPGK[Acetyl (K)]MTWPNNIVK_ |
| Q9UBL3 | 467 | K | 0.572846654 | Down | Set1/Ash2 histone methyltransferase complex subunit ASH2 OS=Homo sapiens OX=9606 GN=ASH2L PE=1 SV=1 | ASH2L | _GTK[Acetyl (K)]FHQSIGK_ |
| Q9UBQ0 | 43 | K | 0.424533359 | Down | Vacuolar protein sorting-associated protein 29 OS=Homo sapiens OX=9606 GN=VPS29 PE=1 SV=1 | VPS29 | _IQHILC[Carbamidomethyl (C)]TGNLC[Carbamidomethyl (C)]TK[Acetyl (K)]ESYDYLK_ |
| Q9UBS4 | 350 | K | 1.764301301 | Up | DnaJ homolog subfamily B member 11 OS=Homo sapiens OX=9606 GN=DNAJB11 PE=1 SV=1 | DNAJB11 | _QGSVQK[Acetyl (K)]VYNGLQGY_ |
| Q9UBT2 | 271 | K | 0.281821635 | Down | SUMO-activating enzyme subunit 2 OS=Homo sapiens OX=9606 GN=UBA2 PE=1 SV=2 | UBA2 | _YLLTMDK[Acetyl (K)]LWR_ |
| Q9UBV2 | 248 | K | 1.665377963 | Up | Protein sel-1 homolog 1 OS=Homo sapiens OX=9606 GN=SEL1L PE=1 SV=3 | SEL1L | _EMFEK[Acetyl (K)]LTEEGSPK_ |
| Q9UDY4 | 177 | K | 0.651754629 | Down | DnaJ homolog subfamily B member 4 OS=Homo sapiens OX=9606 GN=DNAJB4 PE=1 SV=1 | DNAJB4 | _VSLEEIYSGC[Carbamidomethyl (C)]TK[Acetyl (K)]R_ |
| Q9UEE9 | 230 | K | 3.246903737 | Up | Craniofacial development protein 1 OS=Homo sapiens OX=9606 GN=CFDP1 PE=1 SV=1 | CFDP1 | _SSGMSSLLGK[Acetyl (K)]IGAK_ |
| Q9UET6 | 28 | K | 0.53297347 | Down | tRNA (cytidine(32)/guanosine(34)-2'-O)-methyltransferase OS=Homo sapiens OX=9606 GN=FTSJ1 PE=1 SV=2 | FTSJ1 | _SAFK[Acetyl (K)]LLQLDK_ |
| Q9UEU0 | 13 | K | 1.718152085 | Up | Vesicle transport through interaction with t-SNAREs homolog 1B OS=Homo sapiens OX=9606 GN=VTI1B PE=1 SV=3 | VTI1B | _[Acetyl (Protein N-term)]ASSAASSEHFEK[Acetyl (K)]LHEIFR_ |
| Q9UEW8 | 312 | K | 1.507156633 | Up | STE20/SPS1-related proline-alanine-rich protein kinase OS=Homo sapiens OX=9606 GN=STK39 PE=1 SV=3 | STK39 | _K[Acetyl (K)]LLSLC[Carbamidomethyl (C)]LQK_ |
| Q9UEW8 | 512 | K | 1.527585145 | Up | STE20/SPS1-related proline-alanine-rich protein kinase OS=Homo sapiens OX=9606 GN=STK39 PE=1 SV=3 | STK39 | _IVDDPK[Acetyl (K)]ALK_ |
| Q9UFC0 | 151 | K | 1.517267859 | Up | Leucine-rich repeat and WD repeat-containing protein 1 OS=Homo sapiens OX=9606 GN=LRWD1 PE=1 SV=2 | LRWD1 | _VTAHWEK[Acetyl (K)]FMATLGPEEEAEK_ |
| Q9UGR2 | 726 | K | 0.197763484 | Down | Zinc finger CCCH domain-containing protein 7B OS=Homo sapiens OX=9606 GN=ZC3H7B PE=1 SV=2 | ZC3H7B | _HC[Carbamidomethyl (C)]WTK[Acetyl (K)]ER_ |
| Q9UH62 | 260 | K | 0.52827649 | Down | Armadillo repeat-containing X-linked protein 3 OS=Homo sapiens OX=9606 GN=ARMCX3 PE=1 SV=1 | ARMCX3 | _LFSAGNEETK[Acetyl (K)]LQVLK_ |
| Q9UHK0 | 277 | K | 0.09377142 | Down | FMR1-interacting protein NUFIP1 OS=Homo sapiens OX=9606 GN=NUFIP1 PE=1 SV=2 | NUFIP1 | _GAVLTTTQYGK[Acetyl (K)]MK_ |
| Q9UHQ4 | 96 | K | 2.720557112 | Up | B-cell receptor-associated protein 29 OS=Homo sapiens OX=9606 GN=BCAP29 PE=1 SV=2 | BCAP29 | _SSTSRPDAYEHTQMK[Acetyl (K)]LFR_ |
| Q9UHR4 | 94 | K | 0.521921444 | Down | Brain-specific angiogenesis inhibitor 1-associated protein 2-like protein 1 OS=Homo sapiens OX=9606 GN=BAIAP2L1 PE=1 SV=2 | BAIAP2L1 | _LNESLDENFK[Acetyl (K)]K_ |
| Q9UHR5 | 137 | K | 0.250158132 | Down | SAP30-binding protein OS=Homo sapiens OX=9606 GN=SAP30BP PE=1 SV=1 | SAP30BP | _IQK[Acetyl (K)]LYER_ |
| Q9UHR5 | 220 | K | 1.533959694 | Up | SAP30-binding protein OS=Homo sapiens OX=9606 GN=SAP30BP PE=1 SV=1 | SAP30BP | _TK[Acetyl (K)]IEFVTGTK_ |
| Q9UHR5 | 304 | K | 1.826423108 | Up | SAP30-binding protein OS=Homo sapiens OX=9606 GN=SAP30BP PE=1 SV=1 | SAP30BP | _TTVISAVGTIVK[Acetyl (K)]K_ |
| Q9UHV7 | 366 | K | 1.76806255 | Up | Mediator of RNA polymerase II transcription subunit 13 OS=Homo sapiens OX=9606 GN=MED13 PE=1 SV=3 | MED13 | _FSSVSDGFNSDSTSHHGGK[Acetyl (K)]IPR_ |
| Q9UHW9 | 1051 | K | 0.62125061 | Down | Solute carrier family 12 member 6 OS=Homo sapiens OX=9606 GN=SLC12A6 PE=1 SV=2 | SLC12A6 | _VHMTWTK[Acetyl (K)]DK_ |
| Q9UHW9 | 1089 | K | 0.661742194 | Down | Solute carrier family 12 member 6 OS=Homo sapiens OX=9606 GN=SLC12A6 PE=1 SV=2 | SLC12A6 | _MHTAVK[Acetyl (K)]LNEVIVNK_ |
| Q9UHX1 | 454 | K | 1.989995991 | Up | Poly(U)-binding-splicing factor PUF60 OS=Homo sapiens OX=9606 GN=PUF60 PE=1 SV=1 | PUF60 | _HM[Oxidation (M)]VMQK[Acetyl (K)]LLR_ |
| Q9UHX3 | 284 | K | 0.417382894 | Down | Adhesion G protein-coupled receptor E2 OS=Homo sapiens OX=9606 GN=ADGRE2 PE=1 SV=2 | ADGRE2 | _FFDK[Acetyl (K)]VQDLGR_ |
| Q9UI10 | 176 | K | 0.460930007 | Down | Translation initiation factor eIF-2B subunit delta OS=Homo sapiens OX=9606 GN=EIF2B4 PE=1 SV=2 | EIF2B4 | _DYGSK[Acetyl (K)]VSLFSHLPQYSR_ |
| Q9UI26 | 216 | K | 0.367761309 | Down | Importin-11 OS=Homo sapiens OX=9606 GN=IPO11 PE=1 SV=1 | IPO11 | _TLLSLK[Acetyl (K)]VLR_ |
| Q9UIF9 | 585 | K | 1.670972973 | Up | Bromodomain adjacent to zinc finger domain protein 2A OS=Homo sapiens OX=9606 GN=BAZ2A PE=1 SV=4 | BAZ2A | _WQGETWYYGPC[Carbamidomethyl (C)]GK[Acetyl (K)]R_ |
| Q9UIF9 | 680 | K | 1.539494316 | Up | Bromodomain adjacent to zinc finger domain protein 2A OS=Homo sapiens OX=9606 GN=BAZ2A PE=1 SV=4 | BAZ2A | _VK[Acetyl (K)]ITELLNK_ |
| Q9UIG0 | 853 | K | 1.876932918 | Up | Tyrosine-protein kinase BAZ1B OS=Homo sapiens OX=9606 GN=BAZ1B PE=1 SV=2 | BAZ1B | _LLAIQAK[Acetyl (K)]K_ |
| Q9UIG0 | 1335 | K | 1.938579077 | Up | Tyrosine-protein kinase BAZ1B OS=Homo sapiens OX=9606 GN=BAZ1B PE=1 SV=2 | BAZ1B | _APPVDDAEVDELVLQTK[Acetyl (K)]R_ |
| Q9UIJ7 | 189 | K | 2.096646249 | Up | GTP:AMP phosphotransferase AK3, mitochondrial OS=Homo sapiens OX=9606 GN=AK3 PE=1 SV=4 | AK3 | _PVLEYYQK[Acetyl (K)]K_ |
| Q9UIQ6 | 608 | K | 0.596199322 | Down | Leucyl-cystinyl aminopeptidase OS=Homo sapiens OX=9606 GN=LNPEP PE=1 SV=3 | LNPEP | _MMK[Acetyl (K)]TWTLQK_ |
| Q9UIV1 | 25 | K | 0.582068007 | Down | CCR4-NOT transcription complex subunit 7 OS=Homo sapiens OX=9606 GN=CNOT7 PE=1 SV=3 | CNOT7 | _IC[Carbamidomethyl (C)]EVWAC[Carbamidomethyl (C)]NLDEEMK[Acetyl (K)]K_ |
| Q9UJS0 | 408 | K | 1.809626817 | Up | Electrogenic aspartate/glutamate antiporter SLC25A13, mitochondrial OS=Homo sapiens OX=9606 GN=SLC25A13 PE=1 SV=2 | SLC25A13 | _AIK[Acetyl (K)]LTVNDFVR_ |
| Q9UJV9 | 49 | K | 1.967460992 | Up | Probable ATP-dependent RNA helicase DDX41 OS=Homo sapiens OX=9606 GN=DDX41 PE=1 SV=2 | DDX41 | _QLLLQK[Acetyl (K)]LLQR_ |
| Q9UJX2 | 472 | K | 0.468601816 | Down | Cell division cycle protein 23 homolog OS=Homo sapiens OX=9606 GN=CDC23 PE=1 SV=3 | CDC23 | _MALVK[Acetyl (K)]LAK_ |
| Q9UK61 | 353 | K | 2.912802882 | Up | Protein TASOR OS=Homo sapiens OX=9606 GN=TASOR PE=1 SV=3 | TASOR | _NIDK[Acetyl (K)]YNYTLWK_ |
| Q9UKD2 | 191 | K | 0.248266931 | Down | mRNA turnover protein 4 homolog OS=Homo sapiens OX=9606 GN=MRTO4 PE=1 SV=2 | MRTO4 | _VLK[Acetyl (K)]LFGYEMAEFK_ |
| Q9UKD2 | 205 | K | 0.640640792 | Down | mRNA turnover protein 4 homolog OS=Homo sapiens OX=9606 GN=MRTO4 PE=1 SV=2 | MRTO4 | _VTIK[Acetyl (K)]YMWDSQSGR_ |
| Q9UKJ3 | 1150 | K | 1.79667768 | Up | G patch domain-containing protein 8 OS=Homo sapiens OX=9606 GN=GPATCH8 PE=1 SV=2 | GPATCH8 | _PVLPLIGK[Acetyl (K)]LPATR_ |
| Q9UKL3 | 1059 | K | 0.600544185 | Down | CASP8-associated protein 2 OS=Homo sapiens OX=9606 GN=CASP8AP2 PE=1 SV=1 | CASP8AP2 | _AK[Acetyl (K)]FSLIQFHR_ |
| Q9UKM9 | 4 | K | 2.187174833 | Up | RNA-binding protein Raly OS=Homo sapiens OX=9606 GN=RALY PE=1 SV=1 | RALY | _[Acetyl (Protein N-term)]SLK[Acetyl (K)]LQASNVTNK_ |
| Q9UKM9 | 159 | K | 0.401871553 | Down | RNA-binding protein Raly OS=Homo sapiens OX=9606 GN=RALY PE=1 SV=1 | RALY | _VK[Acetyl (K)]TNVPVK[Acetyl (K)]LFAR_ |
| Q9UKM9 | 165 | K | 0.069175714 | Down | RNA-binding protein Raly OS=Homo sapiens OX=9606 GN=RALY PE=1 SV=1 | RALY | _TNVPVK[Acetyl (K)]LFAR_ |
| Q9UKN8 | 119 | K | 0.588801775 | Down | General transcription factor 3C polypeptide 4 OS=Homo sapiens OX=9606 GN=GTF3C4 PE=1 SV=2 | GTF3C4 | _TSVPAPLNSC[Carbamidomethyl (C)]LLK[Acetyl (K)]VGSK_ |
| Q9UKN8 | 165 | K | 0.608976781 | Down | General transcription factor 3C polypeptide 4 OS=Homo sapiens OX=9606 GN=GTF3C4 PE=1 SV=2 | GTF3C4 | _GFK[Acetyl (K)]YTSWSPMGC[Carbamidomethyl (C)]DANGR_ |
| Q9UKX7 | 275 | K | 0.470805024 | Down | Nuclear pore complex protein Nup50 OS=Homo sapiens OX=9606 GN=NUP50 PE=1 SV=2 | NUP50 | _TDPSSLGATSASFNFGK[Acetyl (K)]K_ |
| Q9UL03 | 835 | K | 0.642648862 | Down | Integrator complex subunit 6 OS=Homo sapiens OX=9606 GN=INTS6 PE=1 SV=1 | INTS6 | _IFTLLK[Acetyl (K)]HVQGSLQTR_ |
| Q9ULC5 | 399 | K | 1.655855115 | Up | Long-chain-fatty-acid--CoA ligase 5 OS=Homo sapiens OX=9606 GN=ACSL5 PE=1 SV=1 | ACSL5 | _HDSFWDK[Acetyl (K)]LIFAK_ |
| Q9ULG1 | 118 | K | 5.206764755 | Up | Chromatin-remodeling ATPase INO80 OS=Homo sapiens OX=9606 GN=INO80 PE=1 SV=2 | INO80 | _GNLYNFSK[Acetyl (K)]LK_ |
| Q9ULH1 | 872 | K | 2.105074122 | Up | Arf-GAP with SH3 domain, ANK repeat and PH domain-containing protein 1 OS=Homo sapiens OX=9606 GN=ASAP1 PE=1 SV=4 | ASAP1 | _TTNK[Acetyl (K)]FEGLSQQSSTSSAK_ |
| Q9ULM3 | 773 | K | 0.548527867 | Down | YEATS domain-containing protein 2 OS=Homo sapiens OX=9606 GN=YEATS2 PE=1 SV=2 | YEATS2 | _GK[Acetyl (K)]LLLIPQGAILR_ |
| Q9ULQ0 | 738 | K | 0.479143187 | Down | Striatin-interacting protein 2 OS=Homo sapiens OX=9606 GN=STRIP2 PE=1 SV=2 | STRIP2 | _TMSAIYQK[Acetyl (K)]VR_ |
| Q9UM54 | 302 | K | 1.775421561 | Up | Unconventional myosin-VI OS=Homo sapiens OX=9606 GN=MYO6 PE=1 SV=4 | MYO6 | _SPEYLK[Acetyl (K)]AGSMK_ |
| Q9UMY1 | 210 | K | 1.557680191 | Up | Nucleolar protein 7 OS=Homo sapiens OX=9606 GN=NOL7 PE=1 SV=2 | NOL7 | _TTVNK[Acetyl (K)]FLSLANK_ |
| Q9UMZ2 | 513 | K | 0.291833849 | Down | Synergin gamma OS=Homo sapiens OX=9606 GN=SYNRG PE=1 SV=2 | SYNRG | _ALPSMDK[Acetyl (K)]YAVFK_ |
| Q9UMZ2 | 744 | K | 0.226650765 | Down | Synergin gamma OS=Homo sapiens OX=9606 GN=SYNRG PE=1 SV=2 | SYNRG | _GGQNSTAASTK[Acetyl (K)]YDVFR_ |
| Q9UNF1 | 133 | K | 0.458704529 | Down | Melanoma-associated antigen D2 OS=Homo sapiens OX=9606 GN=MAGED2 PE=1 SV=2 | MAGED2 | _VSHVADTK[Acetyl (K)]VNTK_ |
| Q9UNL4 | 127 | K | 1.532083391 | Up | Inhibitor of growth protein 4 OS=Homo sapiens OX=9606 GN=ING4 PE=1 SV=1 | ING4 | _QIESSDYDSSSSK[Acetyl (K)]GK[Acetyl (K)]K[Acetyl (K)]K_ |
| Q9UNL4 | 129 | K | 1.532083391 | Up | Inhibitor of growth protein 4 OS=Homo sapiens OX=9606 GN=ING4 PE=1 SV=1 | ING4 | _QIESSDYDSSSSK[Acetyl (K)]GK[Acetyl (K)]K[Acetyl (K)]K_ |
| Q9UNL4 | 130 | K | 1.532083391 | Up | Inhibitor of growth protein 4 OS=Homo sapiens OX=9606 GN=ING4 PE=1 SV=1 | ING4 | _QIESSDYDSSSSK[Acetyl (K)]GK[Acetyl (K)]K[Acetyl (K)]K_ |
| Q9UNL4 | 146 | K | 4.330016 | Up | Inhibitor of growth protein 4 OS=Homo sapiens OX=9606 GN=ING4 PE=1 SV=1 | ING4 | _SK[Acetyl (K)]GK[Acetyl (K)]NSDEEAPK[Acetyl (K)]TAQK[Acetyl (K)]K_ |
| Q9UNL4 | 148 | K | 4.330016 | Up | Inhibitor of growth protein 4 OS=Homo sapiens OX=9606 GN=ING4 PE=1 SV=1 | ING4 | _SK[Acetyl (K)]GK[Acetyl (K)]NSDEEAPK[Acetyl (K)]TAQK[Acetyl (K)]K_ |
| Q9UNL4 | 156 | K | 4.330016 | Up | Inhibitor of growth protein 4 OS=Homo sapiens OX=9606 GN=ING4 PE=1 SV=1 | ING4 | _SK[Acetyl (K)]GK[Acetyl (K)]NSDEEAPK[Acetyl (K)]TAQK[Acetyl (K)]K_ |
| Q9UNL4 | 160 | K | 4.330016 | Up | Inhibitor of growth protein 4 OS=Homo sapiens OX=9606 GN=ING4 PE=1 SV=1 | ING4 | _SK[Acetyl (K)]GK[Acetyl (K)]NSDEEAPK[Acetyl (K)]TAQK[Acetyl (K)]K_ |
| Q9UNM6 | 31 | K | 0.581649117 | Down | 26S proteasome non-ATPase regulatory subunit 13 OS=Homo sapiens OX=9606 GN=PSMD13 PE=1 SV=2 | PSMD13 | _LEELYTK[Acetyl (K)]K_ |
| Q9UNX3 | 103 | K | 1.649216967 | Up | Ribosomal protein uL24-like OS=Homo sapiens OX=9606 GN=RPL26L1 PE=1 SV=1 | RPL26L1 | _ANGTTVHVGIHPSK[Acetyl (K)]VVITR_ |
| Q9UNY4 | 755 | K | 0.548513599 | Down | Transcription termination factor 2 OS=Homo sapiens OX=9606 GN=TTF2 PE=1 SV=2 | TTF2 | _VQTSIAVC[Carbamidomethyl (C)]K[Acetyl (K)]LQAC[Carbamidomethyl (C)]AR_ |
| Q9UPN9 | 763 | K | 0.61470344 | Down | E3 ubiquitin-protein ligase TRIM33 OS=Homo sapiens OX=9606 GN=TRIM33 PE=1 SV=3 | TRIM33 | _TAEK[Acetyl (K)]TSLSFK[Acetyl (K)]SDQVK_ |
| Q9UPN9 | 769 | K | 0.61470344 | Down | E3 ubiquitin-protein ligase TRIM33 OS=Homo sapiens OX=9606 GN=TRIM33 PE=1 SV=3 | TRIM33 | _TAEK[Acetyl (K)]TSLSFK[Acetyl (K)]SDQVK_ |
| Q9UPN9 | 950 | K | 3.325818662 | Up | E3 ubiquitin-protein ligase TRIM33 OS=Homo sapiens OX=9606 GN=TRIM33 PE=1 SV=3 | TRIM33 | _DIGKPEVEYDC[Carbamidomethyl (C)]DNLQHSK[Acetyl (K)]K_ |
| Q9UPT6 | 119 | K | 0.370882811 | Down | C-Jun-amino-terminal kinase-interacting protein 3 OS=Homo sapiens OX=9606 GN=MAPK8IP3 PE=1 SV=3 | MAPK8IP3 | _FIEFEDALEQEK[Acetyl (K)]K_ |
| Q9UPT8 | 597 | K | 0.57443277 | Down | Zinc finger CCCH domain-containing protein 4 OS=Homo sapiens OX=9606 GN=ZC3H4 PE=1 SV=3 | ZC3H4 | _PTGQLAEK[Acetyl (K)]LGVR_ |
| Q9UQ80 | 210 | K | 0.230468608 | Down | Proliferation-associated protein 2G4 OS=Homo sapiens OX=9606 GN=PA2G4 PE=1 SV=3 | PA2G4 | _TIIQNPTDQQK[Acetyl (K)]K_ |
| Q9UQ80 | 258 | K | 0.618960338 | Down | Proliferation-associated protein 2G4 OS=Homo sapiens OX=9606 GN=PA2G4 PE=1 SV=3 | PA2G4 | _QYGLK[Acetyl (K)]MK_ |
| Q9UQE7 | 4 | K | 0.631114711 | Down | Structural maintenance of chromosomes protein 3 OS=Homo sapiens OX=9606 GN=SMC3 PE=1 SV=2 | SMC3 | _MYIK[Acetyl (K)]QVIIQGFR_ |
| Q9UQE7 | 105 | K | 0.446888721 | Down | Structural maintenance of chromosomes protein 3 OS=Homo sapiens OX=9606 GN=SMC3 PE=1 SV=2 | SMC3 | _VIGAK[Acetyl (K)]K[Acetyl (K)]DQYFLDKK_ |
| Q9UQE7 | 106 | K | 0.446888721 | Down | Structural maintenance of chromosomes protein 3 OS=Homo sapiens OX=9606 GN=SMC3 PE=1 SV=2 | SMC3 | _VIGAK[Acetyl (K)]K[Acetyl (K)]DQYFLDKK_ |
| Q9UQN3 | 81 | K | 0.255877003 | Down | Charged multivesicular body protein 2b OS=Homo sapiens OX=9606 GN=CHMP2B PE=1 SV=1 | CHMP2B | _TFAVSSK[Acetyl (K)]VTSMSTQTK_ |
| Q9Y221 | 50 | K | 4.881340237 | Up | 60S ribosome subunit biogenesis protein NIP7 homolog OS=Homo sapiens OX=9606 GN=NIP7 PE=1 SV=1 | NIP7 | _VYYVSEK[Acetyl (K)]IMK_ |
| Q9Y221 | 100 | K | 0.532598904 | Down | 60S ribosome subunit biogenesis protein NIP7 homolog OS=Homo sapiens OX=9606 GN=NIP7 PE=1 SV=1 | NIP7 | _VWIK[Acetyl (K)]PGAEQSFLYGNHVLK_ |
| Q9Y230 | 9 | K | 0.571510803 | Down | RuvB-like 2 OS=Homo sapiens OX=9606 GN=RUVBL2 PE=1 SV=3 | RUVBL2 | _[Acetyl (Protein N-term)]ATVTATTK[Acetyl (K)]VPEIR_ |
| Q9Y262 | 143 | K | 1.691701238 | Up | Eukaryotic translation initiation factor 3 subunit L OS=Homo sapiens OX=9606 GN=EIF3L PE=1 SV=1 | EIF3L | _HIYAK[Acetyl (K)]VSGGPSLEQR_ |
| Q9Y266 | 247 | K | 1.759924582 | Up | Nuclear migration protein nudC OS=Homo sapiens OX=9606 GN=NUDC PE=1 SV=1 | NUDC | _VVTVHLEK[Acetyl (K)]INK_ |
| Q9Y285 | 311 | K | 0.264331424 | Down | Phenylalanine--tRNA ligase alpha subunit OS=Homo sapiens OX=9606 GN=FARSA PE=1 SV=3 | FARSA | _THSQGGYGSQGYK[Acetyl (K)]YNWK_ |
| Q9Y285 | 427 | K | 0.429336417 | Down | Phenylalanine--tRNA ligase alpha subunit OS=Homo sapiens OX=9606 GN=FARSA PE=1 SV=3 | FARSA | _FKPAYNPYTEPSMEVFSYHQGLK[Acetyl (K)]K_ |
| Q9Y291 | 35 | K | 0.598034654 | Down | Small ribosomal subunit protein mS33 OS=Homo sapiens OX=9606 GN=MRPS33 PE=1 SV=1 | MRPS33 | _VVK[Acetyl (K)]LFSELPLAK_ |
| Q9Y294 | 134 | K | 2.024418131 | Up | Histone chaperone ASF1A OS=Homo sapiens OX=9606 GN=ASF1A PE=1 SV=1 | ASF1A | _ENPPVKPDFSK[Acetyl (K)]LQR_ |
| Q9Y2A7 | 16 | K | 0.466313454 | Down | Nck-associated protein 1 OS=Homo sapiens OX=9606 GN=NCKAP1 PE=1 SV=1 | NCKAP1 | _LAEK[Acetyl (K)]LTILNDR_ |
| Q9Y2G3 | 778 | K | 1.895785356 | Up | Phospholipid-transporting ATPase IF OS=Homo sapiens OX=9606 GN=ATP11B PE=1 SV=2 | ATP11B | _EHEK[Acetyl (K)]LFMEVC[Carbamidomethyl (C)]R_ |
| Q9Y2H6 | 432 | K | 0.647428065 | Down | Fibronectin type-III domain-containing protein 3A OS=Homo sapiens OX=9606 GN=FNDC3A PE=1 SV=4 | FNDC3A | _ITK[Acetyl (K)]LSPAMGC[Carbamidomethyl (C)]K_ |
| Q9Y2H6 | 440 | K | 0.646330233 | Down | Fibronectin type-III domain-containing protein 3A OS=Homo sapiens OX=9606 GN=FNDC3A PE=1 SV=4 | FNDC3A | _LSPAMGC[Carbamidomethyl (C)]K[Acetyl (K)]FR_ |
| Q9Y2L1 | 107 | K | 0.574961115 | Down | Exosome complex exonuclease RRP44 OS=Homo sapiens OX=9606 GN=DIS3 PE=1 SV=2 | DIS3 | _SAPVYK[Acetyl (K)]R_ |
| Q9Y2R5 | 16 | K | 0.376885101 | Down | Small ribosomal subunit protein uS17m OS=Homo sapiens OX=9606 GN=MRPS17 PE=1 SV=1 | MRPS17 | _WIVGK[Acetyl (K)]VIGTK_ |
| Q9Y2R9 | 125 | K | 2.027864352 | Up | Small ribosomal subunit protein uS7m OS=Homo sapiens OX=9606 GN=MRPS7 PE=1 SV=2 | MRPS7 | _QFEK[Acetyl (K)]YHAASAEEQATIER_ |
| Q9Y2U8 | 859 | K | 1.888092092 | Up | Inner nuclear membrane protein Man1 OS=Homo sapiens OX=9606 GN=LEMD3 PE=1 SV=2 | LEMD3 | _ALHGSWFDGK[Acetyl (K)]LVTVK_ |
| Q9Y2U8 | 864 | K | 0.623980539 | Down | Inner nuclear membrane protein Man1 OS=Homo sapiens OX=9606 GN=LEMD3 PE=1 SV=2 | LEMD3 | _LVTVK[Acetyl (K)]YLR_ |
| Q9Y2W1 | 455 | K | 1.521352887 | Up | Thyroid hormone receptor-associated protein 3 OS=Homo sapiens OX=9606 GN=THRAP3 PE=1 SV=2 | THRAP3 | _FMSK[Acetyl (K)]VIGANK_ |
| Q9Y2W1 | 519 | K | 1.900850797 | Up | Thyroid hormone receptor-associated protein 3 OS=Homo sapiens OX=9606 GN=THRAP3 PE=1 SV=2 | THRAP3 | _GFVPEK[Acetyl (K)]NFR_ |
| Q9Y2W2 | 13 | K | 0.208687565 | Down | WW domain-binding protein 11 OS=Homo sapiens OX=9606 GN=WBP11 PE=1 SV=1 | WBP11 | _SGK[Acetyl (K)]FMNPTDQAR_ |
| Q9Y305 | 407 | K | 0.372013214 | Down | Acyl-coenzyme A thioesterase 9, mitochondrial OS=Homo sapiens OX=9606 GN=ACOT9 PE=1 SV=2 | ACOT9 | _EVPLVFPK[Acetyl (K)]TYGESMLYLDGQR_ |
| Q9Y316 | 171 | K | 1.619087875 | Up | Protein MEMO1 OS=Homo sapiens OX=9606 GN=MEMO1 PE=1 SV=1 | MEMO1 | _EQEFGK[Acetyl (K)]LFSK_ |
| Q9Y383 | 213 | K | 2.957826722 | Up | Putative RNA-binding protein Luc7-like 2 OS=Homo sapiens OX=9606 GN=LUC7L2 PE=1 SV=2 | LUC7L2 | _LADHFGGK[Acetyl (K)]LHLGFIEIR_ |
| Q9Y3A2 | 218 | K | 0.633904916 | Down | Probable U3 small nucleolar RNA-associated protein 11 OS=Homo sapiens OX=9606 GN=UTP11 PE=1 SV=2 | UTP11 | _LFVIAQK[Acetyl (K)]IQTR_ |
| Q9Y3A3 | 155 | K | 1.586215893 | Up | MOB-like protein phocein OS=Homo sapiens OX=9606 GN=MOB4 PE=1 SV=1 | MOB4 | _ESSVAK[Acetyl (K)]LGSVC[Carbamidomethyl (C)]R_ |
| Q9Y3A5 | 35 | K | 0.169923778 | Down | Ribosome maturation protein SBDS OS=Homo sapiens OX=9606 GN=SBDS PE=1 SV=4 | SBDS | _NK[Acetyl (K)]VVGWR_ |
| Q9Y3B4 | 100 | K | 1.624088378 | Up | Splicing factor 3B subunit 6 OS=Homo sapiens OX=9606 GN=SF3B6 PE=1 SV=1 | SF3B6 | _AFQK[Acetyl (K)]MDTK_ |
| Q9Y3E0 | 62 | K | 1.938772119 | Up | Vesicle transport protein GOT1B OS=Homo sapiens OX=9606 GN=GOLT1B PE=1 SV=1 | GOLT1B | _FFFQK[Acetyl (K)]HK_ |
| Q9Y3T9 | 226 | K | 1.679132837 | Up | Nucleolar complex protein 2 homolog OS=Homo sapiens OX=9606 GN=NOC2L PE=1 SV=4 | NOC2L | _LLFGK[Acetyl (K)]VAK_ |
| Q9Y3Y2 | 70 | K | 1.679140582 | Up | Chromatin target of PRMT1 protein OS=Homo sapiens OX=9606 GN=CHTOP PE=1 SV=2 | CHTOP | _LAQQMENRPSVQAALK[Acetyl (K)]LK_ |
| Q9Y450 | 332 | K | 0.628122404 | Down | HBS1-like protein OS=Homo sapiens OX=9606 GN=HBS1L PE=1 SV=1 | HBS1L | _GVTMDVGMTK[Acetyl (K)]FETTTK_ |
| Q9Y450 | 424 | K | 0.551177503 | Down | HBS1-like protein OS=Homo sapiens OX=9606 GN=HBS1L PE=1 SV=1 | HBS1L | _FQEITGK[Acetyl (K)]LGHFLK_ |
| Q9Y4A5 | 3495 | K | 0.63032225 | Down | Transformation/transcription domain-associated protein OS=Homo sapiens OX=9606 GN=TRRAP PE=1 SV=3 | TRRAP | _PTHYYIK[Acetyl (K)]IAR_ |
| Q9Y4B6 | 284 | K | 0.527387838 | Down | DDB1- and CUL4-associated factor 1 OS=Homo sapiens OX=9606 GN=DCAF1 PE=1 SV=3 | DCAF1 | _QK[Acetyl (K)]LGFSSSDPDR_ |
| Q9Y4C2 | 194 | K | 0.66016041 | Down | TRPM8 channel-associated factor 1 OS=Homo sapiens OX=9606 GN=TCAF1 PE=1 SV=3 | TCAF1 | _GDTSFFK[Acetyl (K)]VSK_ |
| Q9Y4C8 | 752 | K | 1.670265365 | Up | Probable RNA-binding protein 19 OS=Homo sapiens OX=9606 GN=RBM19 PE=1 SV=3 | RBM19 | _EVFSK[Acetyl (K)]VGTVK_ |
| Q9Y4E5 | 731 | K | 1.982984711 | Up | E3 SUMO-protein ligase ZNF451 OS=Homo sapiens OX=9606 GN=ZNF451 PE=1 SV=2 | ZNF451 | _ESYIC[Carbamidomethyl (C)]K[Acetyl (K)]VNR_ |
| Q9Y4F3 | 1297 | K | 0.622456523 | Down | Meiosis regulator and mRNA stability factor 1 OS=Homo sapiens OX=9606 GN=MARF1 PE=1 SV=6 | MARF1 | _QC[Carbamidomethyl (C)]K[Acetyl (K)]LAYYGFTK_ |
| Q9Y4P3 | 120 | K | 0.64291781 | Down | Transducin beta-like protein 2 OS=Homo sapiens OX=9606 GN=TBL2 PE=1 SV=1 | TBL2 | _IWSTK[Acetyl (K)]DFLQR_ |
| Q9Y4W6 | 488 | K | 0.588448176 | Down | AFG3-like protein 2 OS=Homo sapiens OX=9606 GN=AFG3L2 PE=1 SV=2 | AFG3L2 | _ASIFK[Acetyl (K)]VHLRPLK_ |
| Q9Y520 | 47 | K | 0.533353699 | Down | Protein PRRC2C OS=Homo sapiens OX=9606 GN=PRRC2C PE=1 SV=4 | PRRC2C | _HGLQSLGK[Acetyl (K)]VGISR_ |
| Q9Y5A7 | 305 | K | 0.413666786 | Down | NEDD8 ultimate buster 1 OS=Homo sapiens OX=9606 GN=NUB1 PE=1 SV=2 | NUB1 | _LEQLEC[Carbamidomethyl (C)]LDDAEK[Acetyl (K)]K_ |
| Q9Y5A9 | 428 | K | 0.596369371 | Down | YTH domain-containing family protein 2 OS=Homo sapiens OX=9606 GN=YTHDF2 PE=1 SV=2 | YTHDF2 | _SIK[Acetyl (K)]YNIWC[Carbamidomethyl (C)]STEHGNK_ |
| Q9Y5B6 | 278 | K | 0.641503869 | Down | PAX3- and PAX7-binding protein 1 OS=Homo sapiens OX=9606 GN=PAXBP1 PE=1 SV=2 | PAXBP1 | _IVFSVK[Acetyl (K)]EK_ |
| Q9Y5B9 | 7 | K | 0.237615864 | Down | FACT complex subunit SPT16 OS=Homo sapiens OX=9606 GN=SUPT16H PE=1 SV=1 | SUPT16H | _[Acetyl (Protein N-term)]AVTLDK[Acetyl (K)]DAYYR_ |
| Q9Y5B9 | 196 | K | 0.397960726 | Down | FACT complex subunit SPT16 OS=Homo sapiens OX=9606 GN=SUPT16H PE=1 SV=1 | SUPT16H | _AASITSEVFNK[Acetyl (K)]FFK_ |
| Q9Y5B9 | 904 | K | 0.627684529 | Down | FACT complex subunit SPT16 OS=Homo sapiens OX=9606 GN=SUPT16H PE=1 SV=1 | SUPT16H | _YTEGVQSLNWTK[Acetyl (K)]IM[Oxidation (M)]K_ |
| Q9Y5Q9 | 481 | K | 1.653717178 | Up | General transcription factor 3C polypeptide 3 OS=Homo sapiens OX=9606 GN=GTF3C3 PE=1 SV=1 | GTF3C3 | _AAESYGK[Acetyl (K)]VVDLAPLHLDAR_ |
| Q9Y5S9 | 114 | K | 0.369723461 | Down | RNA-binding protein 8A OS=Homo sapiens OX=9606 GN=RBM8A PE=1 SV=1 | RBM8A | _TGYLK[Acetyl (K)]GYTLVEYETYK_ |
| Q9Y5U2 | 217 | K | 1.60003633 | Up | U5 small nuclear ribonucleoprotein TSSC4 OS=Homo sapiens OX=9606 GN=TSSC4 PE=1 SV=3 | TSSC4 | _VIFTK[Acetyl (K)]PVR_ |
| Q9Y617 | 127 | K | 1.85735422 | Up | Phosphoserine aminotransferase OS=Homo sapiens OX=9606 GN=PSAT1 PE=1 SV=2 | PSAT1 | _FGTINIVHPK[Acetyl (K)]LGSYTK_ |
| Q9Y624 | 125 | K | 1.676419341 | Up | Junctional adhesion molecule A OS=Homo sapiens OX=9606 GN=F11R PE=1 SV=1 | F11R | _VK[Acetyl (K)]LIVLVPPSK_ |
| Q9Y676 | 99 | K | 1.709284195 | Up | Small ribosomal subunit protein mS40 OS=Homo sapiens OX=9606 GN=MRPS18B PE=1 SV=1 | MRPS18B | _NK[Acetyl (K)]VVGNPC[Carbamidomethyl (C)]PIC[Carbamidomethyl (C)]R_ |
| Q9Y676 | 112 | K | 1.678089183 | Up | Small ribosomal subunit protein mS40 OS=Homo sapiens OX=9606 GN=MRPS18B PE=1 SV=1 | MRPS18B | _DHK[Acetyl (K)]LHVDFR_ |
| Q9Y678 | 175 | K | 0.517922207 | Down | Coatomer subunit gamma-1 OS=Homo sapiens OX=9606 GN=COPG1 PE=1 SV=1 | COPG1 | _C[Carbamidomethyl (C)]SFDVVK[Acetyl (K)]R_ |
| Q9Y6C9 | 293 | K | 0.639972253 | Down | Mitochondrial carrier homolog 2 OS=Homo sapiens OX=9606 GN=MTCH2 PE=1 SV=1 | MTCH2 | _VPFGK[Acetyl (K)]TYC[Carbamidomethyl (C)]C[Carbamidomethyl (C)]DLK_ |
| Q9Y6G9 | 303 | K | 17.66357822 | Up | Cytoplasmic dynein 1 light intermediate chain 1 OS=Homo sapiens OX=9606 GN=DYNC1LI1 PE=1 SV=3 | DYNC1LI1 | _YIVQK[Acetyl (K)]LYGFPYK_ |
| Q9Y6G9 | 428 | K | 0.24658015 | Down | Cytoplasmic dynein 1 light intermediate chain 1 OS=Homo sapiens OX=9606 GN=DYNC1LI1 PE=1 SV=3 | DYNC1LI1 | _SVSSNVASVSPIPAGSK[Acetyl (K)]K_ |
| Q9Y6I4 | 145 | K | 1.811260907 | Up | Ubiquitin carboxyl-terminal hydrolase 3 OS=Homo sapiens OX=9606 GN=USP3 PE=1 SV=2 | USP3 | _LLENSTLNSK[Acetyl (K)]LLK_ |
| Q9Y6N5 | 115 | K | 0.503106592 | Down | Sulfide:quinone oxidoreductase, mitochondrial OS=Homo sapiens OX=9606 GN=SQOR PE=1 SV=1 | SQOR | _PTASVIPSGVEWIK[Acetyl (K)]AR_ |
| Q9Y6N5 | 173 | K | 0.584342102 | Down | Sulfide:quinone oxidoreductase, mitochondrial OS=Homo sapiens OX=9606 GN=SQOR PE=1 SV=1 | SQOR | _IGSNYSVK[Acetyl (K)]TVEK_ |
| Q9Y6X4 | 368 | K | 3.033484762 | Up | Soluble lamin-associated protein of 75 kDa OS=Homo sapiens OX=9606 GN=FAM169A PE=1 SV=2 | FAM169A | _TSQTSLTASINK[Acetyl (K)]LESTAR_ |
| Q9Y6Y8 | 477 | K | 0.556804634 | Down | SEC23-interacting protein OS=Homo sapiens OX=9606 GN=SEC23IP PE=1 SV=1 | SEC23IP | _VVSLK[Acetyl (K)]LLR_ |

Supplementary Table 3. Differentially Expressed Lactylated Proteins

| **Protein accession** | **Position** | **Amino acid** | **R116/NC116 Ratio** | **Regulated Type** | **Protein description** | **Gene name** | **Modified sequence** |
| --- | --- | --- | --- | --- | --- | --- | --- |
| A0A8I5KQE6 | 42 | K | 0.580846167 | Down | Small ribosomal subunit protein uS2B OS=Homo sapiens OX=9606 GN=RPSA2 PE=3 SV=1 | RPSA2 | _K[Lac (K)]SDGIYIINLK_ |
| A0FGR8 | 405 | K | 0.55961696 | Down | Extended synaptotagmin-2 OS=Homo sapiens OX=9606 GN=ESYT2 PE=1 SV=1 | ESYT2 | _DTYLK[Lac (K)]GLVK_ |
| A0MZ66 | 546 | K | 1.663750974 | Up | Shootin-1 OS=Homo sapiens OX=9606 GN=SHTN1 PE=1 SV=4 | SHTN1 | _K[Lac (K)]LEGC[Carbamidomethyl (C)]TSSK_ |
| A1X283 | 531 | K | 0.454268104 | Down | SH3 and PX domain-containing protein 2B OS=Homo sapiens OX=9606 GN=SH3PXD2B PE=1 SV=3 | SH3PXD2B | _ESIIK[Lac (K)]SEGELLER_ |
| A1X283 | 669 | K | 0.649834878 | Down | SH3 and PX domain-containing protein 2B OS=Homo sapiens OX=9606 GN=SH3PXD2B PE=1 SV=3 | SH3PXD2B | _SK[Lac (K)]LRPAK_ |
| A1X283 | 821 | K | 0.507086542 | Down | SH3 and PX domain-containing protein 2B OS=Homo sapiens OX=9606 GN=SH3PXD2B PE=1 SV=3 | SH3PXD2B | _GK[Lac (K)]GSLGPWGTGK_ |
| A1X283 | 831 | K | 0.456089508 | Down | SH3 and PX domain-containing protein 2B OS=Homo sapiens OX=9606 GN=SH3PXD2B PE=1 SV=3 | SH3PXD2B | _GSLGPWGTGK[Lac (K)]IGENR_ |
| A1X283 | 838 | K | 0.647864865 | Down | SH3 and PX domain-containing protein 2B OS=Homo sapiens OX=9606 GN=SH3PXD2B PE=1 SV=3 | SH3PXD2B | _EK[Lac (K)]AAAASVPNADGLK_ |
| A1XBS5 | 242 | K | 0.215938396 | Down | CBY1-interacting BAR domain-containing protein 1 OS=Homo sapiens OX=9606 GN=CIBAR1 PE=1 SV=2 | CIBAR1 | _ANSK[Lac (K)]SPLQR_ |
| A1XBS5 | 252 | K | 0.203162957 | Down | CBY1-interacting BAR domain-containing protein 1 OS=Homo sapiens OX=9606 GN=CIBAR1 PE=1 SV=2 | CIBAR1 | _SLSAK[Lac (K)]C[Carbamidomethyl (C)]VSGTGQVSTC[Carbamidomethyl (C)]R_ |
| A2AJT9 | 315 | K | 0.241210266 | Down | BCLAF1 and THRAP3 family member 3 OS=Homo sapiens OX=9606 GN=BCLAF3 PE=1 SV=1 | BCLAF3 | _YSFQK[Lac (K)]GPLNR_ |
| A2AJT9 | 347 | K | 0.280776158 | Down | BCLAF1 and THRAP3 family member 3 OS=Homo sapiens OX=9606 GN=BCLAF3 PE=1 SV=1 | BCLAF3 | _K[Lac (K)]DSIAC[Carbamidomethyl (C)]TYSNK_ |
| A3KN83 | 149 | K | 0.29876113 | Down | Protein strawberry notch homolog 1 OS=Homo sapiens OX=9606 GN=SBNO1 PE=1 SV=1 | SBNO1 | _NAMTSAPSK[Lac (K)]DQVQLK_ |
| A3KN83 | 218 | K | 0.32211741 | Down | Protein strawberry notch homolog 1 OS=Homo sapiens OX=9606 GN=SBNO1 PE=1 SV=1 | SBNO1 | _SFSPTMK[Lac (K)]VPVVK_ |
| A3KN83 | 708 | K | 0.547718717 | Down | Protein strawberry notch homolog 1 OS=Homo sapiens OX=9606 GN=SBNO1 PE=1 SV=1 | SBNO1 | _K[Lac (K)]GEEITR_ |
| A5YKK6 | 808 | K | 0.556014801 | Down | CCR4-NOT transcription complex subunit 1 OS=Homo sapiens OX=9606 GN=CNOT1 PE=1 SV=2 | CNOT1 | _K[Lac (K)]LGTSGLNQPTFQQSK_ |
| A5YKK6 | 823 | K | 0.586726679 | Down | CCR4-NOT transcription complex subunit 1 OS=Homo sapiens OX=9606 GN=CNOT1 PE=1 SV=2 | CNOT1 | _LGTSGLNQPTFQQSK[Lac (K)]MK_ |
| A6ND36 | 348 | K | 0.315342027 | Down | Protein FAM83G OS=Homo sapiens OX=9606 GN=FAM83G PE=1 SV=2 | FAM83G | _LVNPK[Lac (K)]YALVK_ |
| A6NHR9 | 1349 | K | 0.528630058 | Down | Structural maintenance of chromosomes flexible hinge domain-containing protein 1 OS=Homo sapiens OX=9606 GN=SMCHD1 PE=1 SV=2 | SMCHD1 | _AIYNK[Lac (K)]SIIEGPIIK_ |
| A6NHR9 | 1987 | K | 0.603338497 | Down | Structural maintenance of chromosomes flexible hinge domain-containing protein 1 OS=Homo sapiens OX=9606 GN=SMCHD1 PE=1 SV=2 | SMCHD1 | _VETTDC[Carbamidomethyl (C)]PVPPK[Lac (K)]R_ |
| A8CG34 | 484 | K | 0.500600035 | Down | Nuclear envelope pore membrane protein POM 121C OS=Homo sapiens OX=9606 GN=POM121C PE=1 SV=3 | POM121C | _K[Lac (K)]VQLLPSR_ |
| A8MW92 | 210 | K | 0.345153335 | Down | PHD finger protein 20-like protein 1 OS=Homo sapiens OX=9606 GN=PHF20L1 PE=1 SV=2 | PHF20L1 | _WFK[Lac (K)]VPSK_ |
| A8MW92 | 280 | K | 0.263677085 | Down | PHD finger protein 20-like protein 1 OS=Homo sapiens OX=9606 GN=PHF20L1 PE=1 SV=2 | PHF20L1 | _LNK[Lac (K)]ITGLLASK_ |
| A8MW92 | 288 | K | 0.479176153 | Down | PHD finger protein 20-like protein 1 OS=Homo sapiens OX=9606 GN=PHF20L1 PE=1 SV=2 | PHF20L1 | _ITGLLASK[Lac (K)]AVGVDGAEK_ |
| A8MW92 | 346 | K | 0.288050351 | Down | PHD finger protein 20-like protein 1 OS=Homo sapiens OX=9606 GN=PHF20L1 PE=1 SV=2 | PHF20L1 | _PALLSSTLSSGK[Lac (K)]AR_ |
| A8MW92 | 530 | K | 0.392510891 | Down | PHD finger protein 20-like protein 1 OS=Homo sapiens OX=9606 GN=PHF20L1 PE=1 SV=2 | PHF20L1 | _GAPAAAGISK[Lac (K)]TEK_ |
| E9PAV3 | 2005 | K | 1.627111415 | Up | Nascent polypeptide-associated complex subunit alpha, muscle-specific form OS=Homo sapiens OX=9606 GN=NACA PE=1 SV=1 | NACA | _IEDLSQQAQLAAAEK[Lac (K)]FK_ |
| E9PRG8 | 67 | K | 0.470761549 | Down | Uncharacterized protein C11orf98 OS=Homo sapiens OX=9606 GN=C11orf98 PE=4 SV=2 | C11orf98 | _ANITLSGK[Lac (K)]K_ |
| E9PRG8 | 103 | K | 0.576211102 | Down | Uncharacterized protein C11orf98 OS=Homo sapiens OX=9606 GN=C11orf98 PE=4 SV=2 | C11orf98 | _TSEPQLK[Lac (K)]R_ |
| E9PRG8 | 109 | K | 0.505658966 | Down | Uncharacterized protein C11orf98 OS=Homo sapiens OX=9606 GN=C11orf98 PE=4 SV=2 | C11orf98 | _TK[Lac (K)]APQDVEMK_ |
| O00139 | 102 | K | 0.618866441 | Down | Kinesin-like protein KIF2A OS=Homo sapiens OX=9606 GN=KIF2A PE=1 SV=3 | KIF2A | _TVASIK[Lac (K)]NDPPSR_ |
| O00151 | 98 | K | 0.621290206 | Down | PDZ and LIM domain protein 1 OS=Homo sapiens OX=9606 GN=PDLIM1 PE=1 SV=4 | PDLIM1 | _VWSPLVTEEGK[Lac (K)]R_ |
| O00151 | 241 | K | 0.548328785 | Down | PDZ and LIM domain protein 1 OS=Homo sapiens OX=9606 GN=PDLIM1 PE=1 SV=4 | PDLIM1 | _SVK[Lac (K)]APVTK_ |
| O00151 | 256 | K | 0.555180959 | Down | PDZ and LIM domain protein 1 OS=Homo sapiens OX=9606 GN=PDLIM1 PE=1 SV=4 | PDLIM1 | _VAASIGNAQK[Lac (K)]LPMC[Carbamidomethyl (C)]DK_ |
| O00267 | 143 | K | 0.291675632 | Down | Transcription elongation factor SPT5 OS=Homo sapiens OX=9606 GN=SUPT5H PE=1 SV=1 | SUPT5H | _EEELGEYYMK[Lac (K)]K_ |
| O00418 | 58 | K | 0.379818367 | Down | Eukaryotic elongation factor 2 kinase OS=Homo sapiens OX=9606 GN=EEF2K PE=1 SV=2 | EEF2K | _VNK[Lac (K)]YYSNLTK_ |
| O00429 | 597 | K | 0.474202028 | Down | Dynamin-1-like protein OS=Homo sapiens OX=9606 GN=DNM1L PE=1 SV=2 | DNM1L | _TSK[Lac (K)]AEELLAEEK_ |
| O00443 | 613 | K | 0.440914558 | Down | Phosphatidylinositol 4-phosphate 3-kinase C2 domain-containing subunit alpha OS=Homo sapiens OX=9606 GN=PIK3C2A PE=1 SV=2 | PIK3C2A | _SK[Lac (K)]TADVTSLFGGEDTSR_ |
| O00499 | 20 | K | 0.542301319 | Down | Myc box-dependent-interacting protein 1 OS=Homo sapiens OX=9606 GN=BIN1 PE=1 SV=1 | BIN1 | _IASNVQK[Lac (K)]K_ |
| O00505 | 47 | K | 0.627791894 | Down | Importin subunit alpha-4 OS=Homo sapiens OX=9606 GN=KPNA3 PE=1 SV=2 | KPNA3 | _DEHLLK[Lac (K)]K_ |
| O00515 | 241 | K | 0.617071498 | Down | Ladinin-1 OS=Homo sapiens OX=9606 GN=LAD1 PE=1 SV=2 | LAD1 | _TSVSEK[Lac (K)]SLAPGMALGSGR_ |
| O00515 | 382 | K | 2.542987814 | Up | Ladinin-1 OS=Homo sapiens OX=9606 GN=LAD1 PE=1 SV=2 | LAD1 | _K[Lac (K)]ENSETTLTR_ |
| O00541 | 98 | K | 0.419480581 | Down | Pescadillo homolog OS=Homo sapiens OX=9606 GN=PES1 PE=1 SV=1 | PES1 | _AYGK[Lac (K)]SEWNTVER_ |
| O00541 | 441 | K | 0.493354131 | Down | Pescadillo homolog OS=Homo sapiens OX=9606 GN=PES1 PE=1 SV=1 | PES1 | _LK[Lac (K)]LLALQR_ |
| O00541 | 517 | K | 0.356868709 | Down | Pescadillo homolog OS=Homo sapiens OX=9606 GN=PES1 PE=1 SV=1 | PES1 | _VMAGTLK[Lac (K)]LEDK_ |
| O00566 | 157 | K | 0.633074532 | Down | U3 small nucleolar ribonucleoprotein protein MPP10 OS=Homo sapiens OX=9606 GN=MPHOSPH10 PE=1 SV=2 | MPHOSPH10 | _AENSSK[Lac (K)]SDLR_ |
| O00566 | 370 | K | 0.634884193 | Down | U3 small nucleolar ribonucleoprotein protein MPP10 OS=Homo sapiens OX=9606 GN=MPHOSPH10 PE=1 SV=2 | MPHOSPH10 | _MNEK[Lac (K)]IASLEK_ |
| O00566 | 612 | K | 0.539074458 | Down | U3 small nucleolar ribonucleoprotein protein MPP10 OS=Homo sapiens OX=9606 GN=MPHOSPH10 PE=1 SV=2 | MPHOSPH10 | _YSK[Lac (K)]TVASEK_ |
| O00566 | 641 | K | 0.153345886 | Down | U3 small nucleolar ribonucleoprotein protein MPP10 OS=Homo sapiens OX=9606 GN=MPHOSPH10 PE=1 SV=2 | MPHOSPH10 | _ALK[Lac (K)]SSQAFFSK_ |
| O00566 | 649 | K | 0.337951745 | Down | U3 small nucleolar ribonucleoprotein protein MPP10 OS=Homo sapiens OX=9606 GN=MPHOSPH10 PE=1 SV=2 | MPHOSPH10 | _SSQAFFSK[Lac (K)]LQDQVK_ |
| O00567 | 484 | K | 0.483406104 | Down | Nucleolar protein 56 OS=Homo sapiens OX=9606 GN=NOP56 PE=1 SV=4 | NOP56 | _K[Lac (K)]QKPQEVPQENGMEDPSISFSKPK_ |
| O00571 | 55 | K | 0.638260768 | Down | ATP-dependent RNA helicase DDX3X OS=Homo sapiens OX=9606 GN=DDX3X PE=1 SV=3 | DDX3X | _GFYDK[Lac (K)]DSSGWSSSK_ |
| O00571 | 81 | K | 0.651119804 | Down | ATP-dependent RNA helicase DDX3X OS=Homo sapiens OX=9606 GN=DDX3X PE=1 SV=3 | DDX3X | _GK[Lac (K)]SSFFSDR_ |
| O00629 | 47 | K | 0.602677972 | Down | Importin subunit alpha-3 OS=Homo sapiens OX=9606 GN=KPNA4 PE=1 SV=1 | KPNA4 | _DEHLLK[Lac (K)]R_ |
| O00716 | 151 | K | 0.568590887 | Down | Transcription factor E2F3 OS=Homo sapiens OX=9606 GN=E2F3 PE=1 SV=1 | E2F3 | _LELGESGHQYLSDGLK[Lac (K)]TPK_ |
| O00767 | 196 | K | 2.459935098 | Up | Stearoyl-CoA desaturase OS=Homo sapiens OX=9606 GN=SCD PE=1 SV=2 | SCD | _EK[Lac (K)]GSTLDLSDLEAEK_ |
| O14497 | 1382 | K | 0.443625217 | Down | AT-rich interactive domain-containing protein 1A OS=Homo sapiens OX=9606 GN=ARID1A PE=1 SV=3 | ARID1A | _RPMDGTYGPPAK[Lac (K)]R_ |
| O14562 | 130 | K | 0.43630987 | Down | Ubiquitin domain-containing protein UBFD1 OS=Homo sapiens OX=9606 GN=UBFD1 PE=1 SV=2 | UBFD1 | _VMYK[Lac (K)]GLVPEDK_ |
| O14639 | 549 | K | 0.42187759 | Down | Actin-binding LIM protein 1 OS=Homo sapiens OX=9606 GN=ABLIM1 PE=1 SV=3 | ABLIM1 | _FSK[Lac (K)]FPAAQAPDPSETPK_ |
| O14646 | 80 | K | 0.280074311 | Down | Chromodomain-helicase-DNA-binding protein 1 OS=Homo sapiens OX=9606 GN=CHD1 PE=1 SV=2 | CHD1 | _VQAKPPK[Lac (K)]VDGAEFWK_ |
| O14646 | 1078 | K | 0.235128904 | Down | Chromodomain-helicase-DNA-binding protein 1 OS=Homo sapiens OX=9606 GN=CHD1 PE=1 SV=2 | CHD1 | _NC[Carbamidomethyl (C)]AK[Lac (K)]QISFNGSEGR_ |
| O14647 | 1125 | K | 0.584650506 | Down | Chromodomain-helicase-DNA-binding protein 2 OS=Homo sapiens OX=9606 GN=CHD2 PE=1 SV=2 | CHD2 | _K[Lac (K)]DLVEGFTDAEIR_ |
| O14647 | 1629 | K | 0.646363174 | Down | Chromodomain-helicase-DNA-binding protein 2 OS=Homo sapiens OX=9606 GN=CHD2 PE=1 SV=2 | CHD2 | _DNYNHPNK[Lac (K)]R_ |
| O14647 | 1769 | K | 0.430321038 | Down | Chromodomain-helicase-DNA-binding protein 2 OS=Homo sapiens OX=9606 GN=CHD2 PE=1 SV=2 | CHD2 | _SFHTDK[Lac (K)]LGEYK_ |
| O14737 | 105 | K | 0.278783913 | Down | Programmed cell death protein 5 OS=Homo sapiens OX=9606 GN=PDCD5 PE=1 SV=3 | PDCD5 | _VSQQTEK[Lac (K)]TTTVK_ |
| O14737 | 110 | K | 0.489785175 | Down | Programmed cell death protein 5 OS=Homo sapiens OX=9606 GN=PDCD5 PE=1 SV=3 | PDCD5 | _TTTVK[Lac (K)]FNR_ |
| O14745 | 350 | K | 0.384899145 | Down | Na(+)/H(+) exchange regulatory cofactor NHE-RF1 OS=Homo sapiens OX=9606 GN=NHERF1 PE=1 SV=4 | NHERF1 | _APQMDWSK[Lac (K)]K_ |
| O14777 | 42 | K | 0.326009916 | Down | Kinetochore protein NDC80 homolog OS=Homo sapiens OX=9606 GN=NDC80 PE=1 SV=1 | NDC80 | _EKPTFGK[Lac (K)]LSINKPTSER_ |
| O14867 | 185 | K | 0.134166752 | Down | Transcription regulator protein BACH1 OS=Homo sapiens OX=9606 GN=BACH1 PE=1 SV=2 | BACH1 | _NVQTPQC[Carbamidomethyl (C)]K[Lac (K)]LR_ |
| O14867 | 232 | K | 0.158428792 | Down | Transcription regulator protein BACH1 OS=Homo sapiens OX=9606 GN=BACH1 PE=1 SV=2 | BACH1 | _FQK[Lac (K)]AFGTDR_ |
| O14908 | 10 | K | 0.36611835 | Down | PDZ domain-containing protein GIPC1 OS=Homo sapiens OX=9606 GN=GIPC1 PE=1 SV=2 | GIPC1 | _K[Lac (K)]APPLVENEEAEPGR_ |
| O14929 | 15 | K | 0.618545908 | Down | Histone acetyltransferase type B catalytic subunit OS=Homo sapiens OX=9606 GN=HAT1 PE=1 SV=2 | HAT1 | _FLVEYK[Lac (K)]SAVEK_ |
| O14964 | 202 | K | 0.635402904 | Down | Hepatocyte growth factor-regulated tyrosine kinase substrate OS=Homo sapiens OX=9606 GN=HGS PE=1 SV=1 | HGS | _YSTIPK[Lac (K)]FGIEK_ |
| O14974 | 320 | K | 0.64906038 | Down | Protein phosphatase 1 regulatory subunit 12A OS=Homo sapiens OX=9606 GN=PPP1R12A PE=1 SV=1 | PPP1R12A | _NK[Lac (K)]ETLIIEPEK_ |
| O14974 | 420 | K | 0.451951232 | Down | Protein phosphatase 1 regulatory subunit 12A OS=Homo sapiens OX=9606 GN=PPP1R12A PE=1 SV=1 | PPP1R12A | _FPTTATK[Lac (K)]ISPK_ |
| O14974 | 442 | K | 0.210364034 | Down | Protein phosphatase 1 regulatory subunit 12A OS=Homo sapiens OX=9606 GN=PPP1R12A PE=1 SV=1 | PPP1R12A | _K[Lac (K)]TGSYGALAEITASK_ |
| O14976 | 390 | K | 0.368888724 | Down | Cyclin-G-associated kinase OS=Homo sapiens OX=9606 GN=GAK PE=1 SV=2 | GAK | _LFTNLK[Lac (K)]DTSSK_ |
| O14976 | 395 | K | 0.280812186 | Down | Cyclin-G-associated kinase OS=Homo sapiens OX=9606 GN=GAK PE=1 SV=2 | GAK | _DTSSK[Lac (K)]VIQSVANYAK_ |
| O14976 | 658 | K | 0.565698113 | Down | Cyclin-G-associated kinase OS=Homo sapiens OX=9606 GN=GAK PE=1 SV=2 | GAK | _LQAK[Lac (K)]MASMK_ |
| O14979 | 142 | K | 1.655397322 | Up | Heterogeneous nuclear ribonucleoprotein D-like OS=Homo sapiens OX=9606 GN=HNRNPDL PE=1 SV=3 | HNRNPDL | _INASK[Lac (K)]NQQDDGK_ |
| O14981 | 217 | K | 0.388415254 | Down | TATA-binding protein-associated factor 172 OS=Homo sapiens OX=9606 GN=BTAF1 PE=1 SV=2 | BTAF1 | _MAK[Lac (K)]LFAK_ |
| O14981 | 248 | K | 0.512452883 | Down | TATA-binding protein-associated factor 172 OS=Homo sapiens OX=9606 GN=BTAF1 PE=1 SV=2 | BTAF1 | _K[Lac (K)]IANVVINQSANDSK_ |
| O15014 | 84 | K | 0.38071919 | Down | Zinc finger protein 609 OS=Homo sapiens OX=9606 GN=ZNF609 PE=1 SV=2 | ZNF609 | _FVTPVPGPQGK[Lac (K)]EGK_ |
| O15014 | 441 | K | 0.142968497 | Down | Zinc finger protein 609 OS=Homo sapiens OX=9606 GN=ZNF609 PE=1 SV=2 | ZNF609 | _K[Lac (K)]NKPLSDMELNSSSEDSK_ |
| O15014 | 586 | K | 0.398605653 | Down | Zinc finger protein 609 OS=Homo sapiens OX=9606 GN=ZNF609 PE=1 SV=2 | ZNF609 | _FSTK[Lac (K)]GLC[Carbamidomethyl (C)]K_ |
| O15014 | 639 | K | 0.495031995 | Down | Zinc finger protein 609 OS=Homo sapiens OX=9606 GN=ZNF609 PE=1 SV=2 | ZNF609 | _K[Lac (K)]PSSLKPEK_ |
| O15014 | 1082 | K | 0.217230083 | Down | Zinc finger protein 609 OS=Homo sapiens OX=9606 GN=ZNF609 PE=1 SV=2 | ZNF609 | _SVIIPK[Lac (K)]LDDSSK_ |
| O15014 | 1192 | K | 0.381155408 | Down | Zinc finger protein 609 OS=Homo sapiens OX=9606 GN=ZNF609 PE=1 SV=2 | ZNF609 | _ESTSSDC[Carbamidomethyl (C)]K[Lac (K)]LPTSEESR_ |
| O15042 | 80 | K | 0.380148184 | Down | U2 snRNP-associated SURP motif-containing protein OS=Homo sapiens OX=9606 GN=U2SURP PE=1 SV=2 | U2SURP | _PLLENK[Lac (K)]LK_ |
| O15042 | 88 | K | 0.228255587 | Down | U2 snRNP-associated SURP motif-containing protein OS=Homo sapiens OX=9606 GN=U2SURP PE=1 SV=2 | U2SURP | _AFSIGK[Lac (K)]MSTAK_ |
| O15042 | 188 | K | 0.542277972 | Down | U2 snRNP-associated SURP motif-containing protein OS=Homo sapiens OX=9606 GN=U2SURP PE=1 SV=2 | U2SURP | _NPPNQSSNERPPSLLVIETK[Lac (K)]KPPLK_ |
| O15042 | 212 | K | 0.581589112 | Down | U2 snRNP-associated SURP motif-containing protein OS=Homo sapiens OX=9606 GN=U2SURP PE=1 SV=2 | U2SURP | _EELK[Lac (K)]QIQEER_ |
| O15042 | 392 | K | 0.664982455 | Down | U2 snRNP-associated SURP motif-containing protein OS=Homo sapiens OX=9606 GN=U2SURP PE=1 SV=2 | U2SURP | _LK[Lac (K)]NPNAPMLPPPK_ |
| O15042 | 755 | K | 0.590762972 | Down | U2 snRNP-associated SURP motif-containing protein OS=Homo sapiens OX=9606 GN=U2SURP PE=1 SV=2 | U2SURP | _NEPIFK[Lac (K)]VAPSK_ |
| O15042 | 760 | K | 2.047889607 | Up | U2 snRNP-associated SURP motif-containing protein OS=Homo sapiens OX=9606 GN=U2SURP PE=1 SV=2 | U2SURP | _VAPSK[Lac (K)]WEAVDESELEAQAVTTSK_ |
| O15042 | 915 | K | 1.5029138 | Up | U2 snRNP-associated SURP motif-containing protein OS=Homo sapiens OX=9606 GN=U2SURP PE=1 SV=2 | U2SURP | _EK[Lac (K)]DEC[Carbamidomethyl (C)]TPTR_ |
| O15131 | 20 | K | 0.276015925 | Down | Importin subunit alpha-6 OS=Homo sapiens OX=9606 GN=KPNA5 PE=1 SV=3 | KPNA5 | _NK[Lac (K)]ALNPQEMR_ |
| O15144 | 275 | K | 0.336400224 | Down | Actin-related protein 2/3 complex subunit 2 OS=Homo sapiens OX=9606 GN=ARPC2 PE=1 SV=1 | ARPC2 | _TSDFLK[Lac (K)]VLNR_ |
| O15144 | 295 | K | 0.307455619 | Down | Actin-related protein 2/3 complex subunit 2 OS=Homo sapiens OX=9606 GN=ARPC2 PE=1 SV=1 | ARPC2 | _TITGK[Lac (K)]TFSSR_ |
| O15156 | 206 | K | 0.615066658 | Down | Zinc finger and BTB domain-containing protein 7B OS=Homo sapiens OX=9606 GN=ZBTB7B PE=1 SV=2 | ZBTB7B | _K[Lac (K)]AFLQTK_ |
| O15156 | 360 | K | 0.660180168 | Down | Zinc finger and BTB domain-containing protein 7B OS=Homo sapiens OX=9606 GN=ZBTB7B PE=1 SV=2 | ZBTB7B | _IIHGAGK[Lac (K)]LPR_ |
| O15164 | 711 | K | 0.162150734 | Down | Transcription intermediary factor 1-alpha OS=Homo sapiens OX=9606 GN=TRIM24 PE=1 SV=3 | TRIM24 | _PAGADSTHK[Lac (K)]VPVVMLEPIR_ |
| O15212 | 32 | K | 0.334924467 | Down | Prefoldin subunit 6 OS=Homo sapiens OX=9606 GN=PFDN6 PE=1 SV=1 | PFDN6 | _QK[Lac (K)]LEAQLTENNIVK_ |
| O15213 | 65 | K | 0.421159693 | Down | WD repeat-containing protein 46 OS=Homo sapiens OX=9606 GN=WDR46 PE=1 SV=3 | WDR46 | _NAYILK[Lac (K)]K_ |
| O15226 | 68 | K | 1.980261253 | Up | NF-kappa-B-repressing factor OS=Homo sapiens OX=9606 GN=NKRF PE=1 SV=2 | NKRF | _FEPVHFVASSSK[Lac (K)]DER_ |
| O15226 | 234 | K | 0.251881514 | Down | NF-kappa-B-repressing factor OS=Homo sapiens OX=9606 GN=NKRF PE=1 SV=2 | NKRF | _K[Lac (K)]LLTDGYAC[Carbamidomethyl (C)]EVR_ |
| O15226 | 513 | K | 0.627289279 | Down | NF-kappa-B-repressing factor OS=Homo sapiens OX=9606 GN=NKRF PE=1 SV=2 | NKRF | _K[Lac (K)]TQPTVINNLK_ |
| O15226 | 546 | K | 0.47549608 | Down | NF-kappa-B-repressing factor OS=Homo sapiens OX=9606 GN=NKRF PE=1 SV=2 | NKRF | _SAEEAYK[Lac (K)]QQIK_ |
| O15226 | 561 | K | 0.368607173 | Down | NF-kappa-B-repressing factor OS=Homo sapiens OX=9606 GN=NKRF PE=1 SV=2 | NKRF | _K[Lac (K)]MGWTGGGLGK_ |
| O15294 | 16 | K | 0.451588214 | Down | UDP-N-acetylglucosamine--peptide N-acetylglucosaminyltransferase 110 kDa subunit OS=Homo sapiens OX=9606 GN=OGT PE=1 SV=3 | OGT | _[Acetyl (Protein N-term)]ASSVGNVADSTEPTK[Lac (K)]R_ |
| O15318 | 131 | K | 0.375417135 | Down | DNA-directed RNA polymerase III subunit RPC7 OS=Homo sapiens OX=9606 GN=POLR3G PE=1 SV=2 | POLR3G | _DAGK[Lac (K)]GTPLTNTEDVLK_ |
| O15355 | 166 | K | 0.46331031 | Down | Protein phosphatase 1G OS=Homo sapiens OX=9606 GN=PPM1G PE=1 SV=1 | PPM1G | _YGQNC[Carbamidomethyl (C)]HK[Lac (K)]GPPHSK_ |
| O15355 | 519 | K | 0.637781522 | Down | Protein phosphatase 1G OS=Homo sapiens OX=9606 GN=PPM1G PE=1 SV=1 | PPM1G | _NTAELQPESGK[Lac (K)]R_ |
| O15371 | 41 | K | 0.661019231 | Down | Eukaryotic translation initiation factor 3 subunit D OS=Homo sapiens OX=9606 GN=EIF3D PE=1 SV=1 | EIF3D | _LGK[Lac (K)]VADWTGATYQDK_ |
| O15371 | 142 | K | 0.585554782 | Down | Eukaryotic translation initiation factor 3 subunit D OS=Homo sapiens OX=9606 GN=EIF3D PE=1 SV=1 | EIF3D | _FQK[Lac (K)]QFGVR_ |
| O15371 | 153 | K | 2.210764339 | Up | Eukaryotic translation initiation factor 3 subunit D OS=Homo sapiens OX=9606 GN=EIF3D PE=1 SV=1 | EIF3D | _WDQK[Lac (K)]SQKPR_ |
| O15372 | 5 | K | 1.533424585 | Up | Eukaryotic translation initiation factor 3 subunit H OS=Homo sapiens OX=9606 GN=EIF3H PE=1 SV=1 | EIF3H | _K[Lac (K)]EGTGSTATSSSSTAGAAGK_ |
| O15381 | 129 | K | 0.332016731 | Down | Nuclear valosin-containing protein-like OS=Homo sapiens OX=9606 GN=NVL PE=1 SV=1 | NVL | _K[Lac (K)]GNPDSVSNTPEMEQR_ |
| O15381 | 156 | K | 0.552392497 | Down | Nuclear valosin-containing protein-like OS=Homo sapiens OX=9606 GN=NVL PE=1 SV=1 | NVL | _ISSK[Lac (K)]TGSIPLK_ |
| O15381 | 167 | K | 0.177704367 | Down | Nuclear valosin-containing protein-like OS=Homo sapiens OX=9606 GN=NVL PE=1 SV=1 | NVL | _TPAK[Lac (K)]DSEGGWFIDK_ |
| O15427 | 448 | K | 0.663351231 | Down | Monocarboxylate transporter 4 OS=Homo sapiens OX=9606 GN=SLC16A3 PE=1 SV=1 | SLC16A3 | _EVEHFLK[Lac (K)]AEPEK_ |
| O15446 | 174 | K | 0.557577213 | Down | DNA-directed RNA polymerase I subunit RPA34 OS=Homo sapiens OX=9606 GN=POLR1G PE=1 SV=1 | POLR1G | _SALAPNLLTSGK[Lac (K)]K_ |
| O15446 | 259 | K | 0.595031756 | Down | DNA-directed RNA polymerase I subunit RPA34 OS=Homo sapiens OX=9606 GN=POLR1G PE=1 SV=1 | POLR1G | _GK[Lac (K)]ETFEPEDK_ |
| O15446 | 294 | K | 0.661797396 | Down | DNA-directed RNA polymerase I subunit RPA34 OS=Homo sapiens OX=9606 GN=POLR1G PE=1 SV=1 | POLR1G | _QK[Lac (K)]GTEGMEPEEGVTVESQPQVK_ |
| O15446 | 328 | K | 0.402432359 | Down | DNA-directed RNA polymerase I subunit RPA34 OS=Homo sapiens OX=9606 GN=POLR1G PE=1 SV=1 | POLR1G | _VEPLEEAIPLPPTK[Lac (K)]K_ |
| O15479 | 80 | K | 0.165865398 | Down | Melanoma-associated antigen B2 OS=Homo sapiens OX=9606 GN=MAGEB2 PE=1 SV=3 | MAGEB2 | _APTTAAAAAAGVSSTK[Lac (K)]SK_ |
| O15498 | 141 | K | 0.444554508 | Down | Synaptobrevin homolog YKT6 OS=Homo sapiens OX=9606 GN=YKT6 PE=1 SV=1 | YKT6 | _EADPMTK[Lac (K)]VQAELDETK_ |
| O15541 | 139 | K | 0.309284733 | Down | E3 ubiquitin-protein ligase RNF113A OS=Homo sapiens OX=9606 GN=RNF113A PE=1 SV=1 | RNF113A | _SQK[Lac (K)]IQEELR_ |
| O43143 | 407 | K | 0.320866957 | Down | ATP-dependent RNA helicase DHX15 OS=Homo sapiens OX=9606 GN=DHX15 PE=1 SV=2 | DHX15 | _IFEPPPPK[Lac (K)]K_ |
| O43148 | 60 | K | 0.195396573 | Down | mRNA cap guanine-N7 methyltransferase OS=Homo sapiens OX=9606 GN=RNMT PE=1 SV=1 | RNMT | _K[Lac (K)]EFEDDLVK_ |
| O43148 | 83 | K | 0.411268406 | Down | mRNA cap guanine-N7 methyltransferase OS=Homo sapiens OX=9606 GN=RNMT PE=1 SV=1 | RNMT | _K[Lac (K)]LDPEIVPEEK_ |
| O43148 | 128 | K | 0.299224226 | Down | mRNA cap guanine-N7 methyltransferase OS=Homo sapiens OX=9606 GN=RNMT PE=1 SV=1 | RNMT | _K[Lac (K)]IALEDVPEK_ |
| O43172 | 15 | K | 0.205436599 | Down | U4/U6 small nuclear ribonucleoprotein Prp4 OS=Homo sapiens OX=9606 GN=PRPF4 PE=1 SV=2 | PRPF4 | _TK[Lac (K)]APDDLVAPVVK_ |
| O43172 | 26 | K | 0.224710961 | Down | U4/U6 small nuclear ribonucleoprotein Prp4 OS=Homo sapiens OX=9606 GN=PRPF4 PE=1 SV=2 | PRPF4 | _APDDLVAPVVK[Lac (K)]K_ |
| O43172 | 45 | K | 0.382284177 | Down | U4/U6 small nuclear ribonucleoprotein Prp4 OS=Homo sapiens OX=9606 GN=PRPF4 PE=1 SV=2 | PRPF4 | _LAK[Lac (K)]GESGILGK_ |
| O43172 | 53 | K | 0.457131377 | Down | U4/U6 small nuclear ribonucleoprotein Prp4 OS=Homo sapiens OX=9606 GN=PRPF4 PE=1 SV=2 | PRPF4 | _GESGILGK[Lac (K)]DGLK_ |
| O43172 | 146 | K | 0.34265933 | Down | U4/U6 small nuclear ribonucleoprotein Prp4 OS=Homo sapiens OX=9606 GN=PRPF4 PE=1 SV=2 | PRPF4 | _NILSVVGTDALK[Lac (K)]K_ |
| O43175 | 21 | K | 0.660426596 | Down | D-3-phosphoglycerate dehydrogenase OS=Homo sapiens OX=9606 GN=PHGDH PE=1 SV=4 | PHGDH | _K[Lac (K)]ILQDGGLQVVEK_ |
| O43237 | 440 | K | 0.413930817 | Down | Cytoplasmic dynein 1 light intermediate chain 2 OS=Homo sapiens OX=9606 GN=DYNC1LI2 PE=1 SV=1 | DYNC1LI2 | _K[Lac (K)]TGSPGSPGAGGVQSTAK_ |
| O43252 | 12 | K | 0.201344849 | Down | Bifunctional 3'-phosphoadenosine 5'-phosphosulfate synthase 1 OS=Homo sapiens OX=9606 GN=PAPSS1 PE=1 SV=2 | PAPSS1 | _VK[Lac (K)]LSNNAQNWGMQR_ |
| O43290 | 125 | K | 0.526521328 | Down | U4/U6.U5 tri-snRNP-associated protein 1 OS=Homo sapiens OX=9606 GN=SART1 PE=1 SV=1 | SART1 | _TSSGDASSLSIEETNK[Lac (K)]LR_ |
| O43290 | 129 | K | 0.404308295 | Down | U4/U6.U5 tri-snRNP-associated protein 1 OS=Homo sapiens OX=9606 GN=SART1 PE=1 SV=1 | SART1 | _AK[Lac (K)]LGLKPLEVNAIK_ |
| O43290 | 183 | K | 0.399673234 | Down | U4/U6.U5 tri-snRNP-associated protein 1 OS=Homo sapiens OX=9606 GN=SART1 PE=1 SV=1 | SART1 | _LLNQK[Lac (K)]LGK_ |
| O43290 | 336 | K | 0.251489787 | Down | U4/U6.U5 tri-snRNP-associated protein 1 OS=Homo sapiens OX=9606 GN=SART1 PE=1 SV=1 | SART1 | _SILSK[Lac (K)]YDEELEGERPHSFR_ |
| O43290 | 530 | K | 0.365806318 | Down | U4/U6.U5 tri-snRNP-associated protein 1 OS=Homo sapiens OX=9606 GN=SART1 PE=1 SV=1 | SART1 | _VVEIVK[Lac (K)]K_ |
| O43290 | 666 | K | 0.273881862 | Down | U4/U6.U5 tri-snRNP-associated protein 1 OS=Homo sapiens OX=9606 GN=SART1 PE=1 SV=1 | SART1 | _APNK[Lac (K)]SLPSAVYC[Carbamidomethyl (C)]IEDK_ |
| O43298 | 234 | K | 0.257123984 | Down | Zinc finger and BTB domain-containing protein 43 OS=Homo sapiens OX=9606 GN=ZBTB43 PE=1 SV=1 | ZBTB43 | _PMYSK[Lac (K)]PSIMAHK_ |
| O43390 | 114 | K | 1.745843469 | Up | Heterogeneous nuclear ribonucleoprotein R OS=Homo sapiens OX=9606 GN=HNRNPR PE=1 SV=1 | HNRNPR | _QGSK[Lac (K)]VQESTK_ |
| O43390 | 120 | K | 1.670016181 | Up | Heterogeneous nuclear ribonucleoprotein R OS=Homo sapiens OX=9606 GN=HNRNPR PE=1 SV=1 | HNRNPR | _VQESTK[Lac (K)]GPDEAK_ |
| O43395 | 92 | K | 0.271418816 | Down | U4/U6 small nuclear ribonucleoprotein Prp3 OS=Homo sapiens OX=9606 GN=PRPF3 PE=1 SV=2 | PRPF3 | _ELK[Lac (K)]EVFGDDSEISK_ |
| O43395 | 103 | K | 0.405864236 | Down | U4/U6 small nuclear ribonucleoprotein Prp3 OS=Homo sapiens OX=9606 GN=PRPF3 PE=1 SV=2 | PRPF3 | _EVFGDDSEISK[Lac (K)]ESSGVK_ |
| O43395 | 282 | K | 0.292461899 | Down | U4/U6 small nuclear ribonucleoprotein Prp3 OS=Homo sapiens OX=9606 GN=PRPF3 PE=1 SV=2 | PRPF3 | _MPTLK[Lac (K)]ANIR_ |
| O43395 | 294 | K | 0.246784846 | Down | U4/U6 small nuclear ribonucleoprotein Prp3 OS=Homo sapiens OX=9606 GN=PRPF3 PE=1 SV=2 | PRPF3 | _EQFK[Lac (K)]QQLK_ |
| O43395 | 328 | K | 0.217830956 | Down | U4/U6 small nuclear ribonucleoprotein Prp3 OS=Homo sapiens OX=9606 GN=PRPF3 PE=1 SV=2 | PRPF3 | _TFK[Lac (K)]FHDK_ |
| O43395 | 337 | K | 0.364062055 | Down | U4/U6 small nuclear ribonucleoprotein Prp3 OS=Homo sapiens OX=9606 GN=PRPF3 PE=1 SV=2 | PRPF3 | _FEK[Lac (K)]IAQR_ |
| O43395 | 376 | K | 0.412780508 | Down | U4/U6 small nuclear ribonucleoprotein Prp3 OS=Homo sapiens OX=9606 GN=PRPF3 PE=1 SV=2 | PRPF3 | _LALIAPK[Lac (K)]K_ |
| O43399 | 128 | K | 0.52879406 | Down | Tumor protein D54 OS=Homo sapiens OX=9606 GN=TPD52L2 PE=1 SV=2 | TPD52L2 | _K[Lac (K)]TQETLSQAGQK_ |
| O43399 | 165 | K | 0.312374945 | Down | Tumor protein D54 OS=Homo sapiens OX=9606 GN=TPD52L2 PE=1 SV=2 | TPD52L2 | _NSATFK[Lac (K)]SFEDR_ |
| O43432 | 714 | K | 0.591770701 | Down | Eukaryotic translation initiation factor 4 gamma 3 OS=Homo sapiens OX=9606 GN=EIF4G3 PE=1 SV=2 | EIF4G3 | _K[Lac (K)]IITVSVK_ |
| O43491 | 601 | K | 0.596231003 | Down | Band 4.1-like protein 2 OS=Homo sapiens OX=9606 GN=EPB41L2 PE=1 SV=1 | EPB41L2 | _SPTK[Lac (K)]APHLQLIEGK_ |
| O43633 | 94 | K | 0.290991918 | Down | Charged multivesicular body protein 2a OS=Homo sapiens OX=9606 GN=CHMP2A PE=1 SV=1 | CHMP2A | _IQTLK[Lac (K)]SNNSMAQAMK_ |
| O43633 | 197 | K | 0.305178322 | Down | Charged multivesicular body protein 2a OS=Homo sapiens OX=9606 GN=CHMP2A PE=1 SV=1 | CHMP2A | _K[Lac (K)]AEAAASALADADADLEER_ |
| O43660 | 180 | K | 0.273281018 | Down | Pleiotropic regulator 1 OS=Homo sapiens OX=9606 GN=PLRG1 PE=1 SV=1 | PLRG1 | _NSALMAK[Lac (K)]K_ |
| O43663 | 534 | K | 2.111810576 | Up | Protein regulator of cytokinesis 1 OS=Homo sapiens OX=9606 GN=PRC1 PE=1 SV=2 | PRC1 | _PVAASTC[Carbamidomethyl (C)]SGK[Lac (K)]K_ |
| O43665 | 154 | K | 0.365143907 | Down | Regulator of G-protein signaling 10 OS=Homo sapiens OX=9606 GN=RGS10 PE=1 SV=3 | RGS10 | _SDLFLK[Lac (K)]HK_ |
| O43665 | 173 | K | 0.35313893 | Down | Regulator of G-protein signaling 10 OS=Homo sapiens OX=9606 GN=RGS10 PE=1 SV=3 | RGS10 | _TEEEEEDLPDAQTAAK[Lac (K)]R_ |
| O43719 | 119 | K | 0.320405294 | Down | HIV Tat-specific factor 1 OS=Homo sapiens OX=9606 GN=HTATSF1 PE=1 SV=1 | HTATSF1 | _K[Lac (K)]AESGWFHVEEDR_ |
| O43719 | 221 | K | 0.364455148 | Down | HIV Tat-specific factor 1 OS=Homo sapiens OX=9606 GN=HTATSF1 PE=1 SV=1 | HTATSF1 | _FQLK[Lac (K)]GEYDASK_ |
| O43719 | 239 | K | 0.244487626 | Down | HIV Tat-specific factor 1 OS=Homo sapiens OX=9606 GN=HTATSF1 PE=1 SV=1 | HTATSF1 | _K[Lac (K)]LSMQQK_ |
| O43719 | 297 | K | 0.60915337 | Down | HIV Tat-specific factor 1 OS=Homo sapiens OX=9606 GN=HTATSF1 PE=1 SV=1 | HTATSF1 | _VEC[Carbamidomethyl (C)]SK[Lac (K)]FGQIR_ |
| O43719 | 303 | K | 0.567506083 | Down | HIV Tat-specific factor 1 OS=Homo sapiens OX=9606 GN=HTATSF1 PE=1 SV=1 | HTATSF1 | _K[Lac (K)]LLLFDR_ |
| O43719 | 509 | K | 0.421821733 | Down | HIV Tat-specific factor 1 OS=Homo sapiens OX=9606 GN=HTATSF1 PE=1 SV=1 | HTATSF1 | _TLK[Lac (K)]NDC[Carbamidomethyl (C)]EENGLAK_ |
| O43768 | 74 | K | 0.235673273 | Down | Alpha-endosulfine OS=Homo sapiens OX=9606 GN=ENSA PE=1 SV=1 | ENSA | _YFDSGDYNMAK[Lac (K)]AK_ |
| O43768 | 80 | K | 0.376092839 | Down | Alpha-endosulfine OS=Homo sapiens OX=9606 GN=ENSA PE=1 SV=1 | ENSA | _NK[Lac (K)]QLPSAGPDK_ |
| O43768 | 107 | K | 0.483574779 | Down | Alpha-endosulfine OS=Homo sapiens OX=9606 GN=ENSA PE=1 SV=1 | ENSA | _K[Lac (K)]SSLVTSK_ |
| O43813 | 128 | K | 2.083374274 | Up | Glutathione S-transferase LANCL1 OS=Homo sapiens OX=9606 GN=LANCL1 PE=1 SV=1 | LANCL1 | _MNNEK[Lac (K)]QAEDC[Carbamidomethyl (C)]ITR_ |
| O43815 | 500 | K | 0.477132383 | Down | Striatin OS=Homo sapiens OX=9606 GN=STRN PE=1 SV=4 | STRN | _K[Lac (K)]STSLDVEPIYTFR_ |
| O43818 | 25 | K | 0.578026108 | Down | U3 small nucleolar RNA-interacting protein 2 OS=Homo sapiens OX=9606 GN=RRP9 PE=1 SV=1 | RRP9 | _GKPASGAGAGAGAGK[Lac (K)]R_ |
| O43818 | 84 | K | 0.663146386 | Down | U3 small nucleolar RNA-interacting protein 2 OS=Homo sapiens OX=9606 GN=RRP9 PE=1 SV=1 | RRP9 | _LAK[Lac (K)]LYLEQLR_ |
| O43823 | 306 | K | 0.35491751 | Down | A-kinase anchor protein 8 OS=Homo sapiens OX=9606 GN=AKAP8 PE=1 SV=1 | AKAP8 | _K[Lac (K)]QFQLYEEPDTK_ |
| O43865 | 36 | K | 0.399686538 | Down | S-adenosylhomocysteine hydrolase-like protein 1 OS=Homo sapiens OX=9606 GN=AHCYL1 PE=1 SV=2 | AHCYL1 | _YSFMATVTK[Lac (K)]APK_ |
| O43865 | 40 | K | 0.139579602 | Down | S-adenosylhomocysteine hydrolase-like protein 1 OS=Homo sapiens OX=9606 GN=AHCYL1 PE=1 SV=2 | AHCYL1 | _K[Lac (K)]QIQFADDMQEFTK_ |
| O43896 | 294 | K | 0.453440861 | Down | Kinesin-like protein KIF1C OS=Homo sapiens OX=9606 GN=KIF1C PE=1 SV=3 | KIF1C | _K[Lac (K)]SDFIPYR_ |
| O43929 | 7 | K | 0.474237771 | Down | Origin recognition complex subunit 4 OS=Homo sapiens OX=9606 GN=ORC4 PE=1 SV=2 | ORC4 | _SK[Lac (K)]SNSLIHTEC[Carbamidomethyl (C)]LSQVQR_ |
| O60216 | 323 | K | 0.489421856 | Down | Double-strand-break repair protein rad21 homolog OS=Homo sapiens OX=9606 GN=RAD21 PE=1 SV=2 | RAD21 | _K[Lac (K)]LIVDSVK_ |
| O60216 | 477 | K | 0.406354518 | Down | Double-strand-break repair protein rad21 homolog OS=Homo sapiens OX=9606 GN=RAD21 PE=1 SV=2 | RAD21 | _TNIDESAMPPPPPQGVK[Lac (K)]R_ |
| O60231 | 73 | K | 0.408651794 | Down | Pre-mRNA-splicing factor ATP-dependent RNA helicase DHX16 OS=Homo sapiens OX=9606 GN=DHX16 PE=1 SV=2 | DHX16 | _K[Lac (K)]AVVEKPAR_ |
| O60231 | 143 | K | 0.579019843 | Down | Pre-mRNA-splicing factor ATP-dependent RNA helicase DHX16 OS=Homo sapiens OX=9606 GN=DHX16 PE=1 SV=2 | DHX16 | _EEEEEEEASEK[Lac (K)]GK_ |
| O60231 | 210 | K | 0.582442652 | Down | Pre-mRNA-splicing factor ATP-dependent RNA helicase DHX16 OS=Homo sapiens OX=9606 GN=DHX16 PE=1 SV=2 | DHX16 | _AYEEAQK[Lac (K)]R_ |
| O60231 | 213 | K | 0.299792663 | Down | Pre-mRNA-splicing factor ATP-dependent RNA helicase DHX16 OS=Homo sapiens OX=9606 GN=DHX16 PE=1 SV=2 | DHX16 | _LK[Lac (K)]MAEEDRK_ |
| O60231 | 291 | K | 0.209288625 | Down | Pre-mRNA-splicing factor ATP-dependent RNA helicase DHX16 OS=Homo sapiens OX=9606 GN=DHX16 PE=1 SV=2 | DHX16 | _AAGEQEK[Lac (K)]LEATNR_ |
| O60231 | 340 | K | 0.352118721 | Down | Pre-mRNA-splicing factor ATP-dependent RNA helicase DHX16 OS=Homo sapiens OX=9606 GN=DHX16 PE=1 SV=2 | DHX16 | _LGAASLK[Lac (K)]FGAR_ |
| O60264 | 119 | K | 0.661368638 | Down | SWI/SNF-related matrix-associated actin-dependent regulator of chromatin subfamily A member 5 OS=Homo sapiens OX=9606 GN=SMARCA5 PE=1 SV=1 | SMARCA5 | _TPTSPLK[Lac (K)]MK_ |
| O60264 | 132 | K | 0.453859857 | Down | SWI/SNF-related matrix-associated actin-dependent regulator of chromatin subfamily A member 5 OS=Homo sapiens OX=9606 GN=SMARCA5 PE=1 SV=1 | SMARCA5 | _DEK[Lac (K)]QNLLSVGDYR_ |
| O60264 | 440 | K | 0.454491053 | Down | SWI/SNF-related matrix-associated actin-dependent regulator of chromatin subfamily A member 5 OS=Homo sapiens OX=9606 GN=SMARCA5 PE=1 SV=1 | SMARCA5 | _DIDILNSAGK[Lac (K)]MDK_ |
| O60264 | 644 | K | 0.556210664 | Down | SWI/SNF-related matrix-associated actin-dependent regulator of chromatin subfamily A member 5 OS=Homo sapiens OX=9606 GN=SMARCA5 PE=1 SV=1 | SMARCA5 | _LVDQNLNK[Lac (K)]IGK_ |
| O60264 | 691 | K | 0.5706332 | Down | SWI/SNF-related matrix-associated actin-dependent regulator of chromatin subfamily A member 5 OS=Homo sapiens OX=9606 GN=SMARCA5 PE=1 SV=1 | SMARCA5 | _TAEMNEK[Lac (K)]LSK_ |
| O60264 | 739 | K | 0.272588436 | Down | SWI/SNF-related matrix-associated actin-dependent regulator of chromatin subfamily A member 5 OS=Homo sapiens OX=9606 GN=SMARCA5 PE=1 SV=1 | SMARCA5 | _K[Lac (K)]ANYAVDAYFR_ |
| O60264 | 1036 | K | 0.575736839 | Down | SWI/SNF-related matrix-associated actin-dependent regulator of chromatin subfamily A member 5 OS=Homo sapiens OX=9606 GN=SMARCA5 PE=1 SV=1 | SMARCA5 | _K[Lac (K)]MDGAPDGR_ |
| O60271 | 373 | K | 0.64204682 | Down | C-Jun-amino-terminal kinase-interacting protein 4 OS=Homo sapiens OX=9606 GN=SPAG9 PE=1 SV=4 | SPAG9 | _GIENK[Lac (K)]AFDR_ |
| O60271 | 603 | K | 0.510816388 | Down | C-Jun-amino-terminal kinase-interacting protein 4 OS=Homo sapiens OX=9606 GN=SPAG9 PE=1 SV=4 | SPAG9 | _SSTLSQLPGDK[Lac (K)]SK_ |
| O60287 | 7 | K | 0.456512358 | Down | Nucleolar pre-ribosomal-associated protein 1 OS=Homo sapiens OX=9606 GN=URB1 PE=1 SV=4 | URB1 | _K[Lac (K)]ASGGQDGAASSAGAAK_ |
| O60287 | 27 | K | 0.616321241 | Down | Nucleolar pre-ribosomal-associated protein 1 OS=Homo sapiens OX=9606 GN=URB1 PE=1 SV=4 | URB1 | _K[Lac (K)]EELTGVR_ |
| O60292 | 1115 | K | 0.356744287 | Down | Signal-induced proliferation-associated 1-like protein 3 OS=Homo sapiens OX=9606 GN=SIPA1L3 PE=1 SV=3 | SIPA1L3 | _PLK[Lac (K)]QTPIVPFR_ |
| O60293 | 490 | K | 0.397144579 | Down | Zinc finger C3H1 domain-containing protein OS=Homo sapiens OX=9606 GN=ZFC3H1 PE=1 SV=3 | ZFC3H1 | _FMK[Lac (K)]LVGGK_ |
| O60293 | 687 | K | 0.366075553 | Down | Zinc finger C3H1 domain-containing protein OS=Homo sapiens OX=9606 GN=ZFC3H1 PE=1 SV=3 | ZFC3H1 | _IQNPK[Lac (K)]FHR_ |
| O60306 | 762 | K | 0.637184369 | Down | RNA helicase aquarius OS=Homo sapiens OX=9606 GN=AQR PE=1 SV=4 | AQR | _K[Lac (K)]DADVEDEDTEEAK_ |
| O60318 | 555 | K | 0.367067686 | Down | Germinal-center associated nuclear protein OS=Homo sapiens OX=9606 GN=MCM3AP PE=1 SV=2 | MCM3AP | _TGASSLLNK[Lac (K)]SSPVK_ |
| O60343 | 653 | K | 0.594287719 | Down | TBC1 domain family member 4 OS=Homo sapiens OX=9606 GN=TBC1D4 PE=1 SV=2 | TBC1D4 | _K[Lac (K)]LNLQDGR_ |
| O60361 | 109 | K | 1.974234935 | Up | Putative nucleoside diphosphate kinase OS=Homo sapiens OX=9606 GN=NME2P1 PE=5 SV=1 | NME2P1 | _NIIHGSDSVK[Lac (K)]SAEK_ |
| O60502 | 597 | K | 0.49132055 | Down | Protein O-GlcNAcase OS=Homo sapiens OX=9606 GN=OGA PE=1 SV=2 | OGA | _ANSSVVSVNC[Carbamidomethyl (C)]K[Lac (K)]GK_ |
| O60508 | 42 | K | 0.627485727 | Down | Pre-mRNA-processing factor 17 OS=Homo sapiens OX=9606 GN=CDC40 PE=1 SV=1 | CDC40 | _C[Carbamidomethyl (C)]PLPAADSLMHLTK[Lac (K)]SPSSK_ |
| O60508 | 173 | K | 0.276654778 | Down | Pre-mRNA-processing factor 17 OS=Homo sapiens OX=9606 GN=CDC40 PE=1 SV=1 | CDC40 | _NQGLTVFETGQK[Lac (K)]K_ |
| O60524 | 799 | K | 1.704617694 | Up | Ribosome quality control complex subunit NEMF OS=Homo sapiens OX=9606 GN=NEMF PE=1 SV=4 | NEMF | _LASK[Lac (K)]EESSNSSDSK_ |
| O60563 | 380 | K | 0.548650716 | Down | Cyclin-T1 OS=Homo sapiens OX=9606 GN=CCNT1 PE=1 SV=1 | CCNT1 | _QNSK[Lac (K)]SVPSAK_ |
| O60563 | 386 | K | 0.276771621 | Down | Cyclin-T1 OS=Homo sapiens OX=9606 GN=CCNT1 PE=1 SV=1 | CCNT1 | _SVPSAK[Lac (K)]VSLK_ |
| O60563 | 471 | K | 0.222051631 | Down | Cyclin-T1 OS=Homo sapiens OX=9606 GN=CCNT1 PE=1 SV=1 | CCNT1 | _IPVAGGDK[Lac (K)]AASSKPEEIK_ |
| O60563 | 492 | K | 0.412227895 | Down | Cyclin-T1 OS=Homo sapiens OX=9606 GN=CCNT1 PE=1 SV=1 | CCNT1 | _VHAAADK[Lac (K)]HNSVEDSVTK_ |
| O60563 | 540 | K | 0.203453989 | Down | Cyclin-T1 OS=Homo sapiens OX=9606 GN=CCNT1 PE=1 SV=1 | CCNT1 | _HSHSQLPVGTGNK[Lac (K)]R_ |
| O60563 | 590 | K | 0.176006732 | Down | Cyclin-T1 OS=Homo sapiens OX=9606 GN=CCNT1 PE=1 SV=1 | CCNT1 | _GPSEETGGAVFDHPAK[Lac (K)]IAK_ |
| O60563 | 638 | K | 0.405613631 | Down | Cyclin-T1 OS=Homo sapiens OX=9606 GN=CCNT1 PE=1 SV=1 | CCNT1 | _VPHSK[Lac (K)]LDK_ |
| O60583 | 438 | K | 0.332204965 | Down | Cyclin-T2 OS=Homo sapiens OX=9606 GN=CCNT2 PE=1 SV=2 | CCNT2 | _MSLDK[Lac (K)]YR_ |
| O60610 | 35 | K | 0.315196765 | Down | Protein diaphanous homolog 1 OS=Homo sapiens OX=9606 GN=DIAPH1 PE=1 SV=2 | DIAPH1 | _SPDELPSAGGDGGK[Lac (K)]SK_ |
| O60610 | 1252 | K | 0.211603643 | Down | Protein diaphanous homolog 1 OS=Homo sapiens OX=9606 GN=DIAPH1 PE=1 SV=2 | DIAPH1 | _VSK[Lac (K)]NSETFPTILEEAK_ |
| O60664 | 65 | K | 1.539145865 | Up | Perilipin-3 OS=Homo sapiens OX=9606 GN=PLIN3 PE=1 SV=3 | PLIN3 | _TVC[Carbamidomethyl (C)]DAAEK[Lac (K)]GVR_ |
| O60664 | 166 | K | 0.566169928 | Down | Perilipin-3 OS=Homo sapiens OX=9606 GN=PLIN3 PE=1 SV=3 | PLIN3 | _TK[Lac (K)]SVVTGGVQSVMGSR_ |
| O60684 | 18 | K | 0.254098187 | Down | Importin subunit alpha-7 OS=Homo sapiens OX=9606 GN=KPNA6 PE=1 SV=1 | KPNA6 | _SYK[Lac (K)]NNALNPEEMR_ |
| O60684 | 49 | K | 0.122391201 | Down | Importin subunit alpha-7 OS=Homo sapiens OX=9606 GN=KPNA6 PE=1 SV=1 | KPNA6 | _EQQLFK[Lac (K)]R_ |
| O60701 | 490 | K | 1.546530894 | Up | UDP-glucose 6-dehydrogenase OS=Homo sapiens OX=9606 GN=UGDH PE=1 SV=1 | UGDH | _FSLQDPPNK[Lac (K)]KPK_ |
| O60716 | 633 | K | 0.518127348 | Down | Catenin delta-1 OS=Homo sapiens OX=9606 GN=CTNND1 PE=1 SV=1 | CTNND1 | _GK[Lac (K)]KPIEDPANDTVDFPK_ |
| O60749 | 116 | K | 0.363491206 | Down | Sorting nexin-2 OS=Homo sapiens OX=9606 GN=SNX2 PE=1 SV=2 | SNX2 | _IESK[Lac (K)]SMSAPVIFDR_ |
| O60828 | 223 | K | 0.60344927 | Down | Polyglutamine-binding protein 1 OS=Homo sapiens OX=9606 GN=PQBP1 PE=1 SV=1 | PQBP1 | _GTWSTGLPK[Lac (K)]R_ |
| O60828 | 228 | K | 0.502083302 | Down | Polyglutamine-binding protein 1 OS=Homo sapiens OX=9606 GN=PQBP1 PE=1 SV=1 | PQBP1 | _NEAK[Lac (K)]TGADTTAAGPLFQQR_ |
| O60832 | 46 | K | 0.433025632 | Down | H/ACA ribonucleoprotein complex subunit DKC1 OS=Homo sapiens OX=9606 GN=DKC1 PE=1 SV=3 | DKC1 | _VAK[Lac (K)]LDTSQWPLLLK_ |
| O60832 | 394 | K | 0.563024488 | Down | H/ACA ribonucleoprotein complex subunit DKC1 OS=Homo sapiens OX=9606 GN=DKC1 PE=1 SV=3 | DKC1 | _LMIK[Lac (K)]QGLLDK_ |
| O60841 | 50 | K | 0.566502719 | Down | Eukaryotic translation initiation factor 5B OS=Homo sapiens OX=9606 GN=EIF5B PE=1 SV=4 | EIF5B | _K[Lac (K)]QDFDEDDILK_ |
| O60841 | 424 | K | 0.459775165 | Down | Eukaryotic translation initiation factor 5B OS=Homo sapiens OX=9606 GN=EIF5B PE=1 SV=4 | EIF5B | _AEATLK[Lac (K)]LLQAQGVEVPSK_ |
| O60841 | 436 | K | 0.618915655 | Down | Eukaryotic translation initiation factor 5B OS=Homo sapiens OX=9606 GN=EIF5B PE=1 SV=4 | EIF5B | _LLQAQGVEVPSK[Lac (K)]DSLPK_ |
| O60869 | 23 | K | 1.730806308 | Up | Endothelial differentiation-related factor 1 OS=Homo sapiens OX=9606 GN=EDF1 PE=1 SV=1 | EDF1 | _GPTAAQAK[Lac (K)]SK_ |
| O60869 | 143 | K | 0.436679981 | Down | Endothelial differentiation-related factor 1 OS=Homo sapiens OX=9606 GN=EDF1 PE=1 SV=1 | EDF1 | _DIGKPIEK[Lac (K)]GPR_ |
| O60870 | 10 | K | 0.149498743 | Down | DNA/RNA-binding protein KIN17 OS=Homo sapiens OX=9606 GN=KIN PE=1 SV=2 | KIN | _SDFLTPK[Lac (K)]AIANR_ |
| O60870 | 171 | K | 0.447481122 | Down | DNA/RNA-binding protein KIN17 OS=Homo sapiens OX=9606 GN=KIN PE=1 SV=2 | KIN | _TAK[Lac (K)]FIEEQVR_ |
| O60870 | 209 | K | 0.508158784 | Down | DNA/RNA-binding protein KIN17 OS=Homo sapiens OX=9606 GN=KIN PE=1 SV=2 | KIN | _VTFNLSK[Lac (K)]GAC[Carbamidomethyl (C)]SSSGATSSK_ |
| O60870 | 231 | K | 0.168065163 | Down | DNA/RNA-binding protein KIN17 OS=Homo sapiens OX=9606 GN=KIN PE=1 SV=2 | KIN | _SSTLGPSALK[Lac (K)]TIGSSASVK_ |
| O60870 | 240 | K | 0.343027366 | Down | DNA/RNA-binding protein KIN17 OS=Homo sapiens OX=9606 GN=KIN PE=1 SV=2 | KIN | _TIGSSASVK[Lac (K)]R_ |
| O60884 | 152 | K | 0.347318315 | Down | DnaJ homolog subfamily A member 2 OS=Homo sapiens OX=9606 GN=DNAJA2 PE=1 SV=1 | DNAJA2 | _NVLC[Carbamidomethyl (C)]SAC[Carbamidomethyl (C)]SGQGGK[Lac (K)]SGAVQK_ |
| O60885 | 1197 | K | 0.080493117 | Down | Bromodomain-containing protein 4 OS=Homo sapiens OX=9606 GN=BRD4 PE=1 SV=2 | BRD4 | _IK[Lac (K)]NMGSWASLVQK_ |
| O60885 | 1208 | K | 0.172861609 | Down | Bromodomain-containing protein 4 OS=Homo sapiens OX=9606 GN=BRD4 PE=1 SV=2 | BRD4 | _NMGSWASLVQK[Lac (K)]HPTTPSSTAK_ |
| O60927 | 40 | K | 0.23273833 | Down | E3 ubiquitin-protein ligase PPP1R11 OS=Homo sapiens OX=9606 GN=PPP1R11 PE=1 SV=1 | PPP1R11 | _K[Lac (K)]VEWTSDTVDNEHMGR_ |
| O60942 | 220 | K | 0.490352809 | Down | mRNA-capping enzyme OS=Homo sapiens OX=9606 GN=RNGTT PE=1 SV=1 | RNGTT | _ESEPGSSASFGK[Lac (K)]R_ |
| O60942 | 575 | K | 0.112517138 | Down | mRNA-capping enzyme OS=Homo sapiens OX=9606 GN=RNGTT PE=1 SV=1 | RNGTT | _K[Lac (K)]HHLDPDTELMPPPPPK_ |
| O60942 | 591 | K | 0.367486163 | Down | mRNA-capping enzyme OS=Homo sapiens OX=9606 GN=RNGTT PE=1 SV=1 | RNGTT | _HHLDPDTELMPPPPPK[Lac (K)]RPR_ |
| O75150 | 517 | K | 0.423344515 | Down | E3 ubiquitin-protein ligase BRE1B OS=Homo sapiens OX=9606 GN=RNF40 PE=1 SV=5 | RNF40 | _EVQAEIGK[Lac (K)]LR_ |
| O75150 | 562 | K | 0.418715006 | Down | E3 ubiquitin-protein ligase BRE1B OS=Homo sapiens OX=9606 GN=RNF40 PE=1 SV=5 | RNF40 | _K[Lac (K)]EMAPVPGTTTTTTSVK_ |
| O75151 | 569 | K | 0.406383308 | Down | Lysine-specific demethylase PHF2 OS=Homo sapiens OX=9606 GN=PHF2 PE=1 SV=4 | PHF2 | _ATK[Lac (K)]SVLSVPNK_ |
| O75151 | 606 | K | 0.463876142 | Down | Lysine-specific demethylase PHF2 OS=Homo sapiens OX=9606 GN=PHF2 PE=1 SV=4 | PHF2 | _YK[Lac (K)]NSKPDSLLK_ |
| O75151 | 711 | K | 0.341107187 | Down | Lysine-specific demethylase PHF2 OS=Homo sapiens OX=9606 GN=PHF2 PE=1 SV=4 | PHF2 | _K[Lac (K)]AVLPTPVTKPK_ |
| O75152 | 288 | K | 0.311400989 | Down | Zinc finger CCCH domain-containing protein 11A OS=Homo sapiens OX=9606 GN=ZC3H11A PE=1 SV=3 | ZC3H11A | _K[Lac (K)]FSAGGDSDPPLKR_ |
| O75152 | 300 | K | 0.532280091 | Down | Zinc finger CCCH domain-containing protein 11A OS=Homo sapiens OX=9606 GN=ZC3H11A PE=1 SV=3 | ZC3H11A | _FSAGGDSDPPLK[Lac (K)]R_ |
| O75152 | 381 | K | 0.562447201 | Down | Zinc finger CCCH domain-containing protein 11A OS=Homo sapiens OX=9606 GN=ZC3H11A PE=1 SV=3 | ZC3H11A | _LK[Lac (K)]TEGPSK_ |
| O75152 | 413 | K | 0.393612955 | Down | Zinc finger CCCH domain-containing protein 11A OS=Homo sapiens OX=9606 GN=ZC3H11A PE=1 SV=3 | ZC3H11A | _TFSEVLAEK[Lac (K)]K_ |
| O75152 | 427 | K | 0.629135068 | Down | Zinc finger CCCH domain-containing protein 11A OS=Homo sapiens OX=9606 GN=ZC3H11A PE=1 SV=3 | ZC3H11A | _K[Lac (K)]DTTC[Carbamidomethyl (C)]IK_ |
| O75152 | 435 | K | 0.410150681 | Down | Zinc finger CCCH domain-containing protein 11A OS=Homo sapiens OX=9606 GN=ZC3H11A PE=1 SV=3 | ZC3H11A | _LK[Lac (K)]IDSEIKK_ |
| O75152 | 553 | K | 0.623495194 | Down | Zinc finger CCCH domain-containing protein 11A OS=Homo sapiens OX=9606 GN=ZC3H11A PE=1 SV=3 | ZC3H11A | _EASGETTGVDITK[Lac (K)]IQVK_ |
| O75152 | 575 | K | 0.278639452 | Down | Zinc finger CCCH domain-containing protein 11A OS=Homo sapiens OX=9606 GN=ZC3H11A PE=1 SV=3 | ZC3H11A | _EK[Lac (K)]SVLTPLR_ |
| O75152 | 615 | K | 0.540492729 | Down | Zinc finger CCCH domain-containing protein 11A OS=Homo sapiens OX=9606 GN=ZC3H11A PE=1 SV=3 | ZC3H11A | _RLPTK[Lac (K)]SSQK_ |
| O75152 | 659 | K | 0.315214911 | Down | Zinc finger CCCH domain-containing protein 11A OS=Homo sapiens OX=9606 GN=ZC3H11A PE=1 SV=3 | ZC3H11A | _VNVKPSVVK[Lac (K)]VVSSPK_ |
| O75152 | 665 | K | 0.303328072 | Down | Zinc finger CCCH domain-containing protein 11A OS=Homo sapiens OX=9606 GN=ZC3H11A PE=1 SV=3 | ZC3H11A | _VVSSPK[Lac (K)]LAPK_ |
| O75152 | 699 | K | 0.43187878 | Down | Zinc finger CCCH domain-containing protein 11A OS=Homo sapiens OX=9606 GN=ZC3H11A PE=1 SV=3 | ZC3H11A | _K[Lac (K)]AAVAVVPLVSEDK_ |
| O75164 | 885 | K | 0.506062283 | Down | Lysine-specific demethylase 4A OS=Homo sapiens OX=9606 GN=KDM4A PE=1 SV=2 | KDM4A | _HK[Lac (K)]IPNLER_ |
| O75164 | 1012 | K | 0.445482097 | Down | Lysine-specific demethylase 4A OS=Homo sapiens OX=9606 GN=KDM4A PE=1 SV=2 | KDM4A | _RDDVYTLDEELPK[Lac (K)]R_ |
| O75179 | 63 | K | 0.612417026 | Down | Ankyrin repeat domain-containing protein 17 OS=Homo sapiens OX=9606 GN=ANKRD17 PE=1 SV=3 | ANKRD17 | _VC[Carbamidomethyl (C)]DLLLK[Lac (K)]K_ |
| O75182 | 292 | K | 0.305514419 | Down | Paired amphipathic helix protein Sin3b OS=Homo sapiens OX=9606 GN=SIN3B PE=1 SV=2 | SIN3B | _LRGTK[Lac (K)]DLSIAAVGK_ |
| O75182 | 319 | K | 0.211395191 | Down | Paired amphipathic helix protein Sin3b OS=Homo sapiens OX=9606 GN=SIN3B PE=1 SV=2 | SIN3B | _VLK[Lac (K)]SQEVYENFLR_ |
| O75391 | 166 | K | 0.514565556 | Down | Sperm-associated antigen 7 OS=Homo sapiens OX=9606 GN=SPAG7 PE=1 SV=2 | SPAG7 | _DK[Lac (K)]YSHLIGK_ |
| O75396 | 169 | K | 0.584454967 | Down | Vesicle-trafficking protein SEC22b OS=Homo sapiens OX=9606 GN=SEC22B PE=1 SV=5 | SEC22B | _GEALSALDSK[Lac (K)]ANNLSSLSK_ |
| O75400 | 184 | K | 0.517417534 | Down | Pre-mRNA-processing factor 40 homolog A OS=Homo sapiens OX=9606 GN=PRPF40A PE=1 SV=2 | PRPF40A | _TPAEQLLSK[Lac (K)]C[Carbamidomethyl (C)]PWK_ |
| O75410 | 360 | K | 0.509964262 | Down | Transforming acidic coiled-coil-containing protein 1 OS=Homo sapiens OX=9606 GN=TACC1 PE=1 SV=2 | TACC1 | _DGISK[Lac (K)]SAGLEQPTDPVAR_ |
| O75410 | 517 | K | 0.571067222 | Down | Transforming acidic coiled-coil-containing protein 1 OS=Homo sapiens OX=9606 GN=TACC1 PE=1 SV=2 | TACC1 | _LASTSC[Carbamidomethyl (C)]GQK[Lac (K)]SAGAEVK_ |
| O75427 | 430 | K | 0.15763664 | Down | Leucine-rich repeat and calponin homology domain-containing protein 4 OS=Homo sapiens OX=9606 GN=LRCH4 PE=1 SV=2 | LRCH4 | _K[Lac (K)]DSLLKPGLR_ |
| O75449 | 44 | K | 0.299933143 | Down | Katanin p60 ATPase-containing subunit A1 OS=Homo sapiens OX=9606 GN=KATNA1 PE=1 SV=1 | KATNA1 | _YLYSVK[Lac (K)]DTYLQQK_ |
| O75475 | 189 | K | 0.604758967 | Down | PC4 and SFRS1-interacting protein OS=Homo sapiens OX=9606 GN=PSIP1 PE=1 SV=1 | PSIP1 | _PAATEVK[Lac (K)]IPK_ |
| O75475 | 317 | K | 0.465862783 | Down | PC4 and SFRS1-interacting protein OS=Homo sapiens OX=9606 GN=PSIP1 PE=1 SV=1 | PSIP1 | _K[Lac (K)]QEEQMETEQQNKDEGK_ |
| O75475 | 407 | K | 0.315305772 | Down | PC4 and SFRS1-interacting protein OS=Homo sapiens OX=9606 GN=PSIP1 PE=1 SV=1 | PSIP1 | _FK[Lac (K)]VSQVIMEK_ |
| O75496 | 27 | K | 0.30464487 | Down | Geminin OS=Homo sapiens OX=9606 GN=GMNN PE=1 SV=1 | GMNN | _TLK[Lac (K)]MIQPSASGSLVGR_ |
| O75530 | 19 | K | 0.602056563 | Down | Polycomb protein EED OS=Homo sapiens OX=9606 GN=EED PE=1 SV=2 | EED | _EVSTAPAGTDMPAAK[Lac (K)]K_ |
| O75533 | 6 | K | 0.412216783 | Down | Splicing factor 3B subunit 1 OS=Homo sapiens OX=9606 GN=SF3B1 PE=1 SV=3 | SF3B1 | _IAK[Lac (K)]THEDIEAQIR_ |
| O75533 | 141 | K | 0.360181678 | Down | Splicing factor 3B subunit 1 OS=Homo sapiens OX=9606 GN=SF3B1 PE=1 SV=3 | SF3B1 | _LDPFADGGK[Lac (K)]TPDPK_ |
| O75533 | 146 | K | 0.540707193 | Down | Splicing factor 3B subunit 1 OS=Homo sapiens OX=9606 GN=SF3B1 PE=1 SV=3 | SF3B1 | _TPDPK[Lac (K)]MNAR_ |
| O75533 | 163 | K | 0.627902852 | Down | Splicing factor 3B subunit 1 OS=Homo sapiens OX=9606 GN=SF3B1 PE=1 SV=3 | SF3B1 | _EQHLTK[Lac (K)]EER_ |
| O75533 | 182 | K | 0.600509956 | Down | Splicing factor 3B subunit 1 OS=Homo sapiens OX=9606 GN=SF3B1 PE=1 SV=3 | SF3B1 | _AGELK[Lac (K)]VVNGAAASQPPSK_ |
| O75533 | 213 | K | 0.404527476 | Down | Splicing factor 3B subunit 1 OS=Homo sapiens OX=9606 GN=SF3B1 PE=1 SV=3 | SF3B1 | _WDQTADQTPGATPK[Lac (K)]K_ |
| O75533 | 214 | K | 0.491640058 | Down | Splicing factor 3B subunit 1 OS=Homo sapiens OX=9606 GN=SF3B1 PE=1 SV=3 | SF3B1 | _K[Lac (K)]LSSWDQAETPGHTPSLR_ |
| O75533 | 298 | K | 0.659431704 | Down | Splicing factor 3B subunit 1 OS=Homo sapiens OX=9606 GN=SF3B1 PE=1 SV=3 | SF3B1 | _WDETPK[Lac (K)]TER_ |
| O75533 | 333 | K | 0.662100608 | Down | Splicing factor 3B subunit 1 OS=Homo sapiens OX=9606 GN=SF3B1 PE=1 SV=3 | SF3B1 | _GGDSIGETPTPGASK[Lac (K)]R_ |
| O75533 | 430 | K | 0.289985946 | Down | Splicing factor 3B subunit 1 OS=Homo sapiens OX=9606 GN=SF3B1 PE=1 SV=3 | SF3B1 | _K[Lac (K)]LTATPTPLGGMTGFHMQTEDR_ |
| O75534 | 166 | K | 0.531032332 | Down | Cold shock domain-containing protein E1 OS=Homo sapiens OX=9606 GN=CSDE1 PE=1 SV=2 | CSDE1 | _INFVIDNNK[Lac (K)]HTGAVSAR_ |
| O75534 | 180 | K | 0.351775194 | Down | Cold shock domain-containing protein E1 OS=Homo sapiens OX=9606 GN=CSDE1 PE=1 SV=2 | CSDE1 | _NIMLLK[Lac (K)]K_ |
| O75534 | 277 | K | 0.369937051 | Down | Cold shock domain-containing protein E1 OS=Homo sapiens OX=9606 GN=CSDE1 PE=1 SV=2 | CSDE1 | _VPSK[Lac (K)]NQNDPLPGR_ |
| O75534 | 758 | K | 0.531926896 | Down | Cold shock domain-containing protein E1 OS=Homo sapiens OX=9606 GN=CSDE1 PE=1 SV=2 | CSDE1 | _LK[Lac (K)]NITLDDASAPR_ |
| O75554 | 6 | K | 0.183279753 | Down | WW domain-binding protein 4 OS=Homo sapiens OX=9606 GN=WBP4 PE=1 SV=1 | WBP4 | _[Acetyl (Protein N-term)]ADYWK[Lac (K)]SQPK_ |
| O75554 | 11 | K | 0.413842131 | Down | WW domain-binding protein 4 OS=Homo sapiens OX=9606 GN=WBP4 PE=1 SV=1 | WBP4 | _K[Lac (K)]FC[Carbamidomethyl (C)]DYC[Carbamidomethyl (C)]K_ |
| O75554 | 256 | K | 0.205194035 | Down | WW domain-binding protein 4 OS=Homo sapiens OX=9606 GN=WBP4 PE=1 SV=1 | WBP4 | _NK[Lac (K)]NSDGGSDPETQK_ |
| O75554 | 274 | K | 0.343019918 | Down | WW domain-binding protein 4 OS=Homo sapiens OX=9606 GN=WBP4 PE=1 SV=1 | WBP4 | _SIQK[Lac (K)]QNSLGSNEEK_ |
| O75554 | 290 | K | 0.2040505 | Down | WW domain-binding protein 4 OS=Homo sapiens OX=9606 GN=WBP4 PE=1 SV=1 | WBP4 | _K[Lac (K)]SNPYGEWQEIK_ |
| O75554 | 357 | K | 0.118413384 | Down | WW domain-binding protein 4 OS=Homo sapiens OX=9606 GN=WBP4 PE=1 SV=1 | WBP4 | _TVTSLGVM[Oxidation (M)]ADGVAPVFK[Lac (K)]K_ |
| O75569 | 25 | K | 0.186942459 | Down | Interferon-inducible double-stranded RNA-dependent protein kinase activator A OS=Homo sapiens OX=9606 GN=PRKRA PE=1 SV=1 | PRKRA | _EDSGTFSLGK[Lac (K)]MITAK_ |
| O75575 | 34 | K | 0.494815171 | Down | DNA-directed RNA polymerase III subunit RPC9 OS=Homo sapiens OX=9606 GN=CRCP PE=1 SV=1 | CRCP | _NK[Lac (K)]HSSGQQNLNTITYETLK_ |
| O75582 | 737 | K | 0.565903719 | Down | Ribosomal protein S6 kinase alpha-5 OS=Homo sapiens OX=9606 GN=RPS6KA5 PE=1 SV=1 | RPS6KA5 | _EGFC[Carbamidomethyl (C)]LQNVDK[Lac (K)]APLAK_ |
| O75643 | 1404 | K | 0.15000293 | Down | U5 small nuclear ribonucleoprotein 200 kDa helicase OS=Homo sapiens OX=9606 GN=SNRNP200 PE=1 SV=2 | SNRNP200 | _K[Lac (K)]VVLLTGETSTDLK_ |
| O75683 | 73 | K | 0.532176086 | Down | Surfeit locus protein 6 OS=Homo sapiens OX=9606 GN=SURF6 PE=1 SV=3 | SURF6 | _SLGEK[Lac (K)]SPAASGAR_ |
| O75683 | 213 | K | 0.577695994 | Down | Surfeit locus protein 6 OS=Homo sapiens OX=9606 GN=SURF6 PE=1 SV=3 | SURF6 | _VEVSEDEPASK[Lac (K)]AQR_ |
| O75683 | 225 | K | 0.517908532 | Down | Surfeit locus protein 6 OS=Homo sapiens OX=9606 GN=SURF6 PE=1 SV=3 | SURF6 | _VK[Lac (K)]GNLTPLTGR_ |
| O75683 | 269 | K | 0.388753696 | Down | Surfeit locus protein 6 OS=Homo sapiens OX=9606 GN=SURF6 PE=1 SV=3 | SURF6 | _MK[Lac (K)]WTNLLYK_ |
| O75683 | 276 | K | 0.296075588 | Down | Surfeit locus protein 6 OS=Homo sapiens OX=9606 GN=SURF6 PE=1 SV=3 | SURF6 | _WTNLLYK[Lac (K)]AEGVK_ |
| O75691 | 2510 | K | 0.645915171 | Down | Small subunit processome component 20 homolog OS=Homo sapiens OX=9606 GN=UTP20 PE=1 SV=3 | UTP20 | _K[Lac (K)]HLPEPVAIK_ |
| O75691 | 2731 | K | 0.498336003 | Down | Small subunit processome component 20 homolog OS=Homo sapiens OX=9606 GN=UTP20 PE=1 SV=3 | UTP20 | _K[Lac (K)]ALEFVTNPDIAAK_ |
| O75691 | 2744 | K | 0.518976411 | Down | Small subunit processome component 20 homolog OS=Homo sapiens OX=9606 GN=UTP20 PE=1 SV=3 | UTP20 | _ALEFVTNPDIAAK[Lac (K)]K_ |
| O75691 | 2760 | K | 0.423842166 | Down | Small subunit processome component 20 homolog OS=Homo sapiens OX=9606 GN=UTP20 PE=1 SV=3 | UTP20 | _K[Lac (K)]IEFLRPGYK_ |
| O75717 | 805 | K | 0.366728903 | Down | WD repeat and HMG-box DNA-binding protein 1 OS=Homo sapiens OX=9606 GN=WDHD1 PE=1 SV=1 | WDHD1 | _K[Lac (K)]LILAQK_ |
| O75717 | 840 | K | 0.375336466 | Down | WD repeat and HMG-box DNA-binding protein 1 OS=Homo sapiens OX=9606 GN=WDHD1 PE=1 SV=1 | WDHD1 | _K[Lac (K)]LNAGYSNTATEWSQPR_ |
| O75717 | 941 | K | 0.249054759 | Down | WD repeat and HMG-box DNA-binding protein 1 OS=Homo sapiens OX=9606 GN=WDHD1 PE=1 SV=1 | WDHD1 | _STNILDNMGK[Lac (K)]SSK_ |
| O75717 | 962 | K | 0.337092565 | Down | WD repeat and HMG-box DNA-binding protein 1 OS=Homo sapiens OX=9606 GN=WDHD1 PE=1 SV=1 | WDHD1 | _SPIIK[Lac (K)]PLIPK_ |
| O75717 | 971 | K | 0.132815952 | Down | WD repeat and HMG-box DNA-binding protein 1 OS=Homo sapiens OX=9606 GN=WDHD1 PE=1 SV=1 | WDHD1 | _PKPK[Lac (K)]QASAASYFQK_ |
| O75717 | 1070 | K | 0.576123662 | Down | WD repeat and HMG-box DNA-binding protein 1 OS=Homo sapiens OX=9606 GN=WDHD1 PE=1 SV=1 | WDHD1 | _AK[Lac (K)]GETASEGTEAK_ |
| O75717 | 1081 | K | 0.612840905 | Down | WD repeat and HMG-box DNA-binding protein 1 OS=Homo sapiens OX=9606 GN=WDHD1 PE=1 SV=1 | WDHD1 | _GETASEGTEAK[Lac (K)]K_ |
| O75717 | 1108 | K | 0.465361156 | Down | WD repeat and HMG-box DNA-binding protein 1 OS=Homo sapiens OX=9606 GN=WDHD1 PE=1 SV=1 | WDHD1 | _ENLNLSK[Lac (K)]K_ |
| O75794 | 308 | K | 0.384348041 | Down | Cell division cycle protein 123 homolog OS=Homo sapiens OX=9606 GN=CDC123 PE=1 SV=1 | CDC123 | _LPK[Lac (K)]DFVDLSTGEDAHK_ |
| O75915 | 151 | K | 0.649427391 | Down | PRA1 family protein 3 OS=Homo sapiens OX=9606 GN=ARL6IP5 PE=1 SV=1 | ARL6IP5 | _LENK[Lac (K)]MEGIGLK_ |
| O75928 | 489 | K | 0.406312248 | Down | E3 SUMO-protein ligase PIAS2 OS=Homo sapiens OX=9606 GN=PIAS2 PE=1 SV=3 | PIAS2 | _K[Lac (K)]C[Carbamidomethyl (C)]IFMSETQSSPTK_ |
| O75937 | 210 | K | 0.485797734 | Down | DnaJ homolog subfamily C member 8 OS=Homo sapiens OX=9606 GN=DNAJC8 PE=1 SV=2 | DNAJC8 | _EWQK[Lac (K)]NFEESR_ |
| O75937 | 246 | K | 0.645293383 | Down | DnaJ homolog subfamily C member 8 OS=Homo sapiens OX=9606 GN=DNAJC8 PE=1 SV=2 | DNAJC8 | _TFLRPPK[Lac (K)]VK_ |
| O75940 | 177 | K | 0.234575021 | Down | Survival of motor neuron-related-splicing factor 30 OS=Homo sapiens OX=9606 GN=SMNDC1 PE=1 SV=1 | SMNDC1 | _VK[Lac (K)]WQQFNNR_ |
| O75940 | 229 | K | 0.505102562 | Down | Survival of motor neuron-related-splicing factor 30 OS=Homo sapiens OX=9606 GN=SMNDC1 PE=1 SV=1 | SMNDC1 | _PMTQYQDTSK[Lac (K)]YNVR_ |
| O75943 | 361 | K | 0.145331859 | Down | Cell cycle checkpoint protein RAD17 OS=Homo sapiens OX=9606 GN=RAD17 PE=1 SV=2 | RAD17 | _GMSLK[Lac (K)]SDAVLSK_ |
| O75943 | 368 | K | 0.320711211 | Down | Cell cycle checkpoint protein RAD17 OS=Homo sapiens OX=9606 GN=RAD17 PE=1 SV=2 | RAD17 | _SDAVLSK[Lac (K)]SK_ |
| O76021 | 109 | K | 2.448870464 | Up | Ribosomal L1 domain-containing protein 1 OS=Homo sapiens OX=9606 GN=RSL1D1 PE=1 SV=3 | RSL1D1 | _DEPNSTPEK[Lac (K)]TEQFYR_ |
| O76021 | 426 | K | 2.365166932 | Up | Ribosomal L1 domain-containing protein 1 OS=Homo sapiens OX=9606 GN=RSL1D1 PE=1 SV=3 | RSL1D1 | _AAESETPGK[Lac (K)]SPEK_ |
| O76021 | 461 | K | 0.264864514 | Down | Ribosomal L1 domain-containing protein 1 OS=Homo sapiens OX=9606 GN=RSL1D1 PE=1 SV=3 | RSL1D1 | _KPEAK[Lac (K)]FFTTPSK_ |
| O76041 | 811 | K | 0.325075801 | Down | Nebulette OS=Homo sapiens OX=9606 GN=NEBL PE=1 SV=1 | NEBL | _K[Lac (K)]NTQVVSDAAYK_ |
| O76094 | 569 | K | 0.595051864 | Down | Signal recognition particle subunit SRP72 OS=Homo sapiens OX=9606 GN=SRP72 PE=1 SV=3 | SRP72 | _NYDPK[Lac (K)]VTPDPER_ |
| O94761 | 114 | K | 0.197294719 | Down | ATP-dependent DNA helicase Q4 OS=Homo sapiens OX=9606 GN=RECQL4 PE=1 SV=2 | RECQL4 | _ANLK[Lac (K)]GTLQAGPALGR_ |
| O94762 | 743 | K | 0.295603454 | Down | ATP-dependent DNA helicase Q5 OS=Homo sapiens OX=9606 GN=RECQL5 PE=1 SV=2 | RECQL5 | _SSSGGSSLAK[Lac (K)]GR_ |
| O94762 | 848 | K | 0.443961656 | Down | ATP-dependent DNA helicase Q5 OS=Homo sapiens OX=9606 GN=RECQL5 PE=1 SV=2 | RECQL5 | _DQGTPEVQPTPAK[Lac (K)]DTWK_ |
| O94762 | 869 | K | 0.443201593 | Down | ATP-dependent DNA helicase Q5 OS=Homo sapiens OX=9606 GN=RECQL5 PE=1 SV=2 | RECQL5 | _SQQENPESQPQK[Lac (K)]R_ |
| O94776 | 460 | K | 0.361968877 | Down | Metastasis-associated protein MTA2 OS=Homo sapiens OX=9606 GN=MTA2 PE=1 SV=1 | MTA2 | _QTFLLQTTK[Lac (K)]LTR_ |
| O94776 | 531 | K | 0.124650733 | Down | Metastasis-associated protein MTA2 OS=Homo sapiens OX=9606 GN=MTA2 PE=1 SV=1 | MTA2 | _DLVAQAPLK[Lac (K)]PK_ |
| O94776 | 559 | K | 0.390036593 | Down | Metastasis-associated protein MTA2 OS=Homo sapiens OX=9606 GN=MTA2 PE=1 SV=1 | MTA2 | _GLGGIMVK[Lac (K)]R_ |
| O94776 | 595 | K | 0.262016441 | Down | Metastasis-associated protein MTA2 OS=Homo sapiens OX=9606 GN=MTA2 PE=1 SV=1 | MTA2 | _QK[Lac (K)]LNPADAPNPVVFVATK_ |
| O94804 | 955 | K | 0.364904908 | Down | Serine/threonine-protein kinase 10 OS=Homo sapiens OX=9606 GN=STK10 PE=1 SV=1 | STK10 | _LSEEAEC[Carbamidomethyl (C)]PNPSTPSK[Lac (K)]AAK_ |
| O94811 | 156 | K | 0.381098909 | Down | Tubulin polymerization-promoting protein OS=Homo sapiens OX=9606 GN=TPPP PE=1 SV=1 | TPPP | _APIISGVTK[Lac (K)]AISSPTVSR_ |
| O94842 | 207 | K | 0.549238335 | Down | TOX high mobility group box family member 4 OS=Homo sapiens OX=9606 GN=TOX4 PE=1 SV=1 | TOX4 | _TVVVEAGK[Lac (K)]K_ |
| O94842 | 285 | K | 0.500910256 | Down | TOX high mobility group box family member 4 OS=Homo sapiens OX=9606 GN=TOX4 PE=1 SV=1 | TOX4 | _EYLK[Lac (K)]ALAAYK_ |
| O94874 | 289 | K | 0.427517566 | Down | E3 UFM1-protein ligase 1 OS=Homo sapiens OX=9606 GN=UFL1 PE=1 SV=2 | UFL1 | _YK[Lac (K)]TTQLLFLK_ |
| O94901 | 195 | K | 0.475376581 | Down | SUN domain-containing protein 1 OS=Homo sapiens OX=9606 GN=SUN1 PE=1 SV=4 | SUN1 | _K[Lac (K)]DVLTAHPAAPGPVSR_ |
| O94906 | 61 | K | 0.506515302 | Down | Pre-mRNA-processing factor 6 OS=Homo sapiens OX=9606 GN=PRPF6 PE=1 SV=1 | PRPF6 | _TVGDQMK[Lac (K)]K_ |
| O94906 | 130 | K | 0.478456832 | Down | Pre-mRNA-processing factor 6 OS=Homo sapiens OX=9606 GN=PRPF6 PE=1 SV=1 | PRPF6 | _EKEEIEK[Lac (K)]YR_ |
| O94913 | 331 | K | 0.416896958 | Down | Pre-mRNA cleavage complex 2 protein Pcf11 OS=Homo sapiens OX=9606 GN=PCF11 PE=1 SV=3 | PCF11 | _TSK[Lac (K)]TIPSEK_ |
| O94913 | 382 | K | 0.551848118 | Down | Pre-mRNA cleavage complex 2 protein Pcf11 OS=Homo sapiens OX=9606 GN=PCF11 PE=1 SV=3 | PCF11 | _LSHTK[Lac (K)]DLK_ |
| O94913 | 448 | K | 0.644784977 | Down | Pre-mRNA cleavage complex 2 protein Pcf11 OS=Homo sapiens OX=9606 GN=PCF11 PE=1 SV=3 | PCF11 | _NK[Lac (K)]IINGIVQK_ |
| O94913 | 592 | K | 0.40491501 | Down | Pre-mRNA cleavage complex 2 protein Pcf11 OS=Homo sapiens OX=9606 GN=PCF11 PE=1 SV=3 | PCF11 | _ENVENWQSSK[Lac (K)]SAK_ |
| O94913 | 598 | K | 0.513484219 | Down | Pre-mRNA cleavage complex 2 protein Pcf11 OS=Homo sapiens OX=9606 GN=PCF11 PE=1 SV=3 | PCF11 | _WK[Lac (K)]SGWEENK_ |
| O94913 | 775 | K | 0.344035149 | Down | Pre-mRNA cleavage complex 2 protein Pcf11 OS=Homo sapiens OX=9606 GN=PCF11 PE=1 SV=3 | PCF11 | _MIFEGPNK[Lac (K)]LSPR_ |
| O94913 | 1278 | K | 0.385103923 | Down | Pre-mRNA cleavage complex 2 protein Pcf11 OS=Homo sapiens OX=9606 GN=PCF11 PE=1 SV=3 | PCF11 | _LLK[Lac (K)]TGILK_ |
| O94913 | 1419 | K | 0.60381536 | Down | Pre-mRNA cleavage complex 2 protein Pcf11 OS=Homo sapiens OX=9606 GN=PCF11 PE=1 SV=3 | PCF11 | _VHEEVVLK[Lac (K)]TQEAAK_ |
| O95149 | 34 | K | 0.522387609 | Down | Snurportin-1 OS=Homo sapiens OX=9606 GN=SNUPN PE=1 SV=1 | SNUPN | _SK[Lac (K)]YSSLEQSER_ |
| O95208 | 423 | K | 0.594755853 | Down | Epsin-2 OS=Homo sapiens OX=9606 GN=EPN2 PE=1 SV=3 | EPN2 | _NSDPWAASQQPASSAGK[Lac (K)]R_ |
| O95218 | 54 | K | 0.603507075 | Down | Zinc finger Ran-binding domain-containing protein 2 OS=Homo sapiens OX=9606 GN=ZRANB2 PE=1 SV=2 | ZRANB2 | _AGGTEIGK[Lac (K)]TLAEK_ |
| O95218 | 137 | K | 0.419759856 | Down | Zinc finger Ran-binding domain-containing protein 2 OS=Homo sapiens OX=9606 GN=ZRANB2 PE=1 SV=2 | ZRANB2 | _GK[Lac (K)]AVGPASILK_ |
| O95218 | 195 | K | 0.489974405 | Down | Zinc finger Ran-binding domain-containing protein 2 OS=Homo sapiens OX=9606 GN=ZRANB2 PE=1 SV=2 | ZRANB2 | _YNLDASEEEDSNK[Lac (K)]K_ |
| O95235 | 37 | K | 0.208679352 | Down | Kinesin-like protein KIF20A OS=Homo sapiens OX=9606 GN=KIF20A PE=1 SV=1 | KIF20A | _K[Lac (K)]NLLSDC[Carbamidomethyl (C)]SVVSTSLEDK_ |
| O95235 | 453 | K | 0.310073602 | Down | Kinesin-like protein KIF20A OS=Homo sapiens OX=9606 GN=KIF20A PE=1 SV=1 | KIF20A | _SK[Lac (K)]QNLVPFR_ |
| O95235 | 537 | K | 0.437774921 | Down | Kinesin-like protein KIF20A OS=Homo sapiens OX=9606 GN=KIF20A PE=1 SV=1 | KIF20A | _EHSLQVSPSLEK[Lac (K)]GAK_ |
| O95239 | 688 | K | 0.27325443 | Down | Chromosome-associated kinesin KIF4A OS=Homo sapiens OX=9606 GN=KIF4A PE=1 SV=3 | KIF4A | _QYELLK[Lac (K)]LER_ |
| O95239 | 794 | K | 0.521300312 | Down | Chromosome-associated kinesin KIF4A OS=Homo sapiens OX=9606 GN=KIF4A PE=1 SV=3 | KIF4A | _ESGENPPPK[Lac (K)]LR_ |
| O95239 | 1033 | K | 0.285029596 | Down | Chromosome-associated kinesin KIF4A OS=Homo sapiens OX=9606 GN=KIF4A PE=1 SV=3 | KIF4A | _EK[Lac (K)]FLEQSMDIEDLK_ |
| O95239 | 1179 | K | 0.233598744 | Down | Chromosome-associated kinesin KIF4A OS=Homo sapiens OX=9606 GN=KIF4A PE=1 SV=3 | KIF4A | _EMC[Carbamidomethyl (C)]DVEQVLSK[Lac (K)]K_ |
| O95243 | 239 | K | 0.150755664 | Down | Methyl-CpG-binding domain protein 4 OS=Homo sapiens OX=9606 GN=MBD4 PE=1 SV=1 | MBD4 | _VTILK[Lac (K)]GIPIK_ |
| O95251 | 90 | K | 0.421555363 | Down | Histone acetyltransferase KAT7 OS=Homo sapiens OX=9606 GN=KAT7 PE=1 SV=1 | KAT7 | _SQQQPTPVTPK[Lac (K)]K_ |
| O95292 | 155 | K | 0.37234897 | Down | Vesicle-associated membrane protein-associated protein B/C OS=Homo sapiens OX=9606 GN=VAPB PE=1 SV=3 | VAPB | _TETPIVSK[Lac (K)]SLSSSLDDTEVKK_ |
| O95292 | 200 | K | 0.229215767 | Down | Vesicle-associated membrane protein-associated protein B/C OS=Homo sapiens OX=9606 GN=VAPB PE=1 SV=3 | VAPB | _K[Lac (K)]TVQSNSPISALAPTGK_ |
| O95347 | 749 | K | 8.66004155 | Up | Structural maintenance of chromosomes protein 2 OS=Homo sapiens OX=9606 GN=SMC2 PE=1 SV=2 | SMC2 | _QQEELDALK[Lac (K)]K_ |
| O95352 | 309 | K | 2.417456621 | Up | Ubiquitin-like modifier-activating enzyme ATG7 OS=Homo sapiens OX=9606 GN=ATG7 PE=1 SV=1 | ATG7 | _NQK[Lac (K)]GGMGPR_ |
| O95365 | 383 | K | 1.537243879 | Up | Zinc finger and BTB domain-containing protein 7A OS=Homo sapiens OX=9606 GN=ZBTB7A PE=1 SV=1 | ZBTB7A | _AFQK[Lac (K)]C[Carbamidomethyl (C)]PIC[Carbamidomethyl (C)]EK_ |
| O95365 | 396 | K | 2.119934766 | Up | Zinc finger and BTB domain-containing protein 7A OS=Homo sapiens OX=9606 GN=ZBTB7A PE=1 SV=1 | ZBTB7A | _VIQGAGK[Lac (K)]LPR_ |
| O95365 | 487 | K | 1.53915396 | Up | Zinc finger and BTB domain-containing protein 7A OS=Homo sapiens OX=9606 GN=ZBTB7A PE=1 SV=1 | ZBTB7A | _K[Lac (K)]DGC[Carbamidomethyl (C)]NGVPSR_ |
| O95391 | 114 | K | 0.390280357 | Down | Pre-mRNA-splicing factor SLU7 OS=Homo sapiens OX=9606 GN=SLU7 PE=1 SV=2 | SLU7 | _ENSIITK[Lac (K)]YR_ |
| O95391 | 199 | K | 0.341159266 | Down | Pre-mRNA-splicing factor SLU7 OS=Homo sapiens OX=9606 GN=SLU7 PE=1 SV=2 | SLU7 | _AQK[Lac (K)]LQEELASGK_ |
| O95391 | 355 | K | 0.297660008 | Down | Pre-mRNA-splicing factor SLU7 OS=Homo sapiens OX=9606 GN=SLU7 PE=1 SV=2 | SLU7 | _LELLYK[Lac (K)]SFK_ |
| O95391 | 527 | K | 0.380204812 | Down | Pre-mRNA-splicing factor SLU7 OS=Homo sapiens OX=9606 GN=SLU7 PE=1 SV=2 | SLU7 | _K[Lac (K)]ALNAEEAR_ |
| O95400 | 26 | K | 0.429349484 | Down | CD2 antigen cytoplasmic tail-binding protein 2 OS=Homo sapiens OX=9606 GN=CD2BP2 PE=1 SV=1 | CD2BP2 | _K[Lac (K)]LVDPVAGSGGPGSR_ |
| O95400 | 321 | K | 0.541701958 | Down | CD2 antigen cytoplasmic tail-binding protein 2 OS=Homo sapiens OX=9606 GN=CD2BP2 PE=1 SV=1 | CD2BP2 | _K[Lac (K)]LDPPGGQFYNSK_ |
| O95433 | 3 | K | 0.638896091 | Down | Activator of 90 kDa heat shock protein ATPase homolog 1 OS=Homo sapiens OX=9606 GN=AHSA1 PE=1 SV=1 | AHSA1 | _[Acetyl (Protein N-term)]AK[Lac (K)]WGEGDPR_ |
| O95453 | 525 | K | 0.53222275 | Down | Poly(A)-specific ribonuclease PARN OS=Homo sapiens OX=9606 GN=PARN PE=1 SV=1 | PARN | _K[Lac (K)]WTEDSWK_ |
| O95453 | 566 | K | 0.419756246 | Down | Poly(A)-specific ribonuclease PARN OS=Homo sapiens OX=9606 GN=PARN PE=1 SV=1 | PARN | _NNSFTAPSTVGK[Lac (K)]R_ |
| O95453 | 616 | K | 0.221371449 | Down | Poly(A)-specific ribonuclease PARN OS=Homo sapiens OX=9606 GN=PARN PE=1 SV=1 | PARN | _K[Lac (K)]ELSPAGSISK_ |
| O95456 | 55 | K | 0.13025451 | Down | Proteasome assembly chaperone 1 OS=Homo sapiens OX=9606 GN=PSMG1 PE=1 SV=1 | PSMG1 | _QTK[Lac (K)]TSLEVSLLEK_ |
| O95478 | 116 | K | 0.309903519 | Down | Ribosome biogenesis protein NSA2 homolog OS=Homo sapiens OX=9606 GN=NSA2 PE=1 SV=1 | NSA2 | _AGK[Lac (K)]WEVPLPK_ |
| O95568 | 282 | K | 0.27460658 | Down | Histidine protein methyltransferase 1 homolog OS=Homo sapiens OX=9606 GN=METTL18 PE=1 SV=1 | METTL18 | _LVLSSEK[Lac (K)]LFVK_ |
| O95602 | 1360 | K | 0.565185607 | Down | DNA-directed RNA polymerase I subunit RPA1 OS=Homo sapiens OX=9606 GN=POLR1A PE=1 SV=2 | POLR1A | _NNK[Lac (K)]ASAFR_ |
| O95625 | 321 | K | 0.251695787 | Down | Zinc finger and BTB domain-containing protein 11 OS=Homo sapiens OX=9606 GN=ZBTB11 PE=1 SV=2 | ZBTB11 | _K[Lac (K)]GEVQTVASTQDLR_ |
| O95644 | 500 | K | 0.492484599 | Down | Nuclear factor of activated T-cells, cytoplasmic 1 OS=Homo sapiens OX=9606 GN=NFATC1 PE=1 SV=3 | NFATC1 | _ITGK[Lac (K)]TVSTTSHEAILSNTK_ |
| O95696 | 100 | K | 0.581177096 | Down | Bromodomain-containing protein 1 OS=Homo sapiens OX=9606 GN=BRD1 PE=1 SV=1 | BRD1 | _K[Lac (K)]NEALPSAHGTPASASALPEPK_ |
| O95721 | 169 | K | 0.330922468 | Down | Synaptosomal-associated protein 29 OS=Homo sapiens OX=9606 GN=SNAP29 PE=1 SV=1 | SNAP29 | _K[Lac (K)]LDDTDPVPR_ |
| O95721 | 201 | K | 0.229933269 | Down | Synaptosomal-associated protein 29 OS=Homo sapiens OX=9606 GN=SNAP29 PE=1 SV=1 | SNAP29 | _AYHQK[Lac (K)]IDSNLDELSMGLGR_ |
| O95785 | 933 | K | 0.610924642 | Down | Protein Wiz OS=Homo sapiens OX=9606 GN=WIZ PE=1 SV=2 | WIZ | _GLPDAHLGLPPGLAK[Lac (K)]K_ |
| O95785 | 967 | K | 0.566430069 | Down | Protein Wiz OS=Homo sapiens OX=9606 GN=WIZ PE=1 SV=2 | WIZ | _AIK[Lac (K)]SPPGFSAK_ |
| O95785 | 987 | K | 0.532403027 | Down | Protein Wiz OS=Homo sapiens OX=9606 GN=WIZ PE=1 SV=2 | WIZ | _GLGHPPSSPLLK[Lac (K)]K_ |
| O95785 | 988 | K | 0.323277393 | Down | Protein Wiz OS=Homo sapiens OX=9606 GN=WIZ PE=1 SV=2 | WIZ | _K[Lac (K)]TPLALAGSPTPK_ |
| O95785 | 1108 | K | 0.501454721 | Down | Protein Wiz OS=Homo sapiens OX=9606 GN=WIZ PE=1 SV=2 | WIZ | _PGGPPNPPGPSPK[Lac (K)]ALAK_ |
| O95785 | 1112 | K | 0.3227034 | Down | Protein Wiz OS=Homo sapiens OX=9606 GN=WIZ PE=1 SV=2 | WIZ | _ALAK[Lac (K)]MMGGAGPGSSLEAR_ |
| O95785 | 1356 | K | 0.363655786 | Down | Protein Wiz OS=Homo sapiens OX=9606 GN=WIZ PE=1 SV=2 | WIZ | _PSATGYLGSVAAK[Lac (K)]R_ |
| O95785 | 1370 | K | 0.407552624 | Down | Protein Wiz OS=Homo sapiens OX=9606 GN=WIZ PE=1 SV=2 | WIZ | _LLPAEVK[Lac (K)]AK_ |
| O95785 | 1382 | K | 0.425267169 | Down | Protein Wiz OS=Homo sapiens OX=9606 GN=WIZ PE=1 SV=2 | WIZ | _TYIQTELPFK[Lac (K)]AK_ |
| O95785 | 1448 | K | 0.575970838 | Down | Protein Wiz OS=Homo sapiens OX=9606 GN=WIZ PE=1 SV=2 | WIZ | _HRPQK[Lac (K)]VGAYR_ |
| O95785 | 1477 | K | 0.262759974 | Down | Protein Wiz OS=Homo sapiens OX=9606 GN=WIZ PE=1 SV=2 | WIZ | _DSDK[Lac (K)]RPSLGLAPGGLAVVGR_ |
| O95785 | 1534 | K | 0.629402691 | Down | Protein Wiz OS=Homo sapiens OX=9606 GN=WIZ PE=1 SV=2 | WIZ | _QNINK[Lac (K)]FER_ |
| O95793 | 382 | K | 0.232884673 | Down | Double-stranded RNA-binding protein Staufen homolog 1 OS=Homo sapiens OX=9606 GN=STAU1 PE=1 SV=2 | STAU1 | _K[Lac (K)]VTFFEPGSGDENGTSNK_ |
| O95816 | 9 | K | 1.681298519 | Up | BAG family molecular chaperone regulator 2 OS=Homo sapiens OX=9606 GN=BAG2 PE=1 SV=1 | BAG2 | _INAK[Lac (K)]ANEGR_ |
| O95819 | 670 | K | 0.61240103 | Down | Mitogen-activated protein kinase kinase kinase kinase 4 OS=Homo sapiens OX=9606 GN=MAP4K4 PE=1 SV=2 | MAP4K4 | _VEK[Lac (K)]LVPR_ |
| O95983 | 41 | K | 0.522601231 | Down | Methyl-CpG-binding domain protein 3 OS=Homo sapiens OX=9606 GN=MBD3 PE=1 SV=1 | MBD3 | _DVFYYSPSGK[Lac (K)]K_ |
| O95983 | 46 | K | 0.65496675 | Down | Methyl-CpG-binding domain protein 3 OS=Homo sapiens OX=9606 GN=MBD3 PE=1 SV=1 | MBD3 | _SK[Lac (K)]PQLAR_ |
| O95983 | 109 | K | 0.611240695 | Down | Methyl-CpG-binding domain protein 3 OS=Homo sapiens OX=9606 GN=MBD3 PE=1 SV=1 | MBD3 | _QTASIFK[Lac (K)]QPVTK_ |
| O96028 | 598 | K | 0.481077618 | Down | Histone-lysine N-methyltransferase NSD2 OS=Homo sapiens OX=9606 GN=NSD2 PE=1 SV=1 | NSD2 | _NLSDAC[Carbamidomethyl (C)]KPLK[Lac (K)]K_ |
| P00338 | 232 | K | 0.601523942 | Down | L-lactate dehydrogenase A chain OS=Homo sapiens OX=9606 GN=LDHA PE=1 SV=2 | LDHA | _EVHK[Lac (K)]QVVESAYEVIK_ |
| P00338 | 318 | K | 2.067190777 | Up | L-lactate dehydrogenase A chain OS=Homo sapiens OX=9606 GN=LDHA PE=1 SV=2 | LDHA | _K[Lac (K)]SADTLWGIQK_ |
| P00367 | 480 | K | 2.217205328 | Up | Glutamate dehydrogenase 1, mitochondrial OS=Homo sapiens OX=9606 GN=GLUD1 PE=1 SV=2 | GLUD1 | _FGK[Lac (K)]HGGTIPIVPTAEFQDR_ |
| P00441 | 137 | K | 1.678713722 | Up | Superoxide dismutase [Cu-Zn] OS=Homo sapiens OX=9606 GN=SOD1 PE=1 SV=2 | SOD1 | _GGNEESTK[Lac (K)]TGNAGSR_ |
| P00558 | 131 | K | 1.922991042 | Up | Phosphoglycerate kinase 1 OS=Homo sapiens OX=9606 GN=PGK1 PE=1 SV=3 | PGK1 | _FHVEEEGK[Lac (K)]GK_ |
| P00558 | 323 | K | 1.706194612 | Up | Phosphoglycerate kinase 1 OS=Homo sapiens OX=9606 GN=PGK1 PE=1 SV=3 | PGK1 | _K[Lac (K)]YAEAVTR_ |
| P02545 | 417 | K | 0.56098716 | Down | Prelamin-A/C OS=Homo sapiens OX=9606 GN=LMNA PE=1 SV=1 | LMNA | _ASSHSSQTQGGGSVTK[Lac (K)]K_ |
| P02768 | 499 | K | 1.715915718 | Up | Albumin OS=Homo sapiens OX=9606 GN=ALB PE=1 SV=2 | ALB | _VTK[Lac (K)]C[Carbamidomethyl (C)]C[Carbamidomethyl (C)]TESLVNR_ |
| P02795 | 31 | K | 1.577791439 | Up | Metallothionein-2 OS=Homo sapiens OX=9606 GN=MT2A PE=1 SV=1 | MT2A | _K[Lac (K)]SC[Carbamidomethyl (C)]C[Carbamidomethyl (C)]SC[Carbamidomethyl (C)]C[Carbamidomethyl (C)]PVGC[Carbamidomethyl (C)]AK_ |
| P04040 | 23 | K | 0.434423976 | Down | Catalase OS=Homo sapiens OX=9606 GN=CAT PE=1 SV=3 | CAT | _AAQK[Lac (K)]ADVLTTGAGNPVGDK_ |
| P04075 | 42 | K | 1.52905264 | Up | Fructose-bisphosphate aldolase A OS=Homo sapiens OX=9606 GN=ALDOA PE=1 SV=2 | ALDOA | _GILAADESTGSIAK[Lac (K)]R_ |
| P04075 | 108 | K | 1.524973183 | Up | Fructose-bisphosphate aldolase A OS=Homo sapiens OX=9606 GN=ALDOA PE=1 SV=2 | ALDOA | _GGVVGIK[Lac (K)]VDK_ |
| P04083 | 90 | K | 2.202384139 | Up | Annexin A1 OS=Homo sapiens OX=9606 GN=ANXA1 PE=1 SV=2 | ANXA1 | _AAYLQETGK[Lac (K)]PLDETLK_ |
| P04083 | 166 | K | 1.509068293 | Up | Annexin A1 OS=Homo sapiens OX=9606 GN=ANXA1 PE=1 SV=2 | ANXA1 | _DLAK[Lac (K)]DITSDTSGDFR_ |
| P04083 | 287 | K | 2.218724347 | Up | Annexin A1 OS=Homo sapiens OX=9606 GN=ANXA1 PE=1 SV=2 | ANXA1 | _LHQAMK[Lac (K)]GVGTR_ |
| P04083 | 312 | K | 1.884089342 | Up | Annexin A1 OS=Homo sapiens OX=9606 GN=ANXA1 PE=1 SV=2 | ANXA1 | _SEIDMNDIK[Lac (K)]AFYQK_ |
| P04264 | 246 | K | 1.911641696 | Up | Keratin, type II cytoskeletal 1 OS=Homo sapiens OX=9606 GN=KRT1 PE=1 SV=6 | KRT1 | _VDQLK[Lac (K)]SDQSR_ |
| P04264 | 364 | K | 1.579323833 | Up | Keratin, type II cytoskeletal 1 OS=Homo sapiens OX=9606 GN=KRT1 PE=1 SV=6 | KRT1 | _AQYEDIAQK[Lac (K)]SK_ |
| P04264 | 366 | K | 1.738754304 | Up | Keratin, type II cytoskeletal 1 OS=Homo sapiens OX=9606 GN=KRT1 PE=1 SV=6 | KRT1 | _SK[Lac (K)]AEAESLYQSK_ |
| P04406 | 61 | K | 2.126425594 | Up | Glyceraldehyde-3-phosphate dehydrogenase OS=Homo sapiens OX=9606 GN=GAPDH PE=1 SV=3 | GAPDH | _FHGTVK[Lac (K)]AENGK_ |
| P04406 | 117 | K | 1.529937021 | Up | Glyceraldehyde-3-phosphate dehydrogenase OS=Homo sapiens OX=9606 GN=GAPDH PE=1 SV=3 | GAPDH | _AGAHLQGGAK[Lac (K)]R_ |
| P04406 | 139 | K | 0.341840603 | Down | Glyceraldehyde-3-phosphate dehydrogenase OS=Homo sapiens OX=9606 GN=GAPDH PE=1 SV=3 | GAPDH | _VIISAPSADAPMFVMGVNHEK[Lac (K)]YDNSLK_ |
| P04406 | 219 | K | 1.88474439 | Up | Glyceraldehyde-3-phosphate dehydrogenase OS=Homo sapiens OX=9606 GN=GAPDH PE=1 SV=3 | GAPDH | _AVGK[Lac (K)]VIPELNGK_ |
| P04406 | 263 | K | 1.685012869 | Up | Glyceraldehyde-3-phosphate dehydrogenase OS=Homo sapiens OX=9606 GN=GAPDH PE=1 SV=3 | GAPDH | _VVK[Lac (K)]QASEGPLK_ |
| P04843 | 187 | K | 2.102607611 | Up | Dolichyl-diphosphooligosaccharide--protein glycosyltransferase subunit 1 OS=Homo sapiens OX=9606 GN=RPN1 PE=1 SV=1 | RPN1 | _NVESYTK[Lac (K)]LGNPTR_ |
| P04843 | 538 | K | 1.531473979 | Up | Dolichyl-diphosphooligosaccharide--protein glycosyltransferase subunit 1 OS=Homo sapiens OX=9606 GN=RPN1 PE=1 SV=1 | RPN1 | _LK[Lac (K)]TEGSDLC[Carbamidomethyl (C)]DR_ |
| P05114 | 61 | K | 1.509335736 | Up | Non-histone chromosomal protein HMG-14 OS=Homo sapiens OX=9606 GN=HMGN1 PE=1 SV=3 | HMGN1 | _GK[Lac (K)]QAEVANQETK_ |
| P05198 | 61 | K | 0.530954662 | Down | Eukaryotic translation initiation factor 2 subunit 1 OS=Homo sapiens OX=9606 GN=EIF2S1 PE=1 SV=3 | EIF2S1 | _SINK[Lac (K)]LIR_ |
| P05198 | 190 | K | 0.641605639 | Down | Eukaryotic translation initiation factor 2 subunit 1 OS=Homo sapiens OX=9606 GN=EIF2S1 PE=1 SV=3 | EIF2S1 | _LTPQAVK[Lac (K)]IR_ |
| P05387 | 25 | K | 0.617716887 | Down | Large ribosomal subunit protein P2 OS=Homo sapiens OX=9606 GN=RPLP2 PE=1 SV=1 | RPLP2 | _K[Lac (K)]ILDSVGIEADDDR_ |
| P05556 | 774 | K | 0.272974448 | Down | Integrin beta-1 OS=Homo sapiens OX=9606 GN=ITGB1 PE=1 SV=2 | ITGB1 | _MNAK[Lac (K)]WDTGENPIYK_ |
| P05783 | 187 | K | 3.055913887 | Up | Keratin, type I cytoskeletal 18 OS=Homo sapiens OX=9606 GN=KRT18 PE=1 SV=2 | KRT18 | _K[Lac (K)]VIDDTNITR_ |
| P05783 | 372 | K | 0.476415954 | Down | Keratin, type I cytoskeletal 18 OS=Homo sapiens OX=9606 GN=KRT18 PE=1 SV=2 | KRT18 | _VK[Lac (K)]LEAEIATYR_ |
| P05787 | 8 | K | 0.27523523 | Down | Keratin, type II cytoskeletal 8 OS=Homo sapiens OX=9606 GN=KRT8 PE=1 SV=7 | KRT8 | _VTQK[Lac (K)]SYK_ |
| P05787 | 11 | K | 0.363442212 | Down | Keratin, type II cytoskeletal 8 OS=Homo sapiens OX=9606 GN=KRT8 PE=1 SV=7 | KRT8 | _SYK[Lac (K)]VSTSGPR_ |
| P05787 | 92 | K | 1.888035601 | Up | Keratin, type II cytoskeletal 8 OS=Homo sapiens OX=9606 GN=KRT8 PE=1 SV=7 | KRT8 | _TQEK[Lac (K)]EQIK_ |
| P05787 | 117 | K | 1.51547767 | Up | Keratin, type II cytoskeletal 8 OS=Homo sapiens OX=9606 GN=KRT8 PE=1 SV=7 | KRT8 | _FLEQQNK[Lac (K)]MLETK_ |
| P05787 | 295 | K | 1.801444188 | Up | Keratin, type II cytoskeletal 8 OS=Homo sapiens OX=9606 GN=KRT8 PE=1 SV=7 | KRT8 | _YEELQSLAGK[Lac (K)]HGDDLR_ |
| P05787 | 304 | K | 1.650851855 | Up | Keratin, type II cytoskeletal 8 OS=Homo sapiens OX=9606 GN=KRT8 PE=1 SV=7 | KRT8 | _TK[Lac (K)]TEISEMNR_ |
| P05787 | 393 | K | 1.703810719 | Up | Keratin, type II cytoskeletal 8 OS=Homo sapiens OX=9606 GN=KRT8 PE=1 SV=7 | KRT8 | _K[Lac (K)]LLEGEESR_ |
| P06400 | 791 | K | 0.426569058 | Down | Retinoblastoma-associated protein OS=Homo sapiens OX=9606 GN=RB1 PE=1 SV=2 | RB1 | _SPYK[Lac (K)]FPSSPLR_ |
| P06400 | 810 | K | 0.246047074 | Down | Retinoblastoma-associated protein OS=Homo sapiens OX=9606 GN=RB1 PE=1 SV=2 | RB1 | _IPGGNIYISPLK[Lac (K)]SPYK_ |
| P06400 | 814 | K | 0.152900979 | Down | Retinoblastoma-associated protein OS=Homo sapiens OX=9606 GN=RB1 PE=1 SV=2 | RB1 | _SPYK[Lac (K)]ISEGLPTPTK_ |
| P06400 | 824 | K | 0.585735327 | Down | Retinoblastoma-associated protein OS=Homo sapiens OX=9606 GN=RB1 PE=1 SV=2 | RB1 | _ISEGLPTPTK[Lac (K)]MTPR_ |
| P06400 | 847 | K | 0.315334887 | Down | Retinoblastoma-associated protein OS=Homo sapiens OX=9606 GN=RB1 PE=1 SV=2 | RB1 | _FQK[Lac (K)]INQMVC[Carbamidomethyl (C)]NSDR_ |
| P06400 | 873 | K | 0.628413528 | Down | Retinoblastoma-associated protein OS=Homo sapiens OX=9606 GN=RB1 PE=1 SV=2 | RB1 | _SAEGSNPPKPLK[Lac (K)]K_ |
| P06400 | 896 | K | 0.491918535 | Down | Retinoblastoma-associated protein OS=Homo sapiens OX=9606 GN=RB1 PE=1 SV=2 | RB1 | _HLPGESK[Lac (K)]FQQK_ |
| P06400 | 900 | K | 0.413715694 | Down | Retinoblastoma-associated protein OS=Homo sapiens OX=9606 GN=RB1 PE=1 SV=2 | RB1 | _FQQK[Lac (K)]LAEMTSTR_ |
| P06400 | 915 | K | 0.457008251 | Down | Retinoblastoma-associated protein OS=Homo sapiens OX=9606 GN=RB1 PE=1 SV=2 | RB1 | _QK[Lac (K)]MNDSMDTSNK_ |
| P06454 | 15 | K | 0.440963209 | Down | Prothymosin alpha OS=Homo sapiens OX=9606 GN=PTMA PE=1 SV=2 | PTMA | _[Acetyl (Protein N-term)]SDAAVDTSSEITTK[Lac (K)]DLK_ |
| P06730 | 54 | K | 0.606107862 | Down | Eukaryotic translation initiation factor 4E OS=Homo sapiens OX=9606 GN=EIF4E PE=1 SV=2 | EIF4E | _SK[Lac (K)]TWQANLR_ |
| P06730 | 162 | K | 1.634608736 | Up | Eukaryotic translation initiation factor 4E OS=Homo sapiens OX=9606 GN=EIF4E PE=1 SV=2 | EIF4E | _GDK[Lac (K)]IAIWTTEC[Carbamidomethyl (C)]ENR_ |
| P06730 | 206 | K | 0.56570816 | Down | Eukaryotic translation initiation factor 4E OS=Homo sapiens OX=9606 GN=EIF4E PE=1 SV=2 | EIF4E | _IVIGYQSHADTATK[Lac (K)]SGSTTK_ |
| P06733 | 5 | K | 1.620116363 | Up | Alpha-enolase OS=Homo sapiens OX=9606 GN=ENO1 PE=1 SV=2 | ENO1 | _[Acetyl (Protein N-term)]SILK[Lac (K)]IHAR_ |
| P06733 | 60 | K | 1.909256783 | Up | Alpha-enolase OS=Homo sapiens OX=9606 GN=ENO1 PE=1 SV=2 | ENO1 | _YMGK[Lac (K)]GVSK_ |
| P06733 | 64 | K | 1.903867063 | Up | Alpha-enolase OS=Homo sapiens OX=9606 GN=ENO1 PE=1 SV=2 | ENO1 | _GVSK[Lac (K)]AVEHINK_ |
| P06733 | 81 | K | 1.814943322 | Up | Alpha-enolase OS=Homo sapiens OX=9606 GN=ENO1 PE=1 SV=2 | ENO1 | _K[Lac (K)]LNVTEQEK_ |
| P06733 | 89 | K | 2.105458093 | Up | Alpha-enolase OS=Homo sapiens OX=9606 GN=ENO1 PE=1 SV=2 | ENO1 | _LNVTEQEK[Lac (K)]IDK_ |
| P06733 | 193 | K | 3.827597177 | Up | Alpha-enolase OS=Homo sapiens OX=9606 GN=ENO1 PE=1 SV=2 | ENO1 | _IGAEVYHNLK[Lac (K)]NVIK_ |
| P06733 | 233 | K | 1.792470198 | Up | Alpha-enolase OS=Homo sapiens OX=9606 GN=ENO1 PE=1 SV=2 | ENO1 | _TAIGK[Lac (K)]AGYTDK_ |
| P06733 | 420 | K | 1.579741114 | Up | Alpha-enolase OS=Homo sapiens OX=9606 GN=ENO1 PE=1 SV=2 | ENO1 | _IEEELGSK[Lac (K)]AK_ |
| P06748 | 27 | K | 1.548838681 | Up | Nucleophosmin OS=Homo sapiens OX=9606 GN=NPM1 PE=1 SV=2 | NPM1 | _ADK[Lac (K)]DYHFK_ |
| P06748 | 273 | K | 0.570968093 | Down | Nucleophosmin OS=Homo sapiens OX=9606 GN=NPM1 PE=1 SV=2 | NPM1 | _FINYVK[Lac (K)]NC[Carbamidomethyl (C)]FR_ |
| P07237 | 309 | K | 2.327690052 | Up | Protein disulfide-isomerase OS=Homo sapiens OX=9606 GN=P4HB PE=1 SV=3 | P4HB | _K[Lac (K)]EEC[Carbamidomethyl (C)]PAVR_ |
| P07237 | 328 | K | 2.076265861 | Up | Protein disulfide-isomerase OS=Homo sapiens OX=9606 GN=P4HB PE=1 SV=3 | P4HB | _YK[Lac (K)]PESEELTAER_ |
| P07355 | 49 | K | 7.171472386 | Up | Annexin A2 OS=Homo sapiens OX=9606 GN=ANXA2 PE=1 SV=2 | ANXA2 | _TK[Lac (K)]GVDEVTIVNILTNR_ |
| P07355 | 81 | K | 0.472189179 | Down | Annexin A2 OS=Homo sapiens OX=9606 GN=ANXA2 PE=1 SV=2 | ANXA2 | _K[Lac (K)]ELASALK_ |
| P07355 | 157 | K | 3.204352298 | Up | Annexin A2 OS=Homo sapiens OX=9606 GN=ANXA2 PE=1 SV=2 | ANXA2 | _TDLEK[Lac (K)]DIISDTSGDFR_ |
| P07355 | 227 | K | 1.873125136 | Up | Annexin A2 OS=Homo sapiens OX=9606 GN=ANXA2 PE=1 SV=2 | ANXA2 | _SVPHLQK[Lac (K)]VFDR_ |
| P07384 | 365 | K | 0.570470196 | Down | Calpain-1 catalytic subunit OS=Homo sapiens OX=9606 GN=CAPN1 PE=1 SV=1 | CAPN1 | _K[Lac (K)]WNTTLYEGTWR_ |
| P07814 | 716 | K | 1.933982816 | Up | Bifunctional glutamate/proline--tRNA ligase OS=Homo sapiens OX=9606 GN=EPRS1 PE=1 SV=5 | EPRS1 | _EMPTSGSK[Lac (K)]EK_ |
| P07814 | 991 | K | 2.940378914 | Up | Bifunctional glutamate/proline--tRNA ligase OS=Homo sapiens OX=9606 GN=EPRS1 PE=1 SV=5 | EPRS1 | _KDPSK[Lac (K)]NQGGGLSSSGAGEGQGPK_ |
| P07900 | 84 | K | 2.092286085 | Up | Heat shock protein HSP 90-alpha OS=Homo sapiens OX=9606 GN=HSP90AA1 PE=1 SV=5 | HSP90AA1 | _ELHINLIPNK[Lac (K)]QDR_ |
| P07900 | 567 | K | 2.09614163 | Up | Heat shock protein HSP 90-alpha OS=Homo sapiens OX=9606 GN=HSP90AA1 PE=1 SV=5 | HSP90AA1 | _TK[Lac (K)]FENLC[Carbamidomethyl (C)]K_ |
| P07910 | 42 | K | 2.21074832 | Up | Heterogeneous nuclear ribonucleoproteins C1/C2 OS=Homo sapiens OX=9606 GN=HNRNPC PE=1 SV=4 | HNRNPC | _YGK[Lac (K)]IVGC[Carbamidomethyl (C)]SVHK_ |
| P07910 | 206 | K | 0.497346986 | Down | Heterogeneous nuclear ribonucleoproteins C1/C2 OS=Homo sapiens OX=9606 GN=HNRNPC PE=1 SV=4 | HNRNPC | _QK[Lac (K)]VDSLLENLEK_ |
| P08047 | 685 | K | 0.514200265 | Down | Transcription factor Sp1 OS=Homo sapiens OX=9606 GN=SP1 PE=1 SV=3 | SP1 | _K[Lac (K)]FAC[Carbamidomethyl (C)]PEC[Carbamidomethyl (C)]PK_ |
| P08133 | 446 | K | 3.280536791 | Up | Annexin A6 OS=Homo sapiens OX=9606 GN=ANXA6 PE=1 SV=3 | ANXA6 | _K[Lac (K)]AMEGAGTDEK_ |
| P08237 | 678 | K | 2.143559816 | Up | ATP-dependent 6-phosphofructokinase, muscle type OS=Homo sapiens OX=9606 GN=PFKM PE=1 SV=2 | PFKM | _NFATK[Lac (K)]MGAK_ |
| P08238 | 107 | K | 1.623766 | Up | Heat shock protein HSP 90-beta OS=Homo sapiens OX=9606 GN=HSP90AB1 PE=1 SV=4 | HSP90AB1 | _ADLINNLGTIAK[Lac (K)]SGTK_ |
| P08238 | 186 | K | 8.414671462 | Up | Heat shock protein HSP 90-beta OS=Homo sapiens OX=9606 GN=HSP90AB1 PE=1 SV=4 | HSP90AB1 | _VILHLK[Lac (K)]EDQTEYLEER_ |
| P08238 | 284 | K | 1.976452485 | Up | Heat shock protein HSP 90-beta OS=Homo sapiens OX=9606 GN=HSP90AB1 PE=1 SV=4 | HSP90AB1 | _YIDQEELNK[Lac (K)]TK_ |
| P08238 | 286 | K | 1.71475311 | Up | Heat shock protein HSP 90-beta OS=Homo sapiens OX=9606 GN=HSP90AB1 PE=1 SV=4 | HSP90AB1 | _TK[Lac (K)]PIWTR_ |
| P08238 | 399 | K | 1.887540683 | Up | Heat shock protein HSP 90-beta OS=Homo sapiens OX=9606 GN=HSP90AB1 PE=1 SV=4 | HSP90AB1 | _EMLQQSK[Lac (K)]ILK_ |
| P08238 | 531 | K | 1.740764947 | Up | Heat shock protein HSP 90-beta OS=Homo sapiens OX=9606 GN=HSP90AB1 PE=1 SV=4 | HSP90AB1 | _EFDGK[Lac (K)]SLVSVTK_ |
| P08238 | 559 | K | 1.753110422 | Up | Heat shock protein HSP 90-beta OS=Homo sapiens OX=9606 GN=HSP90AB1 PE=1 SV=4 | HSP90AB1 | _AK[Lac (K)]FENLC[Carbamidomethyl (C)]K_ |
| P08238 | 577 | K | 1.950462948 | Up | Heat shock protein HSP 90-beta OS=Homo sapiens OX=9606 GN=HSP90AB1 PE=1 SV=4 | HSP90AB1 | _VEK[Lac (K)]VTISNR_ |
| P08729 | 96 | K | 2.292023002 | Up | Keratin, type II cytoskeletal 7 OS=Homo sapiens OX=9606 GN=KRT7 PE=1 SV=5 | KRT7 | _VRQEESEQIK[Lac (K)]TLNNK[Lac (K)]FASFIDK_ |
| P08729 | 101 | K | 2.292023002 | Up | Keratin, type II cytoskeletal 7 OS=Homo sapiens OX=9606 GN=KRT7 PE=1 SV=5 | KRT7 | _VRQEESEQIK[Lac (K)]TLNNK[Lac (K)]FASFIDK_ |
| P08729 | 199 | K | 2.246649931 | Up | Keratin, type II cytoskeletal 7 OS=Homo sapiens OX=9606 GN=KRT7 PE=1 SV=5 | KRT7 | _K[Lac (K)]DVDAAYM[Oxidation (M)]SK_ |
| P09012 | 123 | K | 0.534511851 | Down | U1 small nuclear ribonucleoprotein A OS=Homo sapiens OX=9606 GN=SNRPA PE=1 SV=3 | SNRPA | _K[Lac (K)]AVQGGGATPVVGAVQGPVPGMPPM[Oxidation (M)]TQAPR_ |
| P09429 | 12 | K | 0.160265489 | Down | High mobility group protein B1 OS=Homo sapiens OX=9606 GN=HMGB1 PE=1 SV=3 | HMGB1 | _GK[Lac (K)]MSSYAFFVQTC[Carbamidomethyl (C)]R_ |
| P09429 | 43 | K | 1.913706664 | Up | High mobility group protein B1 OS=Homo sapiens OX=9606 GN=HMGB1 PE=1 SV=3 | HMGB1 | _HPDASVNFSEFSK[Lac (K)]K_ |
| P09429 | 90 | K | 1.661225387 | Up | High mobility group protein B1 OS=Homo sapiens OX=9606 GN=HMGB1 PE=1 SV=3 | HMGB1 | _FK[Lac (K)]DPNAPK_ |
| P09601 | 243 | K | 0.588945443 | Down | Heme oxygenase 1 OS=Homo sapiens OX=9606 GN=HMOX1 PE=1 SV=1 | HMOX1 | _ASNK[Lac (K)]VQDSAPVETPR_ |
| P09661 | 179 | K | 0.443123854 | Down | U2 small nuclear ribonucleoprotein A' OS=Homo sapiens OX=9606 GN=SNRPA1 PE=1 SV=2 | SNRPA1 | _SK[Lac (K)]TFNPGAGLPTDK_ |
| P09661 | 193 | K | 0.64789118 | Down | U2 small nuclear ribonucleoprotein A' OS=Homo sapiens OX=9606 GN=SNRPA1 PE=1 SV=2 | SNRPA1 | _K[Lac (K)]GGPSPGDVEAIK_ |
| P09661 | 221 | K | 0.499530165 | Down | U2 small nuclear ribonucleoprotein A' OS=Homo sapiens OX=9606 GN=SNRPA1 PE=1 SV=2 | SNRPA1 | _LK[Lac (K)]GLLQSGQIPGR_ |
| P09874 | 108 | K | 0.663313484 | Down | Poly [ADP-ribose] polymerase 1 OS=Homo sapiens OX=9606 GN=PARP1 PE=1 SV=4 | PARP1 | _AEK[Lac (K)]TLGDFAAEYAK_ |
| P09874 | 126 | K | 2.157467405 | Up | Poly [ADP-ribose] polymerase 1 OS=Homo sapiens OX=9606 GN=PARP1 PE=1 SV=4 | PARP1 | _STC[Carbamidomethyl (C)]K[Lac (K)]GC[Carbamidomethyl (C)]MEK_ |
| P09874 | 512 | K | 0.454501987 | Down | Poly [ADP-ribose] polymerase 1 OS=Homo sapiens OX=9606 GN=PARP1 PE=1 SV=4 | PARP1 | _GQVK[Lac (K)]EEGINK_ |
| P09874 | 528 | K | 0.293857165 | Down | Poly [ADP-ribose] polymerase 1 OS=Homo sapiens OX=9606 GN=PARP1 PE=1 SV=4 | PARP1 | _LTLK[Lac (K)]GGAAVDPDSGLEHSAHVLEK_ |
| P09884 | 149 | K | 0.602330821 | Down | DNA polymerase alpha catalytic subunit OS=Homo sapiens OX=9606 GN=POLA1 PE=1 SV=2 | POLA1 | _TADK[Lac (K)]AVDLSK_ |
| P0C0S8 | 37 | K | 2.445008603 | Up | Histone H2A type 1 OS=Homo sapiens OX=9606 GN=H2AC11 PE=1 SV=2 | H2AC11 | _K[Lac (K)]GNYAER_ |
| P0C0S8 | 96 | K | 2.005102993 | Up | Histone H2A type 1 OS=Homo sapiens OX=9606 GN=H2AC11 PE=1 SV=2 | H2AC11 | _NDEELNK[Lac (K)]LLGK_ |
| P0DMV8 | 246 | K | 2.332741914 | Up | Heat shock 70 kDa protein 1A OS=Homo sapiens OX=9606 GN=HSPA1A PE=1 SV=1 | HSPA1A | _LVNHFVEEFK[Lac (K)]R_ |
| P0DMV8 | 328 | K | 2.078191185 | Up | Heat shock 70 kDa protein 1A OS=Homo sapiens OX=9606 GN=HSPA1A PE=1 SV=1 | HSPA1A | _LDK[Lac (K)]AQIHDLVLVGGSTR_ |
| P0DP91 | 181 | K | 0.446241463 | Down | Chimeric ERCC6-PGBD3 protein OS=Homo sapiens OX=9606 GN=ERCC6 PE=1 SV=1 | ERCC6 | _YNK[Lac (K)]EQQLK_ |
| P10074 | 194 | K | 0.116714171 | Down | Telomere zinc finger-associated protein OS=Homo sapiens OX=9606 GN=ZBTB48 PE=1 SV=2 | ZBTB48 | _LK[Lac (K)]QALKPC[Carbamidomethyl (C)]PLEDK_ |
| P10074 | 560 | K | 0.247823055 | Down | Telomere zinc finger-associated protein OS=Homo sapiens OX=9606 GN=ZBTB48 PE=1 SV=2 | ZBTB48 | _TFK[Lac (K)]AVEQLR_ |
| P10412 | 34 | K | 0.548253987 | Down | Histone H1.4 OS=Homo sapiens OX=9606 GN=H1-4 PE=1 SV=2 | H1-4 | _K[Lac (K)]ASGPPVSELITK_ |
| P10412 | 90 | K | 0.457633996 | Down | Histone H1.4 OS=Homo sapiens OX=9606 GN=H1-4 PE=1 SV=2 | H1-4 | _SLVSK[Lac (K)]GTLVQTK_ |
| P10599 | 94 | K | 1.537871791 | Up | Thioredoxin OS=Homo sapiens OX=9606 GN=TXN PE=1 SV=3 | TXN | _VGEFSGANK[Lac (K)]EK_ |
| P10636 | 571 | K | 0.574563646 | Down | Microtubule-associated protein tau OS=Homo sapiens OX=9606 GN=MAPT PE=1 SV=5 | MAPT | _LQTAPVPMPDLK[Lac (K)]NVK_ |
| P10636 | 584 | K | 0.59478937 | Down | Microtubule-associated protein tau OS=Homo sapiens OX=9606 GN=MAPT PE=1 SV=5 | MAPT | _IGSTENLK[Lac (K)]HQPGGGK_ |
| P10636 | 670 | K | 0.577635381 | Down | Microtubule-associated protein tau OS=Homo sapiens OX=9606 GN=MAPT PE=1 SV=5 | MAPT | _VQSK[Lac (K)]IGSLDNITHVPGGGNK_ |
| P10636 | 686 | K | 0.31540524 | Down | Microtubule-associated protein tau OS=Homo sapiens OX=9606 GN=MAPT PE=1 SV=5 | MAPT | _IGSLDNITHVPGGGNK[Lac (K)]K_ |
| P10809 | 125 | K | 1.881262115 | Up | 60 kDa heat shock protein, mitochondrial OS=Homo sapiens OX=9606 GN=HSPD1 PE=1 SV=2 | HSPD1 | _SIAK[Lac (K)]EGFEK_ |
| P10809 | 473 | K | 1.586930322 | Up | 60 kDa heat shock protein, mitochondrial OS=Homo sapiens OX=9606 GN=HSPD1 PE=1 SV=2 | HSPD1 | _TLK[Lac (K)]IPAMTIAK_ |
| P11021 | 353 | K | 0.619468074 | Down | Endoplasmic reticulum chaperone BiP OS=Homo sapiens OX=9606 GN=HSPA5 PE=1 SV=2 | HSPA5 | _K[Lac (K)]SDIDEIVLVGGSTR_ |
| P11021 | 523 | K | 1.634652337 | Up | Endoplasmic reticulum chaperone BiP OS=Homo sapiens OX=9606 GN=HSPA5 PE=1 SV=2 | HSPA5 | _NK[Lac (K)]ITITNDQNR_ |
| P11387 | 137 | K | 0.338622158 | Down | DNA topoisomerase 1 OS=Homo sapiens OX=9606 GN=TOP1 PE=1 SV=2 | TOP1 | _EDIKPLK[Lac (K)]R_ |
| P11387 | 150 | K | 0.27726617 | Down | DNA topoisomerase 1 OS=Homo sapiens OX=9606 GN=TOP1 PE=1 SV=2 | TOP1 | _DEDDADYKPK[Lac (K)]K_ |
| P11387 | 164 | K | 0.437424082 | Down | DNA topoisomerase 1 OS=Homo sapiens OX=9606 GN=TOP1 PE=1 SV=2 | TOP1 | _K[Lac (K)]LEEEEDGK_ |
| P11387 | 172 | K | 0.172934115 | Down | DNA topoisomerase 1 OS=Homo sapiens OX=9606 GN=TOP1 PE=1 SV=2 | TOP1 | _KLEEEEDGK[Lac (K)]LK_ |
| P11387 | 202 | K | 0.405722441 | Down | DNA topoisomerase 1 OS=Homo sapiens OX=9606 GN=TOP1 PE=1 SV=2 | TOP1 | _KEEEQK[Lac (K)]WK_ |
| P11387 | 642 | K | 0.239911553 | Down | DNA topoisomerase 1 OS=Homo sapiens OX=9606 GN=TOP1 PE=1 SV=2 | TOP1 | _TFEK[Lac (K)]SMMNLQTK_ |
| P11388 | 614 | K | 0.54323757 | Down | DNA topoisomerase 2-alpha OS=Homo sapiens OX=9606 GN=TOP2A PE=1 SV=3 | TOP2A | _YYK[Lac (K)]GLGTSTSK_ |
| P11388 | 1196 | K | 0.536754052 | Down | DNA topoisomerase 2-alpha OS=Homo sapiens OX=9606 GN=TOP2A PE=1 SV=3 | TOP2A | _EKQDEQVGLPGK[Lac (K)]GGK_ |
| P11388 | 1204 | K | 0.214097199 | Down | DNA topoisomerase 2-alpha OS=Homo sapiens OX=9606 GN=TOP2A PE=1 SV=3 | TOP2A | _K[Lac (K)]TQMAEVLPSPR_ |
| P11388 | 1373 | K | 0.418747686 | Down | DNA topoisomerase 2-alpha OS=Homo sapiens OX=9606 GN=TOP2A PE=1 SV=3 | TOP2A | _ELKPQK[Lac (K)]SVVSDLEADDVK_ |
| P11388 | 1484 | K | 0.546687223 | Down | DNA topoisomerase 2-alpha OS=Homo sapiens OX=9606 GN=TOP2A PE=1 SV=3 | TOP2A | _IVSK[Lac (K)]AVTSK_ |
| P11388 | 1492 | K | 0.62756275 | Down | DNA topoisomerase 2-alpha OS=Homo sapiens OX=9606 GN=TOP2A PE=1 SV=3 | TOP2A | _SK[Lac (K)]GESDDFHMDFDSAVAPR_ |
| P11474 | 76 | K | 0.186299201 | Down | Steroid hormone receptor ERR1 OS=Homo sapiens OX=9606 GN=ESRRA PE=1 SV=3 | ESRRA | _LVLSSLPK[Lac (K)]R_ |
| P11717 | 2470 | K | 0.631491098 | Down | Cation-independent mannose-6-phosphate receptor OS=Homo sapiens OX=9606 GN=IGF2R PE=1 SV=3 | IGF2R | _SSSAQQK[Lac (K)]TVSSTK_ |
| P12004 | 80 | K | 1.539091956 | Up | Proliferating cell nuclear antigen OS=Homo sapiens OX=9606 GN=PCNA PE=1 SV=1 | PCNA | _ILK[Lac (K)]C[Carbamidomethyl (C)]AGNEDIITLR_ |
| P12268 | 422 | K | 3.882294886 | Up | Inosine-5'-monophosphate dehydrogenase 2 OS=Homo sapiens OX=9606 GN=IMPDH2 PE=1 SV=2 | IMPDH2 | _GMGSLDAMDK[Lac (K)]HLSSQNR_ |
| P12270 | 925 | K | 0.544413641 | Down | Nucleoprotein TPR OS=Homo sapiens OX=9606 GN=TPR PE=1 SV=3 | TPR | _TGK[Lac (K)]GQPSNKEDVDDLVSQLR_ |
| P12270 | 1347 | K | 0.35123524 | Down | Nucleoprotein TPR OS=Homo sapiens OX=9606 GN=TPR PE=1 SV=3 | TPR | _NQHLVSQQK[Lac (K)]DPDTEEYRK_ |
| P12270 | 1637 | K | 0.527512872 | Down | Nucleoprotein TPR OS=Homo sapiens OX=9606 GN=TPR PE=1 SV=3 | TPR | _DEPQEPSNK[Lac (K)]VPEQQR_ |
| P12270 | 1648 | K | 0.623404846 | Down | Nucleoprotein TPR OS=Homo sapiens OX=9606 GN=TPR PE=1 SV=3 | TPR | _QITLK[Lac (K)]TTPASGER_ |
| P12270 | 1690 | K | 0.411529755 | Down | Nucleoprotein TPR OS=Homo sapiens OX=9606 GN=TPR PE=1 SV=3 | TPR | _VTAAAMAGNK[Lac (K)]STPR_ |
| P12277 | 11 | K | 0.35238863 | Down | Creatine kinase B-type OS=Homo sapiens OX=9606 GN=CKB PE=1 SV=1 | CKB | _PFSNSHNALK[Lac (K)]LR_ |
| P12755 | 200 | K | 0.092843569 | Down | Ski oncogene OS=Homo sapiens OX=9606 GN=SKI PE=1 SV=1 | SKI | _LC[Carbamidomethyl (C)]NALLYGGAYPPPC[Carbamidomethyl (C)]K[Lac (K)]K_ |
| P12755 | 365 | K | 0.431818945 | Down | Ski oncogene OS=Homo sapiens OX=9606 GN=SKI PE=1 SV=1 | SKI | _TLAGSSNK[Lac (K)]SLGC[Carbamidomethyl (C)]VHPR_ |
| P12755 | 652 | K | 0.374912931 | Down | Ski oncogene OS=Homo sapiens OX=9606 GN=SKI PE=1 SV=1 | SKI | _VC[Carbamidomethyl (C)]DK[Lac (K)]GC[Carbamidomethyl (C)]EAGR_ |
| P12757 | 45 | K | 0.44442192 | Down | Ski-like protein OS=Homo sapiens OX=9606 GN=SKIL PE=1 SV=2 | SKIL | _TINK[Lac (K)]VPTVK_ |
| P12956 | 287 | K | 0.562192477 | Down | X-ray repair cross-complementing protein 6 OS=Homo sapiens OX=9606 GN=XRCC6 PE=1 SV=2 | XRCC6 | _ALKPPPIK[Lac (K)]LYR_ |
| P12956 | 317 | K | 0.575297351 | Down | X-ray repair cross-complementing protein 6 OS=Homo sapiens OX=9606 GN=XRCC6 PE=1 SV=2 | XRCC6 | _TFNTSTGGLLLPSDTK[Lac (K)]R_ |
| P12956 | 451 | K | 0.521444138 | Down | X-ray repair cross-complementing protein 6 OS=Homo sapiens OX=9606 GN=XRCC6 PE=1 SV=2 | XRCC6 | _MPFTEK[Lac (K)]IMATPEQVGK_ |
| P12956 | 556 | K | 0.391272881 | Down | X-ray repair cross-complementing protein 6 OS=Homo sapiens OX=9606 GN=XRCC6 PE=1 SV=2 | XRCC6 | _RPK[Lac (K)]VEYSEEELK_ |
| P12956 | 582 | K | 0.507318256 | Down | X-ray repair cross-complementing protein 6 OS=Homo sapiens OX=9606 GN=XRCC6 PE=1 SV=2 | XRCC6 | _FTVPMLK[Lac (K)]EAC[Carbamidomethyl (C)]R_ |
| P12956 | 591 | K | 0.249951534 | Down | X-ray repair cross-complementing protein 6 OS=Homo sapiens OX=9606 GN=XRCC6 PE=1 SV=2 | XRCC6 | _AYGLK[Lac (K)]SGLK_ |
| P12956 | 596 | K | 0.085188932 | Down | X-ray repair cross-complementing protein 6 OS=Homo sapiens OX=9606 GN=XRCC6 PE=1 SV=2 | XRCC6 | _K[Lac (K)]QELLEALTK_ |
| P13010 | 274 | K | 0.437519984 | Down | X-ray repair cross-complementing protein 5 OS=Homo sapiens OX=9606 GN=XRCC5 PE=1 SV=3 | XRCC5 | _K[Lac (K)]TWTVVDAK_ |
| P13010 | 543 | K | 0.148016769 | Down | X-ray repair cross-complementing protein 5 OS=Homo sapiens OX=9606 GN=XRCC5 PE=1 SV=3 | XRCC5 | _TLFPLIEAK[Lac (K)]K_ |
| P13010 | 565 | K | 0.31515548 | Down | X-ray repair cross-complementing protein 5 OS=Homo sapiens OX=9606 GN=XRCC5 PE=1 SV=3 | XRCC5 | _DQVTAQEIFQDNHEDGPTAK[Lac (K)]K_ |
| P13639 | 571 | K | 1.59470127 | Up | Elongation factor 2 OS=Homo sapiens OX=9606 GN=EEF2 PE=1 SV=4 | EEF2 | _DLEEDHAC[Carbamidomethyl (C)]IPIK[Lac (K)]K_ |
| P13645 | 343 | K | 1.829177413 | Up | Keratin, type I cytoskeletal 10 OS=Homo sapiens OX=9606 GN=KRT10 PE=1 SV=6 | KRT10 | _DAEAWFNEK[Lac (K)]SK_ |
| P13667 | 211 | K | 0.282361225 | Down | Protein disulfide-isomerase A4 OS=Homo sapiens OX=9606 GN=PDIA4 PE=1 SV=2 | PDIA4 | _K[Lac (K)]LAPEYEK_ |
| P13693 | 93 | K | 2.487260959 | Up | Translationally-controlled tumor protein OS=Homo sapiens OX=9606 GN=TPT1 PE=1 SV=1 | TPT1 | _YIK[Lac (K)]DYMK_ |
| P13984 | 25 | K | 0.65328624 | Down | General transcription factor IIF subunit 2 OS=Homo sapiens OX=9606 GN=GTF2F2 PE=1 SV=2 | GTF2F2 | _VPK[Lac (K)]YLSQQWAK_ |
| P13984 | 147 | K | 0.351493828 | Down | General transcription factor IIF subunit 2 OS=Homo sapiens OX=9606 GN=GTF2F2 PE=1 SV=2 | GTF2F2 | _LSQQLDK[Lac (K)]VVTTNYK_ |
| P13984 | 226 | K | 0.497712188 | Down | General transcription factor IIF subunit 2 OS=Homo sapiens OX=9606 GN=GTF2F2 PE=1 SV=2 | GTF2F2 | _EIGVQNVK[Lac (K)]GIHK_ |
| P13994 | 38 | K | 0.113494719 | Down | Probable splicing factor YJU2B OS=Homo sapiens OX=9606 GN=YJU2B PE=1 SV=2 | YJU2B | _K[Lac (K)]LSQGILIIR_ |
| P14324 | 123 | K | 2.017142423 | Up | Farnesyl pyrophosphate synthase OS=Homo sapiens OX=9606 GN=FDPS PE=1 SV=4 | FDPS | _EVLEYNAIGGK[Lac (K)]YNR_ |
| P14618 | 66 | K | 1.500320977 | Up | Pyruvate kinase PKM OS=Homo sapiens OX=9606 GN=PKM PE=1 SV=4 | PKM | _EMIK[Lac (K)]SGMNVAR_ |
| P14618 | 166 | K | 1.530525873 | Up | Pyruvate kinase PKM OS=Homo sapiens OX=9606 GN=PKM PE=1 SV=4 | PKM | _NIC[Carbamidomethyl (C)]K[Lac (K)]VVEVGSK_ |
| P14618 | 433 | K | 2.709921497 | Up | Pyruvate kinase PKM OS=Homo sapiens OX=9606 GN=PKM PE=1 SV=4 | PKM | _C[Carbamidomethyl (C)]C[Carbamidomethyl (C)]SGAIIVLTK[Lac (K)]SGR_ |
| P14678 | 8 | K | 1.784640424 | Up | Small nuclear ribonucleoprotein-associated proteins B and B' OS=Homo sapiens OX=9606 GN=SNRPB PE=1 SV=2 | SNRPB | _SSK[Lac (K)]MLQHIDYR_ |
| P14735 | 899 | K | 2.063876855 | Up | Insulin-degrading enzyme OS=Homo sapiens OX=9606 GN=IDE PE=1 SV=4 | IDE | _K[Lac (K)]LSAEC[Carbamidomethyl (C)]AK_ |
| P14866 | 34 | K | 0.33020467 | Down | Heterogeneous nuclear ribonucleoprotein L OS=Homo sapiens OX=9606 GN=HNRNPL PE=1 SV=2 | HNRNPL | _SGAMVK[Lac (K)]MAAAGGGGGGGR_ |
| P14866 | 418 | K | 0.587925741 | Down | Heterogeneous nuclear ribonucleoprotein L OS=Homo sapiens OX=9606 GN=HNRNPL PE=1 SV=2 | HNRNPL | _SK[Lac (K)]PGAAMVEMADGYAVDR_ |
| P15311 | 64 | K | 2.255694653 | Up | Ezrin OS=Homo sapiens OX=9606 GN=EZR PE=1 SV=4 | EZR | _K[Lac (K)]VSAQEVR_ |
| P15336 | 54 | K | 0.407708689 | Down | Cyclic AMP-dependent transcription factor ATF-2 OS=Homo sapiens OX=9606 GN=ATF2 PE=1 SV=4 | ATF2 | _HEMTLK[Lac (K)]FGPAR_ |
| P15407 | 173 | K | 0.252495777 | Down | Fos-related antigen 1 OS=Homo sapiens OX=9606 GN=FOSL1 PE=1 SV=1 | FOSL1 | _LELVLEAHRPIC[Carbamidomethyl (C)]K[Lac (K)]IPEGAK_ |
| P15531 | 100 | K | 1.649865036 | Up | Nucleoside diphosphate kinase A OS=Homo sapiens OX=9606 GN=NME1 PE=1 SV=1 | NME1 | _VMLGETNPADSK[Lac (K)]PGTIR_ |
| P15924 | 2458 | K | 0.486934347 | Down | Desmoplakin OS=Homo sapiens OX=9606 GN=DSP PE=1 SV=3 | DSP | _QVQTSQK[Lac (K)]NTLR_ |
| P15924 | 2592 | K | 0.489457927 | Down | Desmoplakin OS=Homo sapiens OX=9606 GN=DSP PE=1 SV=3 | DSP | _HESVSK[Lac (K)]ISTISSVR_ |
| P16220 | 122 | K | 0.31622532 | Down | Cyclic AMP-responsive element-binding protein 1 OS=Homo sapiens OX=9606 GN=CREB1 PE=1 SV=3 | CREB1 | _K[Lac (K)]ILNDLSSDAPGVPR_ |
| P16220 | 295 | K | 0.329535308 | Down | Cyclic AMP-responsive element-binding protein 1 OS=Homo sapiens OX=9606 GN=CREB1 PE=1 SV=3 | CREB1 | _EYVK[Lac (K)]C[Carbamidomethyl (C)]LENR_ |
| P16591 | 420 | K | 0.503621189 | Down | Tyrosine-protein kinase Fer OS=Homo sapiens OX=9606 GN=FER PE=1 SV=2 | FER | _LSK[Lac (K)]FESIR_ |
| P16949 | 29 | K | 0.404773603 | Down | Stathmin OS=Homo sapiens OX=9606 GN=STMN1 PE=1 SV=3 | STMN1 | _SK[Lac (K)]ESVPEFPLSPPK_ |
| P16949 | 41 | K | 0.592037047 | Down | Stathmin OS=Homo sapiens OX=9606 GN=STMN1 PE=1 SV=3 | STMN1 | _ESVPEFPLSPPK[Lac (K)]K_ |
| P16949 | 43 | K | 0.657018674 | Down | Stathmin OS=Homo sapiens OX=9606 GN=STMN1 PE=1 SV=3 | STMN1 | _K[Lac (K)]DLSLEEIQK_ |
| P16949 | 52 | K | 0.534396968 | Down | Stathmin OS=Homo sapiens OX=9606 GN=STMN1 PE=1 SV=3 | STMN1 | _DLSLEEIQK[Lac (K)]K_ |
| P16949 | 70 | K | 0.357850565 | Down | Stathmin OS=Homo sapiens OX=9606 GN=STMN1 PE=1 SV=3 | STMN1 | _SHEAEVLK[Lac (K)]QLAEK_ |
| P16989 | 124 | K | 2.055420173 | Up | Y-box-binding protein 3 OS=Homo sapiens OX=9606 GN=YBX3 PE=1 SV=4 | YBX3 | _EDVFVHQTAIK[Lac (K)]K_ |
| P17096 | 31 | K | 0.583201273 | Down | High mobility group protein HMG-I/HMG-Y OS=Homo sapiens OX=9606 GN=HMGA1 PE=1 SV=3 | HMGA1 | _K[Lac (K)]QPPVSPGTALVGSQK_ |
| P17275 | 51 | K | 0.239245182 | Down | Transcription factor JunB OS=Homo sapiens OX=9606 GN=JUNB PE=1 SV=1 | JUNB | _SLK[Lac (K)]APGAR_ |
| P17480 | 107 | K | 0.632438765 | Down | Nucleolar transcription factor 1 OS=Homo sapiens OX=9606 GN=UBTF PE=1 SV=1 | UBTF | _K[Lac (K)]HPDFPK_ |
| P17480 | 132 | K | 0.624956436 | Down | Nucleolar transcription factor 1 OS=Homo sapiens OX=9606 GN=UBTF PE=1 SV=1 | UBTF | _YAK[Lac (K)]LHPEMSNLDLTK_ |
| P17480 | 192 | K | 0.616965561 | Down | Nucleolar transcription factor 1 OS=Homo sapiens OX=9606 GN=UBTF PE=1 SV=1 | UBTF | _K[Lac (K)]SDIPEKPK_ |
| P17480 | 216 | K | 0.562583522 | Down | Nucleolar transcription factor 1 OS=Homo sapiens OX=9606 GN=UBTF PE=1 SV=1 | UBTF | _VYLK[Lac (K)]VRPDATTK_ |
| P17480 | 266 | K | 0.418936907 | Down | Nucleolar transcription factor 1 OS=Homo sapiens OX=9606 GN=UBTF PE=1 SV=1 | UBTF | _DYIQK[Lac (K)]HPELNISEEGITK_ |
| P17480 | 279 | K | 0.22088445 | Down | Nucleolar transcription factor 1 OS=Homo sapiens OX=9606 GN=UBTF PE=1 SV=1 | UBTF | _HPELNISEEGITK[Lac (K)]STLTK_ |
| P17480 | 562 | K | 0.578853464 | Down | Nucleolar transcription factor 1 OS=Homo sapiens OX=9606 GN=UBTF PE=1 SV=1 | UBTF | _MK[Lac (K)]FQGEPK_ |
| P17544 | 354 | K | 0.133544095 | Down | Cyclic AMP-dependent transcription factor ATF-7 OS=Homo sapiens OX=9606 GN=ATF7 PE=1 SV=3 | ATF7 | _K[Lac (K)]LWVSSLEK_ |
| P17844 | 33 | K | 0.199579103 | Down | Probable ATP-dependent RNA helicase DDX5 OS=Homo sapiens OX=9606 GN=DDX5 PE=1 SV=1 | DDX5 | _K[Lac (K)]FGNPGEK_ |
| P17844 | 40 | K | 0.231823355 | Down | Probable ATP-dependent RNA helicase DDX5 OS=Homo sapiens OX=9606 GN=DDX5 PE=1 SV=1 | DDX5 | _FGNPGEK[Lac (K)]LVK_ |
| P17844 | 45 | K | 0.11275088 | Down | Probable ATP-dependent RNA helicase DDX5 OS=Homo sapiens OX=9606 GN=DDX5 PE=1 SV=1 | DDX5 | _K[Lac (K)]WNLDELPK_ |
| P17844 | 351 | K | 0.569058671 | Down | Probable ATP-dependent RNA helicase DDX5 OS=Homo sapiens OX=9606 GN=DDX5 PE=1 SV=1 | DDX5 | _TIVFVETK[Lac (K)]R_ |
| P17844 | 523 | K | 0.263171476 | Down | Probable ATP-dependent RNA helicase DDX5 OS=Homo sapiens OX=9606 GN=DDX5 PE=1 SV=1 | DDX5 | _GYSSLLK[Lac (K)]R_ |
| P17858 | 677 | K | 1.693057819 | Up | ATP-dependent 6-phosphofructokinase, liver type OS=Homo sapiens OX=9606 GN=PFKL PE=1 SV=6 | PFKL | _NYGTK[Lac (K)]LGVK_ |
| P17987 | 126 | K | 1.804058865 | Up | T-complex protein 1 subunit alpha OS=Homo sapiens OX=9606 GN=TCP1 PE=1 SV=1 | TCP1 | _LAC[Carbamidomethyl (C)]K[Lac (K)]EAVR_ |
| P18031 | 120 | K | 0.55836267 | Down | Tyrosine-protein phosphatase non-receptor type 1 OS=Homo sapiens OX=9606 GN=PTPN1 PE=1 SV=1 | PTPN1 | _GSLK[Lac (K)]C[Carbamidomethyl (C)]AQYWPQK_ |
| P18031 | 314 | K | 0.277889274 | Down | Tyrosine-protein phosphatase non-receptor type 1 OS=Homo sapiens OX=9606 GN=PTPN1 PE=1 SV=1 | PTPN1 | _ELSHEDLEPPPEHIPPPPRPPK[Lac (K)]R_ |
| P18031 | 323 | K | 0.549406367 | Down | Tyrosine-protein phosphatase non-receptor type 1 OS=Homo sapiens OX=9606 GN=PTPN1 PE=1 SV=1 | PTPN1 | _ILEPHNGK[Lac (K)]C[Carbamidomethyl (C)]R_ |
| P18583 | 16 | K | 0.436426608 | Down | Protein SON OS=Homo sapiens OX=9606 GN=SON PE=1 SV=4 | SON | _SFVVSK[Lac (K)]FR_ |
| P18583 | 84 | K | 0.407772592 | Down | Protein SON OS=Homo sapiens OX=9606 GN=SON PE=1 SV=4 | SON | _YKPDLK[Lac (K)]EGSR_ |
| P18583 | 106 | K | 0.395513115 | Down | Protein SON OS=Homo sapiens OX=9606 GN=SON PE=1 SV=4 | SON | _C[Carbamidomethyl (C)]VSVQTDPTDEIPTK[Lac (K)]K_ |
| P18583 | 141 | K | 0.628276781 | Down | Protein SON OS=Homo sapiens OX=9606 GN=SON PE=1 SV=4 | SON | _TK[Lac (K)]SHDDGNIDLESDSFLK_ |
| P18583 | 277 | K | 0.447669697 | Down | Protein SON OS=Homo sapiens OX=9606 GN=SON PE=1 SV=4 | SON | _SVLK[Lac (K)]SVESTSPEPSK_ |
| P18583 | 1740 | K | 0.351412162 | Down | Protein SON OS=Homo sapiens OX=9606 GN=SON PE=1 SV=4 | SON | _PLLPK[Lac (K)]DMER_ |
| P18583 | 2096 | K | 0.342193517 | Down | Protein SON OS=Homo sapiens OX=9606 GN=SON PE=1 SV=4 | SON | _K[Lac (K)]SGGATIEELTEK_ |
| P18583 | 2108 | K | 0.230108869 | Down | Protein SON OS=Homo sapiens OX=9606 GN=SON PE=1 SV=4 | SON | _SGGATIEELTEK[Lac (K)]C[Carbamidomethyl (C)]K_ |
| P18583 | 2166 | K | 0.395193655 | Down | Protein SON OS=Homo sapiens OX=9606 GN=SON PE=1 SV=4 | SON | _PTPPK[Lac (K)]SQVTLTK_ |
| P18583 | 2173 | K | 0.238190231 | Down | Protein SON OS=Homo sapiens OX=9606 GN=SON PE=1 SV=4 | SON | _SQVTLTK[Lac (K)]EFPVSSGSQHR_ |
| P18583 | 2365 | K | 0.159795513 | Down | Protein SON OS=Homo sapiens OX=9606 GN=SON PE=1 SV=4 | SON | _SGNFSAAMK[Lac (K)]DLSGK_ |
| P18615 | 70 | K | 0.219702505 | Down | Negative elongation factor E OS=Homo sapiens OX=9606 GN=NELFE PE=1 SV=3 | NELFE | _QLVK[Lac (K)]SGAISAIK_ |
| P18615 | 130 | K | 0.232041078 | Down | Negative elongation factor E OS=Homo sapiens OX=9606 GN=NELFE PE=1 SV=3 | NELFE | _K[Lac (K)]SLYESFVSSSDR_ |
| P18621 | 167 | K | 1.686720841 | Up | Large ribosomal subunit protein uL22 OS=Homo sapiens OX=9606 GN=RPL17 PE=1 SV=3 | RPL17 | _EQIVPKPEEEVAQK[Lac (K)]K_ |
| P18669 | 106 | K | 1.613283184 | Up | Phosphoglycerate mutase 1 OS=Homo sapiens OX=9606 GN=PGAM1 PE=1 SV=2 | PGAM1 | _AETAAK[Lac (K)]HGEAQVK_ |
| P18669 | 251 | K | 3.606959208 | Up | Phosphoglycerate mutase 1 OS=Homo sapiens OX=9606 GN=PGAM1 PE=1 SV=2 | PGAM1 | _AMEAVAAQGK[Lac (K)]AK_ |
| P18858 | 36 | K | 0.61307057 | Down | DNA ligase 1 OS=Homo sapiens OX=9606 GN=LIG1 PE=1 SV=1 | LIG1 | _ETEPPPK[Lac (K)]AALK_ |
| P18858 | 226 | K | 0.327989668 | Down | DNA ligase 1 OS=Homo sapiens OX=9606 GN=LIG1 PE=1 SV=1 | LIG1 | _RAPK[Lac (K)]TLSSFFTPR_ |
| P18887 | 169 | K | 0.546978776 | Down | DNA repair protein XRCC1 OS=Homo sapiens OX=9606 GN=XRCC1 PE=1 SV=3 | XRCC1 | _VTVTK[Lac (K)]LGQFR_ |
| P18887 | 247 | K | 0.556567852 | Down | DNA repair protein XRCC1 OS=Homo sapiens OX=9606 GN=XRCC1 PE=1 SV=3 | XRCC1 | _K[Lac (K)]LDLNQEEK_ |
| P18887 | 274 | K | 0.542751293 | Down | DNA repair protein XRCC1 OS=Homo sapiens OX=9606 GN=XRCC1 PE=1 SV=3 | XRCC1 | _RPK[Lac (K)]LPAPTR_ |
| P18887 | 298 | K | 1.521905937 | Up | DNA repair protein XRCC1 OS=Homo sapiens OX=9606 GN=XRCC1 PE=1 SV=3 | XRCC1 | _AQGAVTGK[Lac (K)]PR_ |
| P19338 | 63 | K | 0.503130986 | Down | Nucleolin OS=Homo sapiens OX=9606 GN=NCL PE=1 SV=3 | NCL | _K[Lac (K)]VVVSPTK_ |
| P19338 | 70 | K | 0.468183263 | Down | Nucleolin OS=Homo sapiens OX=9606 GN=NCL PE=1 SV=3 | NCL | _VVVSPTK[Lac (K)]K_ |
| P19338 | 71 | K | 0.387120989 | Down | Nucleolin OS=Homo sapiens OX=9606 GN=NCL PE=1 SV=3 | NCL | _K[Lac (K)]VAVATPAK_ |
| P19338 | 79 | K | 0.430477947 | Down | Nucleolin OS=Homo sapiens OX=9606 GN=NCL PE=1 SV=3 | NCL | _VAVATPAK[Lac (K)]K_ |
| P19338 | 102 | K | 0.333139175 | Down | Nucleolin OS=Homo sapiens OX=9606 GN=NCL PE=1 SV=3 | NCL | _TVTPAK[Lac (K)]AVTTPGK_ |
| P19338 | 109 | K | 0.535952425 | Down | Nucleolin OS=Homo sapiens OX=9606 GN=NCL PE=1 SV=3 | NCL | _AVTTPGK[Lac (K)]K_ |
| P19338 | 116 | K | 0.443967378 | Down | Nucleolin OS=Homo sapiens OX=9606 GN=NCL PE=1 SV=3 | NCL | _GATPGK[Lac (K)]ALVATPGK_ |
| P19338 | 124 | K | 0.380840996 | Down | Nucleolin OS=Homo sapiens OX=9606 GN=NCL PE=1 SV=3 | NCL | _ALVATPGK[Lac (K)]K_ |
| P19338 | 132 | K | 0.440829255 | Down | Nucleolin OS=Homo sapiens OX=9606 GN=NCL PE=1 SV=3 | NCL | _GAAIPAK[Lac (K)]GAK_ |
| P19338 | 223 | K | 0.612293545 | Down | Nucleolin OS=Homo sapiens OX=9606 GN=NCL PE=1 SV=3 | NCL | _AAK[Lac (K)]VVPVK_ |
| P19338 | 545 | K | 1.763051015 | Up | Nucleolin OS=Homo sapiens OX=9606 GN=NCL PE=1 SV=3 | NCL | _EALNSC[Carbamidomethyl (C)]NK[Lac (K)]R_ |
| P20248 | 54 | K | 0.277774654 | Down | Cyclin-A2 OS=Homo sapiens OX=9606 GN=CCNA2 PE=1 SV=2 | CCNA2 | _AALAVLK[Lac (K)]SGNPR_ |
| P20810 | 43 | K | 0.533109582 | Down | Calpastatin OS=Homo sapiens OX=9606 GN=CAST PE=1 SV=4 | CAST | _SQSTK[Lac (K)]LSVVHEK_ |
| P20810 | 95 | K | 1.654904316 | Up | Calpastatin OS=Homo sapiens OX=9606 GN=CAST PE=1 SV=4 | CAST | _SAEQQPSEK[Lac (K)]STEPK_ |
| P20810 | 132 | K | 0.647519761 | Down | Calpastatin OS=Homo sapiens OX=9606 GN=CAST PE=1 SV=4 | CAST | _K[Lac (K)]SLTPAVPVESKPDKPSGK_ |
| P20810 | 204 | K | 0.581357081 | Down | Calpastatin OS=Homo sapiens OX=9606 GN=CAST PE=1 SV=4 | CAST | _EVTIPPK[Lac (K)]YR_ |
| P20810 | 261 | K | 0.622293798 | Down | Calpastatin OS=Homo sapiens OX=9606 GN=CAST PE=1 SV=4 | CAST | _EESTEVLK[Lac (K)]AQSAGTVR_ |
| P21281 | 109 | K | 0.650472119 | Down | V-type proton ATPase subunit B, brain isoform OS=Homo sapiens OX=9606 GN=ATP6V1B2 PE=1 SV=3 | ATP6V1B2 | _K[Lac (K)]TSC[Carbamidomethyl (C)]EFTGDILR_ |
| P21333 | 916 | K | 0.494295652 | Down | Filamin-A OS=Homo sapiens OX=9606 GN=FLNA PE=1 SV=4 | FLNA | _LDVQFSGLTK[Lac (K)]GDAVR_ |
| P21333 | 1071 | K | 0.332796062 | Down | Filamin-A OS=Homo sapiens OX=9606 GN=FLNA PE=1 SV=4 | FLNA | _VK[Lac (K)]AFGPGLQGGSAGSPAR_ |
| P21675 | 1365 | K | 0.333800107 | Down | Transcription initiation factor TFIID subunit 1 OS=Homo sapiens OX=9606 GN=TAF1 PE=1 SV=3 | TAF1 | _SLVLK[Lac (K)]FPK_ |
| P22087 | 143 | K | 0.200167553 | Down | rRNA 2'-O-methyltransferase fibrillarin OS=Homo sapiens OX=9606 GN=FBL PE=1 SV=2 | FBL | _SK[Lac (K)]LAAAILGGVDQIHIKPGAK_ |
| P22234 | 17 | K | 1.690055316 | Up | Bifunctional phosphoribosylaminoimidazole carboxylase/phosphoribosylaminoimidazole succinocarboxamide synthetase OS=Homo sapiens OX=9606 GN=PAICS PE=1 SV=3 | PAICS | _LYEGK[Lac (K)]TK_ |
| P22234 | 247 | K | 0.566941756 | Down | Bifunctional phosphoribosylaminoimidazole carboxylase/phosphoribosylaminoimidazole succinocarboxamide synthetase OS=Homo sapiens OX=9606 GN=PAICS PE=1 SV=3 | PAICS | _K[Lac (K)]NFEWVAER_ |
| P22626 | 104 | K | 1.551719386 | Up | Heterogeneous nuclear ribonucleoproteins A2/B1 OS=Homo sapiens OX=9606 GN=HNRNPA2B1 PE=1 SV=2 | HNRNPA2B1 | _EESGK[Lac (K)]PGAHVTVK_ |
| P22681 | 7 | K | 0.642231111 | Down | E3 ubiquitin-protein ligase CBL OS=Homo sapiens OX=9606 GN=CBL PE=1 SV=2 | CBL | _K[Lac (K)]SSGAGGGSGSGGSGSGGLIGLMK_ |
| P23193 | 19 | K | 0.397499572 | Down | Transcription elongation factor A protein 1 OS=Homo sapiens OX=9606 GN=TCEA1 PE=1 SV=2 | TCEA1 | _K[Lac (K)]NAAGALDLLK_ |
| P23193 | 84 | K | 0.549067186 | Down | Transcription elongation factor A protein 1 OS=Homo sapiens OX=9606 GN=TCEA1 PE=1 SV=2 | TCEA1 | _LLDGPSTEK[Lac (K)]DLDEK_ |
| P23258 | 287 | K | 0.467860749 | Down | Tubulin gamma-1 chain OS=Homo sapiens OX=9606 GN=TUBG1 PE=1 SV=2 | TUBG1 | _K[Lac (K)]TTVLDVMR_ |
| P23284 | 116 | K | 1.965138473 | Up | Peptidyl-prolyl cis-trans isomerase B OS=Homo sapiens OX=9606 GN=PPIB PE=1 SV=2 | PPIB | _GDGTGGK[Lac (K)]SIYGER_ |
| P23284 | 215 | K | 1.663311169 | Up | Peptidyl-prolyl cis-trans isomerase B OS=Homo sapiens OX=9606 GN=PPIB PE=1 SV=2 | PPIB | _IEVEKPFAIAK[Lac (K)]E_ |
| P23396 | 10 | K | 0.613153409 | Down | Small ribosomal subunit protein uS3 OS=Homo sapiens OX=9606 GN=RPS3 PE=1 SV=2 | RPS3 | _K[Lac (K)]FVADGIFK_ |
| P23396 | 62 | K | 1.743015185 | Up | Small ribosomal subunit protein uS3 OS=Homo sapiens OX=9606 GN=RPS3 PE=1 SV=2 | RPS3 | _TQNVLGEK[Lac (K)]GR_ |
| P23396 | 108 | K | 0.62097746 | Down | Small ribosomal subunit protein uS3 OS=Homo sapiens OX=9606 GN=RPS3 PE=1 SV=2 | RPS3 | _YK[Lac (K)]LLGGLAVR_ |
| P23396 | 230 | K | 0.644146437 | Down | Small ribosomal subunit protein uS3 OS=Homo sapiens OX=9606 GN=RPS3 PE=1 SV=2 | RPS3 | _GGK[Lac (K)]PEPPAMPQPVPTA_ |
| P23443 | 431 | K | 0.499313597 | Down | Ribosomal protein S6 kinase beta-1 OS=Homo sapiens OX=9606 GN=RPS6KB1 PE=1 SV=2 | RPS6KB1 | _FSFEPK[Lac (K)]IR_ |
| P23497 | 306 | K | 0.284272685 | Down | Nuclear autoantigen Sp-100 OS=Homo sapiens OX=9606 GN=SP100 PE=1 SV=3 | SP100 | _EKPFSNSK[Lac (K)]VEC[Carbamidomethyl (C)]QAQAR_ |
| P23527 | 47 | K | 3.534336326 | Up | Histone H2B type 1-O OS=Homo sapiens OX=9606 GN=H2BC17 PE=1 SV=3 | H2BC17 | _VLK[Lac (K)]QVHPDTGISSK_ |
| P23527 | 86 | K | 3.239372277 | Up | Histone H2B type 1-O OS=Homo sapiens OX=9606 GN=H2BC17 PE=1 SV=3 | H2BC17 | _LAHYNK[Lac (K)]R_ |
| P23527 | 117 | K | 1.993018907 | Up | Histone H2B type 1-O OS=Homo sapiens OX=9606 GN=H2BC17 PE=1 SV=3 | H2BC17 | _HAVSEGTK[Lac (K)]AVTK_ |
| P23527 | 121 | K | 1.578052518 | Up | Histone H2B type 1-O OS=Homo sapiens OX=9606 GN=H2BC17 PE=1 SV=3 | H2BC17 | _AVTK[Lac (K)]YTSSK_ |
| P23528 | 22 | K | 1.877757841 | Up | Cofilin-1 OS=Homo sapiens OX=9606 GN=CFL1 PE=1 SV=3 | CFL1 | _K[Lac (K)]SSTPEEVK_ |
| P23528 | 30 | K | 1.784256923 | Up | Cofilin-1 OS=Homo sapiens OX=9606 GN=CFL1 PE=1 SV=3 | CFL1 | _SSTPEEVK[Lac (K)]K_ |
| P23528 | 92 | K | 1.566847679 | Up | Cofilin-1 OS=Homo sapiens OX=9606 GN=CFL1 PE=1 SV=3 | CFL1 | _YALYDATYETK[Lac (K)]ESK_ |
| P23588 | 333 | K | 0.523233185 | Down | Eukaryotic translation initiation factor 4B OS=Homo sapiens OX=9606 GN=EIF4B PE=1 SV=2 | EIF4B | _GPPQRPK[Lac (K)]LNLKPR_ |
| P23588 | 343 | K | 1.640467957 | Up | Eukaryotic translation initiation factor 4B OS=Homo sapiens OX=9606 GN=EIF4B PE=1 SV=2 | EIF4B | _STPK[Lac (K)]EDDSSASTSQSTR_ |
| P23588 | 586 | K | 0.579962562 | Down | Eukaryotic translation initiation factor 4B OS=Homo sapiens OX=9606 GN=EIF4B PE=1 SV=2 | EIF4B | _KPEENPASK[Lac (K)]FSSASK_ |
| P24928 | 1866 | K | 0.634152615 | Down | DNA-directed RNA polymerase II subunit RPB1 OS=Homo sapiens OX=9606 GN=POLR2A PE=1 SV=2 | POLR2A | _YSPTSPK[Lac (K)]YSPTSPK_ |
| P24941 | 9 | K | 0.626952708 | Down | Cyclin-dependent kinase 2 OS=Homo sapiens OX=9606 GN=CDK2 PE=1 SV=2 | CDK2 | _VEK[Lac (K)]IGEGTYGVVYK_ |
| P25205 | 152 | K | 0.56526401 | Down | DNA replication licensing factor MCM3 OS=Homo sapiens OX=9606 GN=MCM3 PE=1 SV=3 | MCM3 | _SVHYC[Carbamidomethyl (C)]PATK[Lac (K)]K_ |
| P25205 | 556 | K | 1.571078606 | Up | DNA replication licensing factor MCM3 OS=Homo sapiens OX=9606 GN=MCM3 PE=1 SV=3 | MCM3 | _HDNLLHGTK[Lac (K)]K_ |
| P25440 | 193 | K | 0.360886741 | Down | Bromodomain-containing protein 2 OS=Homo sapiens OX=9606 GN=BRD2 PE=1 SV=2 | BRD2 | _VASMPQEEQELVVTIPK[Lac (K)]NSHK_ |
| P25440 | 275 | K | 0.22727264 | Down | Bromodomain-containing protein 2 OS=Homo sapiens OX=9606 GN=BRD2 PE=1 SV=2 | BRD2 | _SLHSAGPPLLAVTAAPPAQPLAK[Lac (K)]K_ |
| P25440 | 329 | K | 0.643885099 | Down | Bromodomain-containing protein 2 OS=Homo sapiens OX=9606 GN=BRD2 PE=1 SV=2 | BRD2 | _K[Lac (K)]DLPDSQQQHQSSK_ |
| P25440 | 614 | K | 0.138645354 | Down | Bromodomain-containing protein 2 OS=Homo sapiens OX=9606 GN=BRD2 PE=1 SV=2 | BRD2 | _KASGSGGGSAALGPSGFGPSGGSGTK[Lac (K)]LPK_ |
| P25440 | 732 | K | 0.467746333 | Down | Bromodomain-containing protein 2 OS=Homo sapiens OX=9606 GN=BRD2 PE=1 SV=2 | BRD2 | _TKEELALEK[Lac (K)]K_ |
| P25440 | 751 | K | 0.347351587 | Down | Bromodomain-containing protein 2 OS=Homo sapiens OX=9606 GN=BRD2 PE=1 SV=2 | BRD2 | _LQDVSGQLNSTK[Lac (K)]KPPK_ |
| P25440 | 760 | K | 0.518016006 | Down | Bromodomain-containing protein 2 OS=Homo sapiens OX=9606 GN=BRD2 PE=1 SV=2 | BRD2 | _ANEK[Lac (K)]TESSSAQQVAVSR_ |
| P25490 | 183 | K | 0.304552386 | Down | Transcriptional repressor protein YY1 OS=Homo sapiens OX=9606 GN=YY1 PE=1 SV=2 | YY1 | _K[Lac (K)]SYLSGGAGAAGGGGADPGNK_ |
| P25490 | 203 | K | 0.245314874 | Down | Transcriptional repressor protein YY1 OS=Homo sapiens OX=9606 GN=YY1 PE=1 SV=2 | YY1 | _SYLSGGAGAAGGGGADPGNK[Lac (K)]K_ |
| P25490 | 208 | K | 0.428932938 | Down | Transcriptional repressor protein YY1 OS=Homo sapiens OX=9606 GN=YY1 PE=1 SV=2 | YY1 | _WEQK[Lac (K)]QVQIK_ |
| P25490 | 301 | K | 1.506574938 | Up | Transcriptional repressor protein YY1 OS=Homo sapiens OX=9606 GN=YY1 PE=1 SV=2 | YY1 | _TIAC[Carbamidomethyl (C)]PHK[Lac (K)]GC[Carbamidomethyl (C)]TK_ |
| P25490 | 305 | K | 0.637895026 | Down | Transcriptional repressor protein YY1 OS=Homo sapiens OX=9606 GN=YY1 PE=1 SV=2 | YY1 | _GC[Carbamidomethyl (C)]TK[Lac (K)]MFR_ |
| P25685 | 159 | K | 0.512263181 | Down | DnaJ homolog subfamily B member 1 OS=Homo sapiens OX=9606 GN=DNAJB1 PE=1 SV=4 | DNAJB1 | _K[Lac (K)]QDPPVTHDLR_ |
| P25685 | 195 | K | 0.434978191 | Down | DnaJ homolog subfamily B member 1 OS=Homo sapiens OX=9606 GN=DNAJB1 PE=1 SV=4 | DNAJB1 | _LNPDGK[Lac (K)]SIR_ |
| P25705 | 531 | K | 2.221838222 | Up | ATP synthase subunit alpha, mitochondrial OS=Homo sapiens OX=9606 GN=ATP5F1A PE=1 SV=1 | ATP5F1A | _ADGK[Lac (K)]ISEQSDAK_ |
| P26358 | 173 | K | 0.517824651 | Down | DNA (cytosine-5)-methyltransferase 1 OS=Homo sapiens OX=9606 GN=DNMT1 PE=1 SV=2 | DNMT1 | _QTTITSHFAK[Lac (K)]GPAK_ |
| P26358 | 347 | K | 0.646598733 | Down | DNA (cytosine-5)-methyltransferase 1 OS=Homo sapiens OX=9606 GN=DNMT1 PE=1 SV=2 | DNMT1 | _TVMNSK[Lac (K)]THPPK_ |
| P26358 | 961 | K | 0.208668928 | Down | DNA (cytosine-5)-methyltransferase 1 OS=Homo sapiens OX=9606 GN=DNMT1 PE=1 SV=2 | DNMT1 | _K[Lac (K)]EPVDEDLYPEHYR_ |
| P26368 | 70 | K | 0.535597881 | Down | Splicing factor U2AF 65 kDa subunit OS=Homo sapiens OX=9606 GN=U2AF2 PE=1 SV=4 | U2AF2 | _GAK[Lac (K)]EEHGGLIR_ |
| P26373 | 123 | K | 0.580235116 | Down | Large ribosomal subunit protein eL13 OS=Homo sapiens OX=9606 GN=RPL13 PE=1 SV=4 | RPL13 | _SK[Lac (K)]LILFPR_ |
| P26373 | 200 | K | 0.580876428 | Down | Large ribosomal subunit protein eL13 OS=Homo sapiens OX=9606 GN=RPL13 PE=1 SV=4 | RPL13 | _AK[Lac (K)]EAAEQDVEK_ |
| P26583 | 30 | K | 1.94878427 | Up | High mobility group protein B2 OS=Homo sapiens OX=9606 GN=HMGB2 PE=1 SV=2 | HMGB2 | _K[Lac (K)]HPDSSVNFAEFSK_ |
| P26583 | 82 | K | 1.805545953 | Up | High mobility group protein B2 OS=Homo sapiens OX=9606 GN=HMGB2 PE=1 SV=2 | HMGB2 | _NYVPPK[Lac (K)]GDK_ |
| P26599 | 259 | K | 0.57654191 | Down | Polypyrimidine tract-binding protein 1 OS=Homo sapiens OX=9606 GN=PTBP1 PE=1 SV=2 | PTBP1 | _IDFSK[Lac (K)]LTSLNVK_ |
| P26639 | 12 | K | 1.859350772 | Up | Threonine--tRNA ligase 1, cytoplasmic OS=Homo sapiens OX=9606 GN=TARS1 PE=1 SV=3 | TARS1 | _ASSPSGK[Lac (K)]MGGEEKPIGAGEEK_ |
| P26639 | 18 | K | 1.516930683 | Up | Threonine--tRNA ligase 1, cytoplasmic OS=Homo sapiens OX=9606 GN=TARS1 PE=1 SV=3 | TARS1 | _ASSPSGKMGGEEK[Lac (K)]PIGAGEEK_ |
| P26885 | 103 | K | 2.79696774 | Up | Peptidyl-prolyl cis-trans isomerase FKBP2 OS=Homo sapiens OX=9606 GN=FKBP2 PE=1 SV=2 | FKBP2 | _K[Lac (K)]LVIPSELGYGER_ |
| P27348 | 9 | K | 1.707078197 | Up | 14-3-3 protein theta OS=Homo sapiens OX=9606 GN=YWHAQ PE=1 SV=1 | YWHAQ | _TELIQK[Lac (K)]AK_ |
| P27540 | 128 | K | 0.195952939 | Down | Aryl hydrocarbon receptor nuclear translocator OS=Homo sapiens OX=9606 GN=ARNT PE=1 SV=1 | ARNT | _KPDK[Lac (K)]LTILR_ |
| P27694 | 88 | K | 0.662854999 | Down | Replication protein A 70 kDa DNA-binding subunit OS=Homo sapiens OX=9606 GN=RPA1 PE=1 SV=2 | RPA1 | _FIVNTLK[Lac (K)]DGR_ |
| P27694 | 163 | K | 0.372939605 | Down | Replication protein A 70 kDa DNA-binding subunit OS=Homo sapiens OX=9606 GN=RPA1 PE=1 SV=2 | RPA1 | _AYGASK[Lac (K)]TFGK_ |
| P27694 | 167 | K | 0.488704971 | Down | Replication protein A 70 kDa DNA-binding subunit OS=Homo sapiens OX=9606 GN=RPA1 PE=1 SV=2 | RPA1 | _TFGK[Lac (K)]AAGPSLSHTSGGTQSK_ |
| P27816 | 403 | K | 1.809769845 | Up | Microtubule-associated protein 4 OS=Homo sapiens OX=9606 GN=MAP4 PE=1 SV=3 | MAP4 | _ENK[Lac (K)]IVPAK_ |
| P27816 | 838 | K | 0.647331822 | Down | Microtubule-associated protein 4 OS=Homo sapiens OX=9606 GN=MAP4 PE=1 SV=3 | MAP4 | _KPTAIK[Lac (K)]TEGK_ |
| P27816 | 871 | K | 0.45665394 | Down | Microtubule-associated protein 4 OS=Homo sapiens OX=9606 GN=MAP4 PE=1 SV=3 | MAP4 | _K[Lac (K)]TTTLSGTAPAAGVVPSR_ |
| P27816 | 946 | K | 0.5720762 | Down | Microtubule-associated protein 4 OS=Homo sapiens OX=9606 GN=MAP4 PE=1 SV=3 | MAP4 | _VGSTENIK[Lac (K)]HQPGGGR_ |
| P27816 | 1070 | K | 0.634168423 | Down | Microtubule-associated protein 4 OS=Homo sapiens OX=9606 GN=MAP4 PE=1 SV=3 | MAP4 | _AQAK[Lac (K)]VGSLDNVGHLPAGGAVK_ |
| P28290 | 83 | K | 0.588971076 | Down | Protein ITPRID2 OS=Homo sapiens OX=9606 GN=ITPRID2 PE=1 SV=3 | ITPRID2 | _IAIWLK[Lac (K)]DC[Carbamidomethyl (C)]R_ |
| P28290 | 729 | K | 0.626766978 | Down | Protein ITPRID2 OS=Homo sapiens OX=9606 GN=ITPRID2 PE=1 SV=3 | ITPRID2 | _YLFAK[Lac (K)]AGYPLR_ |
| P28290 | 762 | K | 2.851656383 | Up | Protein ITPRID2 OS=Homo sapiens OX=9606 GN=ITPRID2 PE=1 SV=3 | ITPRID2 | _LSPGK[Lac (K)]ETR_ |
| P28340 | 16 | K | 0.292606698 | Down | DNA polymerase delta catalytic subunit OS=Homo sapiens OX=9606 GN=POLD1 PE=1 SV=2 | POLD1 | _RPGPGPGVPPK[Lac (K)]R_ |
| P28347 | 57 | K | 0.290691714 | Down | Transcriptional enhancer factor TEF-1 OS=Homo sapiens OX=9606 GN=TEAD1 PE=1 SV=2 | TEAD1 | _K[Lac (K)]IILSDEGK_ |
| P28347 | 108 | K | 0.34472952 | Down | Transcriptional enhancer factor TEF-1 OS=Homo sapiens OX=9606 GN=TEAD1 PE=1 SV=2 | TEAD1 | _DFHSK[Lac (K)]LK_ |
| P28370 | 59 | K | 0.323755224 | Down | Probable global transcription activator SNF2L1 OS=Homo sapiens OX=9606 GN=SMARCA1 PE=1 SV=2 | SMARCA1 | _EK[Lac (K)]NVSSFQLK_ |
| P28370 | 776 | K | 0.447881199 | Down | Probable global transcription activator SNF2L1 OS=Homo sapiens OX=9606 GN=SMARCA1 PE=1 SV=2 | SMARCA1 | _IPK[Lac (K)]APRPPK_ |
| P28702 | 235 | K | 0.621964766 | Down | Retinoic acid receptor RXR-beta OS=Homo sapiens OX=9606 GN=RXRB PE=1 SV=2 | RXRB | _K[Lac (K)]DLTYSC[Carbamidomethyl (C)]R_ |
| P28749 | 657 | K | 0.146874541 | Down | Retinoblastoma-like protein 1 OS=Homo sapiens OX=9606 GN=RBL1 PE=1 SV=3 | RBL1 | _YSSPTAGSAK[Lac (K)]R_ |
| P28749 | 680 | K | 0.493573412 | Down | Retinoblastoma-like protein 1 OS=Homo sapiens OX=9606 GN=RBL1 PE=1 SV=3 | RBL1 | _IITEGTK[Lac (K)]LK_ |
| P28749 | 770 | K | 0.062016534 | Down | Retinoblastoma-like protein 1 OS=Homo sapiens OX=9606 GN=RBL1 PE=1 SV=3 | RBL1 | _QTNLTK[Lac (K)]AQEVHSTGINRPK_ |
| P28749 | 1015 | K | 0.367699088 | Down | Retinoblastoma-like protein 1 OS=Homo sapiens OX=9606 GN=RBL1 PE=1 SV=3 | RBL1 | _SLK[Lac (K)]DINNMIR_ |
| P29083 | 218 | K | 0.569013067 | Down | General transcription factor IIE subunit 1 OS=Homo sapiens OX=9606 GN=GTF2E1 PE=1 SV=2 | GTF2E1 | _QSK[Lac (K)]DHAATTAGAASLAGGHHR_ |
| P29084 | 24 | K | 0.221294398 | Down | Transcription initiation factor IIE subunit beta OS=Homo sapiens OX=9606 GN=GTF2E2 PE=1 SV=1 | GTF2E2 | _ALSTPVVEK[Lac (K)]R_ |
| P29084 | 74 | K | 0.36705566 | Down | Transcription initiation factor IIE subunit beta OS=Homo sapiens OX=9606 GN=GTF2E2 PE=1 SV=1 | GTF2E2 | _ALSGSSGYK[Lac (K)]FGVLAK_ |
| P29084 | 271 | K | 0.258274083 | Down | Transcription initiation factor IIE subunit beta OS=Homo sapiens OX=9606 GN=GTF2E2 PE=1 SV=1 | GTF2E2 | _FK[Lac (K)]THNEHLAGVLK_ |
| P29084 | 282 | K | 0.244263947 | Down | Transcription initiation factor IIE subunit beta OS=Homo sapiens OX=9606 GN=GTF2E2 PE=1 SV=1 | GTF2E2 | _THNEHLAGVLK[Lac (K)]DYSDITSSK_ |
| P29353 | 321 | K | 0.401372691 | Down | SHC-transforming protein 1 OS=Homo sapiens OX=9606 GN=SHC1 PE=1 SV=4 | SHC1 | _NPPK[Lac (K)]LVTPHDR_ |
| P29374 | 800 | K | 0.388184053 | Down | AT-rich interactive domain-containing protein 4A OS=Homo sapiens OX=9606 GN=ARID4A PE=1 SV=3 | ARID4A | _TK[Lac (K)]DLSLEIIK_ |
| P29375 | 216 | K | 0.443660898 | Down | Lysine-specific demethylase 5A OS=Homo sapiens OX=9606 GN=KDM5A PE=1 SV=3 | KDM5A | _MNILPK[Lac (K)]R_ |
| P29375 | 1422 | K | 0.505385756 | Down | Lysine-specific demethylase 5A OS=Homo sapiens OX=9606 GN=KDM5A PE=1 SV=3 | KDM5A | _K[Lac (K)]SPLVPR_ |
| P29401 | 6 | K | 2.040503868 | Up | Transketolase OS=Homo sapiens OX=9606 GN=TKT PE=1 SV=3 | TKT | _[Acetyl (Protein N-term)]MESYHK[Lac (K)]PDQQK_ |
| P29401 | 232 | K | 1.831793718 | Up | Transketolase OS=Homo sapiens OX=9606 GN=TKT PE=1 SV=3 | TKT | _AFGQAK[Lac (K)]HQPTAIIAK_ |
| P29401 | 260 | K | 2.300423418 | Up | Transketolase OS=Homo sapiens OX=9606 GN=TKT PE=1 SV=3 | TKT | _ESWHGK[Lac (K)]PLPK_ |
| P29401 | 310 | K | 0.60474721 | Down | Transketolase OS=Homo sapiens OX=9606 GN=TKT PE=1 SV=3 | TKT | _MPSLPSYK[Lac (K)]VGDK_ |
| P29401 | 319 | K | 2.289415324 | Up | Transketolase OS=Homo sapiens OX=9606 GN=TKT PE=1 SV=3 | TKT | _K[Lac (K)]AYGQALAK_ |
| P29590 | 401 | K | 0.608232381 | Down | Protein PML OS=Homo sapiens OX=9606 GN=PML PE=1 SV=3 | PML | _K[Lac (K)]ASPEAASTPR_ |
| P29590 | 476 | K | 0.435063693 | Down | Protein PML OS=Homo sapiens OX=9606 GN=PML PE=1 SV=3 | PML | _GPSYGEDVSNTTTAQK[Lac (K)]R_ |
| P29590 | 490 | K | 0.460714662 | Down | Protein PML OS=Homo sapiens OX=9606 GN=PML PE=1 SV=3 | PML | _VIK[Lac (K)]MESEEGK_ |
| P29692 | 117 | K | 1.76352615 | Up | Elongation factor 1-delta OS=Homo sapiens OX=9606 GN=EEF1D PE=1 SV=5 | EEF1D | _LNVLEK[Lac (K)]SSPGHR_ |
| P29692 | 242 | K | 0.355620399 | Down | Elongation factor 1-delta OS=Homo sapiens OX=9606 GN=EEF1D PE=1 SV=5 | EEF1D | _K[Lac (K)]LQIQC[Carbamidomethyl (C)]VVEDDK_ |
| P30101 | 130 | K | 2.504558561 | Up | Protein disulfide-isomerase A3 OS=Homo sapiens OX=9606 GN=PDIA3 PE=1 SV=4 | PDIA3 | _K[Lac (K)]QAGPASVPLR_ |
| P30101 | 226 | K | 2.908643285 | Up | Protein disulfide-isomerase A3 OS=Homo sapiens OX=9606 GN=PDIA3 PE=1 SV=4 | PDIA3 | _TVAYTEQK[Lac (K)]MTSGK_ |
| P30414 | 182 | K | 0.515071112 | Down | NK-tumor recognition protein OS=Homo sapiens OX=9606 GN=NKTR PE=1 SV=2 | NKTR | _SIK[Lac (K)]DVFEK_ |
| P30414 | 328 | K | 0.282327291 | Down | NK-tumor recognition protein OS=Homo sapiens OX=9606 GN=NKTR PE=1 SV=2 | NKTR | _IPDVAPIVSDQKPSVSK[Lac (K)]SGR_ |
| P30414 | 388 | K | 0.636688954 | Down | NK-tumor recognition protein OS=Homo sapiens OX=9606 GN=NKTR PE=1 SV=2 | NKTR | _GDK[Lac (K)]LSDPC[Carbamidomethyl (C)]SSR_ |
| P30414 | 581 | K | 0.376482822 | Down | NK-tumor recognition protein OS=Homo sapiens OX=9606 GN=NKTR PE=1 SV=2 | NKTR | _TASQLSENKPVK[Lac (K)]TEPLR_ |
| P30414 | 1216 | K | 0.272372133 | Down | NK-tumor recognition protein OS=Homo sapiens OX=9606 GN=NKTR PE=1 SV=2 | NKTR | _EVAEK[Lac (K)]SQINLIDK_ |
| P30414 | 1224 | K | 0.549755905 | Down | NK-tumor recognition protein OS=Homo sapiens OX=9606 GN=NKTR PE=1 SV=2 | NKTR | _SQINLIDK[Lac (K)]K_ |
| P30414 | 1271 | K | 0.389318319 | Down | NK-tumor recognition protein OS=Homo sapiens OX=9606 GN=NKTR PE=1 SV=2 | NKTR | _NK[Lac (K)]VRPGSLFDEVR_ |
| P30519 | 259 | K | 0.319453333 | Down | Heme oxygenase 2 OS=Homo sapiens OX=9606 GN=HMOX2 PE=1 SV=2 | HMOX2 | _ETLEDGFPVHDGK[Lac (K)]GDMR_ |
| P30519 | 264 | K | 0.333046533 | Down | Heme oxygenase 2 OS=Homo sapiens OX=9606 GN=HMOX2 PE=1 SV=2 | HMOX2 | _K[Lac (K)]C[Carbamidomethyl (C)]PFYAAEQDK_ |
| P31040 | 250 | K | 5.86861845 | Up | Succinate dehydrogenase [ubiquinone] flavoprotein subunit, mitochondrial OS=Homo sapiens OX=9606 GN=SDHA PE=1 SV=2 | SDHA | _AK[Lac (K)]NTVVATGGYGR_ |
| P31350 | 364 | K | 0.351359492 | Down | Ribonucleoside-diphosphate reductase subunit M2 OS=Homo sapiens OX=9606 GN=RRM2 PE=1 SV=1 | RRM2 | _TNFFEK[Lac (K)]R_ |
| P31942 | 97 | K | 0.536694073 | Down | Heterogeneous nuclear ribonucleoprotein H3 OS=Homo sapiens OX=9606 GN=HNRNPH3 PE=1 SV=2 | HNRNPH3 | _SEIK[Lac (K)]GFYDPPR_ |
| P31943 | 35 | K | 0.384923682 | Down | Heterogeneous nuclear ribonucleoprotein H OS=Homo sapiens OX=9606 GN=HNRNPH1 PE=1 SV=4 | HNRNPH1 | _FFSDC[Carbamidomethyl (C)]K[Lac (K)]IQNGAQGIR_ |
| P31943 | 200 | K | 0.498825316 | Down | Heterogeneous nuclear ribonucleoprotein H OS=Homo sapiens OX=9606 GN=HNRNPH1 PE=1 SV=4 | HNRNPH1 | _K[Lac (K)]LMAMQR_ |
| P31946 | 13 | K | 1.777029471 | Up | 14-3-3 protein beta/alpha OS=Homo sapiens OX=9606 GN=YWHAB PE=1 SV=3 | YWHAB | _AK[Lac (K)]LAEQAER_ |
| P31946 | 160 | K | 1.66972348 | Up | 14-3-3 protein beta/alpha OS=Homo sapiens OX=9606 GN=YWHAB PE=1 SV=3 | YWHAB | _K[Lac (K)]EMQPTHPIR_ |
| P31948 | 78 | K | 0.579325836 | Down | Stress-induced-phosphoprotein 1 OS=Homo sapiens OX=9606 GN=STIP1 PE=1 SV=1 | STIP1 | _K[Lac (K)]AAALEFLNR_ |
| P31948 | 100 | K | 3.939645404 | Up | Stress-induced-phosphoprotein 1 OS=Homo sapiens OX=9606 GN=STIP1 PE=1 SV=1 | STIP1 | _TYEEGLK[Lac (K)]HEANNPQLK_ |
| P31948 | 395 | K | 1.5889068 | Up | Stress-induced-phosphoprotein 1 OS=Homo sapiens OX=9606 GN=STIP1 PE=1 SV=1 | STIP1 | _DAK[Lac (K)]LYSNR_ |
| P31948 | 523 | K | 3.330640832 | Up | Stress-induced-phosphoprotein 1 OS=Homo sapiens OX=9606 GN=STIP1 PE=1 SV=1 | STIP1 | _DPQALSEHLK[Lac (K)]NPVIAQK_ |
| P31949 | 3 | K | 1.505184069 | Up | Protein S100-A11 OS=Homo sapiens OX=9606 GN=S100A11 PE=1 SV=2 | S100A11 | _[Acetyl (Protein N-term)]AK[Lac (K)]ISSPTETER_ |
| P32119 | 196 | K | 2.61490203 | Up | Peroxiredoxin-2 OS=Homo sapiens OX=9606 GN=PRDX2 PE=1 SV=5 | PRDX2 | _EYFSK[Lac (K)]HN_ |
| P32780 | 305 | K | 0.623759599 | Down | General transcription factor IIH subunit 1 OS=Homo sapiens OX=9606 GN=GTF2H1 PE=1 SV=1 | GTF2H1 | _ENSNAAIIK[Lac (K)]R_ |
| P32780 | 370 | K | 0.256573723 | Down | General transcription factor IIH subunit 1 OS=Homo sapiens OX=9606 GN=GTF2H1 PE=1 SV=1 | GTF2H1 | _NNSVK[Lac (K)]TIALNLK_ |
| P32969 | 121 | K | 0.6550765 | Down | Large ribosomal subunit protein uL6 OS=Homo sapiens OX=9606 GN=RPL9 PE=1 SV=1 | RPL9 | _NFLGEK[Lac (K)]YIR_ |
| P32969 | 174 | K | 0.661285027 | Down | Large ribosomal subunit protein uL6 OS=Homo sapiens OX=9606 GN=RPL9 PE=1 SV=1 | RPL9 | _K[Lac (K)]FLDGIYVSEK_ |
| P33992 | 702 | K | 1.731572531 | Up | DNA replication licensing factor MCM5 OS=Homo sapiens OX=9606 GN=MCM5 PE=1 SV=5 | MCM5 | _QK[Lac (K)]YPEHAIHK_ |
| P33993 | 596 | K | 0.523055726 | Down | DNA replication licensing factor MCM7 OS=Homo sapiens OX=9606 GN=MCM7 PE=1 SV=4 | MCM7 | _EAWASK[Lac (K)]DATYTSAR_ |
| P34932 | 272 | K | 2.010362906 | Up | Heat shock 70 kDa protein 4 OS=Homo sapiens OX=9606 GN=HSPA4 PE=1 SV=4 | HSPA4 | _LSQEC[Carbamidomethyl (C)]EK[Lac (K)]LK_ |
| P35221 | 889 | K | 0.113564579 | Down | Catenin alpha-1 OS=Homo sapiens OX=9606 GN=CTNNA1 PE=1 SV=1 | CTNNA1 | _K[Lac (K)]HVNPVQALSEFK_ |
| P35226 | 235 | K | 0.250380425 | Down | Polycomb complex protein BMI-1 OS=Homo sapiens OX=9606 GN=BMI1 PE=1 SV=2 | BMI1 | _MK[Lac (K)]ISHQR_ |
| P35232 | 202 | K | 1.843704444 | Up | Prohibitin 1 OS=Homo sapiens OX=9606 GN=PHB1 PE=1 SV=1 | PHB1 | _FVVEK[Lac (K)]AEQQK_ |
| P35241 | 253 | K | 3.189420144 | Up | Radixin OS=Homo sapiens OX=9606 GN=RDX PE=1 SV=1 | RDX | _NISFNDK[Lac (K)]K_ |
| P35241 | 262 | K | 1.918939951 | Up | Radixin OS=Homo sapiens OX=9606 GN=RDX PE=1 SV=1 | RDX | _FVIKPIDK[Lac (K)]K_ |
[truncated: 537,672 more chars]
